# Multi-centre, randomised, open-label, blinded endpoint assessed, trial of corticosteroids plus intravenous immunoglobulin (IVIG) and aspirin, versus IVIG and aspirin for prevention of coronary artery aneurysms (CAA) in Kawasaki disease (KD): the KD-CAA prevention (KD-CAAP) trial

**DOI:** 10.1016/j.eclinm.2026.104044

**Published:** 2026-07-13

**Authors:** Despina Eleftheriou, Roisin Connon, Robert Tulloh, Neil Martin, Filip Kucera, Georgi Christov, Robin Kahn, Maria Mossberg, Charlotta Nordenhäll, Marion Bailhache, Sauli Palmu, Carlotta Webb, Brian McCrossan, Jethro Herberg, Yolanda Collaco Moraes, Rachael McCormack, John Stone, Rachael Hunter, Ann Sarah Walker, Paul Brogan, KD-CAAP trial investigators, Marta Merida Morillas, Molly Pursell, Charalampia Papadopoulou, Muthana AlObaidi, Elena Moraitis, Jo Walsh, Kirsty McLellan, Maria Ilina, Marc Jansen, Mildred Iro, Wendy Dewals, Mari Laan, Lonneke Van Onzenoort, Daniel Langer, Will Coles, Stephan Paulus, Eleri Williams, Esmeralda Núñez-Cuadros, Jordi Antón, Olof Hertting, Lien De Somer, Stefan Berg, Joseph Panzer, Ilse Meerschaut, Piia Jõgi, Frédéric Huet, Eric Jeziorski, Ulrich von Both, Andre Jakob, Tobias Krickau, Silvia Rosina, Gabrielle Simonini, Andrea Taddio, Elisa Fernandez Cooke, Owen Miller, Elinor Sefi, Michael Levin, Veronica Swallow, Mandy Wan, Joseph Standing, Kimberley Pang, James Wyncoll

**Affiliations:** a UCL Great Ormond Street Institute of Child Health, London, UK; b Medical Research Council (MRC) Clinical Trials Unit (CTU) at University College London (UCL), London, UK; c Bristol Heart Institute, Bristol, UK; d Royal Hospital for Children, Glasgow, UK; e Great Ormond Street Hospital, London, UK; f Department of Paediatrics, Lund University, Clinical Sciences, Lund, Sweden; g Wallenberg Centre of Molecular Medicine, Lund University, Lund, Sweden; h Department of Clinical Science and Education, Karolinska Institute, Södersjukhuset, Sweden; i University Hospital of Bordeaux, Pole de Pediatrie, France; j Tampere Centre for Child, Adolescent and Maternal Health Research, Faculty of Medicine and Health Technology, Tampere University and University Hospital, Tampere, Finland; k Skåne University Hospital Malmö, Sweden; l Paediatric Cardiology, Royal Belfast Hospital for Sick Children, Belfast, Ireland; m Department of Infectious Disease - Faculty of Medicine, Imperial College London, UK; n Societi Foundation CIO, The UK Foundation for Kawasaki Disease, Newark, UK; o Massachusetts General Hospital, Harvard Medical School, Boston, MA, USA; p Health Economics Applied Health Research, UCL, London, UK

**Keywords:** Kawasaki disease, Coronary artery aneurysms, Prednisolone

## Abstract

**Background:**

Kawasaki disease (KD) is a childhood vasculitis affecting medium-sized arteries, particularly the coronary arteries. Despite treatment with intravenous immunoglobulin (IVIG), coronary artery aneurysm (CAA) rates remain high in Europe and North America. The KD-CAAP trial evaluated whether adjunctive prednisolone reduces CAA in unselected European children with KD.

**Methods:**

This multicentre, randomised, open-label, blinded endpoint-assessed, superiority trial (ISRCTN71987471) enrolled children aged 30 days to 16 years across 59 centres in 12 European countries. Participants were randomised 1:1 to oral prednisolone (2 mg/kg/day) plus IVIG (2 g/kg) and aspirin (experimental group); or IVIG and aspirin (control group), stratified by age (<1 vs ≥1 year), sex and country. Co-primary outcomes were: CAA within 12 weeks and mean maximum coronary artery z-score across weeks 1–6, analysed using intention-to-treat.

**Findings:**

Between Jan 2021–July 2024, 103 children (58% male; median age 2 years) were randomised: 50 to experimental and 53 to control groups. All children received IVIG + aspirin; fewer experimental participants received a second IVIG dose [9 (18%) vs 20 (38%) control, p = 0.021] or rescue therapy [8 (16%) vs 17 (32%), respectively, p = 0.044]. CAA occurred in 12/50 (24%) experimental vs 12/53 (23%) control participants (adjusted risk difference +1.1% (95% credibility interval −13.8%–16.1%), with 45% probability of benefit. There was no evidence of difference in mean maximum coronary z-scores over weeks 1–6 (mean 0.6 (95% confidence interval 0.4–0.9) vs 0.7 (0.4–0.9); adjusted difference −0.0; 95% confidence interval −0.2 to +0.2; p = 0.72). CAA developed in 6/12 (50%) infants <1 year. Serious Adverse Events occurred in 7 (14%) experimental vs 3 (6%) control participants (p = 0.19). Costs were significantly lower in the experimental group due to less IVIG use over 12 weeks.

**Interpretation:**

Prednisolone reduced treatment escalation, with potential health economic benefits, but did not reduce CAA in unselected European children with KD. CAA rates remained high, particularly in infants.

**Funding:**

Innovative Medicines Initiative grant 777389.

**Research in context**

**Evidence before this study**

We searched PubMed, Embase, and the Cochrane Central Register of Controlled Trials (CENTRAL) for studies from database inception to Feb 2026, without language restrictions. Search terms included “Kawasaki disease,” “coronary artery aneurysm,” “IVIG resistance,” “adjunctive corticosteroids,” “prednisolone,” and “randomised controlled trial.” We also reviewed references in relevant reviews and guidelines. Eligible studies included randomised trials or observational studies investigating the impact of corticosteroids, in addition to intravenous immunoglobulin (IVIG) and aspirin, on coronary artery outcomes in children with Kawasaki disease (KD). Most randomised evidence demonstrating corticosteroid benefit comes from East Asian populations, using risk scores such as Kobayashi to identify patients at high risk of IVIG resistance. Meta-analyses confirm that early adjunctive corticosteroids reduce CAA risk in these groups. However, these risk scores perform poorly in Western populations, and few randomised data exist for unselected children outside East Asia. Emerging European reports also suggest higher than expected CAA rates, particularly in infants, despite timely IVIG treatment. No large trials have tested the effectiveness of adjunctive corticosteroid use in unselected European KD patients.

**Added value of this study**

KD-CAAP is the first multicentre randomised trial in unselected European KD populations to evaluate whether adjunctive corticosteroids (oral prednisolone) reduce CAA incidence when given alongside IVIG and aspirin in acute KD. In a diverse sample of patients recruited from 59 centres across 12 countries, adjunctive prednisolone did not reduce the incidence of CAA or mean coronary artery Z-scores compared to standard therapy. However, prednisolone use was associated with less frequent use of second-line therapies suggesting benefit in limiting treatment escalation and was also associated with significantly lower costs related to reduced IVIG use.

**Implications of all the available evidence**

Despite timely IVIG treatment, CAA rates remain high in European children with KD, especially in infants under one year. Adjunctive corticosteroids do not reduce CAA risk in an unselected population but may help prevent escalation to second-line therapies, offering clinical value and reducing costs. These results contribute to the evolving evidence base for KD treatment in Western populations but highlight the urgent need for better risk stratification tools and more effective CAA prevention strategies.

## Introduction

Kawasaki disease (KD) is an acute, self-limiting inflammatory vasculitis of childhood that predominantly affects medium-sized arteries, particularly the coronaries.^1^, ^2^, ^3^ It is the leading cause of acquired heart disease in children in high-income countries and although uncommon, can result in death due to coronary complications.^1^, ^2^, ^3^ KD occurs globally with seasonal peaks in winter and spring, and a male predominance (male: female ratio ∼1.5:1).^4^^,^^5^ Incidence varies geographically: Japan reports the highest incidence at 308 per 100,000 children <5 years versus 9.1–14.7 across European countries.^4^ Recent data from England (2006–2021) reported an incidence of 8.9/100,000 children <5 years, with higher rates among children of Asian and Black African descent.^5^

Treatment with intravenous immunoglobulin (IVIG; 2 g/kg over 12 h) and aspirin (30–50 mg/kg/day) is the currently recommended first-line treatment for KD.^1^, ^2^, ^3^ Early trials found IVIG lowered coronary artery aneurysm (CAA) occurrence to 4–5% from ∼20% in untreated children; but 20–40% of patients remain IVIG-resistant, a key risk factor for CAA.^6^, ^7^, ^8^ More recent European studies demonstrate persistently high CAA rates despite timely IVIG treatment: 19% in the UK, 22% in Germany, and 16% in Sweden, rising to ≥40% in infants <1 year.^9^, ^10^, ^11^, ^12^ These high reported CAA rates across Europe have prompted concerns about the adequacy of IVIG monotherapy for KD.

Corticosteroids are effective in many forms of vasculitis and may therefore also offer benefit in KD.^13^, ^14^, ^15^ Meta-analyses demonstrate corticosteroids reduce CAA risk in predominantly East Asian patients predicted to be at high risk of IVIG resistance.^14^ A meta-analysis of trials including 2746 KD patients found that early and adequately dosed adjunctive corticosteroids significantly reduced CAA rates (odds ratio (OR) = 0.42, 95% CI 0.27–0.67), with favourable safety profiles.^14^ Similarly, a recent Cochrane review confirmed that corticosteroids given during the acute phase of KD reduced CAA in patients with high risk scores for IVIG resistance (OR = 0.32, 95% CI 0.14–0.75), again without significant adverse events.^15^ This conclusion came with the caveat that difficulties remain applying these results to Western populations where there is no comparable IVIG resistance risk score.^15^^,^^16^

Therefore, equipoise remains about corticosteroid efficacy in preventing CAA in Western KD patients, and lack of accurate risk stratification hampers the ability to identify patients who may benefit most from adjunctive corticosteroid therapy.^1^, ^2^, ^3^^,^^16^ Nevertheless, high CAA rates reported across multiple European studies strongly support considering this population as high risk by default. On this basis, the KD-CAAP trial aimed to evaluate the efficacy and safety of adjunctive prednisolone in combination with IVIG for the prevention of CAA in unselected European KD patients.

## Methods

### Study design

KD-CAAP was an investigator-led, multi-centre, randomised, open-label, blinded-endpoint assessed, superiority trial conducted in 59 hospitals in 12 European countries (ISRCTN71987471; EUDRACT 2019-004433-17). Supported by the pan-European conect4children (c4c) network (https://conect4children.org) the trial was sponsored by University College London (UCL) and coordinated by the Medical Research Council Clinical Trials Unit at UCL (MRC CTU). It received ethical approval from the North East Newcastle & North Tyneside 1 Research Ethics Committee (20/NE/0014), with additional approvals from each participating country's ethics and regulatory authority. We previously published the trial protocol^17^; a full list of participating centres and the final protocol is available at http://kdcaap.mrcctu.ucl.ac.uk and www.mrcctu.ucl.ac.uk/studies/all-studies/k/kd-caap/respectively. The statistical analysis plan is provided as Supplementary Material. RM (co-author) is a patient representative who was involved in the trial design, was a co-applicant on the funding application, facilitated a patient workshop to refine the trial design and conduct, reviewed and edited all patient facing documents, and co-authored the final manuscript reporting the results of the trial.

### Participants

Patients with KD aged 30 days to 16 years inclusive were eligible. KD was defined as per the 2017 American Heart Association criteria^2^: (a) ≥5 days' fever plus ≥4 of 5 clinical features of KD (bilateral conjunctivitis, cervical lymphadenopathy, polymorphous rash, mucosal changes, extremity changes); or (b) < 5 days’ fever but all 5 clinical features of KD; or (c) incomplete KD^1^^,^^2^: fever ≥5 days plus ≥2 clinical features in children >1 year; or fever ≥7 days in infants ≤1 year without other cause; and, for both age groups, C-reactive protein (CRP) ≥30 mg/L or erythrocyte sedimentation rate (ESR) ≥40 mm/h; and either ≥3 supportive laboratory findings (anaemia, thrombocytosis or thrombocytopaenia, hypoalbuminaemia, elevated ALT, leucocytosis, pyuria) or any of the following echocardiographic features suggestive of KD (ventricular dysfunction, mitral regurgitation, pericardial effusion, or coronary dilation with z-score 2–2.5). There was no restriction on days of illness specified in the protocol for inclusion.

European guidelines currently emphasise the importance of not delaying treatment for KD pending echocardiography, since obtaining this often introduces therapeutic delay.^1^ Our trial protocol therefore did not require a baseline echocardiogram before recruitment. If units were able to perform echocardiography prior to randomisation as part of their routine clinical care, patients were only randomised if this revealed no CAA because the goal of KD-CAAP was to prevent CAA, not treat them. Other exclusion criteria were recurrent KD, severe heart failure or cardiogenic shock, congenital coronary abnormalities affecting endpoint assessment, suspected macrophage activation syndrome, IVIG started >24 h before randomisation, hypersensitivity to prednisolone or methylprednisolone or known phenylketonuria in infants <12 weeks (since aspartame is used in some prednisolone formulations), corticosteroid use for >3 days in the 7 days prior to randomisation, previous severe reaction to immunoglobulin, active or recent varicella/influenza exposure in non-immune patients, co-enrolment in another investigational drug trial, and pregnancy or breastfeeding in adolescents. Written informed consent was obtained from parents/legal guardians; assent was obtained from children when appropriate based on age, illness acuity, and local regulations. Sex was self-reported by patients or parents/legal guardians during screening; response options included “male” or “female.” SARS-CoV-2 testing was conducted according to local standards rather than mandated by protocol; patients with suspected multisystem inflammatory system in children (MIS-C)/Paediatric Inflammatory Multisystem Syndrome Temporally associated with SARS-CoV-2 (PIMS/TS) rather than KD were not considered eligible for randomisation based on clinical assessment.

Sex data was as reported by parents with the options male/female. Ethnicity information was collected except in Sweden, where approval was not given to collect these data.

### Randomisation and masking

Participants were randomised 1:1 using a computer-generated minimisation algorithm with a built-in random element via a secure online system. Randomisation was stratified by age (<1 year and ≥1 year), sex and country. Allocation was concealed until eligibility was confirmed through completed screening and randomisation Case Report Forms. Randomisation was performed by delegated staff at the centres or MRC CTU. The trial was open-label; participants, healthcare providers, and statisticians were all unblinded. However, the paediatric cardiologists (RT, GC) reviewing echocardiographic endpoints were blinded to allocation to minimise assessment bias. No unblinding procedures were required.

### Procedures

All participants received standard-of-care: IVIG (2 g/kg) and oral aspirin (40 mg/kg/day), using local hospital pharmacy stock and infusion protocols. Within 24 h of IVIG initiation, participants were randomised to either no additional treatment (control group) or adjunctive corticosteroids (experimental group) given as open-label oral prednisolone (2 mg/kg/day, maximum 80 mg/day) or equivalent intravenous methylprednisolone (1.6 mg/kg/day) if oral intake was not feasible. Once participants were afebrile for ≥48 h, aspirin was reduced to 3–5 mg/kg/day and continued until at least 21 days after fever resolution. Since presence or absence of fever is by itself a crude biomarker for systemic inflammation, we also used CRP to monitor “response or non-response”, in line with contemporary European guidelines (see also Supplementary Methods).^1^ Treatment at days 2 and 5 was therefore adjusted based on temperature and CRP response, allowing second IVIG doses or rescue therapies at investigator discretion. Protocolised prednisolone tapering could begin from day 5 if the patient was afebrile and CRP ≤10 mg/L (Supplementary Tables S1 and S2).

Prednisolone and methylprednisolone were provided in licenced formulations including tablets, soluble tablets, solutions, and injectable preparations. Oral prednisone could substitute prednisolone at the same dose per local practice. Dosing flexibility of x  ± 12.5% (up to 80 mg daily) was allowed. In-hospital administration was supervised; post-discharge adherence was supported with caregiver education, patient medication diaries, and clinical follow-up. IVIG formulations varied by centre but had to meet European medicinal product standards and were administered as per local protocols. Similarly, no specific aspirin preparation was mandated; centres used locally available stock as per routine clinical practice. Proton pump inhibitors were recommended until prednisolone dose was ≤10 mg/day (or 0.15 mg/kg/day) but not mandated. Other than aspirin, use of non-steroidal anti-inflammatory drugs was not permitted.

Participants were assessed daily through Day-5 and at Weeks 1, 2, 6, and 12 post-randomisation with final follow-up at Week-12. Visit windows were ±12 h for Days 1–5; −1 to +3 days for Week-1; ±3 days for Week-2; and ±14 days for Weeks 6 and 12. Each visit included clinical review: history, KD-focused physical examination, vital signs, temperature, medication use, and adverse events. Height was measured at Day-0 and Weeks 1, 2, 6, and 12; weight at Day-0, Week-6, and Week-12. For febrile (temperature ≥38 °C) inpatients on Day-5, daily maximum temperatures were recorded until afebrile for 48 h. Cardiac monitoring (ECG and echocardiography (details in Supplementary Methods)) was done at Weeks 1, 2, 6, and 12, with coronary artery assessment of the left main coronary artery (LMCA), left anterior descending (LAD), and right coronary artery (RCA). Secondary cardiac findings included aneurysm size/morphology, thrombi, valve regurgitation, and pericardial effusion. Corticosteroid toxicity was assessed using the Paediatric Glucocorticoid Toxicity Index (pGTI) at Weeks 1 and 12 in all participants regardless of receipt of steroids,^18^ in addition to standard adverse event reporting procedures. Health economic and quality of life (QoL) data were collected through resource use and paediatric QoL questionnaires at multiple timepoints.^19^^,^^20^ Laboratory tests (full blood count, ESR, CRP, biochemistry, liver function, glucose) were done at every visit. Unscheduled visits occurred as needed.

### Outcomes

KD-CAAP had two co-primary outcome measures, centrally assessed by two cardiologists (RT, GC) blinded to treatment allocation; any CAA documented within the 12 weeks’ follow-up and the mean maximum z-score of the internal diameters of the proximal right coronary artery or left anterior descending coronary artery pooled across weeks 1, 2, and 6, adjusting for rescue treatment (not including week 12 since the greatest differences were expected in the first 6 weeks). CAA was defined as any of: luminal diameter >3.0 mm in a child <5 years; luminal diameter >4.0 mm in a child/adolescent ≥5 years; internal diameter of a segment at least 1.5 times that of an adjacent segment or when a luminal contour is clearly irregular; or luminal internal diameter z-score of ≥2.5 (see attached manual of operations for echocardiography and image upload). Coronary artery z-scores were derived for primary analyses using normative data from www.parameterz.com/refs/lopez-circimaging-2017.^21^ Sensitivity analyses estimated coronary artery z-scores using normative data from other commonly used systems.^22^, ^23^, ^24^, ^25^

Secondary efficacy outcomes were the individual z-score measurements at weeks 1, 2, 6, and 12 (adjusted for rescue therapy), CAA defined strictly by z-score ≥2.5, proportion receiving rescue therapy or second IVIG dose by Week-12, duration of fever from enrolment, mean daily CRP levels, time to CRP normalisation (≤10 mg/L) and duration of hospitalisation. Secondary safety outcomes were serious adverse events (SAEs) (including deaths), grade 3/4 adverse events (AEs) and AEs of any grade judged related to IVIG, aspirin, or corticosteroids. Other outcomes were inflammatory markers (haemoglobin (Hb), total white blood cell count (WBC), platelets, ESR, albumin), duration and cumulative dose of corticosteroids, proportion remaining on prednisolone 2 mg/kg/day beyond Day-5, paediatric QoL scores (change from baseline to Week-12).^19^ AEs were graded using the 2017 Division of AIDS Toxicity Grading Scale v2.1.

Corticosteroid-related morbidity was assessed using the pGTI.^18^ This is a clinician-facing instrument that is weighted and quantitative. pGTI measures the change in toxicity, thus requiring evaluation at two time points (see pGTI Supplemental Material). In KDCAAP, the glucose, lipid and bone domains of the pGTI were excluded for pragmatic reasons including issues of blood volume obtained for research in young children, and need for DEXA scanning. This modified pGTI derives two scores: the cumulative worsening score (CWS) which ranges from 0 to 412, higher scores denoting greater toxicity; and the aggregate improvement score (AIS) which ranges from 412 to −412, higher negative scores indicating greater improvement in baseline toxicity. The AIS and CWS values from baseline to week 1 were added to the corresponding values for the interval from week 1 to week 12 to obtain the total value for each score.

Health economic outcomes were the Child Health Utility Index 9 dimension (CHU-9D) and associated preference-based algorithm^20^; self-reported health care resource use at week 12 and medication costs available from the trial assessments.

### Statistical analysis

The trial was powered to detect a reduction in CAA from 20% to 8%, requiring 262 participants (80% power, α = 0.05). There were no data to inform what effect estimate could be anticipated on the continuous Z-score co-primary endpoint, nor its standard deviation, and therefore the sample size was based on the binary CAA endpoint. The protocol specified that the two co-primary endpoints were to be considered separately, each with a nominal 0.05 level of significance, reflecting the fact that KD is a relatively rare disease in which it is important to generate randomised unbiased evidence and consider its totality.^3^^,^^26^ However, due to delays in centre activation and slow recruitment, given the fixed funding end date without possibility of extension, 103 children were ultimately enrolled (details in Supplementary Methods). Analyses followed a pre-specified statistical analysis plan approved by all oversight committees. The primary analysis was intention-to-treat including all participants based on observed CAA over the study period; for laboratory values and coronary Z-scores, analyses used all available data (complete cases). Binary outcomes were analysed with exact tests and logistic regression adjusted for the stratification factors age and sex, and country in Bayesian analyses (too few outcomes to also adjust for 12 countries in frequentist analyses). Risk differences with 95% confidence intervals were calculated using marginal effects. As the final achieved sample size was <80% target, the primary analysis was Bayesian conducted using uninformative (primary), sceptical, and optimistic priors, reporting the posterior probability of benefit (details in Supplementary Methods): all other analyses were frequentist. Continuous outcomes were analysed using linear regression and generalised estimating equations (independent correlation structure), adjusting for all stratification factors (age, sex and country). Models for the continuous co-primary outcome also adjusted for baseline z-score and, in a more principled version of a standard per-protocol analysis (which introduces bias as it relies on post-randomisation exclusion of participants),^27^ for rescue treatment using inverse probability of treatment weights (IPTW), censoring children when they started rescue treatment and using probability weights to upweight comparable children who did not start rescue treatment. Time-to-event outcomes were analysed using cumulative incidence and Cox regression, adjusted for age, sex and country. Where continuous outcomes had gross evidence of non-normality (assessed by Shapiro–Wilk tests and visual inspection), they were transformed for normality and back-transformed for reporting using either Boxcox transformations (if positive) or log(X + k) (Stata lnskew). For the co-primary outcomes, subgroup analyses examined age, sex, fever duration, and baseline z-score, testing heterogeneity with interaction p-values. Bayesian analyses were performed in R version 4.4.2 using the brms and brmsmargins packages.^28^ All other analyses used Stata version 18.5. The independent Data Monitoring Committee reviewed unblinded interim data at least annually (4 meetings). Early stopping for benefit used a Haybittle-Peto threshold (p < 0.001); no futility stopping rule was applied.

The economic analysis used the CHU-9D to calculate the mean incremental cost per quality-adjusted life-year (QALY) gained of adjunct corticosteroids compared to standard care over 12 weeks. QALYs were calculated using the area-under-the-curve method adjusting for baseline using participant-level utility scores. Resource use was costed in 2023–24 British pounds using unit costs from the Personal Social Services Research Unit.^29^ Medication was costed from the British National Formulary.^30^ MissForest (v4.2.3) was used to impute missing CHU-9D scores.^31^^,^^32^ The fit transform method was used for imputation. Further details can be found at: https://pypi.org/project/MissForest/. Regression and bootstrapping of complete cases was used to calculate 95% CIs, cost-effectiveness acceptability curves, and cost-effectiveness planes. The economic evaluation was conducted in Python 3.12.

### Role of the funding source

The funder had no role in study design, data collection, data analysis, data interpretation, or writing of this report.

## Results

**Fig. 1:**
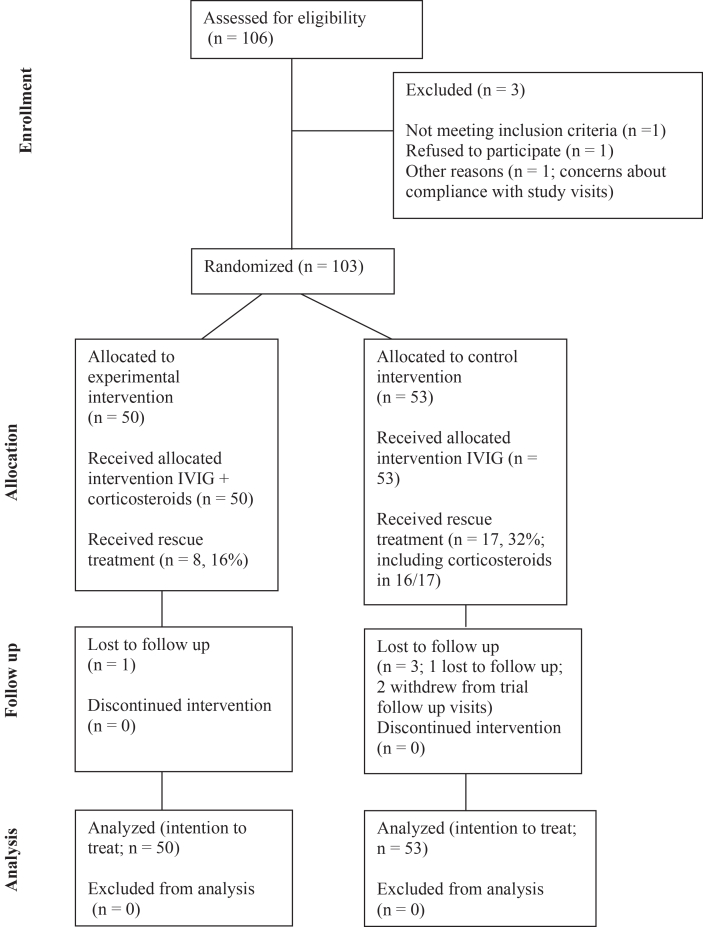
A total of 469 children were informally pre-screened using notes review. 106 children were formally assessed for eligibility between January 2, 2021 and July 11, 2024 based on completion of the screening CRF. IVIG = intravenous immunoglobulin. See Supplementary Tables S4 and S5 for details of additional IVIG and rescue medication as allowed by the protocol.

Participants were recruited between January 2, 2021, and July 11, 2024 (recruitment by country shown in Supplementary Figure S1). Of 106 children formally screened, 103 were randomised: 50 to the experimental group (IVIG + aspirin + corticosteroids) and 53 to the control group (IVIG + aspirin) (Fig. 1). Baseline characteristics were broadly balanced between groups (Table 1). Twelve (12%) children were under 1 year. Only 7 (7%) children had an incomplete KD diagnosis. Median (IQR) CRP was 121 (79, 205) mg/L and children had been febrile for a median (IQR) 7 (6–9) days. 58 (56%) children had a (non-protocolised) echocardiogram done before randomisation (not all results returned before randomisation occurred).

**Table 1:** Baseline characteristics.

Characteristic	Experimental group (n = 50)	Control group (n = 53)
Sex (male)	29 (58%)	31 (58%)
Age (years)	2 (1, 4)	2 (1, 3)
Age <1 year^a^	6 (12%)	6 (11%)
Age ≥1 years	44 (88%)	47 (89%)
Ethnicity – White	24 (48%)	27 (51%)
Ethnicity – Asian	7 (14%)	5 (10%)
Ethnicity – Black	7 (14%)	4 (8%)
Ethnicity – Mixed	2 (4%)	7 (13%)
Ethnicity – Unknown	9 (18%)	7 (13%)
Ethnicity – Other	1 (2%)	2 (4%)
Weight in kg	15 (13, 18)	14 (11, 17)
Complete KD	47 (94%)	49 (92%)
Incomplete KD	3 (6%)	4 (8%)
Duration of fever at enrolment (days)^b^	7 (6, 8)	7 (6, 9)
CRP mg/L [N = 102]	148 (86, 220)	112 (70, 194)
Albumin (g/L) [N = 90]	29 (24, 31)	30 (25, 34)
Haemoglobin g/L [N = 96]	101 (96, 113)	104 (96, 112)
Platelets (x10^9^/L)	322 (270, 463)	388 (296, 421)
White cell count (x10^9^/L) [N = 96]	13 (9, 15)	14 (10, 17)
ESR (mm/hr) [N = 57]	81 (51, 106)	88 (56, 110)
Neutrophil count (x10^9^/L) [N = 95]	8 (6, 11)	9 (5, 12)
Echocardiogram done before randomisation^c^:	30 (60%)	28 (53%)
Maximum measurement of luminal diameter (mm)	2.4 (2.0, 2.5)	2.3 (2.0, 2.6)
Maximum z-score of luminal diameter	0.7 (0.2, 1.5)	1.5 (0.9, 2.2)

Showing number of participants and percentage for categorical, and median with interquartile range (IQR) for continuous variables. When results were available for a subset of patients rather than the entire cohort, the corresponding number of patients is indicated by N. Approval was not given to collect ethnicity data in Sweden. KD = Kawasaki disease; CRP = C-reactive protein; ESR = erythrocyte sedimentation rate.

a Of the 12 infants under the age of 1 year included, 9 were under the age of 6 months, and 5/9 of those had CAA.

b The range of days of illness was 5–36 in the experimental group and 4–19 in the control group.

c Even if done, not all echocardiography results were available before randomisation occurred (see manuscript main text).

Visit completion was high with 100% completing Day 1 and Day 2 visits and >90% completing subsequent schedule visits (overall only 49/927 (5%) scheduled visits not completed). Two participants were lost to follow-up (one in each group), and two additional participants in the control group withdrew from the trial. No participant died.

All children received initial IVIG; at randomisation 84 (82%) had already initiated IVIG a median 17 (IQR 13–20) hours previously (Supplementary Table S3). All children received aspirin. Nine (18%) experimental vs 20 (38%) control participants received a second IVIG dose (p = 0.021 adjusted for age and sex), 47 (38–105) vs 48 (44–55) hours after randomisation (p = 0.56) (Supplementary Tables S3 and S4). Eight (16%) experimental vs 17 (32%) control participants received formal rescue treatment (p = 0.044; not including protocolled second IVIG doses), a median (IQR) 4 (2–12) vs 2 (1–4) days after randomisation (p = 0.13), including additional non-protocolled IVIG doses, corticosteroids, infliximab, ciclosporin and IL-1 blockade therapy (Supplementary Table S5). In total, 16 (30%) participants in the control group received corticosteroids (methylprednisolone and/or prednisolone) as rescue treatment (Supplementary Table S6).

Most planned echocardiograms were completed (Supplementary Table S7). CAA were observed after randomisation in 12 (24%) experimental vs 12 (23%) control group participants; an adjusted difference of +1.1% in the primary Bayesian analysis with an uninformative prior (95% Credible Interval (CrI) −13.8%, 16.1%), with a 45% probability that the experimental group was superior to control (Table 2 and Supplementary Table S8). Excluding three children with CAA present on the scan taken on the day of randomisation but not identified before randomisation, CAA were observed in 10/48 (21%) experimental vs 11/52 (21%) control participants; an adjusted difference of +0.1% (−15.0%, 15.3%), with a 50% probability that the experimental group was superior (Table 2). Sensitivity analyses using an enthusiastic Bayesian prior increased the benefit associated with corticosteroids as expected, whereas using a sceptical prior was similar to the uninformative prior (Table 2, Supplementary Figure S2). Results were broadly similar defining CAA by a luminal internal diameter z-score ≥2.5 (adjusted difference −3.3%, 95% CI −16.7 to 10.3; 68% probability of benefit), or by any domain of the primary CAA definition (Table 2). There was no difference in CAA severity between groups (Supplementary Table S9).

**Table 2:** Co-primary outcome: coronary artery aneurysms (CAA) rates.

	Experimental group observed (n = 50)	Control group: observed (n = 53)	Bayesian prior	Adjusted difference between groups (experimental - control) (95% credible interval)	Bayesian probability of benefit in experimental group (one-sided)	Frequent-ist p-value (2-sided)
Any CAA [Lopez et al.^21^] (co-primary outcome)	12 (24%)	12 (23%)	**Uninformative**	**1.1% (13.8, 16.1)**	**45%**	0.87
			Enthusiastic	−6.8% (−17.4, 3.8)	90%	
			Sceptical	0.6% (−10.1, 11.3)	46%	
Sensitivity analysis: excluding children with CAA present but not identified before randomisation^a^	10/48 (21%)	11/52 (21%)	Uninformative	0.1% (−15.0, 15.3)	50%	0.98
Luminal internal diameter z-score of ≥2.5 (secondary outcome)	8 (16%)	10 (19%)	Uninformative	−3.3% (−16.7, 10.3)	68%	0.66
CAA defined by absolute luminal diameter (>3 mm if < 5 years; >4 mm if ≥ 5 years)	6 (12%)	6 (11%)	Uninformative	0.6% (−12.5, 14.0)	47%	0.92
Internal diameter of a segment at least 1.5 times that of an adjacent segment	7 (14%)	3 (6%)	Uninformative	8.8% (−3.8, 21.3)	8%	0.19
Luminal contour clearly irregular	7 (14%)	5 (9%)	Uninformative	3.6% (−8.8, 16.1)	28%	0.47
Any CAA [McCrindle et al.^22^]	11 (22%)	10 (19%)	Uninformative	3.4 (−11.3, 18.1)	33%	0.69
Any CAA [Dallaire et al.^23^]	14 (28%)	13 (25%)	Uninformative	6.4 (−8.9, 21.3)	20%	0.67
Any CAA [Oliveri et al.^24^]	10 (20%)	9 (17%)	Uninformative	2.9 (−11.7, 17.3)	35%	0.69
Any CAA [Kobayashi et al.^25^]	15 (30%)	15 (28%)	Uninformative	4.0 (−11.2, 19.6)	31%	0.84
Luminal internal diameter z-score of ≥2.5 [McCrindle et al.^22^]	8 (16%)	7 (13%)	Uninformative	3.0 (−10.5, 16.9)	33%	0.69
Luminal internal diameter z-score of ≥2.5 [Dallaire et al.^23^]	11 (22%)	11 (21%)	Uninformative	4.3 (−10.5, 19.2)	29%	0.87
Luminal internal diameter z-score of ≥2.5 [Oliveri et al.^24^]	7 (14%)	6 (11%)	Uninformative	2.7 (−10.3, 16.0)	34%	0.69
Luminal internal diameter z-score of ≥2.5 [Kobayashi et al.^25^]	12 (24%)	14 (26%)	Uninformative	−0.4 (−15.3, 14.9)	52%	0.76

Primary analysis was a Bayesian logistic regression model, as recruitment was <80% target, adjusted for stratification factors (age group, sex, and country), under three priors: uninformative (primary; analogous to the frequentist analysis shown for comparison), enthusiastic (favouring prednisolone), and sceptical (details in Supplementary Methods). Frequentist p-values from logistic regression adjusted for age group and sex, or using Fisher's exact test where there were <5 events in a group. Primary analysis shown in bold. Uninformative prior for treatment effect N(0, 10000) on logit scale. Enthusiastic prior for treatment effect N(−1.0560527, 0.29030801) on logit scale (equivalent to hypothesised risk difference of a 12% reduction). Sceptical prior for treatment effect N(0, 0.29030801) on logit scale.

a One was detected post randomisation after baseline scan returned, one was classified as CAA at baseline only after central review, one scan result was available before randomisation but was not considered CAA by clinician.

There was also no evidence that the co-primary outcome, mean maximum of RCA/LAD coronary artery z-score at weeks 1, 2, and 6 adjusted for receipt of rescue treatment differed between groups (adjusted difference (experimental vs control) −0.0 (95% CI −0.2 to +0.2); p = 0.72; Table 3). Results were consistent in sensitivity analyses using an alternative IPTW model based on baseline factors only, as well as the unadjusted intention-to-treat analysis and analyses excluding children with CAA present but not identified at randomisation (Table 3).

**Table 3:** Co-primary outcome: mean maximum coronary artery z-scores of RCA or LAD across weeks 1, 2, and 6.

Analysis	Experimental group (n = 50)	Control group (n = 53)	Adjusted difference between groups on modelled scale	p-value
**[Lopez et al.****^21^****] (co-primary outcome) Mean maximum z-score at weeks 1, 2 and 6 adjusted for rescue treatment using IPTW from baseline and time-updated factors**	**0.6 (0.4, 0.9)**	**0.7 (0.4, 0.9)**	**−0.0 (−0.2, 0.2)**	**0.72**
[Lopez et al.^21^] Mean maximum z-score at weeks 1, 2 and 6 adjusted for rescue treatment using IPTW from baseline factors only	0.6 (0.4, 0.9)	0.7 (0.5, 1.0)	−0.1 (−0.2, 0.1)	0.58
[Lopez et al.^21^] Mean maximum z-score at weeks 1, 2 and 6 not adjusted for rescue treatment (intention-to-treat)	0.9 (0.5, 1.3)	0.7 (0.5, 1.0)	0.1 (−0.2, 0.3)	0.61
[Lopez et al.^21^] Mean maximum z-score at weeks 1, 2 and 6 excluding 3 children with CAA detected before randomisation or on day 0 adjusted for rescue treatment using IPTW from baseline and time-updated factors	0.6 (0.4, 0.9)	0.7 (0.4, 0.9)	−0.0 (−0.2, 0.2)	0.74
[Lopez et al.^21^] Mean maximum z-score at week 1 adjusted for rescue treatment as in primary analysis	0.9 (0.5, 1.2)	1.0 (0.6, 1.3)	−0.0 (−0.3, 0.2)	0.69
[Lopez et al.^21^] Mean maximum z-score at week 2 adjusted for rescue treatment as in primary analysis	0.8 (0.4, 1.3)	0.6 (0.2, 1.1)	0.1 (−0.2, 0.4)	0.61
[Lopez et al.^21^] Mean maximum z-score at week 6 adjusted for rescue treatment as in primary analysis	0.2 (−0.1, 0.5)	0.4 (0.1, 0.8)	−0.1 (−0.5, 0.2)	0.37
[Lopez et al.^21^] Mean maximum z-score at week 12 adjusted for rescue treatment as in primary analysis	0.1 (−0.2, 0.4)	0.7 (0.4, 1.1)	−0.4 (−0.7, −0.1)	0.011
McCrindle et al.^22^ Mean maximum z-score at weeks 1, 2 and 6 adjusted for rescue treatment using IPTW from baseline and time-updated factors	−0.0 (−0.3, 0.2)	−0.1 (−0.3, 0.2)	0.0 (−0.2, 0.2)	0.98
Dallaire et al.^23^ Mean maximum z-score at weeks 1, 2 and 6 adjusted for rescue treatment using IPTW from baseline and time-updated factors	0.2 (0.0, 0.4)	0.3 (0.2, 0.6)	−0.1 (−0.2, 0.1)	0.45
Oliveri et al.^24^ Mean maximum z-score at weeks 1, 2 and 6 adjusted for rescue treatment using IPTW from baseline and time-updated factors	−0.0 (−0.2, 0.2)	0.1 (−0.1, 0.3)	−0.0 (−0.1, 0.1)	0.59
Kobayashi et al.^25^ Mean maximum z-score at weeks 1, 2 and 6 adjusted for rescue treatment using IPTW from baseline and time-updated factors	0.3 (0.0, 0.5)	0.4 (0.1, 0.6)	−0.0 (−0.2, 0.1)	0.63

Z-scores were log-transformed to improve normality using the formula ln(x + 1.40) for the Lopez method, ln(x + 2.16) for McCrindle, ln(x + 1.42) for Dallaire, ln(x + 2.75) for Oliveri, ln(x + 2.30) for Kobayashi due to negative values (offset selected to maximise normality); results in each randomised group are back-transformed onto the original absolute z-score scale for interpretability. Primary analysis shown in bold. The “adjusted difference” is the estimated mean difference between treatment groups on the log-transformed scale across weeks 1, 2 and 6 from the adjusted GEE model (details in Methods) with corresponding p-value. IPTW, Inverse Probability of Treatment Weighting. IPTW models adjust for receipt of rescue treatment in a more principled version of a standard per-protocol analysis (which introduces bias as it relies on post-randomisation exclusion of trial participants) (details in Supplementary Methods).

To assess the impact of the z-score normative population on coronary outcomes, a sensitivity analysis compared five commonly used coronary artery z-score equations including the pre-specified primary Lopez et al. reference model.^21^, ^22^, ^23^, ^24^, ^25^ Results were consistent showing no evidence of an effect of corticosteroids on either co-primary outcome (Tables 2 and 3). The adjusted CAA risk differences between groups ranged from 1.1% (95% CI −13.8% to 16.1%) to 6.4% (95% CI −8.9 to 21.3%) across the five z-score models with low posterior probabilities of benefit (20–45%). Similarly, adjusted differences in the mean maximum z-score across weeks 1, 2, and 6, were close to zero across all models, with 95% CI spanning the null and p-values ranging from 0.45 to 0.98. These findings confirm robustness of the primary results regardless of z-score model.

There was no evidence of heterogeneity in the effect of corticosteroids in any prespecified subgroup (by age, sex, or days of fever at randomisation) for either co-primary endpoint (p > 0.2; Supplementary Table S10). CAA developed in 6/12 (50%) infants <1 year: 4/6 infants in the experimental group and 2/6 infants in the control group (interaction p = 0.23).

Median duration of fever after randomisation was slightly shorter in the experimental group (1 day [IQR 1–1]) vs. control (1 day [IQR 1–2]) (hazard ratio (HR) = 1.4 (95% CI 1.1–1.7; p < 0.00062); Supplementary Figure S3a, Supplementary Table S4). Median time to CRP normalisation (≤10 mg/L) was 6 days in both groups (IQR 4–8 vs 4–9; p = 0.39) (Supplementary Figure S3b, Supplementary Table S4). In the experimental group, mean CRP was 0.88 times that of the group across Day-2 to Week 6 (95% CI 0.77–1.00 times (relative difference), p = 0.059, Supplementary Table S4). Median hospital stay was 4 days (IQR 3–6) in the experimental group and 5 days (IQR 4–6) in the control group (p = 0.46) (Supplementary Figure S3c; Supplementary Table S4).

Mean haemoglobin levels increased more over time in the experimental vs control group, with a mean adjusted difference of 3 g/L (95% CI 1–5; p = 0.0089) from Day-2 to Week-6 (Supplementary Table S11). White cell count was also consistently higher in the experimental group, with a mean adjusted difference of 0.3 log_2_ 10^9^/L (95% CI 0.2–0.5; p < 0.0001) over the same time period (Supplementary Table S11). Platelet counts increased by 56 × 10^9^/L more in the experimental group at Week-6 (95% CI 4–108; p = 0.036) (mean 28 × 10^9^/L difference between groups across Day-2 to Week-6 (95% CI −9 to +64, p = 0.13) (Supplementary Table S11). There was no evidence of differences between groups in changes in ESR (p = 0.23 overall) or albumin concentrations (p = 0.89 overall) (Supplementary Table S11).

Overall, 10 participants (10%) experienced at least one SAE: 7 (14%) experimental vs 3 (6%) control participants (p = 0.19) (Table 4, Supplementary Table S12). In total, 16 SAEs were reported (11 experimental vs 5 control): none were fatal or resulted in disability. Most SAEs were considered unrelated or unlikely to be related to corticosteroid use (88%; Supplementary Table S12). Grade 3/4 AEs occurred in 17 (34%) experimental vs 24 (45%) control participants (p = 0.31) and were rarely related to study medications (Table 4). Corticosteroid related AEs of any grade occurred in 5 (10%) experimental vs 0 (0%) control participants (p = 0.024), IVIG-related in 4 (8%) experimental vs 8 (15%) control participants (p = 0.36), and aspirin-related in 2 (4%) experimental vs 4 (8%) control participants (p = 0.68) (Table 4, Supplementary Table S13).

**Table 4:** Summary of adverse events (AEs).

Adverse event	Experimental group (n = 50)	Control group (n = 53)	p-value
SAEs			
Number of children affected (%)	7 (14%)	3 (6%)	0.19
Total number of events	11	5	
Life-threatening	0	2	
Hospitalisation	10	3	
Other important medical condition	1	0	
SAE related to corticosteroids			
Number of children affected (%)	2 (4%)	0 (0%)	0.23
Total number of events	2	0	
SAE related to IVIG			
Number of children affected (%)	0 (0%)	2 (4%)	0.50
Total number of events	0	2	
SAE related to aspirin			
Number of children affected (%)	0 (0%)	0 (0%)	1.00
Total number of events	0	0	
Grade 3 or 4 AEs			
Number of children affected (%)	17 (34%)	24 (45%)	0.31
Total number of events	25	41	
Clinical AE of any grade related to corticosteroids			
Number of children affected (%)	5 (10%)	0 (0%)	0.024
Total number of events	6	0	
Clinical AE of any grade related to IVIG			
Number of children affected (%)	4 (8%)	8 (15%)	0.36
Total number of events	5	16	
Clinical AE of any grade related to aspirin			
Number of children affected (%)	2 (4%)	4 (8%)	0.68
Total number of events	2	6	

P-values from Fisher's exact test. See Supplementary Tables S12 and S13 for adverse event details.

Analysis of the pGTI found no evidence of differences between groups in aggregate improvement score or cumulative worsening score at week 12 (Supplementary Table S14).

There was also no evidence of differences in quality of life at week 12: the mean Paediatric Quality of Life Inventory (PedsQL) was 88.3 (95% CI 84.1–92.6) (n = 32) in the experimental group and 92.7 (95% CI 88.4–96.9) (n = 32) in the control group (adjusted difference −4.3 (95% CI −10.6 to 1.9); p = 0.17).

In the experimental group, the cost of IVIG over 12 weeks was significantly lower in the experimental group (difference −£438 (95% CI −865 to −11; Supplementary Table S15), with no significant difference in other drug or healthcare costs (Supplementary Table S16). There was no significant difference in QALYs measured using the CHU-9D over the 12 weeks of the trial (adjusted difference for imputed data −0.004 (95% CI −0.009 to 0.000 p = 0.072; sensitivity analyses using complete cases or mixed effects models similar, Supplementary Table S17) At a £20,000 decision threshold per QALY gained there was a 70% probability that adjunctive corticosteroids were cost-effective compared to current practice due to the reduced cost of IVIG (Supplemental Figure S4).

## Discussion

Our multicentre, randomised trial addressed a critical gap in the evidence for KD management in Europe. We found that adding corticosteroids (predominantly prednisolone) to standard IVIG and aspirin therapy for patients without known baseline CAA did not reduce the incidence of CAA or mean coronary z-scores by week 12 in unselected KD patients. However, prednisolone significantly reduced administration of second IVIG doses (likely due to significantly faster fever resolution), and significantly reduced the use of rescue therapy, with a favourable safety profile. It also significantly reduced costs. While corticosteroids did not prevent CAA in this population, the reduction in treatment escalation suggests potential clinical and significant economic benefits.

The KD-CAAP trial was conducted in response to growing concerns about persistently high rates of CAA in European children with KD, despite timely treatment with IVIG. While randomised controlled trials (notably the Japanese RAISE trial), meta-analyses, and systematic reviews have consistently demonstrated benefits with adjunctive corticosteroids in East Asian children at high risk of IVIG resistance (often identified by Kobayashi score),^13^^,^^14^ evidence of similar efficacy in unselected or non-East Asian KD populations is limited. Importantly, these risk scores perform poorly in European cohorts, where CAA frequently develop even in children not classified as high risk.^1^^,^^16^ In this context, KD-CAAP tested a universal corticosteroid approach rather than risk-based selection, and found no effect on CAA prevention, and of particular concern, confirmed high rates of CAA (23% overall) irrespective of treatment, in our recruited cohort which was representative of typical European KD patients. This finding is in stark contrast to the perceived efficacy of IVIG for CAA prevention, where it is still frequently stated that without treatment 25% of patients develop CAA,^3^ which reduces to less than 5% with IVIG administration within the first 10 days of illness.^1^ Of note patients in KD-CAAP received treatment at a median day 7 of illness.

Our findings therefore contrast with those of several East Asian trials, such as RAISE, where corticosteroids significantly reduced CAA incidence in high-risk patients.^13^ The reasons for this different outcome are unknown but likely reflect multiple genetic and immunological differences between East Asian and European populations. KD-CAAP confirms that CAA rates remain high in European children, affecting ∼23% overall (regardless of specific definition or z-score model) and ∼50% infants <1 year despite adherence to current treatment protocols, and with no impact of corticosteroids at doses and durations used in our protocol which we aligned with the RAISE trial corticosteroid regimen. Nor did we observe any obvious difference in CAA severity between the two groups (Supplementary Table S8). We note that a follow up study to the RAISE trial in fact found that among predicted IVIG non-responders, adding prednisolone did not significantly impact coronary outcomes, although IVIG non-response was significantly lower in the IVIG plus prednisolone group.^33^

Our results thus confirm reports from the UK, Germany, and Sweden, which show high CAA incidence, particularly in infants under 1 year, challenging the historical view that IVIG alone reliably reduces CAA to <5%.^6^, ^7^, ^8^ Our results also support the American Heart Association's most recent position that further evidence is needed to guide treatment in non-East Asian children and that corticosteroids cannot be recommended for routine use in all patients for coronary disease prevention; however, our findings do support corticosteroid use as a cost-effective mechanism to speed fever resolution and reduce the use of rescue treatment.^3^

Beyond coronary outcomes, our trial demonstrated clinical and economic benefits associated with initial prednisolone use in unselected populations. Corticosteroids reduced the use of a second IVIG dose (18% vs. 38%, p = 0.021), and rescue treatments (16% vs. 32%, p = 0.044). This aligns with a 2024 North American retrospective observational study which reported that, while corticosteroids did not significantly reduce the incidence of CAA in high-risk KD patients, they were associated with reduced use of additional IVIG dosing.^34^ Faster fever resolution with prednisolone likely contributed to reduced IVIG use in our trial. There was no evidence of significant differences in hospital stay and CRP normalisation between groups, although numerically hospital stay was shorter in the experimental group. The reduction in the use of high-cost second-line therapies, particularly additional IVIG, resulted in a 70% probability of that routine corticosteroid use was cost-effective at a £20,000/QALY threshold. This may be particularly important in settings with limited access to IVIG or constrained healthcare resources.

Overall, our findings support the safety of corticosteroid use in KD. Adverse events were infrequent, with no evidence of differences between groups. Corticosteroid-related adverse events were rare, and pGTI findings did not reveal any significant toxicity signal in the experimental group. Overall, no clinically meaningful short-term cardiovascular toxicity attributable to corticosteroids was observed in our trial.

Our findings have important implications for both European and global clinical management of KD. In Europe, where existing risk stratification tools underperform and CAA rates remain high despite timely IVIG, our results challenge the adequacy of a one-size-fits-all approach. While corticosteroids did not reduce CAA incidence in unselected patients, the observed reduction in treatment escalation supports its potential utility in settings where IVIG resistance is common or where delays in access to second-line therapies may occur. Globally, particularly in low-resource settings where IVIG supply may be limited or unaffordable, adjunctive corticosteroids, being inexpensive and widely available, may offer a pragmatic strategy to mitigate disease severity and reduce health system burden. Patients should be counselled, however, that the goal of corticosteroid therapy is symptomatic relief and faster resolution of acute inflammatory disease features, and not reduced incidence of coronary sequelae. Our results also suggest that risk stratification should focus on “risk of aneurysm” rather than “risk of IVIG resistance”, the latter defined by recrudescence of fever following IVIG treatment, since despite more rapid resolution of fever in the prednisolone group, CAA occurrence was unaffected.

A key strength of our study was its pragmatic, real-world design, with broad inclusion criteria, diverse European settings, and central, blinded echocardiographic review. Coronary outcomes were analysed using five validated z-score normative populations strengthening our confidence in the consistency of findings. In addition, we are the first to apply the pGTI in the context of KD, providing structured, prospective assessment of corticosteroid-related adverse effects. Although pGTI is likely to capture toxicity in the context of chronic corticosteroid exposure, the overall favourable safety profile observed with short-course prednisolone was reassuring.

The main limitation of our study is low power due to slower than expected recruitment from delays in centre opening, the result of (in part) bureaucratic legislation inherent in multi-national clinical trials; and also pressures on healthcare due to the COVID-19 pandemic. It is well recognised that low power increases the risk of type II error (false negative), meaning that modest effects of corticosteroids on CAA incidence or coronary artery Z-scores might be undetected. Trials are not typically powered to detect effects on secondary endpoints, and therefore the impact of low power to detect the hypothesised differences in the co-primary endpoints on analyses of secondary endpoints will be variable, depending on the magnitude of difference judged clinically meaningful in these secondary endpoints. Whilst results of smaller trials are not biased on average (unless a decision to stop a trial is made on the basis of the results at that time, or where trial results are only published if the result is “significant”), the increased variability due to low power means that any individual realisation of a comparison between intervention and control which achieves an arbitrary level of significance (e.g. p < 0.05) will overestimate the true difference by a magnitude which depends on power to detect this difference.^35^ Thus it is possible that differences in secondary endpoints reported here overestimate the true differences (type I error). Intention-to-treat analysis and high retention/outcome completeness additionally mitigate potential bias. While the trial was open-label, primary outcomes were centrally assessed blinded to randomised allocation, reducing interpretative bias. It was not practically possible to blind clinicians to every aspect of patient care including administration of second IVIG or rescue therapy, an unavoidable limitation of our pragmatic trial protocol and a potential source of bias regarding these secondary endpoints. Aligned with routine clinical practice across Europe, our trial protocol did not require a baseline echo before recruitment. Some units were nevertheless able to perform echocardiography prior to randomisation (Table 1). These patients were only randomised if the (non-protocolised) baseline echo revealed no CAA. Three patients were subsequently identified as having baseline CAA that had not been recognised before randomisation. However, sensitivity analysis excluding these patients did not change our results.

In conclusion, adjunctive prednisolone did not reduce CAA incidence or improve coronary z-scores in unselected European children with KD but was associated with less frequent use of second-line therapies, health economic benefit, and a favourable safety profile. CAA occurrence remained high, historically comparable to untreated KD, particularly in infants despite timely IVIG treatment emphasising the urgent need for better therapeutic approaches for CAA prevention in KD in European cohorts. Our findings highlight a role for corticosteroids in reducing the burden of acute inflammation in KD but not in CAA prevention. Future research should focus on developing reliable risk stratification tools for Western patients and identify therapies to better prevent CAA.

## Contributors

Despina Eleftheriou, Paul Brogan, and Ann Sarah Walker conceived the study, led the application for funding and led the project and protocol development with input from Rachael McCormack, Robin Kahn, Maria Mossberg, Jethro Herberg, Robert Tulloh, Filip Kucera. Oversight of the trial was provided from Yolanda Collaco Moraes, Roisin Connon, Ann Sarah Walker from the MRC CTU. Charlotta Nordenhaell, Marion Bailhache, Sauli Palmu, Carlotta Webb, Brian McCrossan, Jethro Herberg recruited patients to the trial and analysed data. Georgi Christov, Rob Tulloh analysed echocardiography data. John Stone assisted with pGTI data analysis. Roisin Connon and Ann Sarah Walker did the statistical analysis. Rachael Hunter did the health economics analysis. Despina Eleftheriou, Paul Brogan, Ann Sarah Walker prepared the initial manuscript. Despina Eleftheriou, Paul Brogan, Ann Sarah Walker, Roisin Connon assessed and verified the data. All authors had full access to all the data in the study and had final responsibility for the decision to submit for publication.

## Data sharing statement

The final trial protocol is available online (www.mrcctu.ucl.ac.uk/studies/all-studies/k/kd-caap/). Given the potential identifiability of participants given the rarity of KD, a controlled data access approach to data sharing will be used. Those wishing to access the data should contact the corresponding author and complete a data access request form. Subject to approval by the chief investigators, with a data sharing agreement, anonymised trial data and the data dictionary will be made available.

## Declaration of interests

Declaration of interests. Robin Kahn reports serving on an advisory board for AbbVie. Charlotta Nordenhall reports being Chair of the Swedish Paediatric Rheumatology Society. Rachael Hunter reports receiving consulting fees from Abbott, QuidelOrtho, and Lepzi; support from Abbott for meeting attendance; and research grants from HPRU-BBV, DenPRU-QM, the RSS, NIHR. She also served as Co-Chair of the European Union Transforming Health and Care Systems Funding Board in 2023. John Stone reports being Chair of the Scientific Advisory Board for Steritas. All other authors declare no competing interests.

## Appendix A Supplementary data

Manual of operations

Echocardiography

List of KDCAAP investigators/collaborators

Protocol

Statistical analysis plan
