# Supplementary material for: Multi-centre, randomised, open-label, blinded endpoint assessed, trial of corticosteroids plus intravenous immunoglobulin (IVIG) and aspirin, versus IVIG and aspirin for prevention of coronary artery aneurysms (CAA) in Kawasaki disease (KD): the KD-CAA prevention (KD-CAAP) trial
Source: eClinicalMedicine. 2026 Jul 13;97:104044. doi: 10.1016/j.eclinm.2026.104044 (PMC13382441; doi:10.1016/j.eclinm.2026.104044)
Supplement: Manual of operations [file mmc1.pdf]

KD-CAAP: Kawasaki Disease Coronary Artery Aneurysm Prevention trial

Manual of Operations

Chapter 09 – Echocardiography request and image upload

Version 1.0 dated 24-Sep-2021			
**Author**	**Position**	**Signature**	**Date**
Cara Purvis	Trial Manager	DocuSigned by: Cara Purvis D8C31ADAA117462...	24-Sep-2021
**Reviewer**	**Position**	**Signature**	**Date**
Rob Tulloh	Cardiology Co-Investigator	DocuSigned by: Rob Tulloh E5EF4AF4140243C...	25-Sep-2021
Filip Kucera	Cardiology Co-Investigator	DocuSigned by: Filip Kucera 483AC6891C1A441...	24-Sep-2021

Revision History

Version	Date	Author	Reason for revision
1.0	24-Sep-2021	Cara Purvis	Initial

Contents

9 KD-CAAP – Echocardiography request and image upload ... 3
9.1. Echocardiogram Assessment schedule ... 3
9.2. Echocardiogram Request ... 3
9.3. Echocardiogram Data Collection ... 4
Form 11 Completion ... 4
9.4. Central Review of echocardiogram ... 5
9.5. Submission of echocardiogram ... 5
Access ... 5
Upload of images ... 6

9 KD-CAAP – Echocardiography request and image upload

KD-CAAP has the following two co-primary outcome measures based on repeat echocardiography undertaken at participants week 1, 2, 6 and 12 follow up visits:
(i) Any CAA documented within the 12 weeks of trial follow-up (to assess overall effectiveness of the strategy of immediate corticosteroids in preventing CAA, expecting that some patients will receive rescue treatment before reaching this endpoint in both groups)
(ii) An average estimate across weeks 1, 2, and 6 of the maximum Z-score of the internal diameters of the proximal right coronary artery or left anterior descending coronary artery, adjusting for rescue treatment (to assess the efficacy of corticosteroids).
Additional secondary efficacy outcomes are also based on echocardiography assessments.

This chapter goes through the requirements when requesting a Echocardiogram for a KD-CAAP participant.

Prior to site opening, it should be ensured that all participants recruited into KD-CAAP will have the ability to perform the required echocardiograms following the schedule (section 9.1) and that echocardiograms will be conducted at a centre which performs paediatric echocardiograms regularly, also sites will need to confirm they have the capacity to obtain the images in DICOM format and uploaded to the image repository.

9.1. Echocardiogram Assessment schedule

To allow the assessment of the co-primary outcomes, randomised children/adolescents must have echocardiogram assessments completed at the following visits:

- **Follow-up visit Week 1 (visit window: 1 day before and 3 days after the planned date)**
- **Follow-up visit Week 2 (visit window: 3 days either side the planned date)**
- **Follow-up visit Week 6 (visit window: 2 weeks either side the planned date)**
- **Follow-up visit Week 12 (visit window: 2 weeks either side the planned date)**

Echocardiograms should be arranged as soon as the participant is randomised to ensure that they follow the visit schedule where possible.

Children/adolescents who do not meet the complete KD diagnosis may have an echocardiogram performed prior to randomisation (on the day of randomisation or the day before) to allow the assessment of their eligibility in relation to the incomplete KD diagnosis.

Any additional echocardiograms performed outside the required trial visits from Screening/Randomisation until the week 12 visit should also be reported following this manual.

9.2. Echocardiogram Request

To ensure that the echocardiogram images are preformed to the standard required for the trial and to ensure all trial related data can be collected the KD-CAAP Echocardiogram Specification document must be sent alongside **every** echocardiogram request.
The document can be found in appendix 1 of this chapter.

9.3. Echocardiogram Data Collection

For each echocardiogram performed for a child/adolescent during trial follow-up (following Section 2.1) a Form 11a – Echocardiogram Assessment – Research Site must be completed. This can be by the echocardiographer completing the assessment or another member of the site team who has been delegated the responsibility for Case Report Form completion. The information should be extracted from the imaging report.

Form 11 Completion

A paper copy of the echocardiogram form should be completed and stored with the child/adolescent's Case Report Forms. A copy of the completed Form 11a – Echocardiogram Assessment – Research Site must be sent by secure email to the KD-CAAP Coordinating Centre at the following email address: mrcctu.kdcaap@ucl.ac.uk within 2 weeks of the KD-CAAP visit or date the echocardiogram was performed. Further details on the completion of CRFs can be found in the KD-CAAP Manual of Operations Chapter 3.

The following data and parameters are collected on Form 11a – Echocardiogram Assessment – Research Site:

- Date of the echocardiogram
- Participant's height and weight for the visit that the echocardiogram is being completed for, this should match the data collected on Form 04 for that visit.
- Assessment of cardiac function – normal/global dysfunction/regional dysfunction;
- Ejection fraction (biplane Simpson method);
- Left Ventricular End Diastolic Diameter (LVEDD) from M-mode;
- Left Ventricular End Systolic Diameter (LVESD) from M-mode;
- Assessment of mitral valve regurgitation – absent/mild/moderate/severe;
- Transmitral inflow characteristics including the peak early filling (E wave) and late diastolic filling (A wave) velocities and the E/A ratio;
- Pulsed wave tissue Doppler Imaging (TDI) sampling from the septal and lateral mitral annulus including the early diastolic relaxation velocity (e′) and the systolic myocardial velocity (s');
- Measurement of peak tricuspid regurgitation velocity;
- Measurement of diastolic left ventricular eccentricity index;
- Presence of pericardial effusion and depth in parasternal long axis plane;
- Coronary artery dimension measurements (including the measurement, Z-score, appearance; normal, saccular aneurysm, fusiform aneurysm, ectasia, presence of thrombus and presence of pericardial effusion.
    - internal diameter of the left main coronary artery (LMCA)
    - internal diameter of the left anterior descending (LAD)
    - internal diameter of the proximal right coronary artery (RCA)
    - Any additional internal diameters
- If the child/adolescent has any segments which have an internal diameter at least 1.5 times that of an adjacent segment
- If the child/adolescent's luminal contour is irregular
- If the child/adolescent has severe congestive heart failure or cardiogenic shock, defined as the presence of hypertension and shock requiring the initiation of volume expander or inotropic support
- If the child/adolescent has a structurally normal heart
- The name of the echocardiographer that completed the scan

9.4. Central Review of echocardiogram

Echocardiographic studies will be reviewed centrally in the core echocardiographic laboratory (Great Ormond Street Hospital). The validity of parameters in Form 11a will be confirmed with the loops and images sent, by at least one of two independent echocardiographers are blinded to randomised allocation. Repeat echocardiographic measurements may be required if necessary.

9.5. Submission of echocardiogram

To allow the central review of the echocardiograms for the KD-CAAP study, all echocardiograms performed on the child/adolescent during their trial participation must be submitted to the MRC CTU.

The Image Repository is a system which has been developed by the MRC CTU to facilitate the collection of Echocardiogram images from KD-CAAP participants. It is web-based and should ideally be accessed through Chrome.

The image files must be submitted in **DICOM format** and should be uploaded onto the KD-CAAP Image Repository.

Access

Access to the Image Repository is controlled and only those authorised by the KD-CAAP Coordinating Centre at the MRC CTU at UCL will have access to upload the echocardiogram DICOMs on to the Image Repository.

To gain access to the Image Repository, the site staff member must be authorised for this role by the PI on the KD-CAAP Signature and Delegation of Responsibility Log and must have completed the following training (contact the KD-CAAP Coordinating Centre for the training):

- Completed Protocol Training as part of the Site Initiation Training or read and understood the Protocol Training slides or completed the training on the c4c platform – record of this must be on the KD-CAAP training log
- Completed GCP training

Once all the above has been completed a password non-disclosure form will need to be completed prior to access being granted.

The KD-CAAP Coordinating Centre will create an account for you. You should receive an automatic email with your username and password.

The Image Repository can be accessed using the following link: www.cturepo.com

Enter the email address for which the Image Repository was requested for and the password provided in the email you received.

DocuSign Envelope ID: 293316E5-173D-4C36-9E1B-27DF0AFFD072

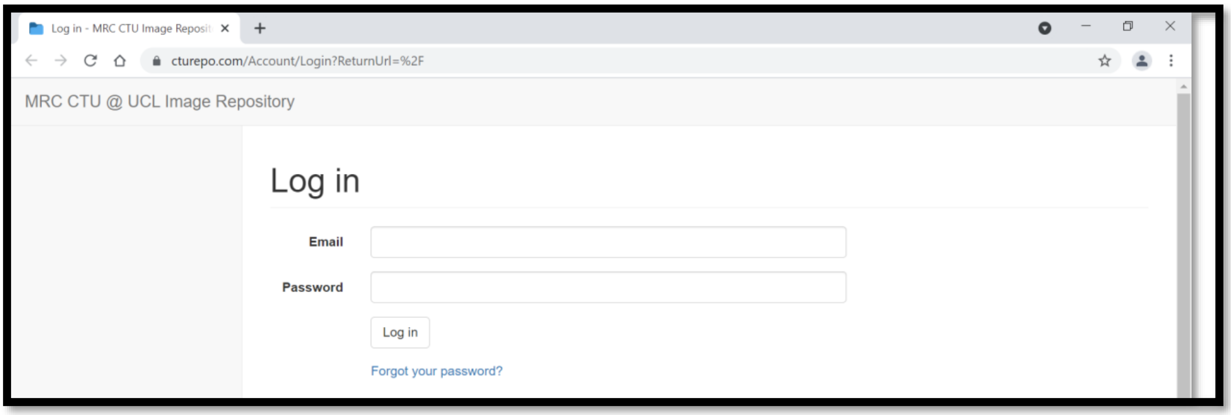

The first time that you access the Image Repository, you should change your password. To change your password log in and go to the 'Profile' icon in the right hand corner

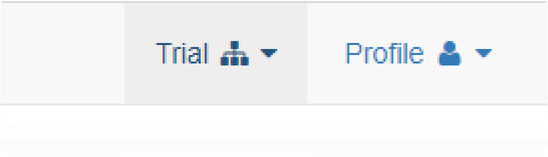

From this drop down, select 'User Profile' and then the following option should appear:

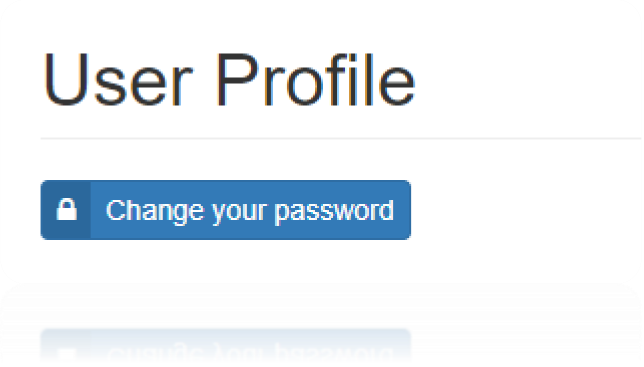

Click on the change your password link and update your details.

If you have forgotten your password, click on the forgotten password link. The link will direct you to a webpage where you can enter your email address. Enter your email address and the instructions on how to reset your password will be sent to your email from CTU IR <noreply@cturepo.com>. If the reset email cannot be found in your inbox, check your junk/spam folder in your mailbox.

Upload of images

Once logged into the image repository, follow the steps below to upload a scan:

1. From the KD-CAAP Dashboard click on the 'Upload' icon, or select it on the tool bar on the left.

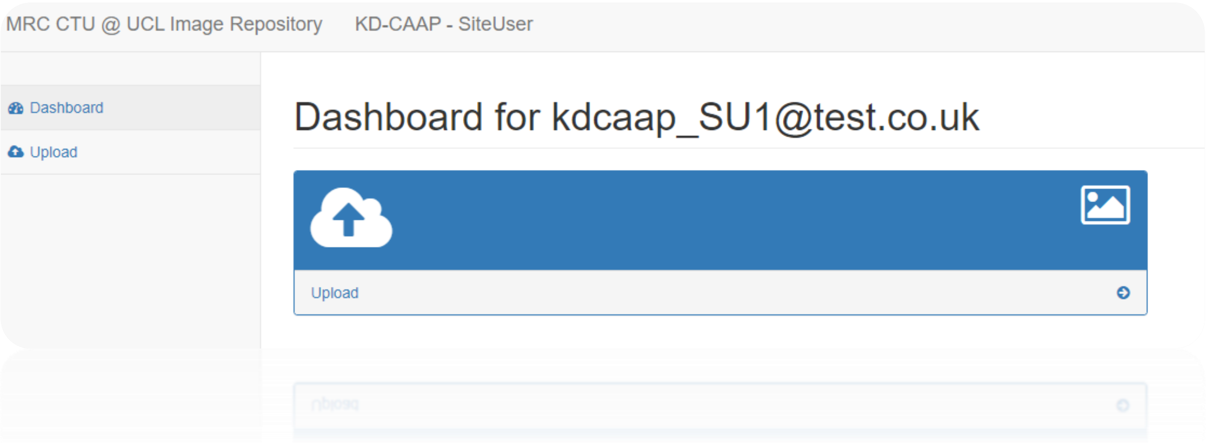

2. This will take you to the following webpage where the patient details should be entered:

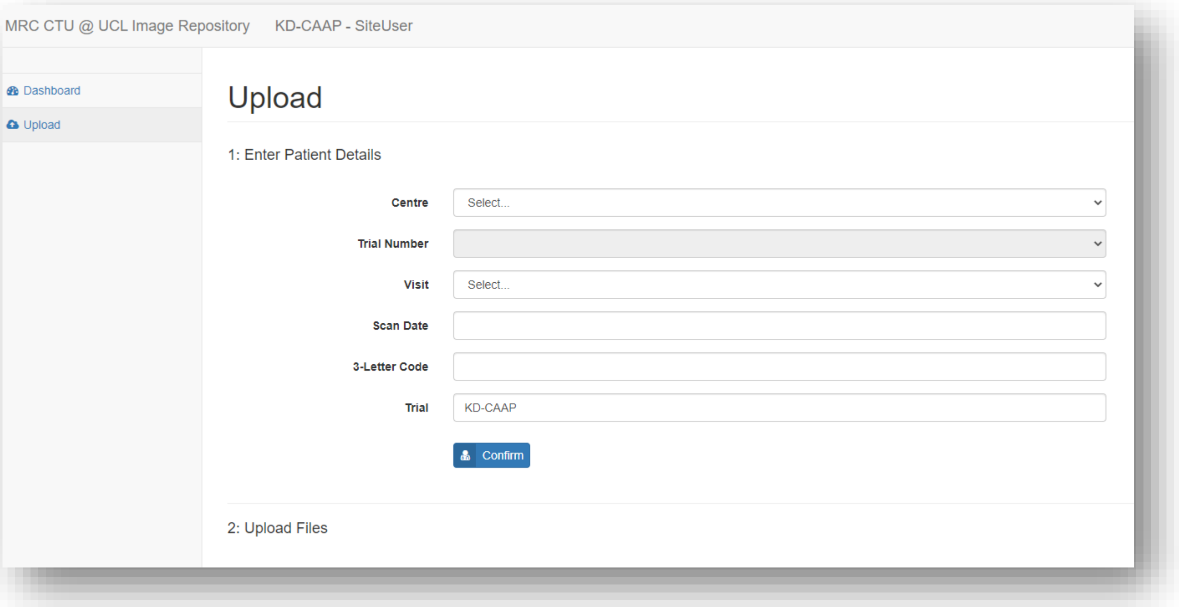

- Your site name will automatically prefill into the Centre.
- Use the drop down list to select the trial number for which you are uploading the DICOM images.
- Use the drop down list to select the appropriate visit for which the scan has been completed. The list includes all KD-CAAP visits, not only the KD-CAAP visits at which an echocardiogram is required.

- Enter the date the scan was completed in the Scan Date – this should correspond with the date on the Form 04 Follow up and Form 11a Echocardiogram Assessment – Research Site.
- Enter the participant's 3-letter code – this is provided to the participant alongside the trial ID following consent/assent.
- Click confirm. The data entered will undergo some checks to ensure that the 3-letter code is correct for the Trial Number. If it is not the following error message will appear:

Error! The entered patient Trial Number and 3-Letter Code do not match

If there are no issues identified during this check step the following message will appear:

Success! The patient scan record has been successfully created

3. Once the patient details have been entered correctly the link to upload file will open and DICOM images can be uploaded. Prior to the upload of the scan, ensure that all personal identifiers have been removed from the image file and the scan can be identified by the Trial ID and the 3-Letter code. Once pseudonymised, click the green upload file icon and locate the folder containing the image files you would like to upload.

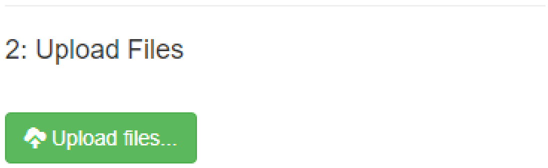

N.B: If you are using Internet Explorer, you will either need to zip the files prior to upload or use control and select all of the image files.

4. Once the folder with your images has been located, click upload. The images will then be uploaded.

5. Whilst the files are uploading the following image appears:

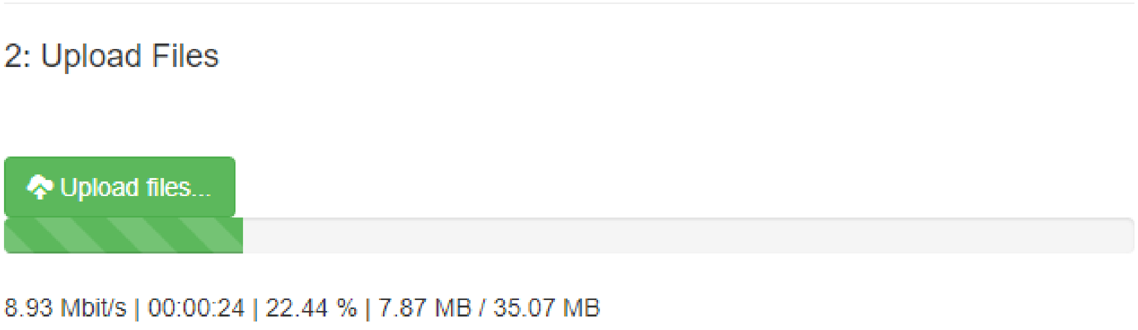

Do not close the browser whilst the images are uploading or else the images will not be successfully uploaded and the upload will need to be restarted. It is possible to perform other tasks while the scan is uploading, however the speed may vary depending on the bandwidth.
Once the images have been uploaded the following message should appear:

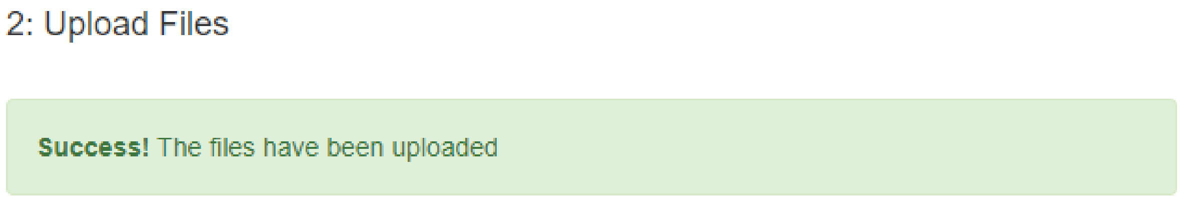

The browser can then be closed.

If you want to ensure the scans have been uploaded successfully, please contact the KD-CAAP Coordinating centre and they will confirm the image upload status.

If there are any issues with the quality of the image or the upload the KD-CAAP Coordinating Centre will contact the site.

9.6. Appendix

Appendix 1 – Echocardiogram specifications

DocuSign

Certificate Of Completion

Envelope Id: 293316E5173D4C369E1B27DF0AFFD072
Subject: Please DocuSign: KD-CAAP MOP_Chapter 9_v1.0.docx
Source Envelope:
Document Pages: 9
Certificate Pages: 5
AutoNav: Enabled
EnvelopeId Stamping: Enabled
Time Zone: (UTC) Dublin, Edinburgh, Lisbon, London

Signatures: 3
Initials: 0

Status: Completed

Envelope Originator:
Cara Purvis
90 High Holborn 2nd Floor London
London, London WC1V 6LJ
c.purvis@ucl.ac.uk
IP Address: 92.18.19.40

Record Tracking

Status	Holder	Location
Status: Original<br>24 September 2021 | 08:44	Holder: Cara Purvis<br>c.purvis@ucl.ac.uk	Location: DocuSign

Signer Events

Signer Events	Signature	Timestamp
Cara Purvis<br>c.purvis@ucl.ac.uk<br>Trial Manager<br>MRC CTU at UCL<br>Security Level: Email, Account Authentication (Optional)	DocuSigned by:<br>Cara Purvis<br>D8C31ADAA117462...<br><br>Signature Adoption: Pre-selected Style<br>Using IP Address: 92.18.19.40	Sent: 24 September 2021 | 08:46<br>Viewed: 24 September 2021 | 08:46<br>Signed: 24 September 2021 | 08:46

Electronic Record and Signature Disclosure:
Not Offered via DocuSign

Signer Events	Signature	Timestamp
Filip Kucera<br>Filip.Kucera@gosh.nhs.uk<br>Security Level: Email, Account Authentication (Optional)	DocuSigned by:<br>Filip Kucera<br>483AC6891C1A441...<br><br>Signature Adoption: Pre-selected Style<br>Using IP Address: 109.232.56.93	Sent: 24 September 2021 | 08:46<br>Viewed: 24 September 2021 | 09:27<br>Signed: 24 September 2021 | 09:28

Electronic Record and Signature Disclosure:
Accepted: 24 September 2021 | 09:27
ID: e203d15d-244e-47f1-a759-4fb9e7ae3c1d

Signer Events	Signature	Timestamp
Rob Tulloh<br>roberttulloh1@gmail.com<br>Security Level: Email, Account Authentication (Optional)	DocuSigned by:<br>Rob Tulloh<br>E5EF4AF4149242C...<br><br>Signature Adoption: Pre-selected Style<br>Using IP Address: 213.205.194.229	Sent: 24 September 2021 | 08:46<br>Resent: 24 September 2021 | 15:43<br>Viewed: 25 September 2021 | 17:36<br>Signed: 25 September 2021 | 17:36

Electronic Record and Signature Disclosure:
Accepted: 25 September 2021 | 17:36
ID: b6c58565-d2a8-4714-a175-3e543cd60281

In Person Signer Events	Signature	Timestamp

Editor Delivery Events	Status	Timestamp

Agent Delivery Events	Status	Timestamp

Intermediary Delivery Events	Status	Timestamp

Certified Delivery Events	Status	Timestamp

Carbon Copy Events	Status	Timestamp

Witness Events	Signature	Timestamp

Notary Events	Signature	Timestamp

Envelope Summary Events	Status	Timestamps
Envelope Sent	Hashed/Encrypted	24 September 2021 | 08:46
Certified Delivered	Security Checked	25 September 2021 | 17:36
Signing Complete	Security Checked	25 September 2021 | 17:36
Completed	Security Checked	25 September 2021 | 17:36

Payment Events	Status	Timestamps

Electronic Record and Signature Disclosure

ELECTRONIC RECORD AND SIGNATURE DISCLOSURE

From time to time, MRC Clinical Trials Unit at UCL (we, us or Company) may be required by law to provide to you certain written notices or disclosures. Described below are the terms and conditions for providing to you such notices and disclosures electronically through the DocuSign system. Please read the information below carefully and thoroughly, and if you can access this information electronically to your satisfaction and agree to this Electronic Record and Signature Disclosure (ERSD), please confirm your agreement by selecting the check-box next to 'I agree to use electronic records and signatures' before clicking 'CONTINUE' within the DocuSign system.

Getting paper copies

At any time, you may request from us a paper copy of any record provided or made available electronically to you by us. You will have the ability to download and print documents we send to you through the DocuSign system during and immediately after the signing session and, if you elect to create a DocuSign account, you may access the documents for a limited period of time (usually 30 days) after such documents are first sent to you. After such time, if you wish for us to send you paper copies of any such documents from our office to you, you will be charged a $0.00 per-page fee. You may request delivery of such paper copies from us by following the procedure described below.

Withdrawing your consent

If you decide to receive notices and disclosures from us electronically, you may at any time change your mind and tell us that thereafter you want to receive required notices and disclosures only in paper format. How you must inform us of your decision to receive future notices and disclosure in paper format and withdraw your consent to receive notices and disclosures electronically is described below.

Consequences of changing your mind

If you elect to receive required notices and disclosures only in paper format, it will slow the speed at which we can complete certain steps in transactions with you and delivering services to you because we will need first to send the required notices or disclosures to you in paper format, and then wait until we receive back from you your acknowledgment of your receipt of such paper notices or disclosures. Further, you will no longer be able to use the DocuSign system to receive required notices and consents electronically from us or to sign electronically documents from us.

All notices and disclosures will be sent to you electronically

Unless you tell us otherwise in accordance with the procedures described herein, we will provide electronically to you through the DocuSign system all required notices, disclosures, authorizations, acknowledgements, and other documents that are required to be provided or made available to you during the course of our relationship with you. To reduce the chance of you inadvertently not receiving any notice or disclosure, we prefer to provide all of the required notices and disclosures to you by the same method and to the same address that you have given us. Thus, you can receive all the disclosures and notices electronically or in paper format through the paper mail delivery system. If you do not agree with this process, please let us know as described below. Please also see the paragraph immediately above that describes the consequences of your electing not to receive delivery of the notices and disclosures electronically from us.

How to contact MRC Clinical Trials Unit at UCL:

You may contact us to let us know of your changes as to how we may contact you electronically, to request paper copies of certain information from us, and to withdraw your prior consent to receive notices and disclosures electronically as follows:
To contact us by email send messages to: s.assam@ucl.ac.uk

To advise MRC Clinical Trials Unit at UCL of your new email address

To let us know of a change in your email address where we should send notices and disclosures electronically to you, you must send an email message to us at s.assam@ucl.ac.uk and in the body of such request you must state: your previous email address, your new email address. We do not require any other information from you to change your email address.

If you created a DocuSign account, you may update it with your new email address through your account preferences.

To request paper copies from MRC Clinical Trials Unit at UCL

To request delivery from us of paper copies of the notices and disclosures previously provided by us to you electronically, you must send us an email to s.assam@ucl.ac.uk and in the body of such request you must state your email address, full name, mailing address, and telephone number. We will bill you for any fees at that time, if any.

To withdraw your consent with MRC Clinical Trials Unit at UCL

To inform us that you no longer wish to receive future notices and disclosures in electronic format you may:

i. decline to sign a document from within your signing session, and on the subsequent page, select the check-box indicating you wish to withdraw your consent, or you may;

ii. send us an email to s.assam@ucl.ac.uk and in the body of such request you must state your email, full name, mailing address, and telephone number. We do not need any other information from you to withdraw consent.. The consequences of your withdrawing consent for online documents will be that transactions may take a longer time to process..

Required hardware and software

The minimum system requirements for using the DocuSign system may change over time. The current system requirements are found here: https://support.docusign.com/guides/signer-guide-signing-system-requirements.

Acknowledging your access and consent to receive and sign documents electronically

To confirm to us that you can access this information electronically, which will be similar to other electronic notices and disclosures that we will provide to you, please confirm that you have read this ERSD, and (i) that you are able to print on paper or electronically save this ERSD for your future reference and access; or (ii) that you are able to email this ERSD to an email address where you will be able to print on paper or save it for your future reference and access. Further, if you consent to receiving notices and disclosures exclusively in electronic format as described herein, then select the check-box next to ‘I agree to use electronic records and signatures’ before clicking ‘CONTINUE’ within the DocuSign system.

By selecting the check-box next to ‘I agree to use electronic records and signatures’, you confirm that:

- You can access and read this Electronic Record and Signature Disclosure; and
- You can print on paper this Electronic Record and Signature Disclosure, or save or send this Electronic Record and Disclosure to a location where you can print it, for future reference and access; and
- Until or unless you notify MRC Clinical Trials Unit at UCL as described above, you consent to receive exclusively through electronic means all notices, disclosures, authorizations, acknowledgements, and other documents that are required to be provided or made available to you by MRC Clinical Trials Unit at UCL during the course of your relationship with MRC Clinical Trials Unit at UCL.
