# Supplementary material for: Multi-centre, randomised, open-label, blinded endpoint assessed, trial of corticosteroids plus intravenous immunoglobulin (IVIG) and aspirin, versus IVIG and aspirin for prevention of coronary artery aneurysms (CAA) in Kawasaki disease (KD): the KD-CAA prevention (KD-CAAP) trial
Source: eClinicalMedicine. 2026 Jul 13;97:104044. doi: 10.1016/j.eclinm.2026.104044 (PMC13382441; doi:10.1016/j.eclinm.2026.104044)
Supplement: Echocardiography [file mmc2.pdf]

KD-CAAP

MRC Clinical Trials Unit

Smarter Studies Global Impact Better Health

UCL

# KD-CAAP: Kawasaki Disease Coronary Artery Aneurysm Prevention trial

# Echocardiogram Specifications

## Performing an Echocardiogram

The echocardiogram is performed on a dedicated cardiology ultrasound using the highest resolution probe available.

**Coronary artery assessment on echo:**

Optimisation of the 2D tissue definition is achieved with a higher resolution probe, tissue harmonic imaging and gain optimisation. The colour Doppler (CD) visualisation of coronary flow is achieved by decreasing the Nyquist limit and optimising the gain or applying coronary default setting. The maximal internal diameter between the endocardial borders is measured at 2-5 mm from the origin of the coronary artery, avoiding sites of branching. Coronary artery measurements should be made using low-scale colour mapping with dual display to avoid erroneous measurement of cardiac veins or artefacts that resemble the coronary arteries.

The left main, proximal and distal left anterior descending, circumflex, and proximal right coronary artery diameters are best measured in parasternal short-axis views; the distal right coronary artery diameter is best measured in apical 4-chamber view with posterior angulation; and the posterior descending coronary artery diameter is best measured in parasternal long-axis views with rightward posterior angulatio n.

Z-score for BSA is presented as calculated by the Lopez et al. (Circ Cardiovasc Imaging 2017) reference values (www.parameterz.com/refs/lopez-circimaging-2017). Coronary artery appearance, presence of thrombi or coronary stenosis is reported when suspected. Three-cycle digital images have to be stored for later off-line detailed Echo analysis. Extreme care must be taken to measure the height/length accurately, since this has a profound effect on the BSA measurement.

**Conventional echocardiographic parameters and myocardial deformation imaging:**

The Echo protocol includes the complete standard 2D echocardiography with the use of colour Doppler (CD), pulsed wave (PW) and continuous wave (CW) Doppler as well as pulsed-wave tissue Doppler imaging (TDI) techniques. Left ventricular size measurements are acquired using M-mode from parasternal long axis view. The 2D ejection fraction by Simpson's method (EF) requires the measurement of left ventricular EF by tracing endocardial border in both apical four-chamber and two-chamber views in end-systole and end-diastole. The PW mitral inflow early diastolic velocity (E), late diastolic velocity (A), E/A ratio, are measured. The TDI annular velocities in systole (s'), early diastole (e') and late diastole (a') are interrogated at the mitral lateral (M lat) and mitral medial (M med) positions. The presence of pericardial effusion will be assessed in particular in apical four chamber view and parasternal long axis view.

## The following data and parameters must be collected for the trial

- Participant's height and weight for the visit that the echocardiogram is being completed for.
- Assessment of cardiac function – normal/global dysfunction/regional dysfunction;
- Ejection fraction (biplane Simpson method);
- Left Ventricular End Diastolic Diameter (LVEDD) and Left Ventricular End Systolic
- Diameter (LVESD) from M-mode;
- Assessment of mitral valve regurgitation – absent/mild/moderate/severe;
- Transmitral inflow characteristics including the peak early filling (E wave) and late diastolic filling (A wave) velocities and the E/A ratio;
- Pulsed wave tissue Doppler Imaging (TDI) sampling from the septal and lateral mitral annulus including the early diastolic relaxation velocity (e') and the systolic myocardial velocity (s');
- Measurement of peak tricuspid regurgitation velocity;
- Measurement of diastolic left ventricular eccentricity index;
- Presence of pericardial effusion and depth in parasternal long axis plane;
- Coronary artery dimension measurements (including the measurement, Z-score, appearance; normal, saccular aneurysm, fusiform aneurysm, ectasia, presence of thrombus and presence of pericardial effusion.
    - internal diameter of the left main coronary artery (LMCA)
    - internal diameter of the left anterior descending (LAD)
    - internal diameter of the proximal right coronary artery (RCA)
    - Any additional internal diameters
- If the child/adolescent has any segments which have an internal diameter at least 1.5 times that of an adjacent segment
- If the child/adolescent's luminal contour is irregular
- If the child/adolescent has severe congestive heart failure or cardiogenic shock, defined as the presence of hypertension and shock requiring the initiation of volume expander or inotropic support
- If the child/adolescent has a structurally normal heart
