# Supplementary material for: Multi-centre, randomised, open-label, blinded endpoint assessed, trial of corticosteroids plus intravenous immunoglobulin (IVIG) and aspirin, versus IVIG and aspirin for prevention of coronary artery aneurysms (CAA) in Kawasaki disease (KD): the KD-CAA prevention (KD-CAAP) trial
Source: eClinicalMedicine. 2026 Jul 13;97:104044. doi: 10.1016/j.eclinm.2026.104044 (PMC13382441; doi:10.1016/j.eclinm.2026.104044)
Supplement: List of KDCAAP investigators/collaborators [file mmc3.docx]

**List of KD-CAAP trial investigators/collaborators**

| **First name** | **Surname** | **Affiliation** |
| --- | --- | --- |
| Marta | Merida Morillas | MRC Clinical trials Unit, UCL, London UK |
| Molly | Pursell | MRC Clinical trials Unit, UCL, London UK |
| Charalampia | Papadopoulou | Great Ormond Street Hospital for Children NHS Foundation Trust |
| Muthana | AlObaidi | Great Ormond Street Hospital for Children NHS Foundation Trust |
| Elena | Moraitis | Great Ormond Street Hospital for Children NHS Foundation Trust |
| Jo | Walsh | Royal Hospital for Children, Glasgow, UK |
| Kirsty | McLellan | Royal Hospital for Children, Glasgow, UK |
| Maria | Ilina | Royal Hospital for Children, Glasgow, UK |
| Marc | Jansen | University Medical Centre Utrecht, Pediatric Immunology and Rheumatology, Netherlands |
| Mildred | Iro | Paediatric infectious diseases and immunology, Royal London Hospital, Barts Health NHS Trust, London, UK |
| Wendy | Dewals | Universitair Ziekenhuis Antwerpen, Belgium |
| Mari | Laan | Tallinn Children’s Hospital, Estonia |
| Lonneke | Van Onzenoort | MMC Veldhoven, Paediatrics, Netherlands |
| Daniel | Langer | Paediatric Emergency Dept, Epsom & St Helier University Hospitals NHS Trust, UK |
| Will | Coles | Birmingham Children’s Hospital, Birmingham UK |
| Stephan | Paulus | Department of Paediatrics, University of Oxford, UK |
| Eleri | Williams | Paediatric Infectious Disease and Immunology, Great North Children’s Hospital, Newcastle-upon-Tyne, UK |
| Esmeralda | Núñez-Cuadros | Pediatric Rheumatology. Hospital Regional Universitario de Málaga. Instituto de Investigación Biomédica de Málga (IBIMA) Spain |
| Jordi | Antón | Hospital Sant Joan de Déu / Pediatric Rheumatology Department, Spain |
| Olof | Hertting | Department of Women's and Children's Health, Karolinska Institutet, Sweden |
| Lien | De Somer | Division of Pediatric Rheumatology, Department of Pediatrics, University Hospitals Leuven, Leuven, Belgium. |
| Stefan | Berg | Sahlgrenska University Hospital, Göteborg, Sweden |
| Joseph | Panzer | Universitair Ziekenhuis Gent, |
| Ilse | Meerschaut | Department of Paediatric Cardiology, Universitair Ziekenhuis Brussel, Vrije Universiteit Brussel (VUB), Brussels, Belgium. |
| Piia | Jõgi | Children´s Clinic of Tartu University Hospital, Department of Acute Infections, Estonia |
| Frédéric | Huet | Child Hospital – CHU DIJON, France |
| Eric | Jeziorski | CHU Montpellier, France |
| Ulrich | von Both | Ludwig-Maximilians-University of Munich, Germany |
| Andre | Jakob | Department of Pediatric Cardiology and Pediatric Intensive Care, Ludwig-Maximilians-University of Munich, Germany |
| Tobias | Krickau | Universitätsklinikum Erlangen, Germany |
| Silvia | Rosina | IRCCS Istituto Giannina Gaslini, Italy |
| Gabrielle | Simonini | Rheumatology Unit, Meyer Children Hospital IRCCS, Firenze. |
| Andrea | Taddio | Institute for Maternal and Child Health - IRCCS Burlo Garofolo and University of Trieste, Italy |
| Elisa | Fernandez Cooke | Hospital Universitario 12 de Octubre. Spain. Imas12. CIBERINFEC |
| Owen | Miller | Paediatric cardiology, Evelina Children’s Hospital, London, UK |
| Elinor | Sefi | Department of Paediatrics, The Whittington Hospital, London, UK |
| Michael | Levin | Department of Infectious Disease - Faculty of Medicine, St Mary’s Hospital, UK |
| Veronica | Swallow | Sheffield Hallam University, Sheffield, UK |
| Mandy | Wan | Evelina Children’s Hospital, London, UK |
| Joseph | Standing | School of Pharmacy, UCL, London, UK |
| Kimberley | Pang | MRC Clinical Trials Unit, UCL, London UK |
| James | Wyncoll | MRC Clinical Trials Unit, UCL, London UK |
