# Supplementary material for: Multi-centre, randomised, open-label, blinded endpoint assessed, trial of corticosteroids plus intravenous immunoglobulin (IVIG) and aspirin, versus IVIG and aspirin for prevention of coronary artery aneurysms (CAA) in Kawasaki disease (KD): the KD-CAA prevention (KD-CAAP) trial
Source: eClinicalMedicine. 2026 Jul 13;97:104044. doi: 10.1016/j.eclinm.2026.104044 (PMC13382441; doi:10.1016/j.eclinm.2026.104044)
Supplement: Protocol [file mmc4.pdf]

DocuSign Envelope ID: 651725BB-F200-474E-A72C-A62C1F4636C1

KD-CAAP: Kawasaki Disease Coronary Artery Aneurysm Prevention trial

Multi-centre, randomised, open-label, blinded endpoint assessed, trial of corticosteroids plus intravenous immunoglobulin (IVIG) and aspirin, versus IVIG and aspirin for prevention of coronary artery aneurysms in Kawasaki disease

Version: 5.0
Date: 22 October 2021

MRC CTU at UCL ID: KD-CAAP
ISRCTN #: ISRCTN71987471
EUDRACT#: 2019-004433-17

Authorised by:

Name: Professor Despina Eleftheriou
Role: Co-Chief Investigator
Signature: DocuSigned by: BC46861BC1E1424...
Date: 27-Oct-2021

Name: Professor Paul Brogan
Role: Co-Chief Investigator
Signature: DocuSigned by: 7F855630096D46B...
Date 27-Oct-2021

Name: Professor Ann Sarah Walker
Role: Trial Statistician
Signature: DocuSigned by: Sarah Walker E13D67A837DD418...
Date: 27-Oct-2021

DocuSign Envelope ID: 651725BB-F200-474E-A72C-A62C1F4636C1

This protocol has been produced using MRC CTU at UCL Protocol Template version 9.0. The template, but not any study-specific content, is licensed under a Creative Commons Attribution 4.0 International License (https://creativecommons.org/licenses/by/4.0/). Use of the template in production of other protocols is allowed, but MRC CTU at UCL must be credited.

DocuSign Envelope ID: 651725BB-F200-474E-A72C-A62C1F4636C1

GENERAL INFORMATION

This document was constructed using the Medical Research Council (MRC) Clinical Trials Unit (CTU) at University College London (UCL) Protocol Template Version 9.0. The MRC CTU at UCL endorses the Standard Protocol Items: Recommendations for Interventional Trials (SPIRIT) initiative. It describes the KD-CAAP trial, coordinated by the MRC CTU at UCL, and provides information about procedures for entering children/adolescents into it. The protocol should not be used as an aide-memoire or guide for the treatment of other children/adolescents. Every care has been taken in drafting this protocol, but corrections or amendments may be necessary. These will be circulated to the registered investigators in the trial, but sites entering children/adolescents for the first time are advised to contact the MRC CTU at UCL, London, UK, to confirm they have the most up-to-date version.

COMPLIANCE

This trial will be conducted in compliance with the approved protocol by the Sponsor, the Investigator, and delegated Investigator staff and Sub-investigator, in accordance with consensus ethics principles derived from international ethics guidelines, including the Declaration of Helsinki (Fortaleza, Brazil, October 2013), and the ICH guidelines for good clinical practice (GCP), General Data Protection Regulation (GDPR) and all applicable laws, rules and regulations.

SPONSOR

University College London is the trial Sponsor and has delegated responsibility for the overall management of the KD-CAAP trial to the MRC CTU at UCL. Queries relating to UCL sponsorship of this trial should be addressed to the Max Parmar, MRC CTU at UCL Director, MRC CTU at UCL, Institute of Clinical Trials & Methodology, 90 High Holborn 2nd Floor, London, WC1V 6LJ.

FUNDING

This trial is funded by the Innovative Medicines Initiative 2 Joint Undertaking (JU), under grant agreement No 777389 that supports the Conect4children (c4c) research consortium. MRC CTU at UCL is supported by the MRC (UK).

AUTHORISATIONS AND APPROVALS

This trial will be submitted for approval by Research Ethics Committees/Institutional Review Boards in each of the participating countries across Europe and the UK; and by all required regulatory authorities in each of the participating countries.

TRIAL REGISTRATION

This trial is registered with the International Standard Randomised Clinical Trials Register (ISRCTN71987471). The trial is registered on EudraCT. The EudraCT number for the trial is 2019-004433-17.

SERIOUS ADVERSE EVENTS (SAE) REPORTING
Within 24 hours of becoming aware of an SAE, please send a completed SAE form to the MRC CTU at UCL on email: mrcctu.kdcaap@ucl.ac.uk

TRIAL SPONSOR

University College London

Gower Street
London
WC1E 6BT
United Kingdom

Tel: +44 207 679 6502
Fax: +44 203 108 2312

TRIAL ADMINISTRATION

Please direct all queries to the KD-CAAP Trial Manager at MRC CTU at UCL in the first instance; clinical queries will be passed to the Medical Experts via the CTU Trial Manager. For out of hours emergency queries contact either Paul Brogan on +447816826714 or Despina Eleftheriou +447729494552.

NB: throughout this document, "MRC CTU at UCL" is generally abbreviated to "CTU".

COORDINATING SITE

MRC Clinical Trials Unit at UCL
Infections Theme
2nd Floor, 90 High Holborn,
London
WC1V 6LJ
United Kingdom

Tel: +44 20 7670 4700
Fax: +44 20 7670 4814
Email: mrcctu.kdcaap@ucl.ac.uk

CTU STAFF AND AFFILIATES

Trial Statistician, Project Lead:	Prof Ann Sarah Walker, PhD FMedSci	Tel:<br>Email:	+44 207 670 4726<br>rmjlasw@ucl.ac.uk
Clinical Project Manager, co-investigator:	Yolanda Collaco Moraes, PhD	Tel:<br>Email:	+44 20 7670 4788<br>y.collaco-moraes@ucl.ac.uk
Statistician:	Roisin Connon	Tel:<br>Email:	+44 20 7670 4915<br>r.connon@ucl.ac.uk
Trial Manager:	Cara Purvis	Tel:<br>Email:	+44 20 7670 4930<br>c.purvis@ucl.ac.uk
Trial Manager:	Helen Webb	Tel:<br>Email:	+44 20 7670 4742<br>helen.webb@ucl.ac.uk

Data Manager: Molly Bush Tel: +44 20 7670 4641
Email: molly.bush@ucl.ac.uk

UCL Great Ormond Street Hospital Institute of Child Health

30 Guilford Street
London WC1N 1EH
United Kingdom

Co-Chief Investigator:	Professor Despina Eleftheriou, PhD	Tel:<br>Email:	+44 20 7905 2182<br>d.eleftheriou@ucl.ac.uk
Co-Chief Investigator:	Professor Paul Brogan, PhD	Tel:<br>Email:	+44 20 7905 2750<br>p.brogan@ucl.ac.uk
Co-investigator:	Professor Nigel Klein, PhD	Tel:<br>Email:	+44 20 7905 2215<br>n.klein@ucl.ac.uk
Co-investigator:	Dr Filip Kucera, MD	Email:	Filip.Kucera@gosh.nhs.uk

Co-investigators

UK-based Co-investigators

Section of Paediatric Infectious Diseases
Imperial College London,
Norfolk Place,
London, W2 1PG,
United Kingdom

Co- Investigator:	Prof Michael Levin, PhD	Tel:<br>Email:	+44 (0)20 7594 3760<br>m.levin@imperial.ac.uk
Co-investigator:	Dr Jethro Herberg, PhD	Email:	j.herberg@imperial.ac.uk

Sweden-based Co-investigators

Barn och Ungdomsmedicinska kliniken
Skåne University hospital,
Department of Paediatrics
Lund University
221 00 Lund, Sweden

Co-investigator:	Dr Robin Kahn, PhD	Email:	Robin.kahn@med.lu.se
Co-investigator:	Dr Maria Mossberg, PhD	Email:	Maria.Mossberg@med.lu.se

OTHER RESPONSIBLE INDIVIDUALS

CARDIOLOGY

Bristol Heart Institute
Marlborough Street
Bristol BS2 8HW
UK

Co-investigator:	Prof Robert Tulloh, DM, FRCPCH	Tel:	+44 7834 696677
		Email:	roberttulloh1@gmail.com

PHARMACOLOGY

UCL Great Ormond Street Institute of Child Health
30 Guilford Street
London, WC1N 1EH
United Kingdom

Co-investigator:	Dr Joe Standing, PhD	Tel:	+44 20 7905 2370
		Email:	j.standing@ucl.ac.uk

NURSING

Sheffield Hallam University,
Howard St,
Sheffield City Centre
Sheffield, S1 1WB
United Kingdom

Co-investigator:	Professor Veronica Swallow, PhD	Tel:	+44 78 6697 5124
		Email:	v.swallow@shu.ac.uk

HEALTH ECONOMICS

Department of Applied Health Research, UCL
1-19 Torrington Place
London WC1E 7HB
United Kingdom

Co-investigator:	Professor Paula Lorgelly, PhD	Tel:	+44 20 7679 5613
		Email:	p.lorgelly@ucl.ac.uk
Trial health economist:	TBA	Tel:	
		Email:	

DocuSign Envelope ID: 651725BB-F200-474E-A72C-A62C1F4636C1

COLLABORATING NETWORKS

Pediatric Rheumatology International Trials Organisation (PRINTO)
IRCCS G.Gaslini, Pediatria II, Reumatologia, PRINTO
Largo Gaslini, 5, Genova, Italy. 16147

Pediatric European Network for the Treatment of AIDS and Infectious Disease: PENTA-ID
Torre di Ricerca Pediatrica
Corso Stati Uniti 4
35127 Padova, Italy.

COLLABORATING PATIENT ORGANISATIONS

Societi Foundation, the UK Foundation for Kawasaki Disease
Victoria Court, Holme Lane
Winthorpe, Newark
Nottinghamshire NG24 2NU
Society.org.uk

LAY SUMMARY

This study will work out the best way to treat children and adolescents aged between 30 days and 15 years who have Kawasaki disease. Kawasaki disease is a disease where arteries, particularly the coronary arteries in the heart, become inflamed, sometimes causing irreversible heart damage, heart attacks or even death. Kawasaki disease is currently the most common cause of acquired heart disease in childhood, and an important preventable cause of heart disease in the young. These heart complications may occur within a few weeks of getting the disease, or more typically, some years after recovery due to narrowing of the coronary arteries causing lack of blood supply to the heart. To prevent this heart damage, the fact that a child or young person has Kawasaki disease has to be recognised by clinicians early, and promptly treated with anti-inflammatory medicines.

The problem is that Kawasaki disease presents with a range of symptoms that are common in normal childhood infections, including a high fever for five days or more, rash, bloodshot eyes, “strawberry” red tongue, cracked, dry lips, swollen lymph glands in the neck, and redness and swelling of the palms and soles. No one knows what causes Kawasaki disease, and this is an area of ongoing and intense research around the world. Experts suggest that wind borne toxins derived from agriculture might be important triggers, combined with genetic susceptibility, although this is by no means firmly established. As such, there is no laboratory diagnostic test available for Kawasaki disease, and diagnosis therefore depends on early recognition of the clinical features.

Intravenous immunoglobulin (IVIG) is a blood product derived from many different pooled healthy blood donors, containing antibodies naturally produced by the immune system. IVIG is the standard treatment given in Kawasaki disease to “dampen down” inflammatory processes which occur in the first few days of the illness. Many children and adolescents still develop significant heart damage despite IVIG. In the UK, heart damage has been found in 19% of cases despite IVIG; in other countries it is as high as 42%. Corticosteroids (‘steroids’) have been used for decades to treat similar inflammatory conditions, but are not yet widely used as an initial treatment for Kawasaki disease. In this study, we will work out if giving corticosteroids upfront (in addition to IVIG) to children and adolescents with Kawasaki disease across Europe reduces the high rate of heart complications we are currently observing.

All children and adolescents in the study will get the current recommended standard treatment of IVIG and aspirin. They will then be split into two groups, by chance (called “randomisation”). One group will not receive any extra treatment other than the standard IVIG and aspirin. The second group will receive additional treatment with prednisolone (corticosteroids) by mouth (or intravenously, into a vein, if needed) immediately. They will take steroids for around 2-3 weeks, depending on how quickly they get better. Everyone will have frequent assessments of their inflammatory status (temperature and inflammatory blood test markers) to work out whether they still need additional IVIG treatment 2 days after they start treatment. Five days later, they will all be re-evaluated again to work out whether they still need extra treatment if the inflammation has not settled completely. Whichever group they started in, children and adolescents will get any extra treatment they then need.

We will follow children and adolescents for 12 weeks through face-to-face visits (or telephone visits if face-to face is not possible) to find out whether they have had any problems – they will mostly stay in hospital for the first 5-7 days, and there are just three visits after this first week. This duration of follow-up is standard for routine clinical care of Kawasaki disease. We will particularly focus on

- looking for any damage to their coronary arteries (or other heart damage) using heart ultrasound (echocardiography) scans;

DocuSign Envelope ID: 651725BB-F200-474E-A72C-A62C1F4636C1

- whether they experienced any side effects from the medicines they received;
- whether they needed to receive any additional treatments; how long they had to stay in hospital;
- whether they have to be admitted to hospital again;
- how rapidly their blood tests normalised;
- how much all their care costs; and
- if our treatments overall improve their quality of life.

In summary, in this study we will answer all of the following questions to work out the best way to treat children and adolescents aged 30 days to 15 years who have Kawasaki disease:

1. Does the combination of corticosteroid and IVIG/aspirin reduce the rate of heart complications in children/adolescents with Kawasaki disease across Europe? (main question)
2. Does the combination of corticosteroid and IVIG/aspirin reduce the length of stay in hospital for children/adolescents with Kawasaki disease, and do their blood test results improve faster?
3. What side effects do children/adolescents get with corticosteroids or other therapies to treat Kawasaki disease?
4. Is the combination of corticosteroid therapy and IVIG/aspirin a cost effective treatment for the management of Kawasaki disease?

DocuSign Envelope ID: 651725BB-F200-474E-A72C-A62C1F4636C1

TRIAL SCHEMA

Figure 1: KD-CAAP trial schema

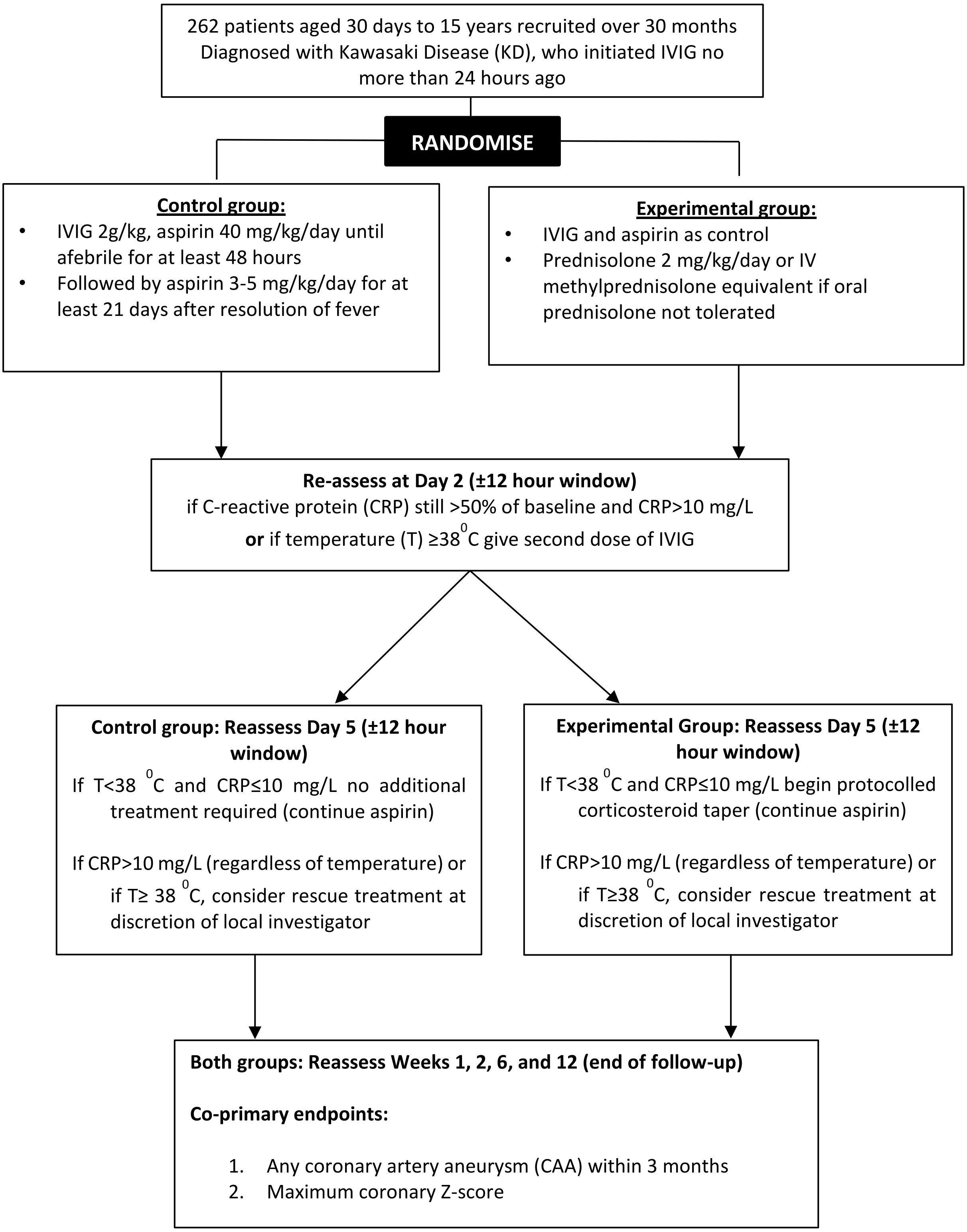

SUMMARY OF TRIAL

INFORMATION TYPE	SUMMARY DETAILS
**Short title**	KD-CAAP (Kawasaki Disease Coronary Artery Aneurysm Prevention trial)
**Long Title of Trial**	Multi-centre, randomised, open-label, blinded endpoint assessed, trial of corticosteroids plus intravenous immunoglobulin (IVIG) and aspirin, versus IVIG and aspirin for prevention of coronary artery aneurysms (CAA) in Kawasaki disease (KD)
**Version**	5.0
**Date**	22 October 2021
**ISRCTN #**	ISRCTN71987471
**EudraCT #**	2019-004433-17
**Trial Design**	Multi-centre, randomised, open-label, blinded endpoint assessed parallel group trial
**Setting**	Hospitals in Europe
**Type of Participants to be Studied**	Patients aged 30 days to 15 years inclusive, with Kawasaki disease (KD) defined by the American Heart Association (AHA) criteria [1] which includes:<br>– fever for at least five days in addition to 4 of 5 additional criteria, or<br>– less than 5 days of fever but otherwise meeting all five AHA clinical criteria, or<br>– incomplete KD cases, as per a modified AHA definition.<br>Patients can be recruited even if they have already received IVIG as long as they are randomised no more than 24 hours after IVIG was initiated.
**Interventions to be Compared**	All patients will receive intravenous immunoglobulin (IVIG) at 2g/kg given as per local standard of care; and aspirin at a dose of 40 mg/kg/day until the patient is afebrile for at least 48 hours, thereafter at 3-5 mg/kg/day for at least 21 days after the fever resolves as per standard of care.<br>Patients will be randomised to<br>▪ **Control group:** no additional initial treatment<br>▪ **Experimental group**: additional oral prednisolone at 2 mg/kg/day or intravenous methylprednisolone at 1.6 mg/kg/day if oral prednisolone is not tolerated.<br>In both groups, patients will be assessed at day 2 (+/-12h) and will receive a second dose of IVIG if they have CRP>50% of baseline and still > 10 mg/L, OR temperature (T) ≥38 °C.<br>At day 5 (+/-12h) further management is again dictated by temperature and CRP:<br>(i) If CRP≤10 mg/L and T<38 °C, no further additional treatment is required. Aspirin should be continued as per above, and children/adolescents in the experimental group should begin tapering corticosteroids.<br>(ii) If CRP>10 mg/L (regardless of temperature) or T≥38 °C rescue treatment should be considered at discretion of local investigator.
**Trial Hypothesis**	Adding immediate corticosteroid treatment to standard of care IVIG and aspirin will reduce coronary artery aneurysm (CAA) rates in unselected KD patients across Europe compared with IVIG and aspirin alone.
**Primary Outcome Measure(s)**	KD-CAAP will have two co-primary outcome measures based on repeat echocardiography undertaken at weeks 1, 2, 6 and 12 weeks:<br>(i) Any CAA documented within the 12 weeks of trial follow-up (to assess overall effectiveness of the strategy of immediate corticosteroids in preventing CAA, expecting that some patients will receive rescue treatment before reaching this endpoint in both groups)

DocuSign Envelope ID: 651725BB-F200-474E-A72C-A62C1F4636C1

INFORMATION TYPE	SUMMARY DETAILS
	(ii) An average estimate across weeks 1, 2, and 6 of the maximum Z-score of the internal diameters of the proximal right coronary artery or left anterior descending coronary artery, adjusting for rescue treatment (to assess the efficacy of corticosteroids).
**Secondary Outcome Measure(s)**	**Efficacy secondary outcomes**<br>(i) At each of weeks 1, 2, 6 and 12 individually, the maximum coronary Z-score<br>(ii) CAA defined solely by a luminal internal diameter z-score of ≥2.5<br>(iii) Receipt of rescue treatment<br>(iv) Receipt of second dose of IVIG<br>(v) Duration of fever after enrolment (time to temperature <38 °C)<br>(vi) Daily serum concentrations of CRP from days 1-5, and at 1 and 2 weeks after enrolment, and time to normalisation of CRP (≤10 mg/L)<br>(vii) Duration of hospitalisation<br>**Safety secondary outcomes**<br>(viii) Serious adverse events including deaths<br>(ix) Grade 3 or 4 adverse events<br>(x) Clinical adverse events of any grade judged related to IVIG, aspirin or corticosteroids
**Other outcomes**	(i) Changes in other laboratory parameters of inflammation<br>(ii) Duration of corticosteroid therapy<br>(iii) Cumulative weight adjusted dose of prednisolone or methylprednisolone received<br>(iv) Proportion of patients who need to continue prednisolone at 2 mg/kg/day beyond day 5 within the experimental group<br>(v) Paediatric appropriate health-related quality of life scores<br>(vi) Paediatric corticosteroid toxicity index (pGTI) to assess glucocorticoid related morbidity [2]<br>(vii) Incremental costs and cost-effectiveness; budget impact.
**Randomisation**	Patients who fulfil the eligibility criteria and have provided informed consent will be randomised 1:1 to receive immediate adjunctive open-label corticosteroids or not, plus standard of care IVIG and aspirin. Minimisation (with a random element) will be used to force balance between randomised groups (i.e. stratify randomisation) according to country, age (<1 versus ≥1 years) and sex.
**Number of Participants**	262 children/adolescents with KD (131 in each group)
**Duration**	Each child/adolescent will be followed for 12 weeks from randomisation. The trial will recruit over 30 months.
**Sub-studies**	**Health economics:** to estimate whether the use of adjunctive corticosteroids is a cost-effective intervention to prevent CAA in KD.<br>**Pharmacometric analysis:** to estimate associations between drug doses and pharmacodynamic (PD) endpoints for efficacy and corticosteroid toxicity (in the experimental group).<br>**Biomarker signatures:** to validate a 13-transcriptomic signature that distinguishes patients with KD from patients with bacterial, viral, and other inflammatory illnesses [3].
**Funder**	Innovative Medicines Initiative 2 Joint Undertaking (JU), under grant agreement No 777389 that supports the Conect4children (c4c) research consortium
**Sponsor**	University College London

DocuSign Envelope ID: 651725BB-F200-474E-A72C-A62C1F4636C1

INFORMATION TYPE	SUMMARY DETAILS
**Chief Investigators**	Professor Despina Eleftheriou, Professor Paul Brogan

DocuSign Envelope ID: 651725BB-F200-474E-A72C-A62C1F4636C1

TRIAL ASSESSMENT SCHEDULE

Table 1: Trial Assessment Schedule

	Screening	D0*	D1	D2	D3	D4	D5	W1	W2	W6	W12	Unscheduled visit
Patient information sheet	X											
Assessment of eligibility criteria	X											
Informed consent (including for sample storage and genetic tests)	X											
Randomisation		X										
History & physical examination, vital signs [1]	X	X	X	X	X	X	X	X	X	X	X	X
Haematology [2]	[X]	X†	[X]	X	[X]	[X]	X	[X]	X	X	[X]	[X]
C-reactive protein	[X]	X†	X	X	X	X	X	X	X	X	[X]	[X]
Biochemistry [3]	[X]	X†	[X]	X	[X]	[X]	X	[X]	X	X	[X]	[X]
Urinalysis [4]	[X]**	X†	[X]	X			X	X	X	X	X	
ECG	[X]	[X]	[X]	[X]	[X]	[X]	[X]	X	X	X	X	[X]
Echocardiography [5]	[X]**	[X]	[X]	[X]	[X]	[X]	[X]	X	X	X	X	[X]
Concomitant drugs & trial treatment		X	X	X	X	X	X	X	X	X	X	
Health-related Quality of Life and resource utilisation [6]		X						X	X	X	X	
Quality of Life [7]		X									X	
pGTI								X			X	
Pregnancy test [8]	X										X	
**Research samples to be stored (scientific substudies):**												
- Plasma serum storage (0.5-1 ml EDTA and 0.5-1 ml serum)		X		X			X		X	X		
- EDTA blood for DNA storage (0.5-1 ml)		X										
- RNA pax gene tube for RNA extraction (2-2.5 ml)		X		X			X		X			
- Throat swab		X										
Maximum total blood draw for research samples (ml)		5.5	0	4.5	0	0	4.5	0	4.5	2	0	0

DocuSign Envelope ID: 651725BB-F200-474E-A72C-A62C1F4636C1

* randomisation occurs on Day 0 (D0).

** microscopy of urine for white cell count and echocardiography are only mandated at screening if required as inclusion criterion for incomplete KD.

† do not need to be repeated at randomisation if values are available on the day of randomisation or the day before randomisation from routine tests.

[] indicates test not mandatory for the trial but results will be collected if available from routine care.

[1] Including vital signs (temperature, heart rate, blood pressure (BP), adverse events. Height (or length in young children) will be assessed at D0, W1, W2, W6, and W12. For children still febrile and in hospital on D5 daily maximum temperatures will be collected until discharge or until afebrile for 2 calendar days to allow assessment of the secondary endpoint 'duration of fever after enrolment'. Weight will be assessed at D0, W6 and W12.

[2] Haematology: Hb, MCV, WCC, lymphocytes, neutrophils, platelets, erythrocyte sedimentation rate (ESR)

[3] Biochemistry: urea, creatinine, aspartate aminotransferase (AST), alanine aminotransferase (ALT), bilirubin, sodium, potassium, albumin, calcium, phosphate, glucose, alkaline phosphatase (ALP), lactate dehydrogenase (LDH)

[4] Urinalysis for proteinuria, haematuria, glycosuria

[5] Echocardiography baseline scan is not required for eligibility.

[6] Child Health Utility 9D (CHU9D) questionnaire and EQ-5D-Y (youth version), depending on age, see **Table 6**, and if questionnaires are available in respective countries language. Should be completed at least one of W1 or W2 depending discharge. Resource utilisation will not be collected at baseline.

[7] Paediatric quality of life (PedsQL™).

[8] Urine or blood pregnancy test must be completed for adolescents who have begun menstruation

Following randomisation, a window of 12 hours either side of each trial visit will be permissible for visits up to D5, up to 1 day before and 3 days after for the week 1 assessment, up to 3 days either side of the week 2 assessment and up to 14 days either side for the week 6 and week 12 assessments. A visit on any given day from randomisation will only be counted once against the scheduled visit to which it is closest.

An unscheduled visit should be used to report a visit if it includes required assessment(s) that were not collected at the nearest scheduled visit, or to report any significant clinical event.

Every attempt should be made to keep the blood draw (including any losses in the manoeuvre) for research samples within the 3% of the total blood volume recommended for children/adolescents during a period of 6 weeks for all children weighing over 8.7kg and will not exceed 1% at any single time for all children weighing over 6.8kg (since the total volume of blood is estimated at 80 to 90 ml/kg body weight, 3% equates to 2.4ml blood per kg body weight [4]. For any enrolled children lighter than 6.8kg, the clinician should endeavour to ensure that the total blood volume for research samples does not exceed 1% at any single time. Since KD is an acute, severe illness, bloods required for routine clinical may occasionally surpass these limits as is often the case when managing critically ill children in routine clinical care.

Most children will remain in hospital for the first 5-7 days in the trial and will have cannulae for medication through which blood draws can be made.

DocuSign Envelope ID: 651725BB-F200-474E-A72C-A62C1F4636C1

CONTENTS

GENERAL INFORMATION 3

LAY SUMMARY 8

TRIAL SCHEMA 10

SUMMARY OF TRIAL 11

TRIAL ASSESSMENT SCHEDULE 14

CONTENTS 16

ABBREVIATIONS 20

BACKGROUND 23
1.1 KAWASAKI DISEASE (KD): EPIDEMIOLOGY, AETIOPATHOGENESIS AND DIAGNOSTIC CRITERIA 23
1.1.1 Epidemiology of KD 23
1.1.2 Aetiopathogenesis of KD 23
1.1.3 Diagnosis of KD 24
1.2 TREATMENT AND OUTCOME OF KD 25
1.2.1 Current Treatment of KD and Reported Rates of Coronary Complications across Europe 25
1.2.2 Patients with KD Complicated by CAA Have a Poor Cardiac Prognosis which could be Preventable 26
1.3 CORTICOSTEROIDS FOR THE TREATMENT OF KD 27
1.3.1 Cumulative Efficacy Data for Corticosteroids in KD 27
1.3.2 Cumulative Safety Data for Corticosteroids in KD 28
1.4 RATIONALE FOR KD-CAAP 29
1.5 HYPOTHESIS AND AIMS OF KD-CAAP 30
1.6 BENEFIT-RISK ASSESSMENT 31

SELECTION OF SITES/CLINICIANS 34
2.1 SITE/INVESTIGATOR INCLUSION CRITERIA 34
2.2 PI'S QUALIFICATIONS & AGREEMENTS 34
2.3 SITE ASSESSMENT 35
2.4 APPROVAL AND ACTIVATION 35

SELECTION OF CHILDREN 36
3.1 INCLUSION CRITERIA 36
3.2 EXCLUSION CRITERIA 37
3.3 NUMBER OF CHILDREN/ADOLESCENTS TO RECRUIT 38
3.4 CO-ENROLMENT GUIDELINES 38
3.5 SCREENING PROCEDURES & PRE-RANDOMISATION INVESTIGATIONS 38
3.6 SAMPLES TO BE TAKEN AS SOON AS CONSENT IS OBTAINED 38

ENROLMENT & RANDOMISATION 40
4.1 RANDOMISATION PRACTICALITIES 41
4.2 CO-ENROLMENT GUIDELINES AND REPORTING 41

DocuSign Envelope ID: 651725BB-F200-474E-A72C-A62C1F4636C1

TRIAL TREATMENTS 42
5.1 INTRAVENOUS IMMUNOGLOBULIN AND ASPIRIN (BOTH RANDOMISED GROUPS) 42
5.1.1 Products & treatment schedule 42
5.1.2 Stopping Drug Early due to Reaction to IVIG and aspirin 43
5.1.3 Compliance & Adherence 43
5.2 CONTROL GROUP: FURTHER MANAGEMENT BASED ON ASSESSMENT OF FEVER AND CRP RESPONSE ON DAY 2 AND ON DAY 5 43
5.3 EXPERIMENTAL GROUP TREATMENT 44
5.3.1 Products & treatment schedule 44
5.3.2 Dispensing and Accountability of IMP 45
5.3.3 Experimental group: further management based on assessment of fever and crp response on Day 2 and day 5 46
5.3.4 Stopping Drug Early 47
5.3.5 Compliance and Adherence 48
5.4 RECOMMENDED NON-MANDATORY RESCUE THERAPIES FOR BOTH EXPERIMENTAL AND CONTROL GROUP . 48
5.5 HANDLING CASES OF TRIAL MEDICATION OVERDOSE 49
5.6 UNBLINDING / UNMASKING 49
5.7 PROTOCOL TREATMENT DISCONTINUATION 49
5.8 TREATMENT DATA COLLECTION 50
5.9 NON-TRIAL TREATMENT 50
5.9.1 Medications Permitted 50
5.10 MEDICATIONS NOT PERMITTED 50
5.11 TREATMENT AFTER TRIAL EVENT 51
5.12 CO-ENROLMENT GUIDELINES 51

ASSESSMENTS & FOLLOW-UP 52
6.1 TRIAL ASSESSMENT SCHEDULE 52
6.2 BASELINE INFORMATION COLLECTED AT ENROLMENT 53
6.3 INFORMATION TO BE COLLECTED AT ALL FOLLOW UP ASSESSMENTS 53
6.4 INFORMATION TO BE COLLECTED AT WEEKS 1, 2, 6 AND 12 54
6.4.1 Acute events 54
6.5 ECHOCARDIOGRAPHY AND ECG 54
6.6 PROCEDURES FOR ASSESSING EFFICACY 55
6.7 PROCEDURES FOR ASSESSING SAFETY 55
6.7.1 Paediatric Glucocorticoid Toxicity (pGTI) 55
6.8 OTHER ASSESSMENTS 56
6.8.1 Health economics 56
6.8.2 Quality of life measures 56
6.9 EARLY STOPPING OF FOLLOW-UP 57
6.10 LOSS TO FOLLOW-UP 57
6.11 COMPLETION OF PROTOCOL FOLLOW UP 57

SAFETY REPORTING 58
7.1 DEFINITIONS 58
7.1.1 Medicinal Products 59
7.1.2 Adverse Events 59
7.2 OTHER NOTABLE EVENTS 59
7.2.1 Pregnancy 59
7.3 INVESTIGATOR RESPONSIBILITIES 59
7.3.1 Investigator Assessment 60
7.3.2 Notification Procedure 61

DocuSign Envelope ID: 651725BB-F200-474E-A72C-A62C1F4636C1

7.4 MRC CTU RESPONSIBILITIES 61

QUALITY ASSURANCE & CONTROL 63
8.1 RISK ASSESSMENT 63
8.2 CENTRAL MONITORING AT CTU 63
8.3 ON-SITE MONITORING 63
8.3.1 Direct Access to Children's Records 63
8.3.2 Confidentiality 67

STATISTICAL CONSIDERATIONS 68
9.1 METHOD OF RANDOMISATION 68
9.2 OUTCOME MEASURES 68
9.2.1 Primary outcome measure 68
9.2.2 Secondary outcome measures 69
9.2.3 Other outcome measures 69
9.2.4 Protection from bias 69
9.3 SAMPLE SIZE 70
9.4 INTERIM MONITORING & ANALYSES 71
9.5 ANALYSIS PLAN (BRIEF) 71

SUBSTUDIES 73
10.1 PHARMACOMETRIC SUBSTUDY 73
10.2 DIAGNOSTIC BIOMARKER SUBSTUDY 73
10.2.1 Diagnosis 73
10.2.2 Identification of pathogenic mechanisms and aetiology 73
10.2.3 Kawasaki Disease genetics 74

REGULATORY & ETHICAL ISSUES 75
11.1 COMPLIANCE 75
11.1.1 Regulatory Compliance 75
11.1.2 Site Compliance 75
11.1.3 Data Collection & Retention 75
11.2 ETHICAL CONDUCT 75
11.2.1 Ethical Considerations 75
11.2.2 Ethical Approvals 76
11.3 COMPETENT AUTHORITY APPROVALS 76
11.4 TRIAL CLOSURE 76
11.4.1 Sample storage and destruction 76

INDEMNITY 77

FINANCE 78

OVERSIGHT & TRIAL COMMITTEES 79
14.1 TRIAL MANAGEMENT TEAM (TMT) 79
14.2 TRIAL MANAGEMENT GROUP (TMG) 79
14.3 TRIAL STEERING COMMITTEE (TSC) 80
14.4 DATA MONITORING COMMITTEE (DMC) 80
14.5 ROLE OF TRIAL SPONSOR 80

DocuSign Envelope ID: 651725BB-F200-474E-A72C-A62C1F4636C1

PATIENT AND PUBLIC INVOLVEMENT 81

PUBLICATION AND DISSEMINATION OF RESULTS 82

DATA AND/OR SAMPLE SHARING 84

PROTOCOL AMENDMENTS 85

APPENDICES 89

19.1 APPENDIX 1 89

REFERENCES 90

DocuSign Envelope ID: 651725BB-F200-474E-A72C-A62C1F4636C1

ABBREVIATIONS

ABBREVIATION	EXPANSION
AE	Adverse event
AHA	American Heart Association
ALT	Alanine aminotransferase
ALP	Alkaline phosphatase
AR	Adverse reaction
AST	Aspartate aminotransferase
BCG	Bacillus Calmette–Guérin
BP	Blood pressure
c4c	Conect4children
CAA	Coronary artery aneurysm
CF	Consent Form
CHU9D	Child Health Utility 9D
CI	Chief Investigator
CI	Confidence interval
CPM	Clinical Project Manager
CRF	Case Report Form
CRP	C reactive protein
CTU	See MRC CTU at UCL
D	Day
DMC	Data Monitoring Committee
DMP	Data management plan
DNA	Deoxyribonucleic acid
DSMB	Data Safety Monitoring Board
E’	Early diastolic relaxation velocity
ECG	Electrocardiogram
EDTA	Ethylenediaminetetraacetic acid
EMA	European Medicines Agency
ESR	Erythrocyte sedimentation rate
ET	Ejection time
EU	European Union
FBC	Full blood count
FDA	(US) Food and Drug Administration
GCP	Good Clinical Practice
GEE	Generalised Estimating Equations
h	Hours
Hb	Haemoglobin
HRQL	Health-related quality of life

DocuSign Envelope ID: 651725BB-F200-474E-A72C-A62C1F4636C1

ABBREVIATION	EXPANSION
ICER	Incremental Cost Effectiveness Ratio
ICH	International Conference on Harmonisation of Technical Requirements for Registration of Pharmaceuticals for Human Use
IMP	Investigational medicinal product
IQR	Interquartile range
IRB	Institutional Review Board
ISRCTN	International Standard Randomised Controlled Trial Number
IVCT	Isovolumic contraction time
IVRT	Isovolumic relaxation time
ITT	Intention-to-treat
IV	Intravenous
IVIG	Intravenous immunoglobulin
JU	Innovative Medicines Initiative 2 Joint Undertaking
KD	Kawasaki disease
KD-CAAP	Kawasaki Disease Coronary Artery Aneurysm Prevention trial
LAD	Left Anterior Descending
LDH	Lactate dehydrogenase
LMCA	Left Main Coronary Artery
LVEDD	Left Ventricular End Diastolic Diameter
LVESD	Left Ventricular End Systolic Diameter
MCV	Mean corpuscular volume
MOP	Manual of Operations
MRC	Medical Research Council
MRC CTU at UCL	Medical Research Council Clinical Trials Unit at University College London (also generally abbreviated to “CTU”)
MAPSE	Mitral annular plane systolic excursion
NSAID	Non-steroidal anti-inflammatory drugs
PD	Pharmacodynamics
PENTA	Fondazione PENTA - for the treatment and care of children with HIV (and related diseases) – ONLUS
pGTI	Paediatric Glucocorticoid toxicity index
PI	Principal Investigator
PIMS-TS	Paediatric Multisystem Inflammatory Syndrome
PIS	Patient Information Sheet
PK	Pharmacokinetics
PMC	PubMed Central
PRINTO	Paediatric Rheumatology International Trials Organisation
QA	Quality Assurance
QALY	Quality Adjusted Life Year
QC	Quality Control
QMAG	Quality Management Advisory Group

DocuSign Envelope ID: 651725BB-F200-474E-A72C-A62C1F4636C1

ABBREVIATION	EXPANSION
QoL	Quality of Life
R&D	Research and Development
RCT	Randomised controlled trial
RCA	Right coronary artery
REC	Research Ethics Committee
RGC	Research Governance Committee
RNA	Ribonucleic acid
S’	Systolic myocardial velocity
SAE	Serious adverse event
SAP	Statistical Analysis Plan
SAR	Serious adverse reaction
SD	Standard deviation
SHARE	Single Hub Access Point for paediatric Rheumatology in Europe
SOP	Standard operating procedure
SPC	Summary of Product Characteristics
SPIRIT	Standard Protocol Items: Recommendations for Interventional Trials
SSA	Site-specific approval
SSG	Scientific Strategy Group
SUSAR	Suspected unexpected serious adverse reaction
T	Temperature
TDI	Tissue Doppler Imaging
TM	Trial Manager
TMF	Trial Master File
TMG	Trial Management Group
TMT	Trial Management Team
TSC	Trial Steering Committee
UAR	Unexpected adverse reaction
UCL	University College London
UK	United Kingdom
US	United States
W	Week
WBC	White Blood Cell
WCC	White Cell Count

DocuSign Envelope ID: 651725BB-F200-474E-A72C-A62C1F4636C1

BACKGROUND

1.1 KAWASAKI DISEASE (KD): EPIDEMIOLOGY, AETIOPATHOGENESIS AND DIAGNOSTIC CRITERIA

1.1.1 EPIDEMIOLOGY OF KD

Kawasaki disease (KD) is an acute self-limiting inflammatory vasculitis affecting predominantly medium-sized arteries, particularly the coronary arteries causing coronary artery aneurysms (CAA) [1, 5-7]. KD is currently the commonest cause of acquired heart disease in children in high-income countries [1, 5-7]. KD causes CAA in 15-25% of untreated patients while 2-3% of untreated cases die as a result of coronary vasculitis [1, 5-7]. Coronary artery vasculitis can cause acute myocardial events in the early stages of the disease leading to myocardial infarction or even death [1, 5-7]. Late morbidity can also arise from late KD vasculopathy, a process involving remodelling following the acute inflammatory event, distinct from atherosclerosis, but ultimately leading to coronary vascular insufficiency and late cardiac events [1, 5-7]. Notably, as more children with KD survive into adulthood, the disease remains an important cause of long-term cardiac disease in adulthood and requires rigorous follow-up, particularly for those with CAA, to reduce risk of myocardial ischaemia and infarction [1, 5-7].

The disease has a world-wide distribution with a male preponderance (male: female ratio of 1.5: 1), seasonality and occasional epidemics [1, 5-7]. KD is more prevalent in Japanese children (308/100,000 under the age of five years) [8]. An increased incidence of KD is also observed in Japanese and other Asian children resident in North America and Europe, suggesting a genetic contribution [8-10]. In the UK, a recent direct British Paediatric Surveillance Unit epidemiological survey (2013-2015) showed that the incidence of KD in the UK and Ireland was 4.55/100,000 children under 5 years, which represents a slight increase since the last survey in 1990 [7, 11]. Whilst the majority of cases were Caucasian, KD in the UK is over-represented in Chinese or Japanese Asians and Black Africans [7]. Other recent studies have demonstrated a higher incidence of KD of 25/100,000 children <5 years in the US [1]; and 5.5/100,000 children < 5 years in Skane, Sweden [12]. Mortality of KD varies by population: 0.015% in Japan; 0.17% in the USA and 0.36% in the UK [1, 7, 10].

1.1.2 AETIOPATHOGENESIS OF KD

The aetiology of KD remains unknown. Pronounced seasonality and clustering of KD cases have led to the hunt for infectious agents as a cause [1, 7, 10]. Many published reports implicate a number of bacterial and viral pathogens, including retroviruses, Epstein-Barr virus, coronavirus, *Propionibacterium acnes*, staphylococcal and streptococcal superantigens, and unidentified virus particles as infectious triggers of KD [13-16]. So far, however, no single agent has been consistently identified [1, 15, 17, 18]. One line of investigation suggests infection with a novel RNA virus that enters through the upper respiratory tract [16]. Intracytoplasmic inclusion bodies in bronchial epithelial cells and multiple other cell types throughout the body appear to contain RNA and could be linked to the KD agent [16]. Efforts to characterise the molecular details of these inclusion bodies have been hampered, however, by a paucity of autopsy tissues available for study. Other studies have implicated tropospheric windborne agents, perhaps arising from agriculture, as potential triggers of KD [19-21], although this is not definitively proven.

The genetic contribution to the risk of KD has long been suggested by much higher risk of the disease in Asian children, particularly Japanese and Koreans, which persists when patients of these ethnicities migrate to other countries. Other clues pointing to a genetic contribution include the observed

DocuSign Envelope ID: 651725BB-F200-474E-A72C-A62C1F4636C1

increased relative risk to siblings of index cases compared to the general population; from twin studies; and lastly from well-documented familial cases [22-24]. Polymorphisms in several candidate genes have previously been suggested, either as susceptibility genes for developing KD; or for increasing risk of CAA should KD develop [25, 26]. Most of these earlier studies, however, failed to identify definitive genetic associations, emphasizing the difficulties of the candidate gene approach for a disease where the pathogenesis is poorly understood. More recently, a number of genome wide association studies of KD were reported [27-33]. From these studies, several single nucleotide polymorphisms were found to be associated with susceptibility to KD, including *ITPKC*, *ABCC4*, and *FCGR2A*; *CD40*; and a gene region near *FAM167A-BLK* [27-33]. Polymorphisms in other genes have been associated with non-response to IVIG and risk of developing CAA, including *CASP3*, and *FCGR3B* [27-33]. It is likely that many other genetic factors have yet to be identified, and the study of the genetic contribution to KD remains an intense area of ongoing research worldwide.

1.1.3 Diagnosis of KD

There is no diagnostic test for KD, thus the diagnosis rests on clinical criteria, supported with laboratory findings for atypical cases [1]. Diagnostic criteria for KD according to the American Heart Association (AHA) guidelines [1], also endorsed by the pan-European Single Hub Access Point for paediatric Rheumatology in Europe (SHARE) guidance [5], are fever with duration of 5 days or more PLUS 4 of 5 of the following:

1. Conjunctivitis: bilateral, bulbar, conjunctival injection without exudate
2. Lymphadenopathy: cervical, often >1.5 cm usually unilateral
3. Rash: maculopapular, diffuse erythroderma or erythema multiform
4. Changes of lips or oral mucosa: red cracked lips; "strawberry" tongue; or diffuse erythema of oropharynx
5. Changes of extremities: erythema and oedema of palms and soles in acute phase; and periungual desquamation in subacute phase.

Both the AHA and SHARE guidelines acknowledge that diagnosis may be made earlier than day 5 of fever if fever plus ≥4 principle clinical features are present (see below) [1, 5]. Important caveats exist around overly strict adherence to these diagnostic criteria: many patients (particularly infants under the age of 12 months) have some but not all of the clinical features of KD, but may still be at high risk of CAA (see below). Secondly, clinical features may present sequentially such that an ‘incomplete’ case can evolve into a ‘complete’ case [1, 5]. Thus, the diagnosis of KD must be considered in any child/adolescent with a febrile exanthematous illness particularly if it persists longer than 4 days, and especially in infants younger than 12 months [1, 5].

1.1.3.A Diagnosing KD before Day 5 of fever

Whilst duration of fever has historically been important for standardising case definitions, there should be no delay in making a diagnosis of KD and instituting treatment before day 5 of fever if (i) sufficient criteria are present to fulfil a complete KD diagnosis before day 5 of fever; (ii) CAA or coronary dilatation are present, or (iii) there is evidence of persistent elevation of inflammatory markers and/or persistent fever, especially in infants or younger children without other explanation [1, 5].

In a patient in whom KD is suspected but all criteria have not yet been fulfilled, two other clinical signs which may strengthen the diagnostic suspicion include: disproportionate or marked irritability; and new erythema or induration at the site of previous BCG immunisation [1, 5]. The exact mechanism of the irritability is unclear, but may be related to the presence of aseptic meningitis [1, 5]. The

DocuSign Envelope ID: 651725BB-F200-474E-A72C-A62C1F4636C1

mechanism of erythema and induration at the site of previous BCG immunisation is believed to be due to cross reactivity of T cells in KD patients between specific epitopes of mycobacterial and human heat shock proteins [34].

1.1.3.B Diagnosing incomplete KD

Whilst the diagnosis of KD is generally straightforward in patients fulfilling all the criteria for KD ('complete' KD), many patients have only some of the clinical features, termed 'incomplete' KD [1, 5]. These cases may still be at risk of CAA and for some patients represent the highest risk group, particularly infants who may have prolonged fever alone, or only fleeting clinical signs [1, 5]. Diagnosing incomplete KD thus relies on a high index of suspicion [1, 5]. In these situations, early echocardiography is recommended [1, 5]. This may reveal evidence of coronary vasculitis, confirming the diagnosis of 'complete' KD [1, 5]. Notably, however, a negative echocardiogram does not exclude KD, and thus should not deter treatment if suspicion remains high [1, 5]. The AHA 2017 and SHARE recommendations provide some guidance regarding the diagnosis of incomplete KD [1, 5], specifically that a diagnosis of incomplete KD should be considered in:

(i) children (>1 year old) with fever for ≥ 5 days AND at least 2 other compatible clinical criteria as listed above; OR infants ≤ 1 year old with fever ≥ 7 days without other explanation;

AND for both age groups:

(ii) CRP ≥30 mg/L or erythrocyte sedimentation rate (ESR) ≥40 mm/h AND

(iii) EITHER the presence of any 3 or more of: anaemia for age (haemoglobin less than the lower limit of normal laboratory reference range for age); platelet count ≥ 450 X10^9/L, or < 140 X10^9/L; albumin < 30 g/L; elevated ALT; white cell count ≥ 15 X10^9/L; urine ≥ 10 white blood cells per high power field)

(iv) OR abnormal echocardiogram compatible with KD but without established CAA, with ≥ 3 of the following suggestive features: decreased left ventricular function, mitral regurgitation, pericardial effusion, or dilated but non-aneurysmal coronary arteries (2≤Z-score<2.5; and not meeting the other criteria for aneurysmal change as defined below).

In summary, KD is an inflammatory vasculitis that has a rising worldwide incidence and causes significant cardiovascular morbidity in childhood leading to an increased burden of preventable cardiac disease in adult life.

1.2 TREATMENT AND OUTCOME OF KD

1.2.1 CURRENT TREATMENT OF KD AND REPORTED RATES OF CORONARY COMPLICATIONS ACROSS EUROPE

Randomised controlled trials and meta-analyses have unequivocally demonstrated that early recognition and treatment of KD with intravenous immunoglobulin (IVIG) and aspirin reduces the occurrence of CAA [35, 36]. Therefore, IVIG and aspirin should be started as soon as a patient is diagnosed with complete or incomplete KD [1, 5, 6, 37]. Two g/kg of IVIG is the optimal dose, usually given as a single infusion (typically over 12 hours), in view of greater therapeutic effect in preventing CAA when compared to a lower, divided dose regimen [38]. Close monitoring of patients is critical, taking into account temperature, acute phase reactants (particularly C-reactive protein (CRP)), clinical symptoms and other signs of systemic inflammation.

All patients should initially receive aspirin at a dose of 30-50 mg/kg/day, in 3-4 divided doses [5, 6]. A recent review of 6 published studies employing different doses of aspirin (30-50 mg/kg/day dose compared to higher-doses of 80–120 mg/kg/day) in the acute treatment of KD concluded that there was no clear evidence favouring higher doses of aspirin over more moderate anti-inflammatory doses,

DocuSign Envelope ID: 651725BB-F200-474E-A72C-A62C1F4636C1

and highlighted the adverse effects of higher dose aspirin such as bleeding [39]. Aspirin should be reduced to an antiplatelet dose of 3-5 mg/kg/day, but only after the fever has settled for 48 hours, clinical features are improving, and CRP levels are falling in line with CRP half-life (approximately 18 hours in the absence of ongoing hepatic production) [1, 5, 6, 37]. If CAA persist in the convalescent phase, continuation of low-dose aspirin (3-5 mg/kg/day) is recommended long-term [1, 5, 6]. In patients with resolved CAA, long-term aspirin (3-5 mg/kg/day) should still be considered, taking into account the risk-benefit ratio for individual patients, because it is increasingly recognised that patients with regressed aneurysms may demonstrate coronary artery endothelial function abnormalities comparable to those with persistent CAA [1, 5, 6, 37, 40]. Ibuprofen and other nonsteroidal anti-inflammatory drugs interfere with the antiplatelet effect of aspirin and thus should be avoided if possible, a point emphasised in the recent AHA guidelines [1]. It is possible that future guidance may recommend low dose aspirin (3-5 mg/kg/day) from the outset, as suggested by data from a retrospective cohort [41] ; there has never been a prospective controlled clinical trial to support this approach, however, and thus at the moment this cannot be routinely recommended

Early recognition and treatment of KD with aspirin (30-50 mg/kg/day, in 3-4 divided doses) and intravenous immunoglobulin (IVIG; 2 g/kg) should theoretically reduce the risk of occurrence of CAA from approximately 20% in untreated patients to 4% [1, 5-7]. IVIG resistance occurs in up to 20-40% of cases, however, and is associated with increased risk of developing CAA [1, 5-7].

Notably however, several recent studies conducted in Europe (UK, Sweden, and Germany), Russia, and North America have found alarmingly high rates of coronary complications despite IVIG [7, 12, 42, 43]. A recent UK survey (2013-2015) suggested that 19% of children with KD developed CAA despite IVIG; and, even more worryingly, 39% of those under 1 year old developed CAA [7]. Similarly, in Germany overall CAA rates of 22% (42% in younger children) have been reported despite treatment with IVIG [44]. In Skane, Sweden, the rate of CAA in a recent survey was reported as 16% despite IVIG, with 45% under the age of 1 year developing CAA [12].

The reasons for these alarmingly high rates of CAA are currently unknown. Late diagnosis is of concern (particularly for those with incomplete KD) and delayed treatment undoubtedly plays a role, since in the latest UK survey, time to IVIG treatment was delayed in those with CAA compared with those without CAA. Additionally, Caucasians may not respond as well to IVIG as non-Caucasians, perhaps due to an as yet unidentified gene-dosing risk effect, such as (speculatively) Fc gamma-receptor polymorphisms that may influence IVIG- responsiveness in different populations [5]. Whatever the reason(s), these very high CAA complication rates now emphasise the need for an urgent reappraisal of IVIG as the primary therapeutic agent for KD.

1.2.2 Patients with KD Complicated by CAA Have a Poor Cardiac Prognosis which could be Preventable

Children who develop CAA (internal coronary diameter Z-score ≥2.5) from KD require lifelong specialist follow-up to monitor for coronary sequelae [1, 5]. A study of 1356 patients diagnosed from 1990-2007 and followed up with serial echocardiograms for up to 15.7 years revealed that subsequent coronary artery events (thrombosis, stenosis, intervention, myocardial infarction) or death occurred in 1% of those with an aneurysm Z-score <10 and an absolute dimension <8mm; in 29% of those with Z-score ≥10 but an absolute dimension <8mm; and in 48% of those with Z-score ≥10 and absolute coronary aneurysm internal diameter ≥8mm [1, 45]. Other studies have confirmed this poor long-term prognosis for those with so-called 'giant' CAA [1, 46]. In fact, an estimated 5% of young adults presenting with myocardial ischaemia have had KD [47]. Even if CAA resolve, it is increasingly recognised that coronary artery function remains abnormal, although the long-term prognostic significance of this is uncertain [40]. In contrast, KD patients without CAA have no symptoms or

DocuSign Envelope ID: 651725BB-F200-474E-A72C-A62C1F4636C1

coronary events on long-term follow-up. Therefore, optimizing treatment in the acute phase of KD would prevent future morbidity from long-term damage to the heart, thus improving outcomes for patients, and reducing healthcare costs associated with lifelong cardiac treatment and follow-up.

1.3 CORTICOSTEROIDS FOR THE TREATMENT OF KD

1.3.1 Cumulative Efficacy Data for Corticosteroids in KD

Corticosteroids are an effective treatment for virtually all forms of vasculitis, but they have not been widely adopted as first-line treatment in unselected KD cases. This is largely due to conflicting efficacy data from previous clinical trials in non-European patients using very different corticosteroid dosing regimens in patients with differing risk profiles for CAA [1, 5, 48-51]. This is illustrated when the American Paediatric Heart Network trial and the recently reported Japanese RAISE trial (two of the largest RCTs examining this issue) are considered in more detail [51, 52]. Both these trials investigated the use of corticosteroids in addition to standard IVIG/aspirin. The American trial evaluated the use of intravenous methylprednisolone (30 mg/kg) given as a single dose combined with IVIG in unselected patients with KD [52]. In contrast, the RAISE trial evaluated lower-dose (2 mg/kg) intravenous methylprednisolone given for 5 days; if fever settled, this was then converted to oral prednisolone which was subsequently tapered over 15 days after the C-reactive protein (CRP) normalised [51]. Moreover, patients were included in RAISE only if they were at high risk of IVIG resistance, based on a risk score (Kobayashi score ≥5) [51]. Perhaps unsurprisingly then, these two studies produced different results, with corticosteroids conferring significant benefit in the Japanese RAISE trial, but a lack of overall benefit in the American trial, probably because this latter trial did not use enough corticosteroid. Importantly, a large "post-RAISE" observational study of 724 high-risk Japanese patients receiving corticosteroid treatment in addition to IVIG/aspirin showed that primary IVIG plus prednisolone therapy had an effect similar to that seen in the RAISE trial, and significantly reduced the incidence of CAA with minimal adverse events [53]. This observational analysis provides additional "real-world" support for the use of corticosteroids in high-risk KD cases in Japan [53].

Notably, meta-analysis of data from several published studies of corticosteroid therapy in KD also provide compelling evidence supporting the use of corticosteroids as primary adjunctive treatment for patients with severe KD [54]. Meta-analysis of 16 comparative studies (mostly observational, few randomised) involving 2,746 KD patients demonstrated that early addition of corticosteroids to conventional IVIG therapy was associated with reduced risk of CAA compared with IVIG therapy alone (odds ratio 0.424; 95%CI, 0.270-0.665) [54]. This benefit was only observed when corticosteroids were used as primary therapy rather than rescue therapy for IVIG resistance, and was greatest for Japanese patients who were determined at baseline to have high-risk for IVIG resistance [54]. Meta-regression analyses also demonstrated that corticosteroids were more effective when started earlier in the disease course [54]. Overall, this meta-analysis provided convincing evidence that corticosteroids combined with IVIG as initial treatment reduces overall risk of CAA in severe KD, but did not resolve the ongoing debate about which KD patients should receive this, probably explaining why less than 5% of patients in the UK currently receive corticosteroids as primary adjunctive treatment [7]. A high risk patient is regarded as one where the risk of CAA is 20-30% despite IVIG treatment, and in Japan is identified as those patients with a Kobayashi score ≥5 [50]. For UK and European patients, however, the Kobayashi risk score had poor sensitivity to identify patients at higher risk of CAA [48, 49]. In line with this evidence, recent European SHARE guidelines for KD recommend adjunctive corticosteroids for high-risk patients, but acknowledge that identifying such patients in Caucasian populations is difficult, and that clinical scores to define high risk patients developed for Japanese patients perform sub-optimally in Caucasians [5].

DocuSign Envelope ID: 651725BB-F200-474E-A72C-A62C1F4636C1

However, given the aforementioned worryingly-high CAA rates emerging from several countries, and the lack of risk assessment tools to accurately identify such cases, it is reasonable now to argue that all European KD patients are at significant risk of CAA despite IVIG (19-45%) [7, 12, 44], and could potentially benefit from primary treatment with corticosteroids.

1.3.2 Cumulative Safety Data for Corticosteroids in KD

Several previous clinical trials have explored the efficacy and safety of adjunctive corticosteroids in mainly non-European patients with severe KD [51, 54, 55]. Despite seemingly conflicting efficacy results, largely due to different corticosteroid dosing regimens and heterogeneous KD patient groups in relation to stratification for CAA risk, these studies all indicated that short courses of corticosteroids were safe in KD [51, 54, 55]. Specifically, in the American Paediatric Heart Network trial [52] that evaluated the use of intravenous methylprednisolone (30 mg/kg) given as a single dose combined with IVIG in unselected patients with KD (experimental group), compared to controls who received placebo plus IVIG, there was no evidence of differences in adverse events reported in both trial groups: 37/101 (36%) in the experimental group compared to 24/97 (25%) in the control group, p=0.18. The majority of these adverse events were judged to be related to IVIG use, and were not related to corticosteroids. The few adverse events clearly attributed to corticosteroid use, observed in only 5/101 patients, included hypotension, and one episode of hypokalaemia; all quickly resolved with no intervention [52].

Similar favourable safety data have been reported from studies of corticosteroids in Japan [51, 55]. The Japanese RAISE trial [51] evaluated lower-dose (2 mg/kg) intravenous methylprednisolone given for 5 days in high-risk, severe (i.e. Kobayashi score ≥5) Japanese patients; when fever settled, this was then converted to oral prednisolone, tapered over 15 days after normalisation of CRP; versus a control group who received IVIG alone [51]. Again, the adverse event profile for both groups was comparable: serious adverse events occurred in 3/121 (2%) patients in the experimental group and 2/121 (2%) in the control group [51]. The types of serious adverse events were also similar between both groups: in the intravenous immunoglobulin plus prednisolone group, two patients had high total cholesterol, and one had neutropenia; and in the intravenous immunoglobulin group, one patient had high total cholesterol, and there was one episode of non-occlusive thrombus [51].

Importantly, observational data involving 724 high-risk Japanese patients (again identified by a high Kobayashi score) routinely treated with adjunctive corticosteroids also demonstrated minimal adverse events relating to corticosteroids, which occurred in only 2/724 patients (hypertension (N=1); bacteraemia (N=1) [53]. Several more side effects were reported in relation to IVIG therapy, however [53]. This observational analysis therefore provides reassurance regarding the safety of corticosteroids in high-risk Japanese KD cases [53].

Lastly, meta-analysis of 16 comparative studies of 2,746 patients with KD supported the use of corticosteroid therapy in severe KD (see above), and highlighted that efficacy was conferred without an increased risk of corticosteroid-related adverse events [54].

These data therefore suggest that overall corticosteroids are a safe treatment for KD.

DocuSign Envelope ID: 651725BB-F200-474E-A72C-A62C1F4636C1

1.4 RATIONALE FOR KD-CAAP

As summarised above, a number of recent studies conducted in different European countries (UK, Sweden, and Germany), Russia, and the United States have recently demonstrated alarmingly high rates of coronary complications despite IVIG [7, 33, 43, 44]. CAA rates ranged from 16% in the Swedish study (45% in infants under 12 months), to as high as 42% in younger children in the German survey [7, 33, 43, 44]. In the UK, a recent survey (2013-2015) suggested that 19% of children with KD still developed CAA despite IVIG; and, even more worryingly in that survey, 39% of patients under the age of 1 year developed CAA despite IVIG [7]. These high complication rates now emphasise the need for an urgent reappraisal of IVIG and aspirin as the primary therapeutic agents for KD.

We have recently published evidence-based, consensus European guidelines for the diagnosis and treatment of KD [5] and in doing so clearly observed:

1. Higher CAA rates than previously considered to be associated with IVIG-treated KD;
2. Important evidence gaps regarding lack of an appropriate clinical tool to stratify patients at highest risk of CAA outside of Japan [48, 49], and hence who to target for more aggressive treatment; and
3. Significant equipoise among the paediatric community across Europe regarding the use of adjunctive treatments for unselected KD cases [5].

These observations have been important drivers for a trial to improve KD outcomes across Europe.

Corticosteroids are an effective treatment for virtually all forms of vasculitis, but they have not been adopted as first-line treatment of unselected KD cases, for which there remains significant equipoise [1, 5]. Increasingly compelling evidence summarised above from randomised controlled trials and meta-analyses supports corticosteroid use as primary adjunctive treatment for patients with severe KD, particularly for Japanese patients with a Kobayashi score ≥5, and for whom CAA risk is 20-30% despite IVIG [54]. This does not, however, resolve the ongoing debate about which KD patients should be considered as “severe” outside of Japan, since the Kobayashi score and other clinical severity-scoring systems have poor predictive value in non-Japanese patients [48, 49]. Given however, the aforementioned high CAA complication rates seen across Europe (16-45%), all KD patients are arguably at high-risk of CAA despite IVIG, and could therefore potentially benefit from adjunctive corticosteroids as primary treatment for KD [7, 12, 44]. Therefore there remains significant equipoise regarding the use of corticosteroids as primary treatment combined with IVIG for all patients, i.e. not just the most severe cases.

This protocol therefore describes a multi-centre randomised, controlled, open-label, blinded endpoint assessed, trial to explore the efficacy and safety of adjunctive corticosteroid therapy combined with IVIG/aspirin, versus IVIG/aspirin alone in unselected KD cases across Europe.

DocuSign Envelope ID: 651725BB-F200-474E-A72C-A62C1F4636C1

1.5 HYPOTHESIS AND AIMS OF KD-CAAP

The overarching goal is to optimise the treatment of KD in children/adolescents across Europe.

KD-CAAP will test the hypothesis that adding immediate adjunctive corticosteroid treatment to IVIG and aspirin will reduce CAA rates in unselected KD patients across Europe compared with IVIG and aspirin alone.

The primary aim of the KD-CAAP trial is therefore to establish:

1. the effectiveness and efficacy of adjunctive corticosteroid therapy combined with IVIG/aspirin for prevention of CAA in unselected patients with KD across Europe;

Secondary aims are to establish:

2. the safety of adjunctive corticosteroid therapy combined with IVIG/aspirin for prevention of CAA in KD;
3. whether adjunctive corticosteroid therapy reduces the duration of fever and length of hospitalisation for patients with KD;
4. the incremental cost-effectiveness ratio for corticosteroid therapy, expressed as the cost per QALY gained, from cost and utility data measured via resource use forms and the Child Health Utility 9D questionnaire.
5. the utility of the Paediatric Glucocorticoid Toxicity (pGTI) tool to assess corticosteroid toxicity.

KD-CAAP will therefore develop an evidence-base that will directly and definitively inform European guidelines for the treatment of KD, which at the moment only target corticosteroids at patients deemed high-risk based on pragmatic, consensus, but non evidence-based clinical features that were formulated as an interim measure pending clinical trials outside of Japan (such as KD-CAAP) [5]. Therefore, beyond the trial, the results will directly influence European clinical guidelines for the treatment of KD, and additionally will likely have international impact beyond Europe as well since data from Russia and the US also suggest poor outcomes with IVIG alone.

DocuSign Envelope ID: 651725BB-F200-474E-A72C-A62C1F4636C1

1.6 BENEFIT-RISK ASSESSMENT

1. IVIG is safe and standard of care. Side effects are generally rare, reversible and mild. Risk of blood product derived infection are minimal with modern screening and processing of IVIG, and since patients receive this for KD anyway, no extra risk from the trial exists. Benefit is prevention of coronary artery aneurysms which would have lifelong consequences, as proven by meta-analyses.
2. Aspirin is safe and standard of care. Side effects are generally rare, reversible and mild. Risk of Gastrointestinal bleeding is minimal in children at dose and duration used for KD, and since patients receive this for KD anyway, no extra risk from the trial exists. Benefit is prevention of coronary artery aneurysms when given with IVIG, which would have lifelong consequences, as proven by meta-analyses.
3. Prednisolone is standard of care for high-risk cases in Europe as highlighted in the European consensus SHARE guideline [5] and many patients receive this for KD, even though there is some degree of equipoise (and hence the need for the trial). Any side effects at the doses and duration used are minimal and a detailed commentary is provided in the Information Sheet. Mitigation of these is use of proton pump inhibitors, and close monitoring for side effects using the Paediatric Glucocorticoid Toxicity Index. Clinicians already use prednisolone for many paediatric indications, and are familiar with their therapeutic index.
4. Despite a number of comparative and non-comparative studies comparing the impact of steroids in KD [54], this potentially highly effective treatment is not commonly used for the treatment of KD. Several factors are likely to contribute – inability to identify high-risk children early on in the disease course, when the meta-analysis suggests benefits will be greatest; lack of clarity on wider benefits in terms of longer-term cardiovascular health in children without overt vasculitis (coronary artery aneurysms, CAA)); relative weakness of the evidence base with Randomised Controlled Trial evidence being relatively small; and concerns about generalisability of findings from Japanese studies on Japanese populations in case ethnic differences contribute to variable efficacy. The proposed trial would delineate the evidence supporting adjunctive corticosteroids (or not), in all patients with KD, leading to a pragmatic and easily implementable recommendation.
5. Epidemiological data suggest worse outcomes in terms of CAA for children under 1 year but there remains significant equipoise about the use of corticosteroids in this age group, therefore inclusion of these patients is still justified in this trial. Randomisation will be stratified however, to ensure an equal balance of children < 1 year in each randomised group.
6. As there is no diagnostic test for KD the diagnosis relies on clinical criteria. The inclusion criteria to the trial allows patients with incomplete cases of KD to be entered in to the trial many patients (particularly infants under the age of 12 months) have some but not all of the clinical features of KD, but may still be at high risk of CAA.
7. There is minimal risk of the overall protocol treatment above routine clinical care because all patients are actively managed with IVIG and aspirin. In addition clear criteria for rescue treatment are built into the trial design for both the control and experimental groups. Trial sites have considerable experience with managing patients with KD which will minimise the risks to the patients and the trial overall. We also established through a survey coordinated by the national c4c hubs and PRINTO that the trial protocol is acceptable with no concerns raised.
8. All children will be closely monitored so that side-effects are identified at the earliest opportunity and appropriate action taken. A detailed risk assessment was conducted by the MRC CTU at UCL prior to starting the trial which will inform the level of monitoring required and the proportion of on-site and central monitoring to ensure safety is being reliably assessed. Safety issues will be explicitly considered by the independent Data Monitoring Committee (DMC) who will review unblinded data regularly during the trial. The DMC will

DocuSign Envelope ID: 651725BB-F200-474E-A72C-A62C1F4636C1

oversee all aspects of safety also taking into account any new data arising from other studies worldwide. The DMC will have a charter clearly setting out their roles and responsibilities. Serious adverse events will be reported to MRC CTU at UCL within 24 hours of becoming aware of the event; this responsibility will lie with site PIs and co-PIs, but may also be delegated to the Trial Physicians. The protocol contains a relatively short duration of corticosteroid treatment (in the experimental arm). We will systematically screen for corticosteroid toxicity using the newly formed Paediatric Glucocorticoid Toxicity Index, a variation of the adult tool we helped develop.

9. As children will be involved (aged 30 days to 15 years inclusive) consent will be obtained from parents or carers and assent from children (dependent the acuity of illness and local requirements). They will be provided information on the purpose and nature of the research, what it involves including the risks and benefits to make an informed decision of their child to be involved. Due to the acute nature of KD consent will be required promptly after KD diagnosis, a short trial introductory leaflet may initially be given to a potential patients parent or carer after consideration and if requested the informed consent form will be given to the parent/carer to provide consent.
10. For laboratory tests and storage samples collected within the trial the child/adolescent will give blood. This may result in unwanted adverse effects. The blood drawn during the trial has been limited and where possible will be taken at the same time as standard clinical monitoring. Local anaesthetic creams or sprays routinely used for blood draws may also be used. Many children will be in hospital for the first 5-7 days when the majority of blood draws will be made and will have a cannulae for medication through which the blood draws will be made.
11. For children/adolescents randomised to receive corticosteroids, there will be a slight oral medication burden in addition to/over standard of care. However, this is will be for a limited time period as once the child/adolescent's fever has resolved and their CRP is equal to or less than 10, after Day 5 the dosing of corticosteroids is tapered. A diary card will be completed by parents to record doses taken by their children to determine their adherence.

<u>COVID specific benefit-risk assessment</u>

The safety of subjects participating in KD-CAAP is of primary importance to the sponsor. The risks of subjects' involvement in KD-CAAP were specifically assessed in the context of the ongoing global COVID-19 pandemic and the applicable precautionary response measures in place at the local or national level. Risks to subjects were assessed against the anticipated benefit of KD-CAAP participation for subjects in accordance with International Council for Harmonisation (ICH) Good Clinical Practice (GCP) E6 (principle 2.2), and risks to quality were also assessed in accordance with ICH GCP E6 (Section 5). Clinical trial management requirements for KD-CAAP were also assessed against the European Medicines Agency guidance on the management of clinical trials during the COVID-19 pandemic (European Medicines Agency 2020).

The Sponsor have established measures to ensure that the conduct of KD-CAAP prioritizes the safety of subjects and the integrity of clinical data. These measures were based on a risk assessment of the impact of COVID-19 on subject safety and on clinical trial conduct. The specific measures established for all subjects and investigative sites participating in KD-CAAP are documented in Trial Risk Assessment maintained in the Trial Master File (TMF).

The Sponsor has established an ongoing risk assessment process to assess the impact of COVID-19 on the conduct of KD-CAAP. If there is a change in the risk assessment for ongoing participation in KD-CAAP, in consultation with investigative sites, KD-CAAP will update measures to ensure that the conduct of KD-CAAP prioritizes the safety of subjects and the validity of clinical data. These changes will be documented in the TMF and investigator's site file.

DocuSign Envelope ID: 651725BB-F200-474E-A72C-A62C1F4636C1

For clarification:

1. KD should be treated as KD irrespective of COVID as a trigger [56].
2. KD is easily distinguishable from COVID-19 infection
3. KD-CAAP excludes patients with cardiogenic shock. Therefore, since cardiogenic shock is a feature of PIMS-TS (Paediatric Multisystem Inflammatory Syndrome), these patients are excluded (and should be considered for recruitment into other PIMS-TS Trials).
4. The treatment of PIMS-TS and KD is essentially the same, with steroid and IVIG forming the mainstay, further reducing risk of any harm to patients if the diagnosis changes after recruitment [56, 57].
5. Biomarker studies plan to examine COVID PCR and serology status and any impact of results of KD-CAAP may need to be analysed. Sites who cannot take part have said so, and have been replaced by other sites who can do this despite COVID, mitigating the risk of failed recruitment.

To mitigate against the delay caused by COVID-19 we have requested a year extension to the funder. The trial team believe this is realistic now that the vaccine programme is being rolled out.

SELECTION OF SITES/CLINICIANS

The trial Sponsor has overall responsibility for site and investigator selection.

KD-CAAP will be conducted at sites across Europe, identified through the c4c network.

2.1 SITE/INVESTIGATOR INCLUSION CRITERIA

To participate in the KD-CAAP trial, investigators and clinical trial sites must fulfil a set of basic criteria that have been agreed by the KD-CAAP Trial Management Group (TMG) and are defined below.

Those sites that meet the criteria will be issued with the KD-CAAP master file documentation for their Site-specific Approval (SSA) and CTU accreditation documents. Sites must complete the KD-CAAP Accreditation Form at the same time as applying for their SSA.

2.2 PI'S QUALIFICATIONS & AGREEMENTS

1. The investigator(s) should be qualified by education, training, and experience to assume responsibility for the proper conduct of the trial at their site and should provide evidence of such qualifications through an up-to-date curriculum vitae and/or other relevant documentation requested by the Sponsor, the Research Ethics Committee (REC) or Institutional Review Board (IRB), and/or the regulatory authority(ies).

2. The investigator should be thoroughly familiar with the appropriate use of the investigational products, as described in this protocol, the product information and in other information sources provided by the Sponsor.

3. The investigator should be aware of, and should comply with, the principles of Good Clinical Practice (GCP) and the applicable regulatory requirements. A record of GCP training should be accessible for all investigators.

4. The investigator/site should permit monitoring and auditing by the Sponsor (or their Delegate), and inspection by the appropriate regulatory authority(ies).

5. The investigator should maintain a delegation log of appropriately-qualified persons to whom the investigator has delegated significant trial-related duties.

6. The investigator should sign an investigator statement, which verifies that the site is willing and able to comply with the requirements of the trial.

2.2.1 ADEQUATE RESOURCES

1. The investigator should be able to demonstrate a potential for recruiting the required number of suitable children/adolescents within the agreed recruitment period (that is, the investigator regularly treats the target population).

2. The investigator should have sufficient time to properly conduct and complete the trial, including conducting follow-up visits, within the agreed trial period.

DocuSign Envelope ID: 651725BB-F200-474E-A72C-A62C1F4636C1

3. The investigator should have available an adequate number of qualified staff and adequate facilities for the foreseen duration of the trial to conduct the trial properly and safely.

4. The investigator should ensure that all persons assisting with the trial are adequately informed about the protocol, the investigational product(s), and their trial-related duties and functions.

5. The investigator is responsible for supervising any individual or party to whom the investigator delegates trial-related duties and functions conducted at the trial site.

6. If the investigator/institution retains the services of any individual or party to perform trial-related duties and functions, the investigator/institution should ensure this individual or party is qualified to perform those trial-related duties and functions and should implement procedures to ensure the integrity of the trial-related duties and functions performed and any data generated.

7. The site should have sufficient data management resources to allow prompt data return to the CTU (refer to the Data Management Plan for timelines).

2.3 SITE ASSESSMENT

Each selected clinical trial site must complete a KD-CAAP Site Initial Assessment Form, KD-CAAP Investigator Site File Assessment Form, KD-CAAP Pharmacy Assessment Form, Investigator Statement, Signature and Delegation of Responsibilities Log containing staff contact details, for more information consult the Manual of Operations (MOP). The Investigator Statement verifies that the site is willing and able to comply with the requirements of the trial. This will be signed by the Principal Investigator at the site. In addition, and in compliance with the principles of GCP, all site staff participating in the trial must complete the Signature and Delegation of Responsibilities Log and forward this to the CTU. The CTU must be notified of any changes to trial personnel and/or their responsibilities. An up-to-date copy of this log must be stored in the Trial Master File (TMF) at the site and also at the CTU.

2.4 APPROVAL AND ACTIVATION

On receipt of the above documents at the CTU, providing formal agreements are in place between, the site and the Sponsor, and the site and the funder, written confirmation will be sent from the CTU to the PI that the trial may start in that site.

1. The site should conduct the trial in compliance with the protocol as agreed by the Sponsor and, if required, by the regulatory authority(ies), and which was given favourable opinion by the REC and/or IRB.
2. The PI or delegate should document and explain any substantive deviation from the approved protocol, and communicate this with the trial team at the CTU (see MOP for further details).

A list of activated sites may be obtained from the Trial Manager.

DocuSign Envelope ID: 651725BB-F200-474E-A72C-A62C1F4636C1

SELECTION OF CHILDREN

There will be **no exceptions** to eligibility requirements at the time of randomisation. Questions about eligibility criteria should be addressed prior to attempting to randomise the child/adolescent.

The eligibility criteria are the standards used to ensure that only medically appropriate children/adolescents are considered for this trial. Children/adolescents not meeting the criteria should not join the trial. For the safety of the children/adolescents, as well as to ensure that the results of this trial can be useful for making treatment decisions regarding other children/adolescents with KD, it is important that no exceptions be made to these criteria for admission to the trial.

Children/adolescents will be considered eligible for enrolment in this trial if they fulfil all the inclusion criteria and none of the exclusion criteria as defined below.

3.1 INCLUSION CRITERIA

1. Aged 30 days (post-natal age) to 15 years inclusive, and below the country-specific age of consent for the duration of the trial
2. KD defined in at least one of the three following ways

(a) as per American Heart Association (AHA) criteria [1]: namely fever for at least 5 days in addition to 4 of the following 5 clinical criteria:
   i. bilateral non purulent conjunctivitis
   ii. cervical lymphadenopathy
   iii. polymorphous skin rash
   iv. changes in lips or mucosa (strawberry tongue, red cracked lips, diffuse erythematous oropharynx)
   v. extremity changes (erythema, oedema of palms and soles in initial phase, and at convalescent stage skin peeling)

(b) OR less than 5 days of fever but all 5 clinical criteria above

(c) OR incomplete KD cases, as per a modified*AHA definition [1], namely:
   i. children/adolescents (>1 year old) with fever greater than or equal to 5 days AND at least 2 other compatible clinical criteria as listed above; OR infants ≤ 1 year old with fever greater than or equal to 7 days without other explanation;

   AND for both age groups
   ii. CRP ≥30 mg/L or erythrocyte sedimentation rate (ESR) ≥40 mm/hr (or both)

   AND for both age groups
   iii. EITHER the presence of any 3 or more of: anaemia for age (haemoglobin < lower limit of normal reference range for local laboratory) platelet count ≥450 $x10^9$/L or <140 $x10^9$/L; albumin <30 g/L; elevated ALT (> upper limit of normal reference range for local laboratory); white cell count ≥15 $x10^9$/L; urine ≥10 white blood cells per high power field
   iv. OR abnormal echocardiogram compatible with KD but without established CAA, with ≥ 3 of the following suggestive features: decreased left ventricular function, mitral regurgitation, pericardial effusion, or dilated but non-aneurysmal coronary arteries (internal diameter 2≤Z<2.5; and not meeting the exclusion criteria for aneurysmal change as defined below).

3. Written informed consent from appropriate legal representative(s), and assent from patients who have not reached the age of consent and will not reach the age of consent for the

DocuSign Envelope ID: 651725BB-F200-474E-A72C-A62C1F4636C1

duration of the trial in the participating country, but are judged to have capacity for this (depending on both age and acuity of illness)

*This definition of incomplete KD is modified from the AHA definition by firstly, the exclusion of aneurysmal coronary artery changes as the sole echo finding, since this is an exclusion criterion for KD-CAAP, and secondly the inclusion of low platelet count as well as high platelet count, as highlighted in recent European consensus SHARE guideline [5].

Note that patients with KD can still be included in KD-CAAP if they have started IVIG treatment, as long as they are randomised no more than 24 hours after the IVIG infusion is initiated (see exclusion criteria below).

Test results must be from tests done on the calendar day of randomisation or the day before.

3.2 EXCLUSION CRITERIA

Disease-related exclusions:

1. This diagnosis is a second or further episode of KD.
2. Already established CAA at screening.
3. Severe Congestive Heart Failure or cardiogenic shock defined as the presence of hypotension and shock requiring the initiation of volume expanders.
4. Known congenital coronary artery abnormality that would impair assessment of the primary endpoint.
5. Suspected macrophage activation syndrome.

Exclusions related to medications:

6. Started IVIG more than 24 hours prior to randomisation.
7. Known hypersensitivity to prednisolone or methylprednisolone, or known phenylketonuria to aspartame used in a formulation in an infant less than 12 weeks.
8. Current oral, intravenous or intramuscular corticosteroid treatment for more than 3 days in previous 7 days prior to randomisation.
9. History of previous severe reaction to any human immune globulin preparation.

Exclusions related to general health or other issues:

10. Active varicella zoster virus or influenza infection; or known exposure to a case of varicella within the previous 21 days prior to randomisation if known to be non-immune.
11. Co-enrolment in another study/trial of an investigative medicinal product.
12. Pregnant or/and breastfeeding adolescents.

Disease-related exclusions relate to those (rare) patients who already have severe fulminant inflammation and/or shock when they are diagnosed with KD, in whom recent European consensus suggests corticosteroids and/or other immunosuppression are required [5]. Such exceptional cases represent a small minority and therefore will not substantial impact on recruitment targets.

A blood or urine pregnancy test must be completed on the day or day before randomisation for adolescents who have begun menstruation.

DocuSign Envelope ID: 651725BB-F200-474E-A72C-A62C1F4636C1

3.3 NUMBER OF CHILDREN/ADOLESCENTS TO RECRUIT

The total number of children/adolescents to be recruited in KD-CAAP will be 262 (131 in each group).

3.4 CO-ENROLMENT GUIDELINES

Co-enrolment in another observational study at the time of randomisation is permitted as long as it will have no impact on treatment delivery, management, or follow-up; that is, as long as it has no impact on adherence to the procedures described in this protocol. Trials of another investigational medicinal product (IMP) are not permitted (see exclusion criteria above). Specific cases should be discussed with the Chief Investigators and CTU before randomisation. Co-enrolment in future trials (i.e. post-randomisation) is considered in **Section 4.2.**

3.5 SCREENING PROCEDURES & PRE-RANDOMISATION INVESTIGATIONS

Potentially eligible children/adolescents will be identified prior to, or within 24 hours after starting IVIG and aspirin, and ideally at the time a diagnosis of KD is established and before treatment is initiated. Parents/carers of potentially eligible children/adolescents may initially be given a very short leaflet describing the trial, also suitable for older children and adolescents, to minimise burden at presentation with acute illness. Parents/carers who are potentially interested in their child/adolescent participating will then be provided with a more detailed information sheet about the KD-CAAP trial and asked to give written consent before any trial-specific procedures, including randomisation, are performed or any blood is taken for the trial. Older children and adolescents will also be given a more detailed but still simplified information sheet, and asked to give written assent before any trial-specific procedures (age threshold for assent depending on regulations in each individual country). The information sheets and consent forms will include the option to be contacted in future for long-term follow-up, but this is not required to join the trial.

Signed consent forms (by either one or both parents/carers according to national regulations) and signed assent forms (as required) must be kept by the investigator and documented in the case record form (CRF) and a copy/copies given to the child/adolescent and/or family.

It will be made completely and unambiguously clear that the child/adolescent and/or parent or legal guardian of the child/adolescent is free to refuse to participate in all or any aspect of the trial, at any time and for any reason, without incurring any penalty or affecting the child/adolescent's treatment. See **Section 5.7** for more details on procedures around child/adolescent withdrawal from the trial.

Children/adolescents should be randomised as soon as possible and within 24 hours after starting IVIG, meeting eligibility criteria, and written informed consent being obtained.

3.6 SAMPLES TO BE TAKEN AS SOON AS CONSENT IS OBTAINED

A blood sample should be collected as soon as possible after consent (and assent as appropriate) in order to assay CRP, haemoglobin, MCV, white cell count, lymphocytes, neutrophils, platelets, ESR, urea, creatinine, AST, ALT, bilirubin, sodium, potassium, albumin, calcium, phosphate, glucose, ALP and LDH. If tests have already been done on the calendar day of randomisation or the day before randomisation they do not need to be repeated. Urinalysis for glycosuria, proteinuria and haematuria, either on urine dipstick or by formal laboratory analysis, should also be obtained; additionally, urine

DocuSign Envelope ID: 651725BB-F200-474E-A72C-A62C1F4636C1

microscopy for white cell count is mandated at screening only for incomplete cases if this is one of the features required to satisfy inclusion. Blood should also be drawn for storage (maximum total 5.5 ml: 0.5-1 ml EDTA for plasma, 0.5-1 ml serum, 0.5-1 ml EDTA for DNA, and 2.0-2.5 ml RNA Paxgene for RNA extraction) and a throat swab, and processed following the Sample Collection Manual.

ENROLMENT & RANDOMISATION

Randomisation (stratified, see below) will be performed at the CTU using a computer algorithm concealed from the investigators/trial management staff, and accessed by either CTU staff or delegated site staff online. Patients who fulfil the eligibility criteria and whose parent/carer (legal representative) have provided informed consent will be randomised 1:1 to receive adjunctive corticosteroids or not, plus standard of care IVIG and aspirin.

Epidemiological data suggest worse outcomes in terms of CAA for very young patients (age <1 years) and male children/adolescents [1, 5, 7], who will therefore form two key subgroup analyses. There remains significant equipoise about the use of corticosteroids in this young age group, however [58], and therefore it is essential to include these patients in KD-CAAP. In order to balance the groups for these two factors (age <1 versus ≥1 year, sex), randomisation will be stratified for these two factors using minimisation (with a built in random element), as well as for recruiting country.

Date of birth will be collected from participants at randomisation and held in a secure database which has restricted access to trial team members and certain site staff. It is critical that appropriate children are identified prior to enrolment into the trial. For the study collection of date of birth is a risk reduction strategy is to ensure all participant are eligible at the point of entering the study and importantly will be eligible for the duration (3months) of their participation in the clinical trial in different EU countries. Date of birth is crucial in the diagnosis of Kawasaki Disease for participants who do not meet the complete KD criteria which also forms part of ensuring the participant is eligible for the study. Due to the worse outcomes for very young participants mentioned above the participants date of birth is required to allow the stratification of participants along with sex as well as recruiting country.

Children/adolescents will be recruited from the paediatric emergency departments and wards of the site hospitals, and will be identified by the research team at the site. Children/adolescents will be randomised prior to, or within 24 hours of starting IVIG and aspirin. Before randomisation, the participant's eligibility for enrolment will be confirmed. Parents/guardians must confirm that they have read the relevant patient information sheets and have provided written informed consent to enter into the trial.

Enrolment assessments will be performed as summarised in the Trial Assessment Schedule (**Table 1)**, including recording of weight, height, vital signs and clinical characteristics relevant to this episode from the medical notes. Results of any blood tests (haematology, biochemistry) and urine tests or echocardiography, ECG done as part of standard clinical care will also be recorded; blood tests and urine tests are required at trial enrolment or on the previous calendar day. Echocardiography and ECG are not required for enrolment.

Blood should be taken for storage as per the schedule of assessments in **Table 1**.

The clinician should complete the Baseline Form and send for data entry directly onto the secure web-based trial database.

Clinical assessments should be scheduled for follow up visits for days (D) 1, 2, 3, 4 and 5 and weeks 1, 2, 6, 12 as per **Table 1**. Children will generally be inpatients for around one week after randomisation, so most of these visits will occur during hospitalisation.

DocuSign Envelope ID: 651725BB-F200-474E-A72C-A62C1F4636C1

A trial register will be kept at the clinical site and will record all children/adolescents who are eligible and invited to join the trial. Those accepting will have names, date of admission, age (months or years), randomisation date and trial number recorded. Those who refuse will have name, date of admission, age (months or years), and reason for refusal recorded. The register will be kept in a secure place in each clinical site; will be available for monitoring, audit and inspection; and will be the responsibility of the Principal Investigator at that site.

4.1 RANDOMISATION PRACTICALITIES

Further details on the generation of randomisation lists can be found in **Section 9.1**.

Randomisation will be performed online using minimisation. To randomise a child/adolescent the information contained on a completed randomisation CRF will be entered into the online trial database, accessible from the local clinical sites and CTU, which will automatically check for eligibility. Only children/adolescents with a completed and verified screening and randomisation CRF on the database will be able to be randomised. Allocation will be made after eligibility has been confirmed through the online database, and will be concealed until the point of randomisation when only the randomisation for the current child/adolescent will be provided. Delegated member(s) of staff at each site will be responsible for carrying out the randomisation process restricted using role-based access. The details of the child's treatment allocation will be notified to clinical staff, and the allocation cross checked between those randomising and those managing the child/adolescent clinically.

If the CTU are to process the randomisation the screening and randomisation CRF should be securely sent via electronic media to staff at the CTU. At the CTU, staff will verify eligibility and perform the randomisation using the online system. The details of the child's treatment allocation will be notified to the trial team at the site by email or phone within one hour of the receipt of the randomisation form (during UK normal working hours).

The child's date of randomisation will be entered into the Trial Register at the site.

The trial is open-label so there is no unblinding.

4.2 CO-ENROLMENT GUIDELINES AND REPORTING

After randomisation, enrolment in any other clinical study of an investigational medicinal product is not allowed for the duration of the follow up period i.e. 12 weeks after randomisation. Post-randomisation enrolment in observational studies is acceptable in accordance with local guidelines, and providing it does not impact the child/adolescent's ability to comply with the KD-CAAP protocol. This should be discussed with the Chief investigator before enrolment.

DocuSign Envelope ID: 651725BB-F200-474E-A72C-A62C1F4636C1

TRIAL TREATMENTS

The investigational medicinal products within the trial are:

- IVIG (Human normal immunoglobulin)
- Aspirin
- Prednisolone or IV methylprednisolone

All children/adolescents will commence treatment with IVIG (2g/kg which should be given as per local standard of care) and aspirin (40mg/kg/day) as per standard of care. Children/adolescents will be randomised no more than 24 hours after IVIG was initiated to two groups in a 1:1 ratio:

- Control group: no additional treatment and
- Experimental group: adjunctive open-label oral prednisolone (2 mg/kg/day) or IV methylprednisolone equivalent (total 1.6mg/kg/day) if oral prednisolone is not tolerated (for example, due to inability to take oral medication).

Further management will be dictated by the temperature (body temperature) and C-reactive protein (CRP) responses (**Figure 1;** and more details provided below).

Treatment will be open-label without blinding and will be dispensed at the point of starting corticosteroids, with adjustments to the prescription performed by local investigators as required by the protocol. KD-CAAP is an open-label trial and uses medicinal products with marketing authorisation. As such the trial treatments (IMPs):

(i) Will be sourced via usual hospital procurement arrangements

(ii) Will be stored in accordance with local hospital practice, in compliance with manufacturer's instructions. There are no trial specific temperature monitoring requirements.

Body weight for IMP dosing should be obtained at Day 0, using standard methods (as to determine the doses of corticosteroids, aspirin and IVIG). Body weight reported by parents is not acceptable.

5.1 INTRAVENOUS IMMUNOGLOBULIN AND ASPIRIN (BOTH RANDOMISED GROUPS)

5.1.1 PRODUCTS & TREATMENT SCHEDULE

All children/adolescents will initiate treatment with IVIG (2 g/kg should be given as per local standard of care) and aspirin (40 mg/kg/day) as per standard of care. The local pharmacy stock of IVIG and aspirin will be used at each site.

The particular formulation for IVIG will be that normally used at the local site, i.e. a particular preparation is not specified. The only specification is that this must be a preparation manufactured to appropriate medicinal product standards in Europe. This should be infused as per local standard of care, with intra-infusion monitoring as per standard of care for IVIG infusion at the local institution.

Aspirin will be administered orally, initially at 40 mg/kg/day in 4 divided doses until the child/adolescent is afebrile for at least 48 hours; reducing to 3-5 mg/kg/day in one dose until at least 21 days after the resolution of the fever. Again, no particular preparation is specified; the site will use whichever preparation they normally use as per their routine clinical care. The oral formulation (tablet versus dispersible) will be determined by the age of the patient (usually dispersible in very young patients).

Flexibility in dose of +/-20% from the weight based dose is allowed for IVIG and aspirin.

5.1.2 STOPPING DRUG EARLY DUE TO REACTION TO IVIG AND ASPIRIN

Overall, intravenous immunoglobulin is a safe therapy, and side effects are usually mild and self-limiting. Reported side-effects include: flulike symptoms; infusion-related dermatological reactions; arrhythmias and transient hypotension in patients with a history of cardiac disease (but rarely reported in KD); thrombotic events; aseptic meningitis; transient renal impairment; and very rarely haemolysis [59]. In the situation when a reaction to IVIG is suspected (e.g. typical, indicative skin rash) the relevant safety reporting procedures should be followed.

Aspirin has been used in the treatment of KD for many years, and overall is a safe medicine. Reported side-effects include gastrointestinal symptoms (dyspepsia); bleeding; cutaneous reactions; headache; hearing impairment; hepatic injury; asthma attack; renal impairment; and bone marrow dyscrasia. Reye syndrome is a risk in children who receive aspirin while they are experiencing active infection with varicella or influenza [1].

Adverse events caused by drug toxicity leading to a treatment change are expected to be rare. In case treatment is stopped, however, children/adolescents should remain in the trial for follow-up, and should continue to follow the assessment schedule. Further treatment can de decided based on local investigator discretion.

5.1.3 COMPLIANCE & ADHERENCE

IVIG will be administered by ward nurses and recorded on CRFs by trial staff. Therefore non-adherence to IVIG will be minimal. During admission, aspirin will also be administered by ward nurses and recorded on CRFs by trial staff. After discharge, we will document as accurately as possible what aspirin the patient actually takes and the reasons for any reported non-compliance, using patient medication diaries.

5.2 CONTROL GROUP: FURTHER MANAGEMENT BASED ON ASSESSMENT OF FEVER AND CRP RESPONSE ON DAY 2 AND ON DAY 5

Patients in the control group will be further assessed on follow-up Day 2 (±12h). A second dose of IVIG (2g/kg) can be given at this assessment if patient has CRP>50% of baseline and still >10 mg/L, or temperature (T) is ≥38 °C. Table 2 below summarises all the possible case scenarios based on CRP and temperature responses, and the treatment plan for each scenario.

Table 2: Management of patients in control group on day 2

TEMPERATURE	CRP	TREATMENT PLAN
<38 °C	≤10 mg/L	No further treatment required [reassess on day 5]; reduce aspirin to 3-5 mg/kg/day when afebrile for at least 48 hours and continue for at least 21 days after resolution of fever*
<38 °C	>10 mg/L but ≤50% of baseline	No further treatment required [reassess on day 5]; reduce aspirin to 3-5 mg/kg/day when afebrile for at least 48 hours and continue for at least 21 days after resolution of fever*

TEMPERATURE	CRP	TREATMENT PLAN
<38 °C	>10 mg/L and still >50% of baseline	Second dose of IVIG; reduce aspirin to 3-5 mg/kg/day when afebrile for at least 48 hours and continue for at least 21 days after resolution of fever*
≥ 38 °C	≤10 mg/L	Second dose of IVIG; continue with aspirin at 40 mg/kg/day until afebrile
≥ 38 °C	>10 mg/L but ≤50% of baseline	Second dose of IVIG; continue with aspirin at 40 mg/kg/day until afebrile
≥ 38 °C	>10 mg/L and still >50% of baseline	Second dose of IVIG; continue with aspirin at 40 mg/kg/day until afebrile

*following local standard of care

At day 5 (±12h) further management is again dictated by temperature and CRP, as per below:

- If CRP≤10 mg/L and T<38° C, no further additional treatment is required. Aspirin should be continued as per above.
- If CRP>10 mg/L or T≥38° C rescue treatment should be considered at discretion of local investigator. See Section 5.4 for recommended (but non-mandatory) rescue treatment options.

Table 3 below summarises the possible scenarios for management of patients in the control group based on assessment of temperature and CRP at Day 5.

Table 3: Management of patients in control group at Day 5

TEMPERATURE	CRP	TREATMENT PLAN
<38 °C	≤10 mg/L	Continue aspirin at 3-5 mg/kg/day for at least 21 days after resolution of fever*; no further treatment required
< 38 °C	>10 mg/L	Consider rescue treatment at discretion of local investigator; continue with aspirin at 3-5 mg/kg/day for at least 21 days after resolution of fever*
≥ 38 °C	≤10 mg/L	Consider rescue treatment at discretion of local investigator; continue with aspirin at 40 mg/kg/day until afebrile
≥ 38 °C	>10 mg/L	Consider rescue treatment at discretion of local investigator continue with aspirin at 40 mg/kg/day until afebrile

*following local standard of care

5.3 EXPERIMENTAL GROUP TREATMENT

5.3.1 PRODUCTS & TREATMENT SCHEDULE

The local pharmacy stock of prednisolone/methylprednisolone will be dispensed for trial treatment as the trial IMP. The following licensed preparation of corticosteroids may be used:

- Methylprednisolone sodium succinate powder and solvent for solution of injection.
- Prednisolone tablets
- Prednisolone soluble tablets
- Prednisolone solution
- Prednisone tablets

DocuSign Envelope ID: 651725BB-F200-474E-A72C-A62C1F4636C1

These preparations are considered bioequivalent with appropriate dose adjustment (i.e. 1 mg prednisolone/prednisone equates to 0.8 mg of IV methylprednisolone) (https://bnf.nice.org.uk/treatment-summary/glucocorticoid-therapy.html). Prednisolone is a medicine with high solubility and high permeability (Biopharmaceutics Classification System - BCS class I). This means two immediate-released products (e.g. soluble tablet versus normal tablets) are likely to be bioequivalent (assuming similar excipients). A bioequivalence study was not considered necessary to support the licensing of prednisolone oral solution. It was considered bioequivalent to soluble tablet and to another prednisolone solution [60, 61].

The experimental group will receive oral prednisolone at a dose of 2 mg/kg/day as soon as possible following randomisation. Maximum daily dose of oral prednisolone in first week (and subsequently) is 80 mg. If oral prednisolone is not tolerated then intravenous methylprednisolone may be given at equivalent doses (1.6 mg/kg/day i.e 0.8 mg/kg IV every 12 hours). Corticosteroid tapering is allowed from day 5 onwards provided there is resolution of fever (temperature <38 °C) and CRP ≤10 mg/L, and should be completed over 15 days in 5-day steps from 2 to 1 to 0.5 mg/kg/day, then to 0 mg.

Oral prednisone may be substituted for prednisolone at the same dose at the discretion of the local investigator following usual practice. Single daily dose soluble forms are acceptable in children.

Doses should be rounded to the nearest mg (easily achievable using soluble forms) that allows whole tablets to be administered (in accordance with dose ranges permitted). Flexibility in dose of +/-12.5% (but not exceeding 80 mg daily) from the mg/kg dose above is allowed.

Proton pump inhibitor should be considered e.g. lansoprazole 15-30 mg/day (or alternative proton pump inhibitor) until prednisolone dose is ≤10 mg/day (or 0.15 mg/kg/day), but are not mandated by the trial. Standard paediatric lansoprazole dosing will apply: for example children/adolescents <30 kg 0.5-1 mg/kg (max dose of 15 mg) once daily; for children/adolescents ≥30 kg 30 mg once daily.

5.3.2 Dispensing and Accountability of IMP

Children/adolescents will only be discharged on the tapering dose of corticosteroids (**Table 5**); carers should be provided with a sufficient supply to complete the total duration of oral corticosteroids or to reach the next KD-CAAP follow-up visit on discharge from hospital. Carers will be requested to return any unused drug to the clinic.

On no account should any drug assigned to a child/adolescent be used by anyone else. Unused trial drug must be returned to the site if a child/adolescent withdraws from treatment before completing therapy.

There are no trial specific accountability arrangements (over and above what is required locally at each hospital) required for IVIG, aspirin and prednisolone, methylprednisolone or prednisone used from locally supplied stock in KD-CAAP unless specifically required as per a country's individual regulatory requirements.

Further details can be found in the appropriate KD-CAAP Pharmacy MOP. Local working instructions for IMP storage and dispensing will be reviewed at site initiation.

DocuSign Envelope ID: 651725BB-F200-474E-A72C-A62C1F4636C1

5.3.3 Experimental group: further management based on assessment of fever and CRP response on Day 2 and day 5

As for the control group, a second dose of IVIG (2g/kg) can be given on day 2 (±12h) if CRP>50% of baseline and CRP > 10 mg/L, or if temperature ≥38 °C.

Table 4 below provides all possible case scenarios for management of the experimental group on day 2 IVIG.

Table 4: Management of patients in the experimental group on day 2 follow-up visit

Temperature	CRP	Treatment plan
<38 °C	≤10 mg/L	Continue with prednisolone at 2 mg/kg/day no additional treatment required [reassess on day 5]; reduce aspirin to 3-5 mg/kg/day when afebrile for at least 48 hours and continue for at least 21 days after resolution of fever*
<38 °C	>10 mg/L but ≤50% of baseline	Continue with prednisolone at 2 mg/kg/day no additional treatment required [reassess on day 5]; reduce aspirin to 3-5 mg/kg/day when afebrile for at least 48 hours and continue for at least 21 days after resolution of fever*
<38 °C	>10 mg/L and still >50% of baseline	Continue with prednisolone at 2 mg/kg/day and administer second dose of IVIG; reduce aspirin to 3-5 mg/kg/day when afebrile for at least 48 hours and continue for at least 21 days after resolution of fever*
≥ 38 °C	≤10 mg/L	Continue with prednisolone at 2 mg/kg and administer second dose of IVIG; continue with aspirin at 40 mg/kg/day until afebrile
≥ 38 °C	≤10 mg/L but ≤50% of baseline	Continue with prednisolone at 2 mg/kg/day and administer second dose of IVIG; continue with aspirin at 40 mg/kg/day until afebrile
≥ 38 °C	≤10 mg/L and still >50% of baseline	Continue with prednisolone at 2 mg/kg/day and administer second dose of IVIG; continue with aspirin at 40 mg/kg/day until afebrile

*following local standard of care

At day 5 (±12h) further management is dictated by temperature and CRP:

- If CRP ≤10 mg/L and T<38 °C, taper corticosteroids as described below. Aspirin should be continued as per above.
- If CRP > 10 mg/L or T≥38 °C rescue treatment should be considered. See Section 5.4 for suggested (non-mandatory) rescue treatments.

DocuSign Envelope ID: 651725BB-F200-474E-A72C-A62C1F4636C1

Table 5 below provides all possible case scenarios for management of patients in experimental group at Day 5.

Table 5: Management of patients in the experimental group at Day 5

TEMPERATURE	CRP	TREATMENT PLAN
<38 °C	≤10 mg/L	Corticosteroid taper: oral prednisolone 1 mg/kg/day for 5 days, then 0·5 mg/kg/day for another 5 days, then stop; continue aspirin at 3-5 mg/kg/day for at least 21 days after resolution of fever*
<38 °C	> 10 mg/L	Continue oral prednisolone 2 mg/kg/day until afebrile AND CRP ≤10 mg/L (then taper as above) and consider rescue treatment at discretion of local investigator; continue aspirin at 3-5 mg/kg/day for at least 21 days after resolution of fever*
≥38 °C	≤10 mg/L	Continue oral prednisolone 2 mg/kg/day until afebrile AND CRP ≤10 mg/L (then taper as above) and consider rescue treatment at discretion of local investigator; continue with aspirin at 40 mg/kg/day until afebrile
≥38 °C	>10 mg/L	Continue oral prednisolone 2 mg/kg/day until afebrile AND CRP ≤10 mg/L (then taper as above) and consider rescue treatment at discretion of local investigator; continue with aspirin at 40 mg/kg/day until afebrile

*following local standard of care

5.3.4 STOPPING DRUG EARLY

An important goal of KD-CAAP is to document corticosteroid toxicity as well as effectiveness and efficacy. Although corticosteroids have been used for decades for the treatment of inflammatory diseases of the young, hitherto there has been no systematic method or tool to collate corticosteroid-related toxicity. We and others have recently developed such a tool for use in adults, the glucocorticoid toxicity index [62]. We have now been involved in the development of a similar tool for use in paediatric trials: the paediatric Glucocorticoid Toxicity Index (pGTI) [2].

The pGTI consists of a Composite Index and a Specific List. The Composite pGTI reflects glucocorticoid toxicity that has the potential to change during a clinical trial: to worsen if glucocorticoid doses increase, or to improve if successful glucocorticoid sparing is achieved. Toxicities included in the Composite pGTI are expected to occur commonly and to vary with glucocorticoid exposure. They are therefore weighted and an aggregate score calculated. In contrast, the Specific List is designed to capture glucocorticoid toxicity not included in the Composite pGTI; these are often clinical events that are not reversible on lower corticosteroid exposure, or are uncommon (typically affecting <5%).

The Composite Index of the pGTI consists of ten domains of glucocorticoid toxicity: body mass index, growth, glucose tolerance, lipid metabolism, systolic blood pressure, bone mineral density, glucocorticoid-induced myopathy, skin toxicity, neuropsychiatric impact, and infections. The Specific List includes six additional unique domains that address other features of glucocorticoid toxicity such as pubertal delay, sex hormone access interruption, ocular toxicity (cataracts, central serous retinopathy), and bone health (osteonecrosis).

Adverse events caused by corticosteroid toxicity leading to a treatment change are expected to be rare. In the situation where this occurs, it is usually not the case that these require to be stopped, but if the adverse event is particularly severe, treatment may be discontinued at the discretion of the local

DocuSign Envelope ID: 651725BB-F200-474E-A72C-A62C1F4636C1

investigator. Children/adolescents should remain in the trial for follow-up and should continue to follow the assessment schedule. Further treatment can be considered based on local investigator discretion. Lastly, if corticosteroids are stopped early, this should be tapered over several days (in accordance to local practice i.e. not protocolised) to prevent adrenocortical insufficiency, in line with routine clinical care. The risk of gastrointestinal ulceration and bleeding may be increased when acetylsalicylic acid and corticosteroids are co-administered. This risk can be mitigated by the use of non mandatory of proton pump inhibitors as per local practice.

5.3.5 COMPLIANCE AND ADHERENCE

Corticosteroids will be initiated whilst the child is in hospital. In hospital they will be administered by ward nurses and recorded on CRFs by trial staff. Therefore non-adherence will be minimal. Intravenous methylprednisolone will only be given in hospital whilst the child is too unwell to tolerate oral medication. After discharge, we will document as accurately as possible what trial oral medication the patient actually takes, and the reasons for any reported noncompliance (including spitting out or refusing doses) using patient medication diaries for corticosteroids (as for aspirin). Corticosteroids are widely used worldwide for the treatment of a wide range of inflammatory diseases. The importance of adherence will be reinforced at the time trial medication is dispensed and during any subsequent contacts with the trial team. Formal assessment of corticosteroid related toxicity, and how this may affect adherence, will also be assessed by using the pGTI [2] (see also Section 6.7.1).

5.4 RECOMMENDED NON-MANDATORY RESCUE THERAPIES FOR BOTH EXPERIMENTAL AND CONTROL GROUP

The trial schema (Figure 1) and Sections 5.2 and 5.3.3 above detail the management that children/adolescents in the trial should receive, including consideration of rescue treatment at day 5 based on fever and CRP responses. However, the local physician may add rescue treatment at any time if this is considered in the best interests of the child/adolescent; wherever possible, this should be discussed with the Chief Investigators first, and reasons for this will be recorded on CRFs. Rescue treatments will be chosen by the local investigator in line with their sites' preferred standard of care. These non-mandatory rescue treatments may include:

(i) Re-treatment with IVIG (2 g/kg)
(ii) Corticosteroids: options (at the discretion of the local investigator) may include: IV methylprednisolone at 10-30 mg/kg/day for 3 days followed by course of oral prednisolone at 2 mg/kg/day until there is resolution of fever and CRP≤ 10 mg/L; starting oral prednisolone (2 mg/kg/day) for 5 days if not previously received; continuation of oral prednisolone at 2 mg/kg/day for experimental group beyond Day 5 until fever resolved and CRP≤ 10 mg/L
(iii) Infliximab 6 mg/kg/dose up to maximum of 2 doses 2 weeks apart
(iv) Ciclosporin at 5 mg/kg per day
(v) IL-1 blockade therapy (e.g. anakinra 2-4 mg/kg/day subcutaneously for 2 weeks, or longer depending on the therapeutic response)
(vi) Other therapies can also be considered at the discretion of the site investigator.

All children/adolescents receiving rescue treatments should continue to be followed up in the trial to the last 12 week follow-up visit, "on-study, off-study-treatment", regardless of reason for initiating rescue treatment. Any concomitant rescue therapy medications will be documented in the CRF.

DocuSign Envelope ID: 651725BB-F200-474E-A72C-A62C1F4636C1

5.5 HANDLING CASES OF TRIAL MEDICATION OVERDOSE

Parents/guardians of the children/adolescents participating in the trial should be counselled about the importance of taking the oral medications as prescribed. Parents/guardians should contact the KD-CAAP research team immediately if their child/adolescent has been overdosed, to receive appropriate advice. Children/adolescents will then be managed on a case by case basis. Instances of overdose with IMP which results in clinical symptoms of any grade will be deemed as a deviation from trial procedure and a notifiable event and will be reported as such.

5.6 UNBLINDING / UNMASKING

KD-CAAP is an open label trial so unblinding/unmasking of treatment will not be necessary.

5.7 PROTOCOL TREATMENT DISCONTINUATION

In consenting to the trial, parents/guardians are consenting to trial treatment, trial follow-up and data collection for their child. However, an individual child/adolescent may stop randomised trial treatment early, or be stopped early for any of the following reasons:

- Inadequate response or relapse on trial treatment
- Unacceptable toxicity or adverse event
- Intercurrent illness that prevents further treatment
- Any change in the child's condition that justifies the discontinuation of trial treatment in the clinician's opinion
- Use of a medication that is essential for the child's management with a known major or moderate drug interaction with the trial treatments
- Inadequate compliance with the protocol treatment in the judgement of the treating physician, including for reasons of tolerability
- Overdose with trial drug
- Withdrawal of consent for treatment by the child

As the child/adolescent's participation in the trial is entirely voluntary, they may choose to discontinue the trial treatment at any time without penalty or loss of benefits to which they are otherwise entitled. Although the child/adolescent is not required to give a reason for discontinuing their trial treatment, a reasonable effort should be made to establish this reason while fully respecting the child/adolescent's and family's rights.

Children/adolescents stopping randomised trial treatment should remain in the trial for the purpose of follow-up and data analysis "on-study, off-study-treatment" (unless the child/adolescent explicitly withdraws their consent from all stages of the trial). Any other non-trial medication a trial child/adolescent receives should be recorded. If a child/adolescent is withdrawn from all follow-up, refer to **Section 6.9**.

Data will be kept and included for children/adolescents who stop follow-up early.

5.8 TREATMENT DATA COLLECTION

Information about all treatments for Kawasaki Disease including formulation, frequency, dose and reasons for change will be collected on the CRF. Start and stop dates of other concomitant medications will also be collected.

5.9 NON-TRIAL TREATMENT

5.9.1 MEDICATIONS PERMITTED

All necessary concomitant medications are allowed except for those listed in **Section 5.10**. Regular medications will be recorded at enrolment. Parents will be asked to report the use of additional medications during follow-up visits. If a medication with a known major or moderate drug interaction with prednisolone or IVIG or aspirin is essential for a child's management and cannot be replaced by a drug that does not have an interaction with these therapies, then the trial medication should be stopped and the concomitant medication used.

Since the main differential diagnosis for KD is infection, in line with routine clinical care, we anticipate that many children enrolled will be treated concomitantly with antibiotics (either oral or intravenous). Similarly, since KD is associated with fever, we anticipate that most, if not all, children will receive paracetamol (orally, as rectal suppository, or even intravenously if preferred by the standard of care at the recruiting site). Nonsteroidal anti-inflammatory drugs (NSAIDs) for the relief of pyrexia are contraindicated, however as discussed below. Antibiotics, paracetamol or any other medicines will be documented in the CRF.

In view of the age of participants eligible for inclusion in the trial (up to 15 years only), that pregnancy/breastfeeding is an exclusion criteria, the relatively short duration of follow-up (12 weeks), the fact that participants will be recruited when they are acutely unwell and hospitalised and will likely remain in hospital for at least a week and only be discharged on low-dose aspirin, and that corticosteroids and low-dose aspirin are commonly used in pregnancy (see Section 7.2.1), the use of contraception is not expected in the trial.

5.10 MEDICATIONS NOT PERMITTED

Use of NSAIDs (e.g. ibuprofen, naproxen, indomethacin, and mefenamic acid, amongst others) except aspirin is not allowed, because they abrogate the anti-platelet effect of low dose aspirin therefore should be avoided following guidelines [1, 5].

Immunisations should follow current recommendations regarding immunisations post IVIG. Immunisation with all live vaccines should generally be deferred for at least 6 months following an episode of KD treated with IVIG, mainly due to the potential lack of effectiveness following IVIG [5]. Thereafter, all vaccines should be administered as recommended by national schedules [5]. Currently available SARS-CoV-2 vaccines are not live and therefore guidance is as per any non-live vaccines.

DocuSign Envelope ID: 651725BB-F200-474E-A72C-A62C1F4636C1

5.11 TREATMENT AFTER TRIAL EVENT

Treatment will be at the discretion of the responsible physician. However, all children should continue to be followed up “off-study-medication, on-study” until 12 weeks’ post randomisation, and all medication received documented on CRFs.

5.12 CO-ENROLMENT GUIDELINES

Co-enrolment in previous or future trials is considered in **Section 4.2**.

ASSESSMENTS & FOLLOW-UP

6.1 TRIAL ASSESSMENT SCHEDULE

The frequency of follow-up visits and assessments are detailed in the Trial Assessment Schedule (**Table 1)**.

Trial visit and contact schedules will be prepared for each child/adolescent at randomisation, and children/adolescents should be followed on that same schedule, until the final follow-up, even if their trial medication is discontinued prematurely (see **Section 5.7**). The target dates for trial contacts are determined by the date of randomisation and are not affected by subsequent events. The schedule defines visit dates (with windows) necessary for data collection.

Trial assessments will be performed on D0, D1, D2, D3, D4 and D5 post-randomisation and at weeks 1, 2, 6. Thereafter the final trial assessment will be carried out at week 12 (last follow-up visit for each individual patient) after randomisation. Parents should be told to contact trial staff if there is any sign of symptom recurrence, and additional visits will be performed at these times. A window of 12 hours either side of each trial visit will be permissible for visits up to D5, up to 1 day before and 3 days after for the week 1 assessment, up to 3 days either side of the week 2 assessment and up to 14 days either side for the week 6 and week 12 assessments. However, if a child/adolescent attends late, all information required at the missed visit should still be collected, even if they attend outside the window. Further, given the age of the participants and the acuity of the illness, visits that do not happen within these windows will not be considered as protocol deviations. Sites may choose to re-schedule contacts to allow for public holidays or other unavoidable circumstances that affect the scheduled visit date, but the re-scheduled visit or contact should preferably be in the window period above.

Most of these trial assessments coincide with standard clinical assessments, thus no extra visits are required for the trial out-with routine clinical care, with the exception of the 12 week visit. These assessments are as follows:

(i) At each visit, history and physical examination (with emphasis on clinical features of KD), vital signs (heart rate, blood pressure), and temperature/documentation of fever (T ≥38°C), concomitant medication, adverse events, blood tests (see below) and resource utilisation. Height (or length in young children) will be assessed at D0, W1, W2, W6, and W12 and weight will be assessed at D0, W6 and W12. For children still febrile and in hospital on D5 maximum daily temperatures will be collected until discharge or afebrile for 2 calendar days whilst still hospitalised.
(ii) Urine dipstick for glycosuria, proteinuria, haematuria at baseline D0, D2, D5, week 1, 2, 6 and 12 (formal laboratory analysis may also be used). Microscopy of urine for white cell count is not mandated post screening.
(iii) Electrocardiogram (ECG) and echocardiography at weeks 1, 2, 6 and 12 (see **Section 6.5** on detailed echocardiography assessment).
(iv) Paediatric glucocorticoid toxicity index (pGTI) at weeks 1 and 12 (see **Section 6.7.1**).
(v) Health economics assessments: resource utilisation at every visit, as above, and Health related quality of life (HRQL) at D0 and at least week 1 or 2 (depending on date of discharge) and weeks 6 and 12 (see **Section 6.8.1**).
(vi) Quality of life (QoL) will be assessed at D0 and week 12 (see **Section 6.8.2**).
(vii) Blood measurements (full blood count, ESR, CRP, biochemistry, liver function tests, glucose) will be performed as in **Table 1** and results recorded in CRFs.

(viii) Blood will also be taken for storage for additional scientific studies as in **Table 1.**

Every attempt should be made to keep the blood draw (including any losses in the manoeuvre) for research samples within the 3% of the total blood volume recommended for children/adolescents during a period of 6 weeks for all children weighing over 8.7kg and will not exceed 1% at any single time for all children weighing over 6.8kg (since the total volume of blood is estimated at 80 to 90 ml/kg body weight, 3% equates to 2.4ml blood per kg body weight [4]. For any enrolled children lighter than 6.8kg, the clinician should endeavour that the total blood volume does not exceed 1% at any single time' Since KD is an acute, severe illness, bloods required for routine clinical may occasionally surpass these limits as is often the case when managing critically ill children in routine clinical care.. Most children will remain in hospital for the first 5-7 days in the trial and will have cannulae for medication through which blood draws can be made.

6.2 BASELINE INFORMATION COLLECTED AT ENROLMENT

At randomisation, medical and relevant social history, including demographics and socioeconomics, documentation of any underlying diseases, duration of symptoms to date will be recorded. Anthropometry will include weight, height (or length in young children). Vital signs (heart rate, blood pressure) and temperature will be measured. Relevant symptoms and signs will be solicited. All children/adolescents will have routine blood tests and urinalysis (see **Table 1**) prior to starting treatment with IVIG, or as soon as possible after initiation of IVIG. HRQL and QoL will be assessed using standard questionnaires. Blood will be collected for storage for research substudies.

6.3 INFORMATION TO BE COLLECTED AT ALL FOLLOW UP ASSESSMENTS

A physical examination must be performed at each face-to-face assessment, including acute events if the child/adolescent returns to clinic. Telephone visits are acceptable if face-to face visits are not possible (due to circumstances related to COVID-19).

The following will be recorded as possible whether it is a face-to-face or telephone visit:

- Vital signs (heart rate, blood pressure) and temperature, additionally height (or length in young children) will be measured on D0, Week 1, 2, 6 and 12 and weight at D0, W6 and W12. For children still febrile and in hospital on D5 maximum daily temperatures will be collected until discharge or afebrile for 2 calendar days whilst still hospitalised.
- Symptoms and clinical signs, specific solicited side-effects and adverse events
- Concomitant care/healthcare utilisation
- Results of any haematology, biochemistry and any other investigations undertaken as per **Table 1**
- Any additional blood/or imaging tests as part of the usual standard of care, but not required by the trial
- Adherence with aspirin for the control and experimental group assessed using standardised diaries.
- Adherence with corticosteroids for the experimental group assessed using standardised diaries.

DocuSign Envelope ID: 651725BB-F200-474E-A72C-A62C1F4636C1

6.4 INFORMATION TO BE COLLECTED AT WEEKS 1, 2, 6 AND 12

In addition, at these time points the following will be recorded:

- Urinalysis for glycosuria, proteinuria, haematuria (assessed using standard bedside dip test) will be undertaken on week 1, 2, 6 and 12 (also D2 and D5).
- Electrocardiogram (ECG) and echocardiography will be performed at weeks 1, 2, 6 and week 12 (see **Section 6.5**).
- HRQL will be assessed at least week 1 or 2 (depending on date of discharge) and weeks 6 and 12 (see **Section 6.8.1**).
- Quality of life will be assessed at week 12 (see **Section 6.8.2**).
- pGTI at weeks 1 and 12 [2] (see **Section 6.7.1**).
- Blood or urine Pregnancy test for adolescents who have begun menstruation at week 12

6.4.1 ACUTE EVENTS

Parents/guardians will be given a card with the contact details for the trial research team at their site, and will be encouraged to return to the site if the child/adolescent becomes acutely unwell during the follow-up period. During any acute events, the child/adolescent can be seen face-to-face if attending the randomising site. Otherwise, telephone contact can be arranged.

6.5 ECHOCARDIOGRAPHY AND ECG

Two-dimensional echocardiograms will be digitally recorded at recruiting sites and interpreted at a core laboratory by at least one of two paediatric echocardiographers who will be blinded to randomised group. Echocardiography will be undertaken at weeks 1, 2, 6 and 12; any results of echocardiography done before enrolment or at any unscheduled timepoints will also be reviewed centrally. Echocardiography should include the following parameters:

- Assessment of cardiac function – normal/global dysfunction/regional dysfunction;
- Ejection fraction (biplane Simpson method);
- Left Ventricular End Diastolic Diameter (LVEDD) and Left Ventricular End Systolic Diameter (LVESD) from M-mode;
- Assessment of mitral valve regurgitation – absent/mild/moderate/severe;
- Transmitral inflow characteristics including the peak early filling (E wave) and late diastolic filling (A wave) velocities and the E/A ratio;
- Pulsed wave tissue Doppler Imaging (TDI) sampling from the septal and lateral mitral annulus including the early diastolic relaxation velocity (e') and the systolic myocardial velocity (s');
- Measurement of peak tricuspid regurgitation velocity;
- Measurement of diastolic left ventricular eccentricity index;
- Presence of pericardial effusion and depth in parasternal long axis plane;

In addition, coronary artery assessment and still frames of each coronary artery dimension with measurements should be obtained. Detailed measurements of internal diameters of the left main coronary artery (LMCA), left anterior descending (LAD) and right coronary artery (RCA) should be performed according to methodology described in Lopez et al [63]. Height and weight must be measured accurately since these affect the body surface area for the z-scores.

Z-scores for internal coronary artery diameter will be documented based on normative data: www.parameterz.com/refs/lopez-circimaging-2017 [64]. For the primary endpoint, CAA will be defined as luminal diameter >3.0 mm in a child <5 yrs; or >4.0 mm in a child/adolescent ≥ 5 yrs; or

DocuSign Envelope ID: 651725BB-F200-474E-A72C-A62C1F4636C1

internal diameter of a segment at least 1.5 times that of an adjacent segment or when a luminal contour is clearly irregular; or a luminal internal diameter z-score of ≥2.5 [1, 5]. CAA defined by a luminal internal diameter z-score of ≥2.5 will be also considered as a standalone secondary endpoint [1, 5] The type of coronary artery abnormality (saccular aneurysm, fusiform aneurysm, coronary ectasia) and the presence of thrombi (occlusive, non-occlusive) should also be recorded [1, 5], as should the presence of pericardial effusion and valve regurgitation.

Standard 12 lead ECG should be obtained at weeks 1, 2, 6 and 12, using standard equipment in clinical use at sites. There are no specific calibration requirements because this is not a formal endpoint, but an additional safety measure.

6.6 PROCEDURES FOR ASSESSING EFFICACY

The primary measures of effectiveness and efficacy in KD-CAAP are based on CAA and coronary Z-scores which will be assessed by echocardiograms at follow-up visits, and the review of these using at least one of two blinded independent assessors. Other efficacy measures include length-of-stay, changes in temperature and blood test results, and use of additional treatments, which will be determined from regular in-hospital follow-up and the medical notes, and recorded on CRFs.

6.7 PROCEDURES FOR ASSESSING SAFETY

The clinical examination at each scheduled visit will explicitly prompt for symptoms relating to possible drug toxicities. Additional safety blood tests or investigations may be performed to investigate symptoms or monitor emergent laboratory test abnormalities as clinically indicated. Parents/carers will be encouraged to return to the trial site if their child becomes acutely unwell during trial follow-up, to support timely identification of adverse events.

Adverse events (clinical and laboratory) will be graded using the 2017 Division of AIDS (DAIDS) toxicity grading scale v2.1 (see **Appendix I**). All grade 3 or 4 adverse events will be reported on CRFs, as will adverse events of any grade that lead to modification of IVIG, aspirin or corticosteroids, clinical adverse events judged definitely/probably/possibly related to IVIG, aspirin or corticosteroids, and any SAEs. Other adverse events will be recorded in the clinical notes but will not be reported on CRFs – the reason is that these grade 1 and 2 adverse events will be very common as the children/adolescents will be sick when admitted. They will therefore not be informative as they commonly reflect the underlying disease process rather than any impact of the medication. SAEs will be defined according to ICH GCP, and should be reported to the CTU within 24 hours of the site being aware (see **Section 0**). All adverse events meeting these definitions should be reported on trial CRFs, regardless of their relationship to KD.

6.7.1 PAEDIATRIC GLUCOCORTICOID TOXICITY (PGTI)

Pre-specified clinical complications relating to corticosteroid use will be assessed using the aforementioned pGTI, which is a comprehensive glucocorticoid toxicity assessment instrument [2]. This tool will assess glucocorticoid related morbidity scored in different domains relating to body mass index, arterial hypertension, lipids, skin, neuropsychiatric symptoms, glucose tolerance and infection risk, amongst others (data will be collected for this tool on CRFs). The tool will be scored at weeks 1 and 12.

DocuSign Envelope ID: 651725BB-F200-474E-A72C-A62C1F4636C1

6.8 OTHER ASSESSMENTS

6.8.1 HEALTH ECONOMICS

Incremental costs and cost-effectiveness are included as secondary endpoints to estimate whether the use of adjunctive corticosteroids is a cost-effective intervention to prevent CAA in KD. The health economic analysis will adopt the perspective of healthcare providers in each country. The health care costs for each patient will be estimated by collecting the use of healthcare resources e.g. treatments, investigations, hospital admissions, and contacts with health professionals on CRFs. Country-specific unit costs to value this resource use will be obtained from published and administrative sources. Outcomes in the economic analysis will be measured in terms of the co-primary outcomes of the trial, and in terms of health-related quality of life (HRQL) and quality-adjusted life years (QALYs). Health utilities suitable for measuring HRQL and QALYs will be assessed at Day 0 and at least week 1 or 2 (depending on date of discharge) and weeks 6 and 12 using the Child Health Utility 9D (CHU9D) questionnaire to the parents/carers of all the participants and to the participants themselves if aged 8 years and over (**Table 6**). The CHU9D has been validated for use in children [65] and is available in multiple languages. EQ-5D-Y (youth version) will additionally be administered to children/adolescents aged 8 years and over, and is available in multiple languages. The tools to be used according to the patients' age are summarised in **Table 6**.

Table 6: Health economics questionnaires

AGE	COMPLETED BY PARTICIPANT	COMPLETED BY PARENT / GUARDIAN (PROXY)
<1 to <8 (from 30 days to 7 years inclusive))	None administered	CHU9D (proxy)
8 to <16 (from age 8 to 15 years inclusive)	CHU9D<br>EQ-5D-Y (including EQ-VAS -visual analogue scale)*	CHU9D (proxy)

* EQ-5D-Y is recommended for 8-11 as well as 12-16 year olds (p4, https://euroqol.org/docs/EQ-5D-Y-User-Guide.pdf)

6.8.2 QUALITY OF LIFE MEASURES

Quality of life (PedsQL™ score) is another endpoint that will be assessed within KD-CAAP [66, 67]. Relevant country specific translations for these tools are available. It will be scored at Day 0 and week 12.

The PedsQL™ is a 23 item generic QoL questionnaire that has a child self report for ages 5 through 18 years and a parent proxy report for children ages 2 through 18 years. The questionnaire takes 5 to 10 minutes to complete [66, 67]. The questionnaire yields information on the physical, emotional, social and school functioning of the child during the previous 4 weeks. It has been extensively tested in both healthy children and children with chronic disease [66, 67]. Mean scores are calculated based on a 5-point response scale for each item and transformed to a 0 to 100 scale with a higher score representing better quality of life [66, 67]. The PedsQL™ yields 3 summary scores: a total scale score, a physical health summary score, and a psychosocial health summary score [66, 67]. There are 4 scale scores: physical functioning, emotional functioning, social functioning, and school functioning [66, 67]. The total score is comprised of the average of all items in the questionnaire. The psychosocial summary score is comprised of the average of the items in the emotional, social, and school functioning scales [66, 67]. The physical health summary score is comprised of the average of items in the physical functioning scale and is the same score as the physical functioning score [66, 67].

DocuSign Envelope ID: 651725BB-F200-474E-A72C-A62C1F4636C1

6.9 EARLY STOPPING OF FOLLOW-UP

A parent/guardian who chooses to discontinue trial treatment for their child/adolescent should be encouraged to follow the trial procedures and follow-up schedule. However, if they do not wish to remain on trial follow-up, their decision must be respected and the child/adolescent will be withdrawn from the trial. The CTU should be informed of this in writing using the appropriate documentation. Prior to transferring to routine follow-up, the parent/guardian will be asked to have assessments performed as appropriate for a final trial visit. They would be at liberty to refuse any or all individual components of the follow-up assessments.

If follow-up is stopped early, the medical data collected during their participation in the trial will be kept and used in the analysis for the KD-CAAP trial, as consent cannot be withdrawn for data already collected. Similarly, samples and data obtained prior to this time will be processed according to the protocol for further research, unless the parent/guardian explicitly and unprompted requests otherwise. Consent for future use of stored samples already collected can be refused if follow-up is stopped early (but this should follow a discussion).

Given the short follow-up period (12 weeks), children/adolescents who have left the trial may not re-consent to participation in the trial subsequently.

Children/adolescents who stop trial follow-up early will not be replaced, since the sample size calculation already incorporates an inflation factor to account for lost-to-follow-up.

6.10 LOSS TO FOLLOW-UP

Follow-up is only for 12 weeks, and therefore we anticipate a low rate of lost to follow-up. For operational management at participating sites, a child/adolescent will be classified as “lost to follow-up” (meaning no further attempts at contact are made) only when three unsuccessful attempts have been made to contact the parent/guardian.

6.11 COMPLETION OF PROTOCOL FOLLOW UP

Each child/adolescent will complete follow-up in the trial at their 12 week visit.

DocuSign Envelope ID: 651725BB-F200-474E-A72C-A62C1F4636C1

SAFETY REPORTING

The principles of GCP require that both investigators and Sponsors follow specific procedures when notifying and reporting adverse events or reactions in clinical trials. These procedures are described in this section of the protocol. Section 7.1 lists definitions, Section 7.3 gives details of the investigator responsibilities and Section 7.4 provides information on CTU responsibilities.

7.1 DEFINITIONS

The definitions of the EU Directive 2001/20/EC Article 2 based on the principles of GCP apply to this trial protocol. These definitions are given in Table 7.

Table 7: Adverse events definitions

TERM	DEFINITION
Adverse Event (AE)	Any untoward medical occurrence in a patient or clinical trial subject to whom a medicinal product has been administered, including occurrences that are not necessarily caused by or related to that product.
Adverse Reaction (AR)	Any untoward and unintended response to an investigational medicinal product related to any dose administered.
Unexpected Adverse Reaction (UAR)	An adverse reaction, the nature or severity of which is not consistent with the information about the medicinal product in question, as set out in the Summary of Product Characteristics (SPC) or Investigator Brochure (IB) for that product.
Serious Adverse Event (SAE) or Serious Adverse Reaction (SAR) or Suspected Unexpected Serious Adverse Reaction (SUSAR)	Any adverse event, adverse reaction or unexpected adverse reaction that:<br>Results in death<br>Is life-threatening*<br>Requires hospitalisation or prolongation of existing hospitalisation**<br>Results in persistent or significant disability or incapacity<br>Consists of a congenital anomaly or birth defect<br>Is another important medical condition***

*The term life-threatening in the definition of a serious event refers to an event in which the patient is at risk of death at the time of the event; it does not refer to an event that hypothetically might cause death if it were more severe, for example, a silent myocardial infarction.

**Hospitalisation is defined as an inpatient admission, regardless of length of stay, even if the hospitalisation is a precautionary measure for continued observation.

*** Medical judgement should be exercised in deciding whether an AE or AR is serious in other situations. The following should also be considered serious: important AEs or ARs that are not immediately life-threatening or do not result in death or hospitalisation but may jeopardise the subject or may require intervention to prevent one of the other outcomes listed in the definition above; for example, a secondary malignancy, an allergic bronchospasm requiring intensive emergency treatment, seizures or blood dyscrasias that do not result in hospitalisation or development of drug dependency.

DocuSign Envelope ID: 651725BB-F200-474E-A72C-A62C1F4636C1

7.1.1 MEDICINAL PRODUCTS

An investigational medicinal product is defined as the tested investigational medicinal product (IMP) and the comparators used in the trial (EU guidance ENTR/CT 3, April 2006 revision). For KD-CAAP this includes prednisolone, methylprednisolone, aspirin and IVIG.

Adverse reactions include any untoward or unintended response to drugs. Reactions to an IMP or comparator should be reported appropriately.

7.1.2 ADVERSE EVENTS

Adverse Events include:

- An exacerbation of a pre-existing illness
- An increase in frequency or intensity of a pre-existing episodic event or condition
- A condition (even though it may have been present prior to the start of the trial) detected after trial drug administration
- Continuous persistent disease or a symptom present at baseline that worsens following administration of the trial treatment

Adverse Events do not include:

- Medical or surgical procedures; the condition that leads to the procedure is the adverse event
- Pre-existing disease or a condition present before treatment that does not worsen
- Hospitalisations where no untoward or unintended response has occurred, e.g. elective cosmetic surgery
- Overdose of medication without signs or symptoms

7.2 OTHER NOTABLE EVENTS

Overdose of IMP which results in clinical symptoms of any grade and pregnancy are notifiable events.

7.2.1 PREGNANCY

Corticosteroids are commonly used in pregnancy (for example, for the treatment of recurrent miscarriage or fetal abnormalities such as congenital adrenal hyperplasia), and their benefits are judged to outweigh any risks [68, 69]. Similarly IVIG and low-dose aspirin (to reduce pre-eclampsia) are used in pregnancy [70, 71]. Given the age range that will be recruited and short duration of follow-up a pregnancy test for adolescents menstruating will be carried out at week 12. Adolescents who become pregnant within their trial participation will be followed up to pregnancy outcome. Administration of the trial IMP if pregnancy is identified should be managed by the local investigator taking into account the risks and benefits to both the participant, given the serious and acute nature of Kawasaki disease, and to the unborn child.

7.3 INVESTIGATOR RESPONSIBILITIES

All AEs, should be recorded in the patient's medical notes. All grade 3 or 4 adverse events should be reported on the relevant CRFs, as should be adverse events of any grade that lead to modification of IVIG, aspirin or corticosteroids, clinical adverse events judged definitely/probably/possibly related to IVIG, aspirin or corticosteroids, and any SAEs. SAEs should be notified to the CTU within 24 hours of the investigator becoming aware of the event.

DocuSign Envelope ID: 651725BB-F200-474E-A72C-A62C1F4636C1

7.3.1 INVESTIGATOR ASSESSMENT

7.3.1.A Seriousness

When an AE or AR occurs, the investigator responsible for the care of the child/adolescent must first assess whether or not the event is serious using the definition given in **Table 7**. If the event is serious, then an SAE Form must be completed and the CTU notified within 24 hours.

7.3.1.B Severity or Grading of Adverse Events

The severity of all AEs and/or ARs (serious and non-serious) in this trial should be graded using the toxicity grading in **Appendix I**.

7.3.1.C Causality

The investigator must assess the causality of all serious events or reactions in relation to the trial IMP using the definitions in **Table 8,** regardless of when during follow-up the event occurred. There are five categories: unrelated, unlikely, possible, probable, and definitely related to receipt of the trial drug. If the causality assessment is unrelated or unlikely to be related, the event is classified as an unrelated SAE. If the causality is assessed as possible, probable or definitely related, the event is classified as an SAR.

Table 8: Assigning Type of SAE Through Causality

RELATIONSHIP	DESCRIPTION	SAE TYPE
Definitely	There is clear evidence to suggest a causal relationship and other possible contributing factors can be ruled out.	SAR
Probable	There is evidence to suggest a causal relationship and the influence of other factors is unlikely.	SAR
Possible	There is some evidence to suggest a causal relationship (for example, because the event occurs within a reasonable time after administration of the trial medication). However, the influence of other factors may have contributed to the event (for example, the child's clinical condition, other concomitant treatments).	SAR
Unlikely	There is little evidence to suggest that there is a causal relationship (for example, the event did not occur within a reasonable time after administration of the trial medication). There is another reasonable explanation for the event (for example, the child's clinical condition, other concomitant treatment).	Unrelated SAE
Unrelated	There is no evidence of any causal relationship	Unrelated SAE

7.3.1.D Notification

The CTU should be notified of all SAEs within 24 hours of the investigator becoming aware of the event.

Investigators should notify the CTU of all SAEs occurring from the time of signature of the informed consent form until the child/adolescent exits the trial. Any subsequent events that may be attributed to treatment should be reported to the national regulators using the relevant system.

DocuSign Envelope ID: 651725BB-F200-474E-A72C-A62C1F4636C1

7.3.2 Notification Procedure

1. The SAE Form must be completed by an investigator (a physician named on the Signature List and Delegation of Responsibilities Log, who is responsible for the child/adolescent's care; this will be either the Principal Investigator or another medically qualified person with delegated authority for SAE reporting). Due care should be paid to the grading and causality of the event, as outlined above. In the absence of the responsible investigator, the form should be completed and signed by a member of the site trial team and emailed as appropriate. The responsible investigator should subsequently check the SAE Form, make changes as appropriate, sign and then re-send to the CTU as soon as possible. The initial report must be followed by detailed, written reports as appropriate.

The minimum criteria required for reporting an SAE are the trial number, name of investigator reporting the event, and why it is considered serious.

2. The SAE Form must be sent by email to mrcctu.kdcaap@ucl.ac.uk within 24 hours of the site trial team being made aware of the event.

3. Follow-up: children/adolescents must be followed up until clinical recovery is complete and laboratory results have returned to normal or baseline, or until the event has stabilised. Follow-up should continue after completion of protocol treatment if necessary. A further SAE Form, indicated as 'Follow-up' should be completed and emailed to the CTU as information becomes available. Extra, annotated information and/or copies of test results may be provided separately. The child/adolescent must be identified by trial number and their random 3-letter code only. The child's name should not be used on any correspondence and should be deleted from any test results.

4. Staff should follow their institution's procedure for local notification requirements.

SERIOUS ADVERSE EVENT (SAE) REPORTING
Within 24 hours of becoming aware of an SAE, please email a completed SAE form to the MRC CTU at UCL on mrcctu.kdcaap@ucl.ac.uk

7.4 MRC CTU RESPONSIBILITIES

Medically-qualified staff at the CTU and/or the Chief Investigator (or a medically-qualified delegate) will review all SAE reports received. The causality assessment given by the local investigator at the hospital cannot be overruled; in the case of disagreement, both opinions will be provided in any subsequent reports.

If the IMP's causal relationship to the Serious Adverse Event has been assessed as possible, probable, or definitely the CTU has the responsibility to determine the expectedness of the event to the trial IMP. An unexpected adverse reaction is one that is not listed within the trial Reference Safety Information or one that is more frequent or more severe than previously reported. If a SAR to the trial IMP is assessed as being unexpected, it becomes a SUSAR. Section 4.8 of a representative SPC will be considered as the Reference Safety Information. The trial Safety Management Plan will define the choice of SPC.

DocuSign Envelope ID: 651725BB-F200-474E-A72C-A62C1F4636C1

The CTU is undertaking the duties of the trial Sponsor and is responsible for the reporting of SUSARs and other SARs to the regulatory authorities (MHRA and competent authorities of other European member states and any other countries in which the trial is taking place) and the research ethics committees, as appropriate. Fatal and life-threatening SUSARs must be reported to the competent authorities within 7 days of the CTU becoming aware of the event; other SUSARs must be reported within 15 days. This responsibility may be delegated to one representative in each country for relevant reporting requirements in individual countries.

The CTU will also keep all investigators informed of any safety issues that arise during the course of the trial.

The CTU, as Sponsor, will submit once a year an Annual Safety Reports in the form of a Developmental Safety Update Report (DSUR) to Competent Authorities (Regulatory Authority and Ethics Committee). The DSUR will include:

- a line list of all suspected (unexpected or expected) serious adverse reactions, along with an cumulative summary table of all reported serious adverse events, ordered by body system
- a report concerning the safety of the subjects, consisting of a complete safety analysis and an evaluation of the balance between the benefit and risk of the IMPs under investigation.

DocuSign Envelope ID: 651725BB-F200-474E-A72C-A62C1F4636C1

QUALITY ASSURANCE & CONTROL

8.1 RISK ASSESSMENT

The Quality Assurance (QA) and Quality Control (QC) considerations have been based on a formal Risk Assessment, which acknowledges the risks associated with the conduct of the trial and how to address them with QA and QC processes. QA includes all the planned and systematic actions established to ensure the trial is performed and data generated, documented and/or recorded and reported in compliance with the principles of GCP and applicable regulatory requirements. QC includes the operational techniques and activities done within the QA system to verify that the requirements for quality of the trial-related activities are fulfilled. This Risk Assessment has been reviewed by the CTU's Research Governance Committee (RGC) and has led to the development of a Data Management Plan (DMP), Safety Management Plan and Monitoring Plan which will be separately reviewed by the Quality Management Advisory Group (QMAG).

8.2 CENTRAL MONITORING AT CTU

KD-CAAP will use an online database. Either sites will be responsible for their own data entry directly onto the online trial database at the site or sites can scan paper CRFs and email them to the CTU for data entry. In both cases, the site will retain the original CRF. Data stored on the central database will be checked at CTU for missing or unusual values (range checks) and checked for consistency within children/adolescents over time. If any problems relating to data quality are identified, the site will be contacted and asked to verify or correct the entry. Changes will be made on the original CRF and entered into the database at the site. CTU will also send reminders for any overdue and/or missing data with the regular inconsistency reports of errors.

Other essential trial issues, events and outputs will be detailed in the Monitoring Plan that is based on the trial-specific Risk Assessment.

8.3 ON-SITE MONITORING

The frequency, type and intensity for monitoring and the requirements for triggered monitoring will be detailed in the Monitoring Plan. This plan will also detail the procedures for review and sign-off.

A detailed site initiation meeting with training will be performed at each trial site by staff from the CTU either face to face or online. The site initiation meeting will include training in the administration and side effects of trial drugs, as well as the trial procedures.

8.3.1 Direct Access to Children's Records

Participating investigators and their institutions should agree to allow trial-related monitoring, including audits, ethics committee review and regulatory inspections by providing direct access to source data and documents as required, including to electronic health records. Parental/caregiver consent and children's assent (where required) must be obtained for this. Such information will be treated as strictly confidential and will in no circumstances be made publicly available.

The trial data and consent/assent should all be verifiable from source documents which may include paper notes and electronic health records.

Not all such information will be monitored; rather the monitoring plan will describe a risk-based approach to monitoring based on ongoing random samples of patient clinical and laboratory data, which may be increased if issues are identified.

The investigator/institution should maintain adequate and accurate source documents and trial records that include all pertinent observations on each of the site's trial subjects. Source data should be attributable, legible, contemporaneous, original, accurate, and complete. Changes to source data should be traceable, should not obscure the original entry, and should be explained if necessary (e.g., via an audit trail).

For this trial, the CRFs that will be the source document for the data elements listed in the following table. These data elements will be recorded directly on the CRFs and therefore the CRFs will be regarded as source data:

**CRF name and number:**	**Data item (Question text)**
Form 04 Follow Up	In the initial admission of Kawasaki Disease, has the participant been in the Intensive Care Unit (ICU)?<br>Total number of nights in an ICU related to Kawasaki Disease<br>Total number of nights in an ICU for other reasons<br>In the initial admission of Kawasaki Disease, has the participant been in the High Dependency Unit (HDU)?<br>Total number of nights in a HDU related to Kawasaki Disease<br>Total number of nights in a HDU for other reasons<br>In the initial admission of Kawasaki Disease, did any family member, friend or companion take any time off paid work (or business activity if self-employed) to help care for the participant, either due to their illness or in order for them to see any healthcare professional?<br>Number of days of lost earnings related to Kawasaki Disease<br>Number of days of lost earnings for other reasons<br>Number of days of lost annual leave time/holiday time related to Kawasaki Disease<br>Number of days of lost annual leave time/holiday time for other reasons<br>Please enter the code for the highest level of education for each parent/carer in the household in which the child/adolescent spends most of their time:<br>Parent/Carer 1, L7b. If 'Other', please specify:<br>Parent/Carer 2, L8b. If 'Other', please specify:<br>Parent/Carer 3, L9b. If 'Other', please specify:
Form 12a Paediatric Glucocorticoid Toxicity Assessment—Specific list	If post-pubertal, maintenance of the same Tanner stage for more than one year<br>Delayed start of puberty<br>New-onset secondary amenorrhea or oligomenorrhea since the start of glucocorticoid therapy<br>Diabetic nephropathy<br>Diabetic neuropathy<br>Diabetic retinopathy<br>Symptomatic adrenal insufficiency<br>Hypertensive emergency<br>Posterior reversible encephalopathy syndrome<br>Osteonecrosis of one site

DocuSign Envelope ID: 651725BB-F200-474E-A72C-A62C1F4636C1

CRF name and number:	Data item (Question text)
	Osteonecrosis of more than one site<br>Bone mineral density decrease ≥ 1 standard deviation (z-score) if scan performed<br>Insufficiency fracture<br>Insufficiency fracture in more than one bone<br>Major glucocorticoid myopathy<br>Tendon rupture<br>More than one tendon rupture<br>Major skin toxicity<br>Sleep disturbance: Severe sleep disturbance with latency >60min, >4 night awakenings, total sleep <8 hours (3-5yrs) <7 hrs (6+ yrs)<br>Mood regulation: Persistent irritability, depression with loss of activity, suicidal, or elevated mood, irrational ambitions<br>Cognitive impairment: Substantial learning difficulties leading to impaired educational progress<br>Psychosis: Clear psychotic features, with persistent hallucinations, thought disorder, hypomania (grandiosity)<br>Glucocorticoid-induced violence toward self or others<br>Grade 5 infection (death from infection)<br>Central serous retinopathy<br>New-onset or worsened elevation of intra-ocular pressure requiring treatment or change in treatment<br>Posterior subscapular cataracts (or history of same)<br>Gastrointestinal perforation (occurring in the absence of regular nonsteroidal anti-inflammatory drug use)<br>Peptic ulcer disease confirmed by endoscopy (excluding H. pylori) or severe dyspeptic symptoms despite treatment
Form 12b Paediatric Glucocorticoid Toxicity Assessment—Composite list	Has there been any change in diabetes medications?<br>Has there been a change in blood pressure medications?<br>Has there been a change in lipid medications?<br>Does the patient have a glucocorticoid myopathy?<br>Acneiform rash<br>Easy bruising<br>Hirsutism<br>Atrophy / Striae<br>Sleep problems<br>Mood regulation<br>Cognitive impairment<br>Psychotic features<br>Infection
Form 13 Health Resource Use	Since the last scheduled visit (or since discharge if this is the first post-discharge visit), has the participant been seen by a local family doctor or other healthcare professional outside of this visit?<br>Number of visits related to Kawasaki Disease to a local family doctor<br>Number of visits for other reasons to a local family doctor<br>Number of visits related to Kawasaki Disease to a local family doctor out-of-hours surgery<br>Number of visits for other reasons to a local family doctor out-of-hours surgery

DocuSign Envelope ID: 651725BB-F200-474E-A72C-A62C1F4636C1

CRF name and number:	Data item (Question text)
	Number of visits related to Kawasaki Disease by a local family doctor home visit (call-out)<br>Number of visits for other reasons by a local family doctor home visit (call-out)<br>Number of visits related to Kawasaki Disease to another healthcare professional outside of hospital, please specify<br>Number of visits for other reasons to another healthcare professional outside of hospital, please specify<br>Since the last scheduled visit (or since discharge if this is the first post-discharge visit), has the participant attended any of the hospital departments listed below?<br>Number of visits related to Kawasaki Disease to an Accident and Emergency/Emergency Room<br>Number of visits for other reasons to an Accident and Emergency/Emergency Room<br>Number of visits related to Kawasaki Disease to an out-patient clinic (hospital) - excluding visit today<br>Number of visits for other reasons to an out-patient clinic (hospital) - excluding visit today<br>Number of visits related to Kawasaki Disease as a Day case admission<br>Number of visits for other reasons as a Day case admission<br>Since the last scheduled visit (or since discharge if this is the first post-discharge visit), has the participant been admitted to any hospital as an in-patient?<br>Number of nights spent related to Kawasaki Disease on a general ward<br>Number of nights spent for other reasons on a general ward<br>Number of nights spent related to Kawasaki Disease in an Intensive Care Unit (ICU)<br>Number of nights spent for other reasons in an Intensive Care Unit (ICU)<br>Number of nights spent related to Kawasaki Disease in a High Dependency Unit (HDU)<br>Number of nights spent for other reasons in a High Dependency Unit (HDU)<br>If the child/adolescent is in school, college or work, did they take any time off either due to illness or in order to see any health professional, since the last scheduled visit (or since discharge if this is the first post-discharge visit)<br>Number of days off school/college/work related to Kawasaki Disease<br>Number of days off school/college/work for other reasons<br>Since the last scheduled visit (or since discharge if this is the first post-discharge visit), did any family member, friend or companion take any time off paid work (or business activity if self-employed) to help care for the participant, either due to the participant's illness or in order for them to see any healthcare professional?<br>Number of days of lost earnings to Kawasaki Disease<br>Number of days of lost earnings for other reasons<br>Number of days of lost annual leave time/holiday time to Kawasaki Disease<br>Number of days of lost annual leave time/holiday time for other reasons

DocuSign Envelope ID: 651725BB-F200-474E-A72C-A62C1F4636C1

8.3.2 CONFIDENTIALITY

The trial will be conducted in compliance with General Data Protection Regulation.

Children/adolescents will be assigned a trial identification number and this will be used on CRFs; children/adolescents will not be identified by name. The investigator will keep securely a patient trial register showing trial numbers, name, date of admission and age at admission (in months or years). The unique trial number will identify all laboratory specimens, case record forms, and other records and no names will be used, in order to maintain confidentiality. All records will be kept in locked locations. Clinical information will not be released without written permission, except as necessary for monitoring.

DocuSign Envelope ID: 651725BB-F200-474E-A72C-A62C1F4636C1

STATISTICAL CONSIDERATIONS

9.1 METHOD OF RANDOMISATION

Randomisation will be stratified according to country, age (<1 versus ≥1 years) and sex. Epidemiological data suggest worse outcomes in terms of CAA for very young patients (age <1 years) and male children/adolescents [1, 5], who will therefore form two key subgroup analyses. There remains significant equipoise about the use of corticosteroids in this young age group, however [58], and therefore it is essential to include these patients in KD-CAAP. Country was chosen as it has the potential to modify treatment effects (i.e. lead to interaction) due to variation in other clinical management across countries, meaning forcing balance across the randomised groups is most important for this factor. Randomisation will be done using minimisation with a random element. See **Section 4.1** for details of randomisation practicalities.

9.2 OUTCOME MEASURES

9.2.1 PRIMARY OUTCOME MEASURE

KD-CAAP will have two co-primary outcome measures:

- Any CAA (definition below) documented within the 12 weeks of trial follow-up (to assess overall effectiveness of the strategy of immediate corticosteroids in preventing CAA, expecting that some patients will receive rescue treatment before reaching this endpoint in both randomised groups).
- An average estimate across weeks 1, 2, and 6 of the maximum of the Z-score of the internal diameters of the proximal right coronary artery or left anterior descending coronary artery, adjusting for rescue treatment (to assess the direct efficacy of corticosteroids).

CAA is defined as any of

- luminal diameter >3.0 mm in a child <5 years
- luminal diameter >4.0 mm in a child/adolescent ≥5 years
- internal diameter of a segment at least 1.5 times that of an adjacent segment or when a luminal contour is clearly irregular
- luminal internal diameter Z-score of ≥2.5 [1, 5]

Z-scores for internal coronary artery diameter will be documented based on normative data: www.parameterz.com/refs/lopez-circimaging-2017 [64].

CAA has been chosen as the primary endpoint because it is the most meaningful from the clinical viewpoint in terms of future risk of poor outcomes, and accords with previous trials in KD. CAA is defined as meeting the criteria at any time point – that is, it is a binary endpoint of the child ever experiencing this severe outcome. The intention-to-treat analysis of this endpoint therefore assesses the effectiveness of corticosteroids, i.e. is a real-world comparison of the intention to start corticosteroids as soon as possible vs not to start them immediately. To protect children, we have to allow children who are not doing well on their randomised therapy to switch to alternatives – it would not be ethical to propose maintaining children on randomised allocation until the point of developing CAA, which is what would be needed to consider the direct efficacy of corticosteroids vs no corticosteroids. This effectiveness comparison is arguably most relevant to clinicians, on the reasonable assumption that the kind of changes that occur to treatment during the trial would be similar to what would occur outside of the trial.

DocuSign Envelope ID: 651725BB-F200-474E-A72C-A62C1F4636C1

However, in order to estimate the direct efficacy of corticosteroids, we are proposing to conduct a novel inverse probability of (change from) treatment weighting co-primary analysis of the continuous outcome coronary diameter Z-score – we will have greatest power to identify differences between corticosteroids vs no corticosteroids (efficacy) with this continuous endpoint. The multiple values will be dealt with using standard repeated measures methods (most commonly generalised estimating equations with the weighting proposed to address efficacy).

9.2.2 SECONDARY OUTCOME MEASURES

The following secondary outcome measures will be assessed:
Efficacy

- At each of weeks 1, 2, 6 and 12 individually, the maximum of the Z-score of the internal diameters of the proximal right coronary artery or left anterior descending coronary artery.
- Any CAA defined using a stricter definition of a luminal internal diameter Z-score of ≥2.5 alone documented within the 12 weeks of trial follow-up
- Receipt of rescue treatment.
- Receipt of second dose of IVIG.
- Duration of fever after enrolment (time to temperature <38°C).
- Daily serum concentrations of CRP from days 1-5, and at 1 and 2 weeks after enrolment, and time to normalisation of CRP (≤10mg/L).
- Duration of hospitalisation.

Safety

- Serious adverse events including deaths.
- Grade 3 or 4 adverse events.
- Clinical adverse events of any grade judged related to IVIG, aspirin or corticosteroids given to treat KD.

9.2.3 OTHER OUTCOME MEASURES

Other outcome measures that will be assessed are:

- Changes in other laboratory parameters of inflammation (haemoglobin, white cell count, platelet count, ESR, albumin).
- Duration of corticosteroid therapy.
- Cumulative weight adjusted dose of prednisolone or methylprednisolone received.
- Proportion of patients who need to continue prednisolone at 2 mg/kg/day beyond day 5 (experimental group).
- Paediatric appropriate quality of life scores.
- Paediatric corticosteroid toxicity index (pGTI) to assess glucocorticoid related morbidity [2]
- Incremental costs and cost-effectiveness (incorporating HRQL); budget impact.

9.2.4 PROTECTION FROM BIAS

To counteract the possibility of bias, objective outcome measures have been chosen as much as possible. The primary endpoint (CAA and their Z-scores) will be assessed by locally trained echocardiographers/cardiologists and reviewed centrally by at least one of two independent echocardiographers blinded to randomised allocation. Guidance for echocardiography interpretation will be agreed prior to the start of the trial and disseminated to local recruiting sites.

DocuSign Envelope ID: 651725BB-F200-474E-A72C-A62C1F4636C1

Receipt of rescue medication/second dose of IVIG and drug related clinical adverse events (AEs) are the only secondary outcome measures where there is substantial risk of subjectivity. Bias will be minimised by setting clear criteria in the protocol for management of rescue treatment (summarised above).

Every effort will be made to minimise loss to follow up and to ascertain outcomes completely thus avoiding bias from differential ascertainment between the randomised groups; given the disease severity this is anticipated to be minimal.

9.3 SAMPLE SIZE

KD-CAAP adopts a novel methodological approach utilizing two co-primary outcomes: one assessing the binary intervention of effectiveness of prednisolone (development of CAA or not); the other assessing intervention efficacy based on a continuous numeric outcome (average of the maximum of the Z-score of the diameter of the proximal right coronary artery or left anterior descending coronary artery, estimated across weeks 1, 2 and 6), improving the clinical relevance of the trial and using inverse probability weighting methods to adjust for non-compliance with the randomised intervention [72].

The advantage of the binary CAA primary endpoint is that it is clinically meaningful in terms of future mortality risk, and has been used in previous KD trials [51, 55]. Our estimated sample size of 262 children/adolescents provides 80% power to detect a reduction in CAA from 20% (based on existing survey data, summarised in **Section 0**) to 8% (based on reductions seen in “high-risk” children) (two-sided alpha=0.05). We assume that this endpoint can be completely ascertained, given its severity and the severity of the condition (meaning 3 month follow-up is likely to be almost complete). Recruiting 262 children/adolescents is realistic over 30 months.

There is, however, a possibility that more children/adolescents in the control group will move to rescue therapy than in the experimental group, resulting in dilution of any efficacy signal in relation to the binary endpoint of presence or absence of CAA. We therefore include a co-primary outcome of absolute maximum Z-score at weeks 1, 2, 6; analysed using generalised estimating equations (GEE) with inverse probability weighting to account for censoring of children/adolescents at the time they initiate rescue treatment (non-compliance with randomised strategy). This analysis would adjust for baseline Z-score using three strata: missing (to allow for the fact that some children/adolescents may not have a scan performed before randomisation), below median, above median. As well as accounting for differences in rescue treatment, this endpoint is continuous, so provides greater power. At any time point, 262 children/adolescents provides >80% power to detect changes in the maximum coronary artery Z-score of 0.4 times the standard deviation (two-sided alpha=0.05), assuming 13% children/adolescents may have missing values. There are no data to inform what effect estimate could be anticipated on continuous Z-scores, and therefore this effect size is pragmatic, based on the sample size for the binary CAA endpoint above.

The two endpoints will be considered separately, each with a nominal 0.05 level of significance, reflecting the fact that KD is a relatively small population in which it is important to generate randomised unbiased evidence and consider its totality [73]. The overall type I error will depend on the correlation between the two effect estimates which is unknown (no data available to estimate this), but will be estimated using bootstrapping at the final analysis. The two endpoints are addressing different aspects (efficacy vs effectiveness) on different outcomes (highly clinically relevant binary endpoint with lower intrinsic power as binary vs potentially less relevant continuous endpoint with higher power as continuous). Therefore it would be entirely possible to have significance on one and

not the other and the clinical judgement is that corticosteroids should still be used immediately in all children.

Given the size of the trial, subgroup analyses are planned only by minimisation factors (excluding country), namely age (<1 vs ≥1 year) and gender.

9.4 INTERIM MONITORING & ANALYSES

A Data Monitoring Committee (DMC) Charter will be drawn up that describes the membership of the DMC, relationships with other committees, terms of reference, decision-making processes, and the timing and frequency of interim analyses (with a description of stopping rules and/or guidelines).

The DMC is planned to meet within 6 months of the first participant recruited; the frequency of subsequent meetings will be determined by the DMC and could be more frequent if deemed necessary. The DMC can recommend premature closure or reporting of the trial, or that recruitment to any randomised group be discontinued or modified. Such recommendations would be made if, in the view of the DMC, there is proof beyond reasonable doubt that one of the allocated strategies is better than its comparator in terms of a difference of clinically significant magnitude in a primary outcome. The guiding statistical criteria for “proof beyond reasonable doubt” is a Haybittle-Peto type rule based on the 99.9% confidence interval in each interim analysis for both superiority and non-inferiority comparisons [74]. See **Section 14.4** for details on DMC membership.

9.5 ANALYSIS PLAN (BRIEF)

The analyses will be described in detail in a full Statistical Analysis Plan. This section summarises the main issues.

The primary analysis population is intention-to-treat, ITT, including all randomised patients, regardless of treatment received (using inverse probability weighting to adjust for rescue treatment for the efficacy co-primary endpoint as above). Primary analysis will adjust for randomisation stratification factors: secondary analyses will be unadjusted. Secondary outcome analysis will use logrank tests and Cox regression for time-to-event outcomes, exact tests and binomial regression for binary outcomes, and t-tests and normal linear regression (potentially on log-transformed data depending on the observed data distribution, adjusted for baseline values) for continuous outcomes. Ranksum tests will be used if there is gross departure from normality that cannot be adequately addressed by data transformation. GEE with independent working correlation will be used to provide global tests of repeated measures. If missing data and/or losses to follow-up reach >10% either multiple imputation or additional probability weights will be used to adjust for this. Adverse events will also be summarised by body system.

A Statistical Analysis Plan will be written and approved by the Trial Management Group (TMG), Trial Steering Committee (TSC) and the independent Data Monitoring Committee (DMC) before the first interim analysis is reviewed by the DMC.

For the economic analysis we will multiply unit costs and resource use to generate total costs for every trial participant. We will do this using unit cost values for each country, allowing costs to be assessed from the health care provider perspective in each country. We will convert the HRQL measures into utility scores using country-specific tariffs where available and will use these to compute three-month QALYs for every participant. We will then undertake a within-trial cost-effectiveness analysis

DocuSign Envelope ID: 651725BB-F200-474E-A72C-A62C1F4636C1

calculating the incremental cost per unit of outcome gained, where outcomes will be measured separately in terms of the co-primary outcomes of the trial and QALYs. We will undertake extensive deterministic and probabilistic sensitivity analysis, including calculating cost-effectiveness acceptability curves. We will also use the incremental cost estimates combined with projected population sizes to calculate the budget impact of the new treatment regime.

DocuSign Envelope ID: 651725BB-F200-474E-A72C-A62C1F4636C1

SUBSTUDIES

Substudies are defined as studies that do not relate to the randomised comparison.

10.1 PHARMACOMETRIC SUBSTUDY

Secondary analysis of pharmacodynamic (PD) endpoints for efficacy and corticosteroid toxicity (in the corticosteroid group) will be performed. Since no pharmacokinetic (PK) data will be collected, drug dose will be taken as the input with an estimated parameter for the decay in drug effect being used as a proxy for PK (K-PD approach) [75]. The efficacy analysis will consider linear and nonlinear mixed effects models of coronary Z-score over time with multivariable covariate analysis. The model will aim to identify possible differences between groups, and dose response (IVIG and corticosteroid cumulative dosing) corrected for important covariates such as CRP, age etc. The pGTI evolution in time with also be modelled using either linear or nonlinear mixed effects. The composite pGTI score will be treated as both a continuous variable and we will also consider sub-score modelling to assess which items are most susceptible to change in short-course therapy, which may lead to refinement of the score itself.

All models will be fitted with both NONMEM (version 7.4 or higher) and the new open-source R-package nlmixr (version 1.0 or higher). Model code will be made available as supplementary material in resulting manuscripts and shared on the DDmore model repository, thereby facilitating pharmacometric knowledge sharing across the c4c network and beyond.

10.2 DIAGNOSTIC BIOMARKER SUBSTUDY

The recruitment of large numbers of KD patients from multiple centres in Europe together with the collection of throat swab and low volume blood samples at multiple timepoints will be invaluable for future research on the diagnosis, aetiology, pathogenesis and genetics of the disease.

10.2.1 DIAGNOSIS

Over the past decade, there has been increasing evidence of the utility of RNA expression profiling to diagnose and understand the mechanisms involved in infectious and inflammatory diseases, and the development of minimal gene signatures that enable each condition to be diagnosed using small numbers of transcripts. KD can be distinguished from a wide range of infectious and inflammatory disorders with as few as 13 gene transcripts [3]. The small number of transcripts, and high level of sensitivity and specificity of the RNA signature, suggest that it could be developed as a diagnostic test. The KD-CAAP trial will enable validation of this approach, and investigation of whether RNA profiling can predict treatment response. Retrospective analysis of RNA samples will enable improved diagnostic accuracy of KD diagnosis for patients recruited to the trial.

In parallel to RNA profiling, collection of serial serum and plasma samples will enable a parallel proteomic and metabolomic approach.

10.2.2 IDENTIFICATION OF PATHOGENIC MECHANISMS AND AETIOLOGY

Collection of DNA, RNA, plasma, and throat swabs from the trial cohort will advance research on mechanisms and aetiology of KD, through interrogation of the host response and of potential viral and bacterial pathogens or triggers. A major international effort is underway to identify precipitating

DocuSign Envelope ID: 651725BB-F200-474E-A72C-A62C1F4636C1

infectious agents, including a metagenomic study of throat or nasopharyngeal aspirate samples. Samples taken on diagnosis from this trial will contribute to this larger study.

10.2.3 KAWASAKI DISEASE GENETICS

Genetic studies have shown important roles for gene variants in KD susceptibility and aneurysm formation. Patients recruited to the trial will be included in the ongoing International KD Genetics Consortium studies.

DocuSign Envelope ID: 651725BB-F200-474E-A72C-A62C1F4636C1

REGULATORY & ETHICAL ISSUES

11.1 COMPLIANCE

11.1.1 REGULATORY COMPLIANCE

This trial will be conducted in compliance with the approved protocol by the Sponsor, the Investigator, and delegated Investigator staff and Sub-investigator, in accordance with consensus ethics principles derived from international ethics guidelines, including the Declaration of Helsinki (Fortaleza, Brazil, October 2013), and the ICH guidelines for good clinical practice (GCP), General Data Protection Regulation (GDPR) and all applicable laws, rules and regulations.

11.1.2 SITE COMPLIANCE

An agreement will be in place between the sites and the CTU, setting out respective roles and responsibilities (see **Section 13 - Finance**).

The sites will inform the CTU as soon as they are aware of a possible serious breach of compliance, so that the CTU can report this breach if necessary within 7 days as per the UK regulatory requirements. For the purposes of the regulations, a 'serious breach' is one that is likely to affect to a significant degree:

- The safety or physical or mental integrity of the children/adolescents in the trial, or
- The scientific value of the trial

11.1.3 DATA COLLECTION & RETENTION

CRFs, clinical notes and administrative documentation should be kept in a secure location (for example, locked filing cabinets in a room with restricted access) and held for a minimum of 25 years after the end of the trial. During this period, all data should be accessible, with suitable notice, to the competent or equivalent authorities, the Sponsor, and other relevant parties in accordance with the applicable regulations. The data may be subject to an audit by the competent authorities. Medical files of children/adolescents in the trial should be retained in accordance with the maximum period of time permitted by the hospital, institution or private practice.

11.2 ETHICAL CONDUCT

11.2.1 ETHICAL CONSIDERATIONS

The risks of the safety of children/adolescents enrolled in the trial relate to the inherent risks of the drugs being studied. All drugs have side-effects. The trial will directly evaluate whether these potential risks are outweighed by potential benefits.

Some children/adolescents will also have blood taken for clinical management and also for research blood tests. The volumes of research blood required will be minimised wherever possible and are within recommended guidelines.

Children/adolescents will be informed (in an age appropriate fashion, since many patients in KD-CAAP will be infants or toddlers) fully of known risks and possible benefits by means of a patient information sheet for parents/carers and for children, and this will be reinforced by discussions with the trial research teams at the individual sites prior to enrolment.

DocuSign Envelope ID: 651725BB-F200-474E-A72C-A62C1F4636C1

Children/adolescent's confidentiality will be maintained throughout the trial. Data submitted on CRFs to CTU, and samples sent to central testing facilities, will be identified only by the trial number (including random check letters to improve accuracy of identification), as well as date, and time for samples.

Travel costs for the additional visit will be available and compensation will be given to participating families for their time, in line with local ethics policies.

11.2.2 Ethical Approvals

Before initiation of the trial at clinical sites, the protocol, all informed consent forms, and information materials to be given to the families will be submitted to an ethics committee for approval. Any further amendments will be submitted and approved by the ethics committee.

The rights of the families to refuse to participate in the trial without giving a reason must be respected. After the child/adolescent has entered into the trial, the clinician must remain free to give alternative treatment to that specified in the protocol, at any stage, if they feel it to be in the best interest of the participant. The reason for doing so, however, should be recorded; the child/adolescent will remain within the trial for the purpose of follow-up and for data analysis by the treatment option to which they have been allocated. Similarly, the parent/guardian must remain free to change their mind at any time about the protocol treatment and trial follow-up without giving a reason and without prejudicing the child's further care.

11.3 COMPETENT AUTHORITY APPROVALS

This protocol will be submitted to the national competent or equivalent authority, as appropriate in each country where the trial will be run.

The progress of the trial and safety issues will be reported to the competent authority, regulatory agency or equivalent in accordance with local requirements and practices in a timely manner.

Safety reports will be submitted to the competent authority in accordance with each authority's requirements in a timely manner.

11.4 TRIAL CLOSURE

End of trial is defined as 12 months after the last scheduled follow-up visit of the last randomised participant. This is to ensure sufficient time for data submission, data cleaning, verification of queries, database lock and final analysis. Each site will be closed once data cleaning is completed at that site, and the relevant regulatory authorities and ethics committee will be informed.

11.4.1 Sample storage and destruction

Specimens for which children/adolescents have consented will be stored and used for analyses as specified in the KD-CAAP protocol, patient information sheet and consent; once analyses are complete, any of these samples that remain will be disposed of according to standard laboratory procedures and guidelines in the respective countries. Samples will be stored for a maximum of 15 years.

DocuSign Envelope ID: 651725BB-F200-474E-A72C-A62C1F4636C1

INDEMNITY

The Sponsor of the trial is UCL. UCL holds insurance against claims from participants for injury caused by their participation in the clinical trial. Participants may be able to claim compensation if they can prove that UCL has been negligent. However, as this clinical trial is being carried out in a hospital, the hospital continues to have a duty of care to the participant of the clinical trial. UCL does not accept liability for any breach in the hospital's duty of care, or any negligence on the part of hospital employees. This applies whether the hospital is a National Health Service Trust in the UK or otherwise.

Participants may also be able to claim compensation for injury caused by participation in this clinical trial without the need to prove negligence on the part of UCL or another party. Participants who sustain injury and wish to make a claim for compensation should be advised to do so in writing in the first instance to the Chief Investigator, who will pass the claim to the Sponsor's Insurers, via the Sponsor's office.

Institutions selected to participate in this clinical trial shall provide clinical negligence insurance cover for harm caused by their employees and a copy of the relevant insurance policy or summary shall be provided to UCL, upon request.

DocuSign Envelope ID: 651725BB-F200-474E-A72C-A62C1F4636C1

FINANCE

KD-CAAP is funded by the Innovative Medicines Initiative 2 Joint Undertaking (JU), under grant agreement No 777389 that supports the Conect4children (c4c) research consortium. In kind support is provided by the UK MRC to the MRC CTU at UCL.

A written agreement with the site PI and c4c research consortium will outline the funding arrangements to sites.

OVERSIGHT & TRIAL COMMITTEES

There are a number of committees involved with the oversight of the trial. These committees are detailed below, and the relationship between them expressed in **Figure 2.**

Figure 2: Trial Organogram

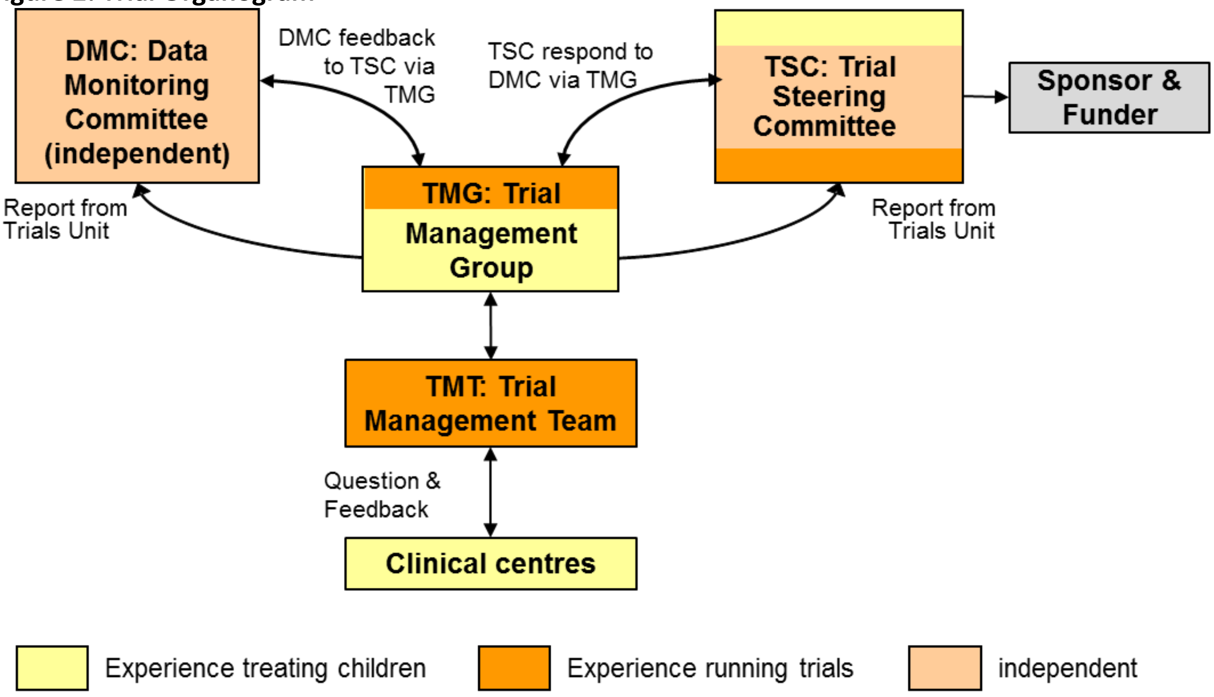

Note: DMC are sometimes referred to as Data Safety Monitoring Boards (DSMB). Trial Steering Committee includes community representatives, see below. Endpoint review committee refers to the two independent echocardiographers who will review scans blinded to randomised group (see **Section 6.5**).

14.1 TRIAL MANAGEMENT TEAM (TMT)

A Trial Management Team (TMT) will be formed to conduct the day-to-day management of the trial at the CTU. This will include the Co-Chief Investigators, trial statisticians, trial physician, clinical project manager, trial manager (TM) and data manager. The group will meet at least once per month, although may meet more often if required. The group will discuss issues related to the progress of the trial at the site and to ensure that the trial is running well.

14.2 TRIAL MANAGEMENT GROUP (TMG)

A Trial Management Group (TMG) will be formed comprising the two co-Chief Investigators, co-investigators and clinical and non-clinical members of the CTU. It will meet every 3-6 months depending on the stage of the trial generally by teleconference. This group will be chaired by the Chief Investigator(s) and all decisions regarding the overall running of the trial will be made in this forum with the exception of matters of fundamental importance to the viability of the trial or that require major changes to the protocol. These will be referred to the Trial Steering Committee (TSC). The full details can be found in the TMG Charter.

DocuSign Envelope ID: 651725BB-F200-474E-A72C-A62C1F4636C1

14.3 TRIAL STEERING COMMITTEE (TSC)

The Trial Steering Committee (TSC) has membership from the TMG plus independent members, including the Chair and community representatives. The role of the TSC is to provide overall supervision for the trial and provide advice through its independent Chair. The ultimate decision for the continuation of the trial lies with the TSC. Further details of TSC functioning are presented in the TSC Charter.

14.4 DATA MONITORING COMMITTEE (DMC)

An independent Data Monitoring Committee (DMC) will be formed. The DMC will be the only group who sees the confidential, accumulating data for the trial. Reports to the DMC will be produced by the CTU statisticians. The DMC is planned to meet within 6 months of the first participant recruited; the frequency of meetings will be determined by the DMC. The DMC will consider data using the statistical analysis plan (see **Section 9.5**) and will advise the TSC. The DMC can recommend premature closure or reporting of the trial, or that recruitment to any randomised group be discontinued.

Further details of DMC functioning, and the procedures for interim analysis and monitoring are provided in the DMC Charter.

14.5 ROLE OF TRIAL SPONSOR

UCL is the Sponsor of KD-CAAP and delegates this responsibility to the MRC CTU at UCL to oversee the implementation of the trial by ensuring that arrangements are put into place for adequate management, monitoring, analysis and reporting of the trial.

DocuSign Envelope ID: 651725BB-F200-474E-A72C-A62C1F4636C1

PATIENT AND PUBLIC INVOLVEMENT

Societi, the UK Foundation for Kawasaki Disease (led by Rachael McCormack) (https://www.societi.org.uk/) together with the Swedish patient organisation for young patients with rheumatic diseases (https://ungareumatiker.se) will ensure ongoing engagement of families, children and young people throughout the course of the trial through the expansion of their social media activities, international awareness raising activities and proactive partnership work with sister organisations. Importantly, information intended for families considering participation in the trial has been developed with input from Societi, and active feedback will be sought from recipients with scope to enhance family information materials during the trial period. Societi is well placed to provide relevant, family focussed materials which will help recruit, retain and enable continued connections with families following conclusion of the trial. It is anticipated that Societi will lead the delivery and distribution of a programme of trial updates for families, with a series of updates over the trial period planned. Liaising with sister organisations in European countries (where these exist) Societi will signpost information relevant to partner nations, and work if possible to achieve translated materials which can be shared widely for all core messages. It is a primary Societi policy that images of child/adolescent patients are never used in materials originated by Societi for charity purposes – to protect the privacy of child/adolescent patients. This policy will be applied to all trial communications.

Where appropriate, general clinician information will also be developed and distributed by Societi, complementing trial protocol and technical information provided by the trial team. Societi has a proven track record in the development of engaging, effective materials for both clinical and public audiences, with RCPCH endorsement of our current clinician information leaflet and a British Medical Association patient information award demonstrating the integrity of content Societi develops.

A patient representative will join the TSC and act as a liaison between the trial oversight, the trial management team and groups of patient and public representatives. Societi (https://www.societi.org.uk/) and the UK Kawasaki support group (https://www.societi.org.uk/kssg/) will also help advertise the trial, and help raise public awareness of KD and KD-CAAP.

DocuSign Envelope ID: 651725BB-F200-474E-A72C-A62C1F4636C1

PUBLICATION AND DISSEMINATION OF RESULTS

The KD-CAAP TSC is responsible for the data and specimens generated from the KD-CAAP trial; KD-CAAP trial data are not the property of individual participating investigators or health care facilities where the data were generated.

It is anticipated that a number of opportunities will arise for publication during the course of and following completion of the KD-CAAP trial. Publications include papers (including abstracts) for presentation at national and international meetings, as well as the preparation of manuscripts for peer-reviewed publication. In order to avoid disputes regarding authorship, it is important to establish a consensus approach that will provide a framework for all publications derived in full or in part from this clinical trial. The following approach is derived from the *Lancet* and from the publication policies used in other clinical trials coordinated by the CTU:

- All publications are to be approved by the TMG and TSC before submission for publication. Any publication arising before the end of the trial (not by randomised groups) will also be approved by the DMC in order to ensure that the primary objective of the trial (the randomised comparison) is not compromised. In particular, no analyses by randomised group of any outcome (primary, secondary or other) in either the main trial or associated sub-studies will be conducted or presented before the end of the trial, other than those for interim review by the DMC. The TMG and TSC will resolve problems of authorship and maintain the quality of publications.

- In line with MRC policy that the results of publicly-funded research should be freely available, manuscripts arising from the trial will, wherever possible, be submitted to peer-reviewed journals which enable Open Access via UK PubMed Central (PMC) within six months of the official date of final publication. All conference presentations will be made available as soon as possible after the event via the KD-CAAP website. All publications will acknowledge the trial's funding sources.

- For all publications, the TMG will nominate a chairperson or approve an individual’s request to chair a manuscript writing committee. The chair will usually be the primary or senior author. The chairperson is responsible for identifying fellow authors and for determining with that group the order of authorship that will appear on the manuscript. The TSC will resolve any problems of authorship and maintain the quality of publications.

- The TMG will maintain a list of investigators to be presented in an appendix at the end of the paper. This list will include investigators who contributed to the investigation being reported but who are not members of the writing committee, together with all relevant expert advisors and members of the TSC and DMC. All families who participated in the trial will be thanked as a group (not by name). In principle, sub-study reports should include all investigators for the main trial, although in some instances where a smaller number of investigators have made any form of contribution, it may be appropriate to abbreviate the listing. All headline authors in any publication arising from the main trial or sub-studies must have a made a substantive academic or project management contribution to the work that is being presented. “Substantive” must be defined by a written declaration of exactly what the contribution of any individual is believed to have been. In addition to fulfilling the criteria based on contribution, additional features that will be considered in selecting an authorship group will include the recruitment of children/adolescents who contributed data to any set of analyses contained in the manuscript and/or the conduct of analyses (laboratory and statistical), leadership and coordination of the project in the absence of a clear academic contribution.

DocuSign Envelope ID: 651725BB-F200-474E-A72C-A62C1F4636C1

- The data derived from this clinical trial are considered the property of the KD-CAAP TSC. The presentation or publication of any data collected by the participating investigators on children/adolescents entered into this trial is under the direct control of the TMG and TSC (and the DMC before the end of the trial). This is true whether the publication or presentation is concerned directly with the results of the trial or is associated with the trial in some other way. However, although individual participating investigators will not have any inherent right to perform analyses or interpretations or to make public presentations or seek publication of any of the data other than under the auspices of and with the approval of the TMG and TSC (and the DMC before the end of the trial), they will be encouraged to develop sub-studies or propose analyses subject to the approval by the TMG and TSC (and the DMC before the end of the trial). Any requests for access to raw data will be welcomed as long as they are scientifically valid and do not conflict with the integrity of the trial or ongoing analyses by the trial team.

Outcome data by randomised group will not be revealed to the participating investigators until the data collection phase and primary full analysis of the trial has been completed. This policy safeguards against possible bias affecting the data collection. The DMC will be monitoring the outcome results and may recommend that the trial be stopped for safety reasons, or if a definitive answer is reached earlier than the scheduled end of the trial.

DocuSign Envelope ID: 651725BB-F200-474E-A72C-A62C1F4636C1

DATA AND/OR SAMPLE SHARING

Data will be shared according to the CTU's controlled access approach [76], based on the following principles:

- No data should be released that would compromise an ongoing trial or study.
- There must be a strong scientific or other legitimate rationale for the data to be used for the requested purpose.
- Investigators who have invested time and effort into developing a trial or study should have a period of exclusivity in which to pursue their aims with the data, before key trial data are made available to other researchers.
- The resources required to process requests should not be under-estimated, particularly successful requests which lead to preparing data for release. Therefore adequate resources must be available in order to comply in a timely manner or at all, and the scientific aims of the study must justify the use of such resources.
- Data exchange complies with Information Governance and Data Security Policies in all of the relevant countries.

Data will be available for sharing after publication of the primary trial results. Researchers wishing to access data should contact the Trial Management Group in the first instance.

DocuSign Envelope ID: 651725BB-F200-474E-A72C-A62C1F4636C1

PROTOCOL AMENDMENTS

**Protocol v1.0 11-Dec-2019**	
**Protocol v2.0 05-Feb-2020**	
**Changes made**	**Sections updated**
Addition of the word 'additional' oral steroids to the experimental group	Page 11: Summary of Trial: Interventions to be Compared
Clarification that 'clinical' adverse events of any grade related to IVIG, aspirin or corticosteroids should be collected.	Page 12: Summary of Trial: Safety Secondary outcomes<br>Page 50: Section 6.7 Procedures for Assessing Safety<br>Page 54: 7.3 Investigator Responsibilities<br>Page 61/62: 9.2.2 Secondary Outcome Measures/9.2.4 Protection from Bias
Removal the following sentence related to IVIG and aspirin *'They are supplied to the trial participants according the protocol but are NOT under investigation.'*	Page 38: Trial Treatments
Update to the Funders grant agreement number	Page 12: Summary of Trial: Funder<br>Page 71: Section 13 Finance
Clarification that Echocardiograms and ECGs completed on a scheduled visit should be collected although they are not mandatory for the trial	Page 14: Table 1: Trial Assessment Schedule
Clarification that IVIG can be infused dependent as per standard of care within the member state	Page 11: Summary of Trial: Interventions to be Compared<br>Page 38: Section 5 Trial Treatments
Typographical error for reason that randomisation will be stratified, should be country rather than site	Page 60: 9.1 Method of randomisation
**Protocol v3.0 15-May-2020**	
**Changes made**	**Sections updated**
Addition of the ISRCTN number	Protocol Cover Page<br>Page 3: Trial Registration<br>Page 11: Summary of Trial
Amendment to the trial compliance statement to allow local national law requirements to be met in EU countries and clarification that in terms of confidentiality GDPR will be followed	Page 3: General Information<br>Page 67: 11.1 Regulatory Compliance<br>Page 59: 8.3.2 Confidentiality
Updates to Trial Administration – addition of CTU Data Manager and amendment to the address for the Nursing co-investigator	Page 4: CTU Staff and affiliates<br>Page 6: Other Responsible individuals
Amendment of the duration of aspirin given to participants to at least 21 days	Page 11: Summary of Trial: Interventions to be Compared<br>Page 38-43: 5 Trial Treatments

DocuSign Envelope ID: 651725BB-F200-474E-A72C-A62C1F4636C1

Clarification of the 48 hour assessment should be performed within the day 2 assessment	Page 11: Summary of Trial: Interventions to be Compared<br>Page 39-42: 5 Trial Treatments
Clarification that the maximum daily temperature will be collected from Day 5 until discharge or until the child/adolescent is afebrile for 2 calendar days	Page 15: Trial Assessment Schedule<br>Page 47: 6.1 Trial Assessment schedule<br>Page 48: 6.3 Information to be collected at all follow up assessments
Clarification on the documents required at site assessment	Page 32: 2.3 Site Assessment
Clarification of the inclusion criteria that the child/adolescent must be below the age of country specific consent for the duration of the trial	Page 33: 3.1 Inclusion Criteria
Minor typographical amendments to make the wording consistent within the inclusion criteria	Page 33: 3.1 Inclusion Criteria
Removal of randomisations being completed by the CTU over the phone	Page 37: 4.1 Randomisation Practicalities
Information on the timing of the first dose of corticosteroids	Page 40: 5.3.1 Products and treatment schedule
Amendment to the trial IMP dispensing and accountability requirements	Page 41: 5.3.2 Dispensing and accountability of IMP
Clarification of the data collected for treatment of KD.	Page 46: 5.8 Treatment Data Collection
Addition that adherence to aspirin will be collected using standardised diaries in the control and experimental group	Page 48: 6.3 Information to be collected at all follow up assessments
Clarification that echocardiograms collected from any unscheduled timepoints will be centrally assessed	Page 49: 6.5 Echocardiography and ECG
Clarification that only overdose of IMP which results in clinical symptoms of any grade is a notifiable event	Page 45: 5.5 Handling Cases of Trial Medication Overdose<br>Page 54: 7.2 Other Notable Events
Addition of detail regarding the use of IVIG and aspirin during pregnancy	Page 54: 7.2 Other Notable Events
Amendment to the TMG membership	Page 72: 14.2 Trial Management Group (TMG)
**Protocol v4.0 07-Apr-2021**	
**Changes made**	**Sections updated**
Addition of details where queries should be sent relating to the sponsorship of the trial	Page 3: General Information
Update to the details within Trial Administration include the addition of the emergency contact details for Paul Brogan and Despina Eleftheriou, addition of a trial manager to the CTU Staff and Affiliates, update to the address and contact details for Cardiology co-investigator Professor Robert Tulloh	Page 4 and 6: General Information
Addition that visits maybe conducted via telephone	Page 8: Lay Summary<br>Page 51: 6.3 Information to be collected at all follow up assessments

DocuSign Envelope ID: 651725BB-F200-474E-A72C-A62C1F4636C1

Clarification that the dose of aspirin should not be reduced until the participant has been afebrile for at least 48 hours	Page 10: Trial Schema<br>Page: 11: Summary of Trial<br>Page 42: 5.2 Control Group: Further management Based on assessment of fever and CRP response on Day 2 and on Day 5<br>Page 44: 5.3.3 Experimental Group: Further management based on assessment of fever and CRP response on Day 2 and Day 5
EudraCT# added	Page 11: Summary of Trial
Update to the wording ancillary studies to substudies	Page 12: Summary of Trial<br>Page 14: Trial Assessment Schedule<br>Page 71: Substudies
Addition of urine or blood pregnancy test for adolescents who are menstruating, and exclusion criteria as pregnant or breastfeeding.	Page 14: Trial Assessment Schedule<br>Page 37: 3.2 Exclusion Criteria<br>Page 58: 7.2.1 Pregnancy
Removal of LDL and HbA1c collection	Page 14: Trial Assessment Schedule<br>Page 38: 3.6 Samples to be taken as soon as consent is obtained<br>Page 50: 6.1 Trial Assessment Schedule
Update to the volume of research blood samples collected	Page 14: Trial Assessment Schedule<br>Page 38: 3.6 Samples to be taken as soon as consent is obtained
Update to the time points weight is collected at	Page 15: Trial Assessment Schedule<br>Page 50: 6.1 Trial Assessment Schedule<br>Page 51: 6.3 Information to be collected at all follow up assessments
Update to remove collection of temperature from the axilla throughout the protocol	Page 15: Trial Assessment Schedule<br>Page 42 &44: Trial Treatments<br>Page 50: 6.1 Trial Assessment Schedule<br>Page 51: 6.3 Information to be collected at all follow up assessments
Clarification that if the CHU9D is not available in the local language it does not have to be completed	Page 15: Trial Assessment Schedule<br>Page 54: 6.8.1 Health Economics
Clarification that the recommendations for the volume of blood collected relate to the research specific bloods	Page 15: Trial Assessment Schedule<br>Page 51: 6.1 Trial Assessment Schedule
Addition of Section 1.6 related to the benefit-risk assessment for the trial	Page 32 and 33: 1.6 Benefit-risk assessment
Addition of 'or known phenylketonuria to aspartame used in a formulation in an infant less than 12 weeks.' To exclusion 7. criteria	Page 37: 3.2 Exclusion Criteria
Rationale for collecting date of birth added	Page 39: Enrolment & Randomisation
The details regarding collection of weight for dosing has been moved from 5.3.1 to 5.	Page 41: Trial Treatments
Clarification that there are no trial specific temperature monitoring requirements for the IMP	Page 41: Trial Treatments
Inclusion of a +/- 20% flexibility in the dosing of IVIG and aspirin	Page 41: 5.1.1 Products & Treatment Schedule

DocuSign Envelope ID: 651725BB-F200-474E-A72C-A62C1F4636C1

Removal of wording surrounding the regular weight collection included in error	Page 44: 5.3.1 Products & Treatment Schedule
Clarification that enough IMP should be dispensed to reach the participants next visit or to allow the completion their duration of corticosteroids	Page 44: 5.3.2 Dispensing and Accountability of IMP
Addition of mitigation for provision of aspirin and corticosteroids	Page 46: 5.3.4 Stopping Drug Early
Clarification on immunisation guidance and addition that the administration of the COVID-19 vaccines should following the guidance as per any non-live vaccines	Page 49: 5.10 Medications Not Permitted
Addition of reporting pregnancy as a notable event.	Page 59: 7.2 Other notable events
Clarification that it is the 'diastolic' left eccentricity value that should be reported	Page 53: 6.5 Echocardiography and ECG
Typographical amendment change from 'future research' to 'further research'	Page 55: 6.9 Early Stopping Of Follow-Up
Addition of the sections of CRFs which can be considered as source data for the trial.	Page 62: 8.3.1 Direct Access to Children's Records
Update to the TMG members and timing for the timing of the DMC meeting	Page 77 and 78: Oversight and Trial Committees.
**Protocol v5.0**	
**Changes made**	**Sections updated**
EudraCT number	Added to cover page and<br>Page 3: General information
Addition of (Fortaleza, Brazil, October 2013) to the Declaration of Helsinki meeting which the trial is run in accordance with	Page 3: General information<br>Page 75: 11.1.1 Regulatory compliance
Update to MRC CTU staff	Page 5: General information
Update to exclusion criteria 10 to include active influenza infection.	Page 37: 3.2 Exclusion Criteria
Removal or wording 'In particular, the investigator must ensure that the children's anonymity will be maintained and that their identities are protected from unauthorised parties.'	Page 67: 8.3.2. Confidentiality
Update from a Standard Operating Procedure to Guidance	Page 69: 9.2.4 Protection from Bias
Update wording in section 9.4 to make it consistent with the wording changed in Protocol v4.0, Oversight and Trial Committees Section	Page 71: 9.4 Interim Monitoring & Analyses

APPENDICES

19.1 APPENDIX 1

The 'Division of AIDS (DIADS) Table for Grading the Severity of Adult and Pediatric Adverse Events: Corrected v2.1 July 2017' can be found at the following link:

https://rsc.niaid.nih.gov/sites/default/files/daidsgradingcorrectedv21.pdf

DocuSign Envelope ID: 651725BB-F200-474E-A72C-A62C1F4636C1

REFERENCES

1. McCrindle, B.W., et al., *Diagnosis, treatment, and long-term management of Kawasaki disease: a scientific statement for health professionals from the American Heart Association.* Circulation, 2017. **135**(17): p. e927-e999.
2. Brogan P, N.R., Ardoin SP, Cooper JC, De Benedetti F, Dicaire JF, Eleftheriou D, Feldman BM, Goldin J, Karol SE, Miloslavsky E, Price-Kuehne F, Skuse D, Stratakis CA, Webb N, Stone JH. , *Development of a Pediatric Glucocorticoid Toxicity Index.* Arthritis Rheumatol. 2018; 70 (suppl 10), 2018.
3. Wright, V.J., et al., *Diagnosis of Kawasaki Disease Using a Minimal Whole-Blood Gene Expression Signature.* JAMA Pediatr, 2018. **172**(10): p. e182293.
4. Daniel B. Hawcutt, A.C.R., Sabine Fuerst-Recktenwaldd and M.A.T. Tony Nunnb, , *Points to Consider when Planning the Collection of Blood or Tissue Samples in Clinical Trials of Investigational Medicinal Products in Children, Infants and Neonates. Rose K, van den Anker JN (eds): Guide to Paediatric Drug Development and Clinical Research.* Basel, Karger, 2010, pp 97–110, 2010., 2010.
5. de Graeff, N., et al., *European consensus-based recommendations for the diagnosis and treatment of Kawasaki disease - the SHARE initiative.* Rheumatology (Oxford), 2018.
6. Eleftheriou, D., et al., *Management of Kawasaki disease.* Archives of disease in childhood, 2014. **99**(1): p. 74-83.
7. Tulloh, R.M.R., et al., *Kawasaki disease: a prospective population survey in the UK and Ireland from 2013 to 2015.* Arch Dis Child. 2019 Jul;104(7):640-646.
8. Nakamura, Y., et al., *Cumulative incidence of Kawasaki disease in Japan.* Pediatr Int, 2018. **60**(1): p. 19-22.
9. Makino, N., et al., *Epidemiological observations of Kawasaki disease in Japan, 2013-2014.* Pediatr Int, 2018. **60**(6): p. 581-587.
10. Uehara, R. and E.D. Belay, *Epidemiology of kawasaki disease in Asia, Europe, and the United States.* Journal of Epidemiology, 2012. **22**(2): p. 79-85.
11. Dhillon, R., et al., *Management of Kawasaki disease in the British Isles.* Arch Dis Child, 1993. **69**(6): p. 631-6; discussion 637-8.
12. Mossberg, M., et al., *Epidemiology of primary systemic vasculitis in children: a population-based study from southern Sweden.* Scand J Rheumatol, 2018. **47**(4): p. 295-302.
13. Kikuta, H., S. Matsumoto, and T. Osato, *Kawasaki disease and Epstein-Barr virus.* Acta Paediatr Jpn, 1991. **33**(6): p. 765-70.
14. Brogan, P.A., et al., *Vbeta-restricted T cell adherence to endothelial cells: a mechanism for superantigen-dependent vascular injury.* Arthritis Rheum, 2004. **50**(2): p. 589-97.
15. Rowley, A.H., *Is Kawasaki disease an infectious disorder?* Int J Rheum Dis, 2018. **21**(1): p. 20-25.
16. Rowley, A.H., et al., *Ultrastructural, immunofluorescence, and RNA evidence support the hypothesis of a "new" virus associated with Kawasaki disease.* J Infect Dis, 2011. **203**(7): p. 1021-30.
17. Esper, F., et al., *Association between a novel human coronavirus and Kawasaki disease.* J Infect Dis, 2005. **191**(4): p. 499-502.
18. Chang, L.Y., et al., *Lack of association between infection with a novel human coronavirus (HCoV), HCoV-NH, and Kawasaki disease in Taiwan.* J Infect Dis, 2006. **193**(2): p. 283-6.
19. Rodo, X., et al., *Association of Kawasaki disease with tropospheric wind patterns.* Sci Rep, 2011. **1**: p. 152.
20. Rodo, X., et al., *Tropospheric winds from northeastern China carry the etiologic agent of Kawasaki disease from its source to Japan.* Proc Natl Acad Sci U S A, 2014. **111**(22): p. 7952-7.

DocuSign Envelope ID: 651725BB-F200-474E-A72C-A62C1F4636C1

21. Manlhiot, C., et al., *Environmental epidemiology of Kawasaki disease: Linking disease etiology, pathogenesis and global distribution.* PLoS One, 2018. **13**(2): p. e0191087.
22. Dergun, M., et al., *Familial occurrence of Kawasaki syndrome in North America.* Arch Pediatr Adolesc Med, 2005. **159**(9): p. 876-81.
23. Holman, R.C., et al., *Racial/ethnic differences in the incidence of Kawasaki syndrome among children in Hawaii.* Hawaii Med J, 2010. **69**(8): p. 194-7.
24. Uehara, R., et al., *Clinical features of patients with Kawasaki disease whose parents had the same disease.* Arch Pediatr Adolesc Med, 2004. **158**(12): p. 1166-9.
25. Weng, K.P., et al., *Cytokine genetic polymorphisms and susceptibility to Kawasaki disease in Taiwanese children.* Circ J, 2010. **74**(12): p. 2726-33.
26. Kuo, H.C., et al., *Polymorphisms of transforming growth factor-beta signaling pathway and Kawasaki disease in the Taiwanese population.* J Hum Genet, 2011. **56**(12): p. 840-5.
27. Onouchi, Y., et al., *A genome-wide association study identifies three new risk loci for Kawasaki disease.* Nat Genet, 2012. **44**(5): p. 517-21.
28. Burgner, D., et al., *A genome-wide association study identifies novel and functionally related susceptibility Loci for Kawasaki disease.* PLoS Genet, 2009. **5**(1): p. e1000319.
29. Onouchi, Y., et al., *ITPKC functional polymorphism associated with Kawasaki disease susceptibility and formation of coronary artery aneurysms.* Nat Genet, 2008. **40**(1): p. 35-42.
30. Khor, C.C., et al., *Genome-wide association study identifies FCGR2A as a susceptibility locus for Kawasaki disease.* Nat Genet, 2011. **43**(12): p. 1241-6.
31. Khor, C.C., et al., *Genome-wide linkage and association mapping identify susceptibility alleles in ABCC4 for Kawasaki disease.* J Med Genet, 2011. **48**(7): p. 467-72.
32. Onouchi, Y., et al., *ITPKC and CASP3 polymorphisms and risks for IVIG unresponsiveness and coronary artery lesion formation in Kawasaki disease.* Pharmacogenomics J, 2013. **13**(1): p. 52-9.
33. Kim, J.J., et al., *A genome-wide association analysis reveals 1p31 and 2p13.3 as susceptibility loci for Kawasaki disease.* Hum Genet, 2011. **129**(5): p. 487-95.
34. Sinha, R. and T. Balakumar, *BCG reactivation: a useful diagnostic tool even for incomplete Kawasaki disease.* Arch Dis Child, 2005. **90**(9): p. 891.
35. Durongpisitkul, K., et al., *The prevention of coronary artery aneurysm in Kawasaki disease: a meta-analysis on the efficacy of aspirin and immunoglobulin treatment.* Pediatrics, 1995. **96**(6): p. 1057-1061.
36. Newburger, J.W., et al., *The treatment of Kawasaki syndrome with intravenous gamma globulin.* New England Journal of Medicine, 1986. **315**(6): p. 341-347.
37. Newburger, J.W., et al., *Diagnosis, treatment, and long-term management of Kawasaki disease.* Circulation, 2004. **110**(17): p. 2747-2771.
38. Newburger, J.W., et al., *A single intravenous infusion of gamma globulin as compared with four infusions in the treatment of acute Kawasaki syndrome.* New England Journal of Medicine, 1991. **324**(23): p. 1633-1639.
39. Ho, L.G.Y. and N. Curtis, *What dose of aspirin should be used in the initial treatment of Kawasaki disease?* Archives of disease in childhood, 2017: p. archdischild-2017-313538.
40. Shah, V., et al., *Cardiovascular status after Kawasaki disease in the UK.* Heart, 2015: p. heartjnl-2015-307734.
41. Terai, M. and S.T. Shulman, *Prevalence of coronary artery abnormalities in Kawasaki disease is highly dependent on gamma globulin dose but independent of salicylate dose.* The Journal of pediatrics, 1997. **131**(6): p. 888-893.
42. Lyskina, G., et al., *Cardiovascular outcomes following Kawasaki disease in Moscow, Russia: A single center experience.* Glob Cardiol Sci Pract, 2017. **2017**(3): p. e201723.
43. Friedman, K.G., et al., *Coronary Artery Aneurysms in Kawasaki Disease: Risk Factors for Progressive Disease and Adverse Cardiac Events in the US Population.* J Am Heart Assoc, 2016. **5**(9).

DocuSign Envelope ID: 651725BB-F200-474E-A72C-A62C1F4636C1

44. Jakob, A., et al., *Kawasaki Disease in Germany: A Prospective, Population-based Study Adjusted for Underreporting.* Pediatr Infect Dis J, 2016. **35**(2): p. 129-34.
45. Manlhiot, C., et al., *Improved classification of coronary artery abnormalities based only on coronary artery z-scores after Kawasaki disease.* Pediatr Cardiol, 2010. **31**(2): p. 242-9.
46. Suda, K., et al., *Long-term prognosis of patients with Kawasaki disease complicated by giant coronary aneurysms: a single-institution experience.* Circulation, 2011. **123**(17): p. 1836-42.
47. Daniels, L.B., et al., *Prevalence of Kawasaki disease in young adults with suspected myocardial ischemia.* Circulation, 2012. **125**(20): p. 2447-53.
48. Davies, S., et al., *Predicting IVIG resistance in UK Kawasaki disease.* Arch Dis Child, 2015. **100**(4): p. 366-8.
49. Jakob, A., et al., *Failure to Predict High-risk Kawasaki Disease Patients in a Population-based Study Cohort in Germany.* Pediatr Infect Dis J, 2018. **37**(9): p. 850-855.
50. Kobayashi, T., et al., *Prediction of intravenous immunoglobulin unresponsiveness in patients with Kawasaki disease.* Circulation, 2006. **113**(22): p. 2606-12.
51. Kobayashi, T., et al., *Efficacy of immunoglobulin plus prednisolone for prevention of coronary artery abnormalities in severe Kawasaki disease (RAISE study): a randomised, open-label, blinded-endpoints trial.* Lancet, 2012. **379**(9826): p. 1613-20.
52. Newburger, J.W., et al., *Randomized trial of pulsed corticosteroid therapy for primary treatment of Kawasaki disease.* N Engl J Med, 2007. **356**(7): p. 663-75.
53. Miyata, K., et al., *Efficacy and safety of intravenous immunoglobulin plus prednisolone therapy in patients with Kawasaki disease (Post RAISE): a multicentre, prospective cohort study.* Lancet Child Adolesc Health, 2018. **2**(12): p. 855-862.
54. Chen, S., et al., *Coronary Artery Complication in Kawasaki Disease and the Importance of Early Intervention : A Systematic Review and Meta-analysis.* JAMA Pediatr, 2016. **170**(12): p. 1156-1163.
55. Inoue, Y., et al., *A multicenter prospective randomized trial of corticosteroids in primary therapy for Kawasaki disease: clinical course and coronary artery outcome.* J Pediatr, 2006. **149**(3): p. 336-341.
56. Harwood, R., et al., *A national consensus management pathway for paediatric inflammatory multisystem syndrome temporally associated with COVID-19 (PIMS-TS): results of a national Delphi process.* Lancet Child Adolesc Health, 2021. **5**(2): p. 133-141.
57. Papadopoulou, C., et al., *Management of severe hyperinflammation in the COVID-19 era: the role of the rheumatologist.* Rheumatology (Oxford), 2021. **60**(2): p. 911-917.
58. Grasa, C.D., et al., *Kawasaki disease in infants 3 months of age and younger: a multicentre Spanish study.* Ann Rheum Dis, 2019. **78**(2): p. 289-290.
59. Guo, Y., et al., *Adverse Effects of Immunoglobulin Therapy.* Front Immunol, 2018. **9**: p. 1299.
60. European Medicines Agency, *Assessment report for Okrido 6 mg/ml Oral Solution (Prednisolone sodium phosphate) (https://www.ema.europa.eu/en/documents/referral/okrido-article-29-referral-chmp-assessment-report_en.pdf).* 2013.
61. Medicines and Healthcare Products Regulatory Agency, *Public Assessment Report for Prednisolone 5mg soluble tablets (http://www.mhra.gov.uk/home/groups/par/documents/websiteresources/con482923.pdf).* 2014.
62. Miloslavsky, E.M., et al., *Development of a Glucocorticoid Toxicity Index (GTI) using multicriteria decision analysis.* Ann Rheum Dis, 2017. **76**(3): p. 543-546.
63. Lopez, L., et al., *Recommendations for quantification methods during the performance of a pediatric echocardiogram: a report from the Pediatric Measurements Writing Group of the American Society of Echocardiography Pediatric and Congenital Heart Disease Council.* J Am Soc Echocardiogr, 2010. **23**(5): p. 465-95; quiz 576-7.

DocuSign Envelope ID: 651725BB-F200-474E-A72C-A62C1F4636C1

64. Lopez, L., et al., *Relationship of Echocardiographic Z Scores Adjusted for Body Surface Area to Age, Sex, Race, and Ethnicity: The Pediatric Heart Network Normal Echocardiogram Database.* Circ Cardiovasc Imaging, 2017. **10**(11).

65. Stevens, K., *Valuation of the Child Health Utility 9D Index.* Pharmacoeconomics, 2012. **30**(8): p. 729-47.

66. Varni, J., et al., *The PedsQLTM 4.0 Generic Core Scales: Sensitivity, responsiveness, and impact on clinical decision-making.* Journal of Behavioral Medicine, 2002. **25**: p. 175 - 193.

67. Varni, J.W., M. Seid, and P.S. Kurtin, *PedsQL™ 4.0: Reliability and validity of the Pediatric Quality of Life Inventory™ Version 4.0 Generic Core Scales in healthy and patient populations.* Medical Care, 2001. **39**.

68. Kemp, M.W., et al., *The clinical use of corticosteroids in pregnancy.* Hum Reprod Update, 2016. **22**(2): p. 240-59.

69. Bandoli, G., et al., *A review of systemic corticosteroid use in pregnancy and the risk of select pregnancy and birth outcomes.* Rheum Dis Clin North Am, 2017. **43**(3): p. 489-502.

70. Clark, A.L., *Clinical uses of intravenous immunoglobulin in pregnancy.* Clin Obstet Gynecol, 1999. **42**(2): p. 368-80.

71. Cui, Y., B. Zhu, and F. Zheng, *Low-dose aspirin at </=16 weeks of gestation for preventing preeclampsia and its maternal and neonatal adverse outcomes: A systematic review and meta-analysis.* Exp Ther Med, 2018. **15**(5): p. 4361-4369.

72. Hernan, M.A. and J.M. Robins, *Per-Protocol Analyses of Pragmatic Trials.* N Engl J Med, 2017. **377**(14): p. 1391-1398.

73. Parmar, M.K., M.R. Sydes, and T.P. Morris, *How do you design randomised trials for smaller populations? A framework.* BMC Med, 2016. **14**(1): p. 183.

74. Lai, T.L., M.C. Shih, and G. Zhu, *Modified Haybittle–Peto group sequential designs for testing superiority and non-inferiority hypotheses in clinical trials.* Statistics in medicine, 2006. **25**(7): p. 1149-1167.

75. Jacqmin, P., et al., *Modelling response time profiles in the absence of drug concentrations: definition and performance evaluation of the K-PD model.* J Pharmacokinet Pharmacodyn, 2007. **34**(1): p. 57-85.

76. Smith, C.T., et al., *How should individual participant data (IPD) from publicly funded clinical trials be shared?* BMC medicine, 2015. **13**(1): p. 298.

DocuSign

Certificate Of Completion

Envelope Id: 651725BBF200474EA72CA62C1F4636C1

Status: Completed

Subject: Please DocuSign: KD-CAAP Protocol V5.0_22Oct2021_clean.docx

Source Envelope:

Document Pages: 93

Signatures: 3

Certificate Pages: 5

Initials: 0

AutoNav: Enabled

EnvelopeId Stamping: Enabled

Time Zone: (UTC) Dublin, Edinburgh, Lisbon, London

Envelope Originator:
Cara Purvis
90 High Holborn 2nd Floor London
London, London WC1V 6LJ
c.purvis@ucl.ac.uk
IP Address: 128.40.216.238

Record Tracking

Status: Original	Holder: Cara Purvis	Location: DocuSign
27 October 2021 | 11:09	c.purvis@ucl.ac.uk	

Signer Events | Signature | Timestamp

Despina Eleftheriou
Despina.Eleftheriou@gosh.nhs.uk
Prof
Security Level: Email, Account Authentication (Optional)

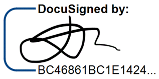

Signature Adoption: Drawn on Device
Using IP Address: 92.40.177.229
Signed using mobile

Sent: 27 October 2021 | 11:11
Viewed: 27 October 2021 | 11:16
Signed: 27 October 2021 | 11:16

Electronic Record and Signature Disclosure:
Accepted: 09 February 2021 | 16:00
ID: 10823482-b027-4b70-829f-4039f6ae6040

Paul Brogan
p.brogan@ucl.ac.uk
Prof
Security Level: Email, Account Authentication (Optional)

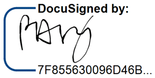

Signature Adoption: Drawn on Device
Using IP Address: 82.36.101.42
Signed using mobile

Sent: 27 October 2021 | 11:11
Viewed: 27 October 2021 | 11:45
Signed: 27 October 2021 | 11:45

Electronic Record and Signature Disclosure:
Accepted: 27 October 2021 | 11:45
ID: cb55ebc3-bc89-4a58-9ebd-c4cd937978c7

Sarah Walker
sarah.walker@ndm.ox.ac.uk
Security Level: Email, Account Authentication (Optional)

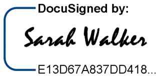

Signature Adoption: Pre-selected Style
Using IP Address: 31.50.186.208

Sent: 27 October 2021 | 11:11
Viewed: 27 October 2021 | 11:30
Signed: 27 October 2021 | 11:30

Electronic Record and Signature Disclosure:
Accepted: 27 October 2021 | 11:30
ID: da24a19c-c17b-4556-bdf0-aa93b8f7e0d5

In Person Signer Events | Signature | Timestamp

Editor Delivery Events | Status | Timestamp

Agent Delivery Events | Status | Timestamp

Intermediary Delivery Events | Status | Timestamp

Certified Delivery Events | Status | Timestamp

Carbon Copy Events	Status	Timestamp

Witness Events	Signature	Timestamp

Notary Events	Signature	Timestamp

Envelope Summary Events	Status	Timestamps
Envelope Sent	Hashed/Encrypted	27 October 2021 | 11:11
Certified Delivered	Security Checked	27 October 2021 | 11:30
Signing Complete	Security Checked	27 October 2021 | 11:30
Completed	Security Checked	27 October 2021 | 11:45

Payment Events	Status	Timestamps

Electronic Record and Signature Disclosure

ELECTRONIC RECORD AND SIGNATURE DISCLOSURE

From time to time, MRC Clinical Trials Unit at UCL (we, us or Company) may be required by law to provide to you certain written notices or disclosures. Described below are the terms and conditions for providing to you such notices and disclosures electronically through the DocuSign system. Please read the information below carefully and thoroughly, and if you can access this information electronically to your satisfaction and agree to this Electronic Record and Signature Disclosure (ERSD), please confirm your agreement by selecting the check-box next to 'I agree to use electronic records and signatures' before clicking 'CONTINUE' within the DocuSign system.

Getting paper copies

At any time, you may request from us a paper copy of any record provided or made available electronically to you by us. You will have the ability to download and print documents we send to you through the DocuSign system during and immediately after the signing session and, if you elect to create a DocuSign account, you may access the documents for a limited period of time (usually 30 days) after such documents are first sent to you. After such time, if you wish for us to send you paper copies of any such documents from our office to you, you will be charged a $0.00 per-page fee. You may request delivery of such paper copies from us by following the procedure described below.

Withdrawing your consent

If you decide to receive notices and disclosures from us electronically, you may at any time change your mind and tell us that thereafter you want to receive required notices and disclosures only in paper format. How you must inform us of your decision to receive future notices and disclosure in paper format and withdraw your consent to receive notices and disclosures electronically is described below.

Consequences of changing your mind

If you elect to receive required notices and disclosures only in paper format, it will slow the speed at which we can complete certain steps in transactions with you and delivering services to you because we will need first to send the required notices or disclosures to you in paper format, and then wait until we receive back from you your acknowledgment of your receipt of such paper notices or disclosures. Further, you will no longer be able to use the DocuSign system to receive required notices and consents electronically from us or to sign electronically documents from us.

All notices and disclosures will be sent to you electronically

Unless you tell us otherwise in accordance with the procedures described herein, we will provide electronically to you through the DocuSign system all required notices, disclosures, authorizations, acknowledgements, and other documents that are required to be provided or made available to you during the course of our relationship with you. To reduce the chance of you inadvertently not receiving any notice or disclosure, we prefer to provide all of the required notices and disclosures to you by the same method and to the same address that you have given us. Thus, you can receive all the disclosures and notices electronically or in paper format through the paper mail delivery system. If you do not agree with this process, please let us know as described below. Please also see the paragraph immediately above that describes the consequences of your electing not to receive delivery of the notices and disclosures electronically from us.

How to contact MRC Clinical Trials Unit at UCL:

You may contact us to let us know of your changes as to how we may contact you electronically, to request paper copies of certain information from us, and to withdraw your prior consent to receive notices and disclosures electronically as follows:
To contact us by email send messages to: s.assam@ucl.ac.uk

To advise MRC Clinical Trials Unit at UCL of your new email address

To let us know of a change in your email address where we should send notices and disclosures electronically to you, you must send an email message to us at s.assam@ucl.ac.uk and in the body of such request you must state: your previous email address, your new email address. We do not require any other information from you to change your email address.

If you created a DocuSign account, you may update it with your new email address through your account preferences.

To request paper copies from MRC Clinical Trials Unit at UCL

To request delivery from us of paper copies of the notices and disclosures previously provided by us to you electronically, you must send us an email to s.assam@ucl.ac.uk and in the body of such request you must state your email address, full name, mailing address, and telephone number. We will bill you for any fees at that time, if any.

To withdraw your consent with MRC Clinical Trials Unit at UCL

To inform us that you no longer wish to receive future notices and disclosures in electronic format you may:

i. decline to sign a document from within your signing session, and on the subsequent page, select the check-box indicating you wish to withdraw your consent, or you may;

ii. send us an email to s.assam@ucl.ac.uk and in the body of such request you must state your email, full name, mailing address, and telephone number. We do not need any other information from you to withdraw consent.. The consequences of your withdrawing consent for online documents will be that transactions may take a longer time to process..

Required hardware and software

The minimum system requirements for using the DocuSign system may change over time. The current system requirements are found here: https://support.docusign.com/guides/signer-guide-signing-system-requirements.

Acknowledging your access and consent to receive and sign documents electronically

To confirm to us that you can access this information electronically, which will be similar to other electronic notices and disclosures that we will provide to you, please confirm that you have read this ERSD, and (i) that you are able to print on paper or electronically save this ERSD for your future reference and access; or (ii) that you are able to email this ERSD to an email address where you will be able to print on paper or save it for your future reference and access. Further, if you consent to receiving notices and disclosures exclusively in electronic format as described herein, then select the check-box next to ‘I agree to use electronic records and signatures’ before clicking ‘CONTINUE’ within the DocuSign system.

By selecting the check-box next to ‘I agree to use electronic records and signatures’, you confirm that:

- You can access and read this Electronic Record and Signature Disclosure; and
- You can print on paper this Electronic Record and Signature Disclosure, or save or send this Electronic Record and Disclosure to a location where you can print it, for future reference and access; and
- Until or unless you notify MRC Clinical Trials Unit at UCL as described above, you consent to receive exclusively through electronic means all notices, disclosures, authorizations, acknowledgements, and other documents that are required to be provided or made available to you by MRC Clinical Trials Unit at UCL during the course of your relationship with MRC Clinical Trials Unit at UCL.
