# Supplementary material for: Multi-centre, randomised, open-label, blinded endpoint assessed, trial of corticosteroids plus intravenous immunoglobulin (IVIG) and aspirin, versus IVIG and aspirin for prevention of coronary artery aneurysms (CAA) in Kawasaki disease (KD): the KD-CAA prevention (KD-CAAP) trial
Source: eClinicalMedicine. 2026 Jul 13;97:104044. doi: 10.1016/j.eclinm.2026.104044 (PMC13382441; doi:10.1016/j.eclinm.2026.104044)
Supplement: Statistical analysis plan [file mmc5.pdf]

Docusign Envelope ID: D60D4999-E9B7-42C5-B5B0-35B5F15967A7

KD-CAAP: Kawasaki Disease Coronary Artery Aneurysm Prevention trial

Statistical Analysis Plan version 5.0 22 November 2024

Multi-centre, randomised, open-label, blinded endpoint assessed, trial of corticosteroids plus intravenous immunoglobulin (IVIG) and aspirin, versus IVIG and aspirin for prevention of coronary artery aneurysms in Kawasaki disease

ISRCTN71987471

Docusign Envelope ID: D60D4999-E9B7-42C5-B5B0-35B5F15967A7

KD-CAAP
Statistical Analysis Plan

Version Number and Date: Version 5.0 22-Nov-2024

In relation to protocol v5.0

**Author**	**Position**	**Signature**	**Date**
Roisin Connon	Delegated Statistician, MRC CTU	DocuSigned by: Roisin Connon 1671D28A21C9466...	22-Nov-2024
**Approved by**			
Professor A. Sarah Walker	Trial Statistician, MRC CTU	Signed by: Ann Sarah Walker A21A8226533E49A...	22-Nov-2024
Professor Paul Brogan	Chief Investigator, UCL GOSH	DocuSigned by: Paul Brogan 9B9612DDBB3049F...	25-Nov-2024
Professor Despina Eleftheriou	Chief Investigator, UCL GOSH	Signed by: Despina Eleftheriou BD2BDAFC2A364D6...	22-Nov-2024

Docusign Envelope ID: D60D4999-E9B7-42C5-B5B0-35B5F15967A7

Revision History

Version	Author	Date	Reason for Revision
Draft 0.1	Dr A. Sarah Walker	15/05/2020	Protocol version 3.0
Draft 0.2	Roisin Connon	04/11/2021	Roisin Connon first draft
Draft 0.3	Roisin Connon	23/11/2021	Incorporated comments from Sarah Walker
Draft 0.4	Roisin Connon	06/12/2021	Edits after comments from Despina Eleftheriou
Draft 0.5	Roisin Connon	25/1/2022	Added clarifications after comments from Toby Prevost and Becky Turner
Version 1.0	Roisin Connon	26/1/2022	Accepted all changes from v0.5
Draft 1.1	Roisin Connon	25/5/2023	Added estimands, details to primary outcome analysis, and Bayesian secondary analysis. Other details added following review of SAP content checklist
Draft 1.2	Roisin Connon	9/6/2023	Updated following SW comments
Version 2.0	Roisin Connon	9/6/2023	Accepted all changes in v1.2 and upversioned.
Draft 2.1	Roisin Connon	13/3/2024	Added additional analysis requested at previous DMC meeting, and clarifications to analysis of primary endpoints.
Version 3.0	Roisin Connon	19/3/2024	Accepted all changes from v2.1
Draft 3.1	Roisin Connon	25/10/2024	Added exploratory subgroup analyses requested by the DMC added to section 7.10. Added unweighted and baseline-only weighted models to analysis of continuous endpoint. Correction to Bayesian secondary analysis section.
Version 4.0	Roisin Connon	14/11/2024	Accepted all changes from v3.1.
Draft 4.1	Roisin Connon	19/11/2024	Added secondary analysis relating to day 0 CAAs in section
Version 5.0	Roisin Connon	22/11/2024	Accepted all changes from v4.1.

Contents

1. Introduction 5
1.1 Background 5
1.2 Objectives 5

2. Study methods 5
2.1 Design 5
2.2 Randomisation 6
2.3 Outcome measures 7
2.4 Estimands 8
2.5 Sample size 9
2.6 Interim analyses 9
2.7 Stopping guidelines 10
2.8 Final analysis 10

3. Trial population 11
3.1 Selection of patients 11

4. Data 12
4.1 CRF forms and variables 12
4.2 Management of datasets 13
4.3 Data verification 13

5. Derivation of data to be analysed 13
5.1 Definitions 13
5.2 Data transformations and coding 14

6. Analysis principles 15
6.1 Statistical significance and p-values 15
6.2 Analysis populations 15
6.3 Protection from bias 15
6.4 Missing data 16

7. Statistical analysis 16
7.1 Recruitment 16
7.2 Protocol deviations 17
7.3 Baseline characteristics 17
7.4 Description of follow-up 17
7.5 Result of Day 2 and Day 5 assessments 18
7.6 Treatment details 18
7.7 Efficacy analyses 19
7.7.1 Primary outcome measures 19
7.7.2 Secondary outcome measures 21
7.7.3 Other outcome measures 22
7.8 Safety analyses 23
7.8.1 Secondary outcome measures 23
7.9 Other analyses 23
7.10 Subgroup analyses 23

8. Dissemination of results 24

9. References 24

1. INTRODUCTION

1.1 Background

Kawasaki disease (KD) is an acute self-limiting inflammatory vasculitis affecting predominantly medium-sized arteries, particularly the coronary arteries causing coronary artery aneurysms (CAA). Treatment of KD with intravenous immunoglobulin (IVIG) and aspirin has been shown to reduce the occurrence of CAA. However, several recent studies conducted in different European countries (UK, Sweden, and Germany), Russia, and the United States have found high rates of coronary complications despite IVIG. Corticosteroids are an effective treatment for virtually all forms of vasculitis, but they have not been widely adopted as first-line treatment in unselected KD cases. European SHARE guidelines for KD recommend adjunctive corticosteroids for high-risk patients, but identifying such patients in Caucasian populations is difficult. Given the high CAA rates emerging from several countries, and the lack of risk assessment tools to accurately identify such cases, it is reasonable now to argue that all European KD patients are at significant risk of CAA despite IVIG, and could potentially benefit from primary treatment with corticosteroids.

1.2 Objectives

The overarching goal is to optimise the treatment of KD in children/adolescents across Europe. KD-CAAP will test the hypothesis that adding immediate adjunctive corticosteroid treatment to IVIG and aspirin will reduce CAA rates in unselected KD patients across Europe compared with IVIG and aspirin alone.

The primary aim of the KD-CAAP trial is therefore to establish:

1. the effectiveness and efficacy of adjunctive corticosteroid therapy combined with IVIG/aspirin for prevention of CAA in unselected patients with KD across Europe;

Secondary aims are to establish:

2. the safety of adjunctive corticosteroid therapy combined with IVIG/aspirin for prevention of CAA in KD;
3. whether adjunctive corticosteroid therapy reduces the duration of fever and length of hospitalisation for patients with KD;
4. the incremental cost-effectiveness ratio for corticosteroid therapy, expressed as the cost per QALY gained, from cost and utility data measured via resource use forms and the Child Health Utility 9D questionnaire.
5. the utility of the Paediatric Glucocorticoid Toxicity (pGTI) tool to assess corticosteroid toxicity.

2. STUDY METHODS

2.1 Design

KD-CAAP is a multi-centre, randomised, open-label, blinded endpoint assessed parallel group superiority trial comparing immediate corticosteroids to standard of care IVIG and aspirin for children with Kawasaki disease, with a 1:1 allocation ratio.

Figure 1 displays the trial schema.

Figure 1: Trial schema

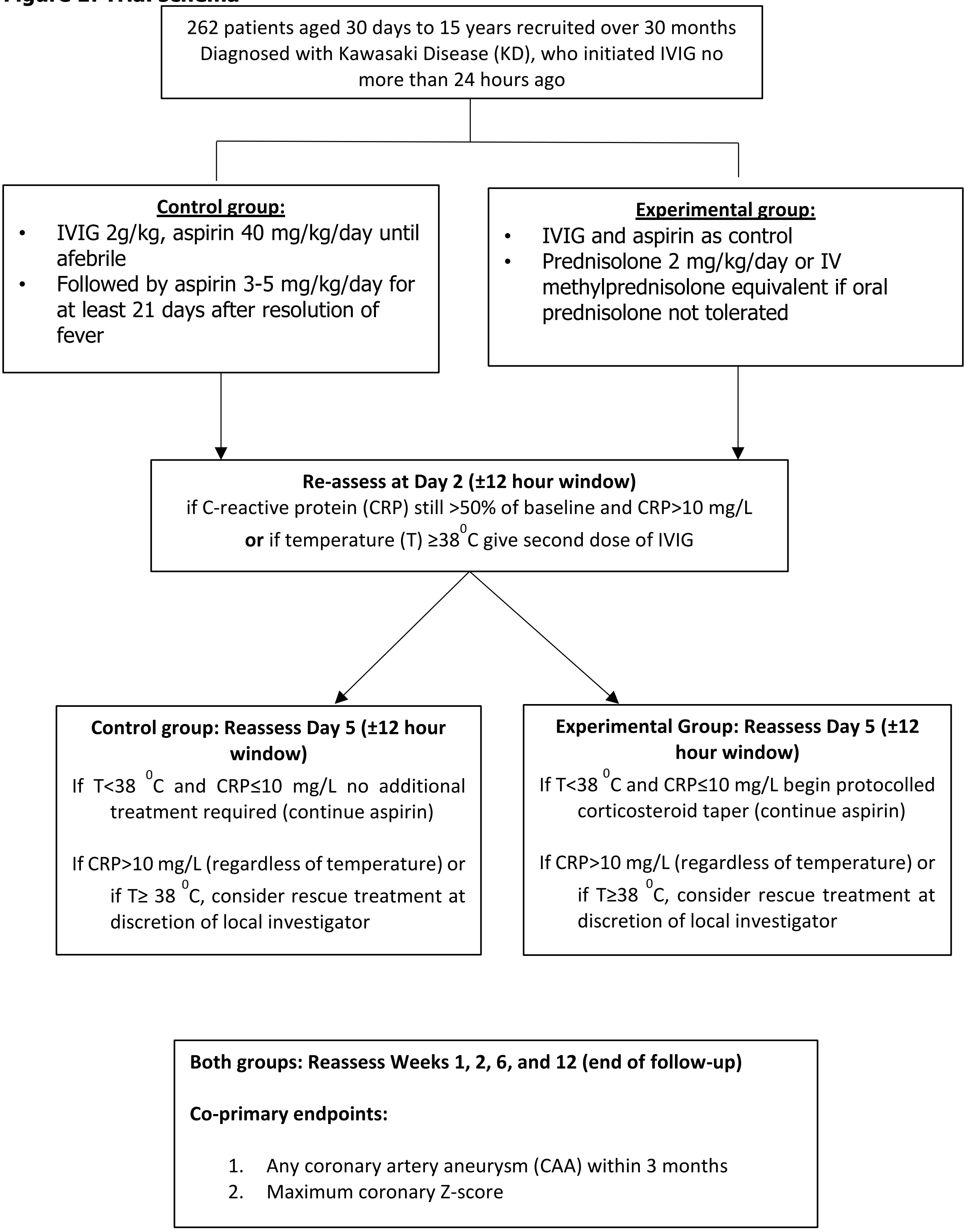

2.2 Randomisation

Randomisation will be performed at the MRC CTU using a computer algorithm concealed from the investigators/trial management staff, and accessed by either CTU staff or delegated site staff online. Randomisation will use minimisation with a random element, stratified for age (<1 year and ≥1 year), sex and recruiting country. These factors have been chosen because

epidemiological data suggest worse outcomes among very young patients (< 1 years) and male children/adolescents. Before randomisation, the participant's eligibility for enrolment will be confirmed. Parents/guardians must confirm that they have read the relevant patient information sheets and have provided written informed consent to enter into the trial.

2.3 Outcome measures

The two co-primary outcome measures are:

- Any CAA (definition below) documented within the 12 weeks of trial follow-up (to assess overall effectiveness of the strategy of immediate corticosteroids in preventing CAA, expecting that some patients will receive rescue treatment before reaching this endpoint in both randomised groups).
- An average estimate across weeks 1, 2, and 6 of the maximum of the Z-score of the internal diameters of the proximal right coronary artery or left anterior descending coronary artery, adjusting for rescue treatment (to assess the direct efficacy of corticosteroids).

CAA is defined as any of

- luminal diameter >3.0 mm in a child <5 years
- luminal diameter >4.0 mm in a child/adolescent ≥5 years
- internal diameter of a segment at least 1.5 times that of an adjacent segment or when a luminal contour is clearly irregular
- luminal internal diameter Z-score of ≥2.5

Z-scores for internal coronary artery diameter will be documented based on normative data: http://www.parameterz.com/refs/lopez-circimaging-2017.

The following secondary outcome measures will be assessed:

Efficacy

- At each of weeks 1, 2, 6 and 12 individually, the maximum of the Z-score of the internal diameters of the proximal right coronary artery or left anterior descending coronary artery.
- Any CAA defined using a stricter definition of a luminal internal diameter Z-score of ≥2.5 alone documented within the 12 weeks of trial follow-up
- Receipt of rescue treatment.
- Receipt of second dose of IVIG.
- Duration of fever after enrolment (time to temperature <38°C).
- Daily serum concentrations of CRP from days 1-5, and at 1 and 2 weeks after enrolment, and time to normalisation of CRP (≤10mg/L).
- Duration of hospitalisation.

Safety

- Serious adverse events including deaths.
- Grade 3 or 4 adverse events.
- Clinical adverse events of any grade judged related to IVIG, aspirin or corticosteroids given to treat KD.

Other outcome measures that will be assessed are:

- Changes in other laboratory parameters of inflammation (haemoglobin, white cell count, platelet count, ESR, albumin).
- Duration of corticosteroid therapy.

Docusign Envelope ID: D60D4999-E9B7-42C5-B5B0-35B5F15967A7

- Cumulative weight adjusted dose of prednisolone or methylprednisolone received.
- Proportion of patients who need to continue prednisolone at 2 mg/kg/day beyond day 5 (experimental group).
- Paediatric appropriate quality of life scores.
- Paediatric corticosteroid toxicity index (pGTI) to assess glucocorticoid related morbidity
- Incremental costs and cost-effectiveness (incorporating HRQL); budget impact.

2.4 Estimands

Estimand for effectiveness of treatment strategies

**Treatments**	The treatments being compared are immediate corticosteroids plus standard of care aspirin and IVIG, versus standard of care alone, allowing for rescue treatment.
**Population**	The population is children aged 30 days to 15 years with Kawasaki disease as defined by the inclusion/exclusion criteria in section 3.1.
**Endpoint**	Any CAA within 12 weeks of randomisation.
**Population-level summary measure**	Risk difference
**Strategy for intercurrent events**	
**Receipt of rescue treatment**	Treatment policy
**Any deviation from randomised strategy, including dose or duration of treatment**	Treatment policy
**Death without prior CAA**	Hypothetical

Docusign Envelope ID: D60D4999-E9B7-42C5-B5B0-35B5F15967A7

Estimand for direct efficacy of corticosteroids

**Treatments**	The treatments being compared are immediate corticosteroids plus standard of care aspirin and IVIG, vs standard of care alone, with no rescue treatment.
**Population**	The population is children aged 30 days to 15 years with Kawasaki disease as defined by the inclusion/exclusion criteria in section 3.1.
**Endpoint**	An average estimate across weeks 1, 2, and 6 of the maximum of the Z-score of the internal diameters of the proximal right coronary artery or left anterior descending coronary artery
**Population-level summary measure**	Mean difference
**Strategy for intercurrent events**	
**Receipt of rescue treatment**	Hypothetical
**Any deviation from randomised strategy, including dose or duration of treatment**	Treatment policy
**Death prior to rescue treatment**	Hypothetical

2.5 Sample size

Binary CAA primary endpoint

The estimated sample size of 262 children/adolescents provides 80% power to detect a reduction in CAA from 20% to 8% (two-sided alpha=0.05). The sample size calculation assumes that this endpoint can be completely ascertained, ie will not be missing for any child. Given its severity, and the severity of the condition, meaning children are closely monitored, including with echocardiography, this is judged a reasonable assumption and agrees with clinical experience.

Maximum Z-score primary endpoint

At any time point, 262 children/adolescents provides >80% power to detect changes in the maximum coronary artery Z-score of 0.4 times the standard deviation (two-sided alpha=0.05), assuming 13% children/adolescents may have missing values. There are no data to inform what effect estimate could be anticipated on continuous Z-scores, and therefore this effect size is pragmatic, based on the sample size for the binary CAA endpoint above.

Further details regarding the sample size calculation and justification for control group event rate is in the protocol Section 9.3.

2.6 Interim analyses

An independent Data Monitoring Committee (DMC) will be formed. Reports to the DMC will be produced by the MRC CTU at UCL statisticians. The DMC will be the only group which sees the

Docusign Envelope ID: D60D4999-E9B7-42C5-B5B0-35B5F15967A7

confidential, accumulating data for the trial separately by randomised group. The DMC will review trial data on recruitment, baseline characteristics, safety, adherence to randomised strategies and efficacy, as well as considering findings from any other relevant studies. Accumulating pooled information relating to recruitment, baseline characteristics, follow-up and compliance with protocol will also be presented in a separate report available to the TSC and TMG. Total numbers of CAA events for the primary outcome measure and other outcome measures may be presented, at the discretion of the independent DMC. The TMG may request any other pooled information they consider necessary for the good management of the trial be included in the Open Report; the independent DMC will decide whether or not such data may be released.

The first DMC is planned to take place within 12 months of the trial opening; the frequency of subsequent meetings will be determined by the DMC. It is anticipated that the DMC will initially consider the data highlighted in bold in this statistical analysis plan, summarised by arm. The DMC may request additional tables pre-specified in the SAP, or new analyses, at any time as required for decisions on stopping, modifying or continuing the trial. Data anticipated to be included in the Open Report (not by randomised treatment) are clearly indicated in the text of this statistical analysis plan.

The DMC can recommend premature closure or reporting of the trial, or that recruitment to any research group be discontinued or modified. Further details of DMC functioning, and the procedures for interim analysis and monitoring are provided in the DMC Charter.

Data not considered by the DMC will be considered in the final analysis at the end of the trial. The results of this final analysis will be reported following the principle of the ICH E3 guidelines on the Structure and Content of Clinical Study Reports.

2.7 Stopping guidelines

The statistical stopping guideline for the trial is a Haybittle-Peto type rule based on the 99.9% confidence interval. At each review by the independent DMC, early stopping of the trial should be considered only if there is evidence beyond reasonable doubt (p-value < 0.001) of benefit on one or other of the co-primary endpoints. The independent DMC will also consider clinical criteria, other efficacy outcome(s) and safety outcomes in any decision about early stopping. Reasons will be recorded for disregarding a stopping guideline.

There are no stopping guidelines for futility because KD-CAAP is a pragmatic trial and all evidence regarding the potential benefits of corticosteroids adds to the evidence base, for example for future meta-analyses.

2.8 Final analysis

The final analysis will take place after all participants have completed their week 12 visit, or are known to have withdrawn, died or been lost to follow up.

3. TRIAL POPULATION

3.1 Selection of patients

Children/adolescents will be considered eligible for enrolment in this trial if they fulfil all the inclusion criteria and none of the exclusion criteria as defined below.

Patient inclusion criteria

1. Aged 30 days (post-natal age) to 15 years inclusive, and below the country-specific age of consent for the duration of the trial
2. KD defined in at least one of the three following ways
   (a) as per American Heart Association (AHA) criteria: namely fever for at least 5 days in addition to 4 of the following 5 clinical criteria:
      i. bilateral non purulent conjunctivitis
      ii. cervical lymphadenopathy
      iii. polymorphous skin rash
      iv. changes in lips or mucosa (strawberry tongue, red cracked lips, diffuse erythematous oropharynx)
      v. extremity changes (erythema, oedema of palms and soles in initial phase, and at convalescent stage skin peeling)
   (b) OR less than 5 days of fever but all 5 clinical criteria above
   (c) OR incomplete KD cases, as per a modified* AHA definition, namely:
      i. children/adolescents (>1 year old) with fever greater than or equal to 5 days AND at least 2 other compatible clinical criteria as listed above; OR infants ≤ 1 year old with fever greater than or equal to 7 days without other explanation;
3. AND for both age groups
      i. CRP ≥30 mg/L or erythrocyte sedimentation rate (ESR) ≥40 mm/hr (or both)
4. AND for both age groups
      i. EITHER the presence of any 3 or more of: anaemia for age (haemoglobin < lower limit of normal reference range for local laboratory) platelet count ≥450 $x10^9$/L or <140 $x10^9$/L; albumin <30 g/L; elevated ALT (> upper limit of normal reference range for local laboratory); white cell count ≥15 $x10^9$/L; urine ≥10 white blood cells per high power field
      ii. OR abnormal echocardiogram compatible with KD but without established CAA, with ≥ 3 of the following suggestive features: decreased left ventricular function, mitral regurgitation, pericardial effusion, or dilated but non-aneurysmal coronary arteries (internal diameter 2≤Z<2.5; and not meeting the exclusion criteria for aneurysmal change as defined below).
5. Written informed consent from appropriate legal representative(s), and assent from patients who have not reached the age of consent and will not reach the age of consent for the duration of the trial in the participating country, but are judged to have capacity for this (depending on both age and acuity of illness)

*This definition of incomplete KD is modified from the AHA definition by firstly, the exclusion of aneurysmal coronary artery changes as the sole echo finding, since this is an exclusion criterion for KD-CAAP, and secondly the inclusion of low platelet count as well as high platelet count, as highlighted in recent European consensus SHARE guideline.[1]

Docusign Envelope ID: D60D4999-E9B7-42C5-B5B0-35B5F15967A7

Patients with KD can still be included in KD-CAAP if they have started IVIG treatment, as long as they are randomised no more than 24 hours after the IVIG infusion is initiated.

Test results must be from tests done on the calendar day of randomisation or the day before.

Patient exclusion criteria

Disease-related exclusions:

1. This diagnosis is a second or further episode of KD.
2. Already established CAA at screening.
3. Severe Congestive Heart Failure or cardiogenic shock defined as the presence of hypotension and shock requiring the initiation of volume expanders.
4. Known congenital coronary artery abnormality that would impair assessment of the primary endpoint.
5. Suspected macrophage activation syndrome.

Exclusions related to medications:

6. Started IVIG more than 24 hours prior to randomisation.
7. Known hypersensitivity to prednisolone or methylprednisolone, or known phenylketonuria to aspartame used in a formulation in an infant less than 12 weeks.
8. Current oral, intravenous or intramuscular corticosteroid treatment for more than 3 days in previous 7 days prior to randomisation.
9. History of previous severe reaction to any human immune globulin preparation.
10. [Germany only] Known contraindication to the study medication
11. [Germany only] pGFR <30 ml/min/1.73 m^2 (using the locally derived Haycock-Schwartz calculation)

Exclusions related to general health or other issues:

12. Active varicella zoster virus or influenza infection; or known exposure to a case of varicella within the previous 21 days prior to randomisation if known to be non-immune.
13. Co-enrolment in another study/trial of an investigative medicinal product.
14. Pregnant and/or breastfeeding adolescents.
15. [Germany only] females of child-bearing potential not willing to use highly effective contraception during participation in the study (refer to section **Error! Reference source not found.** for highly effective contraception methods).
16. [Germany only] Body weight <5kg

Disease-related exclusions relate to those (rare) patients who already have severe fulminant inflammation and/or shock when they are diagnosed with KD, in whom recent European consensus suggests corticosteroids and/or other immunosuppression are required. Such exceptional cases represent a small minority and therefore will not substantial impact on recruitment targets.

4. DATA

4.1 CRF forms and variables

Full details of data collection and timing are described in the trial protocol. A copy of the CRFs are presented in the Trial Master File. Details of the variables are presented within the metadata

which forms part of the Trial Master File. Details of the data management procedures are available in the Data Management Plan.

4.2 Management of datasets

- For an analysis for which a database lock is performed, the Trial Statistician will be responsible for defining when the data are clean and ready for database lock.
- For all analyses, datasets of all data stored in the database will be filed out from CACTUS. This will act as the frozen dataset.
- For interim analyses, new data can continue to be entered onto the CACTUS database. If a database lock is not performed and any outstanding data queries are resolved during the analysis that relate to data in the frozen dataset (e.g. problems that are found during analysis or amended CRFs that are data entered post-freeze), the data should be changed at the start of the set of analysis programs using an auditable statistical program, separate from all other programs (by the Trial/Delegated Statistician). The main CACTUS database will be amended in parallel.

For the final analysis the Trial Statistician will be responsible for defining when the data are clean and ready for database lock in the Data Management Plan.

4.3 Data verification

Data verification, consistency and range checks will have been performed by the MRC CTU at UCL, as well as checks for missing data (copies can be found in the Trial Master File). Additional range, consistency and missing data checks will be performed, as appropriate, when the analysis is performed (and when the datasets for analysis are constructed). All variables will be examined for unusual, outlying, unlabelled or inconsistent values.

Any problems with trial data will be queried with the Trial Managers, Data Managers, or statisticians, as appropriate. For interim analyses, if possible, data queries will be resolved and amended as above, although it is accepted that due to administrative reasons and data availability a small number of problems will continue to exist.

5. DERIVATION OF DATA TO BE ANALYSED

5.1 Definitions

Time

For time to event analyses time will be measured from randomisation.

Definition of baseline

Baseline values for all measurements will be those recorded on the baseline form, and the lab results form and the echocardiogram form at D0/screening (taken on the calendar day of randomisation or the day before). Lab results recorded on the screening form may be taken as baseline where data is not available on lab results form, but the lab results form will be the primary source of baseline data.

Definition of nominal day/week for echocardiograms, laboratory measurements, and other clinical parameters

Docusign Envelope ID: D60D4999-E9B7-42C5-B5B0-35B5F15967A7

Analyses of measurements for days 1 to 5 will be defined based on the visit day reported on the follow up form. For visits at weeks 1, 2, 6 and 12 the closest available measurement to that timepoint will be used. If there are two measurements that are equally close to the timepoint, the earliest measurement will be used.

Echocardiogram data

For the final analysis all echocardiogram data will be taken from the central review of echocardiogram (Form 11b). For interim analyses the values from the research site echocardiogram (Form 11a) may be used if the central review has not been completed yet, with a sensitivity analysis using only data that has been centrally reviewed. The numbers of results taken from central vs research site will be described.

Date of receiving rescue treatment

A patient will be defined as having received rescue treatment on the earliest date where a medication is recorded on the trial medication form, and 'Given as rescue medication' is answered Yes.

A second dose of IVIG given on day 2 or continuing oral prednisolone on day 5 following the treatment plan will not be considered rescue treatment.

Counting of events

SAEs will be analysed as episodes, with all components of the same clinical SAE presented as one episode. Analyses of grade 3 or 4 AEs, and clinical adverse events related to IVIG, aspirin or corticosteroids, will consider each component as separate events.

Death from an unknown cause will be analysed as a grade 4 event. Where cause of death is known and is itself a grade 4 event, the death will not be counted as a separate event to avoid double counting. If the cause of death is a grade 3 (or lower) event, it will be upgraded to a grade 4 event because it has led to death and counted as above.

5.2 Data transformations and coding

Free text

Free text fields will be categorised based on self-evident corrections, e.g. spelling. Adverse events and hospitalisations will be coded consistently in consultation with the Chief Investigator.

Continuous measures

Normality of all continuous measures and their change from baseline will be assessed using the Shapiro-Wilk test. For measures with only positive values, Box-Cox transformations of the original absolute measurements will be used in the case of gross ($p<0.0001$) deviations (these include log where this is most appropriate, but also allow other transformations such as square root). As z-scores have negative values Box-Cox transformations cannot be applied, so an appropriate alternate transformation will be performed, such as adding a constant prior to log transformation. Continuous measures will be truncated at the 1st and 99th percentile before analysis; specifically any value above the 99th percentile will be set to the 99th percentile and any values below the 1st percentile will be set to the 1st percentile.

Docusign Envelope ID: D60D4999-E9B7-42C5-B5B0-35B5F15967A7

Dose of corticosteroids

For analyses of the dose of corticosteroids, dose of methylprednisolone will be converted to the equivalent dose of prednisolone by multiplying by 1.25.

6. ANALYSIS PRINCIPLES

All summaries and analyses will be produced using Stata (updated and validated).

6.1 Statistical significance and p-values

The two endpoints will be considered separately, each with a nominal 0.05 level of significance. The overall type I error will depend on the correlation between the two effect estimates which is unknown (no data available to estimate this), but will be estimated using bootstrapping at the final analysis.

For other comparisons unless otherwise specified the two-sided alpha is 0.05 and no formal adjustment for multiple testing will be made. 95% confidence intervals will be reported. All significance tests will be interpreted in the context of the total number of comparisons performed.

For safety analyses, p-values will be produced for any serious, any grade 3 and 4, and any clinical adverse events related to corticosteroids, IVIG or aspirin (secondary outcomes). P-values will also be produced for each AE type. However, these will only be used as a flagging device to signal potential risk (adjustment for multiplicity is counterproductive for considerations of safety, according to EMEA points to consider on multiplicity issues in clinical trials).

6.2 Analysis populations

The primary analysis population is intention-to-treat, including all randomised patients, regardless of treatment received (using inverse probability weighting to adjust for rescue treatment for the efficacy co-primary endpoint).

Analysis will include all randomised patients with the exception of any patients not consented or randomised in error. Randomisation in error will be judged by the patient not being prescribed IVIG and aspirin, i.e. error identified immediately following randomisation and no drugs for Kawasaki disease ever given to the patient, and patient not being followed up. All other patients will be analysed according to the study group to which they were randomised.

6.3 Protection from bias

To counteract the possibility of bias, objective outcome measures have been chosen as much as possible. The primary endpoint (CAA and their Z-scores) will be assessed by locally trained echocardiographers/cardiologists and reviewed centrally by at least one of two independent echocardiographers blinded to randomised allocation. Standard operating procedures for echocardiography interpretation will be agreed prior to the start of the trial and disseminated to local recruiting sites.

Receipt of rescue medication/second dose of IVIG and drug related clinical adverse events (AEs) are the only secondary outcome measures where there is substantial risk of subjectivity. Bias

in terms of receipt of rescue medication/second dose of IVIG will be minimised by setting clear criteria in the protocol for management of rescue treatment; however, clinicians may always choose to use these outside the protocol if they judge that this is in the best interests of the child, so bias can never be completely excluded. In terms of drug-related clinical adverse events, the protocol contains clear criteria for assessing relatedness according to five categories (Section 7.3.1.C, Table 8).

Every effort will be made to minimise loss to follow up and to ascertain outcomes completely thus avoiding bias from differential ascertainment between the randomised groups; given the disease severity this is anticipated to be minimal.

6.4 Missing data

Missing data for the primary outcomes

If missing data or losses to follow up are less than 10% of participants then analysis of primary outcomes will use observed data only. If there is missing data in >10%, for the binary endpoint multiple imputation (MI) by chained equations will be used to adjust for this. MI will be performed separately for each randomised group using logistic regression. The imputation model will include age, sex, CRP and temperature at screening, baseline z-score (if available) and country. Multiple imputation is an accepted approach to increase power where data are missing at random (depend on observed covariates, not the unobserved outcome itself). The imputation model includes the key covariates which are hypothesised to be most strongly associated with the outcome. If there is missing data for the continuous endpoint in >10% of participants, additional probability weights will be used to adjust for this. The missingness weights will be estimated using the same predictors as above in an initial approach; as the mechanisms underlying missingness are completely unknown, data exploration will be conducted to identify whether there are other important predictors. Weights for missingness and receipt of rescue treatment will be multiplied together, as in causal analysis approaches such as marginal structural models.

Reasons for missing data will be explored descriptively by arm.

Other missing data

Other analyses will be based on observed data only; ie will assume data are Missing At Random.

7. STATISTICAL ANALYSIS

Primary analysis will adjust for randomisation stratification factors, secondary analyses will be unadjusted. Adjusting for randomisation stratification factors will incorporate up to 14 additional parameters. In the case of model non-convergences or perfect prediction, restricted models will be fitted adjusting for the randomisation stratification factors in the following priority order: age, sex, country.

7.1 Recruitment

- **Total screened and randomised by country and centre, with dates of first and latest randomisation**
- **Randomisation by strata**

Docusign Envelope ID: D60D4999-E9B7-42C5-B5B0-35B5F15967A7

- **Eligibility: number and reasons for any ineligibilities**

These data will also be included in the Open Report (not by randomised treatment).

7.2 Protocol deviations

Protocol deviations are defined in the Trial Master File and a list of all protocol deviations is kept in the Protocol Deviation Log.

- Protocol deviations: n (%) critical, major, minor

7.3 Baseline characteristics

For interim analyses, tabulations will be produced overall (i.e. not by randomised treatment). Tabulations will also be produced by randomised group, but only included in the report for variables with a difference between the randomised groups with p-value ≤ 0.05 (used as a flagging device for imbalance that could affect findings), with p-values from rank-sum test for continuous variables, and from chi-square tests for categorical variables or Fisher's exact test if cell values are small. For the final analysis, tabulations by randomised group will be included in the report.

- **Sex: n (%) male, female**
- **Age at last birthday: median (IQR), range, distribution into categories <1 year, 1-5 years, 6-10 years, ≥11 years**
- **Country: n (%) in each country**
- **Ethnicity: n (%) in categories; any white background, Asian, Black, mixed/multiple ethnic groups, unknown, other**
- **Type of KD: n (%) complete, incomplete**
- **Duration of fever at randomisation: median (IQR)**
- **Clinical criteria: n (%) with bilateral non purulent conjunctivitis, cervical lymphadenopathy, polymorphous skin rash, changes in lips or mucosa, extremity changes**
- Clinical examination: median (IQR) height/length, weight, heart rate, systolic blood pressure, diastolic blood pressure, maximum temperature on day of visit
- Significant medical history: n (%)
- **CRP at D0/screening: n (%) with result, median (IQR)**
- Biochemistry at D0/screening: n (%) with result, median (IQR) sodium, potassium, blood urea, creatinine, calcium, albumin, phosphate, glucose, serum bilirubin, AST, ALT, ALP, LDH
- **Haematology at D0/screening: n (%) with result, median (IQR) haemoglobin, white cell count,** neutrophil count, lymphocyte count, **platelet count**, MCV, **ESR**
- Urinalysis at D0/screening: n (%) normal, abnormal, clinically significant, distribution in categories 0, +, ++, +++, ++++; urine-protein (dipstick), urine-protein (laboratory analysis), urine-glucose
- Urine-protein (laboratory analysis) at D0/screening: n (%) with result, median (IQR), n (%) normal, abnormal, clinically significant
- **Echocardiogram at D0/screening: n (%) with result, median (IQR) maximum z-score**

7.4 Description of follow-up

For interim analyses, tabulations will be produced overall (i.e. not by randomised treatment). Tabulations will also be produced by randomised group, but only included in the report for variables with a difference between the randomised groups with p-value ≤ 0.05 (used as a flagging device for imbalance that could affect findings), with p-values from chi-square tests or Fisher's exact test if cell values are small. For the final analysis, tabulations by randomised group will be included in the report. For interim analyses a visit will be considered expected if the visit is due before the date of database lock or database extract.

- **Visits considered complete: number of visits expected, n (% of expected) visit complete (defined as attended or died before the visit took place)**
- **Status at each visit: n (% of expected) visit done within window, visit completed outside window, lost to follow up/withdrawn, died, missed visit; D1, D2, D3, D4, D5, W1, W2, W6, W12.**
- **Echocardiogram: n (%) performed, not performed, lost to follow up, died before visit, withdrawn; n (% of performed) central review complete, all data for primary endpoints complete; W1, W2, W6, W12**
- **Echocardiogram missing data plot: patterns of missing scans, scans missing 1 or more data item, scans not clinically reviewed for W1-W12**
- **CRP missing data plot: patterns of missing CRP results for D1-W2**

7.5 Result of Day 2 and Day 5 assessments

- Day 2 axillary temperature: median (IQR)
- Day 2 CRP: median (IQR)
- **Day 2 treatment assessment outcome: n (%) meeting each criteria (temperature <38°C and CRP≤10mg/L; temperature <38°C and CRP>10mg/L but ≤50% of baseline; temperature <38°C and CRP>10mg/L and still >50% of baseline; temperature ≥38°C and CRP≤10mg/L; temperature ≥38°C and CRP>10mg/L but ≤50% of baseline; temperature ≥38°C and CRP>10mg/L and still >50% of baseline)**
- **IVIG given at day 2: n (%) yes, following treatment plan; yes, not following treatment plan; no, following treatment plan; no, not following treatment plan; received rescue treatment prior to day 2**
- Day 5 axillary temperature: median (IQR)
- Day 5 CRP: median (IQR)
- **Day 5 treatment assessment outcome: n (%) meeting each criteria (temperature <38° and CRP≤10mg/L; temperature <38° and CRP>10mg/L; temperature ≥38°C and CRP≤10mg/L; temperature ≥38°C and CRP>10mg/L)**
- **Rescue treatment given at day 5: n (%) yes, following treatment plan; yes, not following treatment plan; no, following treatment plan; no, not following treatment plan; received rescue treatment prior to day 5**

7.6 Treatment details

Tabulations by randomised group will be produced as follows.

- **Received IVIG: n (%) yes, no**
- **Started IVIG before randomisation: n (%) yes, no**
- **Time from starting IVIG to randomisation, for those who started before: median (IQR) hours**

- **Dose of IVIG prescribed at or before randomisation: median (IQR) g/kg/day**
- **Received second dose of IVIG: n (%) yes, no**
- **Time to second dose of IVIG from randomisation: median (IQR) days**
- **Time from first to second dose of IVIG: median (IQR) hours**
- **Second dose of IVIG: median (IQR) of those who received second dose at day 2**
- **Received aspirin: n (%) yes, no**
- **Dose of aspirin prescribed at randomisation: median (IQR) mg/kg/day**
- **Time to aspirin dose reduction: median (IQR) days**
- **Received corticosteroids: n (%) yes, no**
- **Time to first dose of corticosteroid: median (IQR) days**
- **First dose of corticosteroids: median (IQR) mg/kg/day**
- **Time to first corticosteroid dose reduction: median (IQR) days**
- Dose of corticosteroids after first dose reduction: median (IQR) mg/kg/day
- Time to second corticosteroid dose reduction: median (IQR) days
- Dose of corticosteroids after second dose reduction: median (IQR) mg/kg/day
- **Rescue treatment given: n (%) yes, no**
- **Time from randomisation to starting rescue treatment: median (IQR) days if rescue treatment given**
- **Type of rescue treatment: n (%) retreatment with IVIG, IV methylprednisolone, starting oral prednisolone, continuing oral prednisolone, infliximab, ciclosporin, IL-1 blockade therapy, other**
- **Reasons for rescue treatment: n (%) meeting day 5 criteria, clinician decision at a different time**

7.7 Efficacy analyses

7.7.1 Primary outcome measures

- **Any CAA documented within the 12 weeks of trial follow-up**

Main analysis

The difference in proportions will be compared by calculating a risk difference and 95% confidence interval obtained from marginal effects after a logistic regression, adjusted for randomisation stratification factors in the primary analysis. In the case of non-convergence alternative methods will be used to estimate the CI, such as the Newcombe method. Secondary analysis will be unadjusted. If there are fewer than 5 cases of CAA in either treatment arm, or CAA is observed in less than 5% of participants, Fisher's exact test will be used to test for differences between arms.

Deaths not resulting from a CAA are not expected to occur in the study. If there are any deaths that were not preceded by a CAA, the primary analysis will exclude these deaths and a sensitivity analysis will be conducted which includes them.

In some cases, a scan taken on day 0 could show a CAA that was not known or not identified at the time of randomisation. For the primary analysis, these will be counted as a CAA at day 1. A secondary analysis will exclude these participants.

The number of outcomes based on central vs research site assessment will be tabulated by randomised group.

Docusign Envelope ID: D60D4999-E9B7-42C5-B5B0-35B5F15967A7

The number of CAAs meeting each criteria in the definition will be tabulated. The visit at which CAA was detected will also be presented.

Bayesian secondary analysis

Analysis of the binary primary outcome will also be conducted in a Bayesian framework. If the ultimate achieved sample size is <80% of the planned 262, then this analysis will be considered the primary analysis.

The difference in proportions between treatment groups and 95% credible intervals will be compared by first performing logistic regression using Bayesian methods and then estimating the risk difference by the average difference in risk between the treatment arms. This can be performed by monitoring Y[i,1] and Y[i,0] for each individual i and therefore the average of Y[i,1] – Y[i,0], via Markov chain Monte Carlo methods. The posterior probability of superiority of corticosteroids (ie risk difference < 0%) will be calculated.

The prior for the rate of CAAs in the control group will be beta(0.44, 1.76) which has mean 0.2 and variance 0.05. The prior for the treatment comparison will be the uninformative prior N(0, 10000) on the logit scale. Sensitivity analyses will be carried out with the enthusiastic prior N(-1.0560527, 0.29030801), equivalent to the hypothesised -12% risk difference, and sceptical prior N(0, 0.29030801).

- **An average estimate across weeks 1, 2, and 6 of the maximum of the Z-score of the internal diameters of the proximal right coronary artery or left anterior descending coronary artery, adjusting for rescue treatment**

Generalised estimating equations (GEEs) with an independent correlation structure will be used for a global test of differences in maximum Z-score between treatment arms, since each child will contribute multiple measurements to the analysis. This approach incorporates errors on each individual measurement, rather than simpler approaches taking the mean within child across all measurements and analysing that as a single outcome per child. The independent correlation structure adjusts the variance for the fact that each child contributes multiple measurements to the analysis, but without making strong assumptions about the correlation between measurements. A sensitivity analysis will test an unstructured correlation matrix if this will converge.

Children who received rescue treatment will be censored for this analysis at the time of starting rescue treatment, and inverse probability of (change from) treatment weighting (IPTW) will be used to adjust for use of rescue treatment, following standard causal approaches such as marginal structural models and methods to adjust for non-compliance. If the date of starting rescue treatment is the same as the date of an echocardiogram, data from that scan will not be censored, under the assumption that it is most likely rescue treatment was started as a result of the scan.

This analysis will also adjust for baseline Z-score using three categories: missing (as not all patients will have an echocardiogram at baseline), below median and above median. The primary analysis will adjust for randomisation stratification factors, the secondary analysis will be unadjusted for these factors. Weights will be calculated using logistic regression to determine the probability of receiving rescue treatment based on baseline characteristics (age, CRP and temperature at screening, baseline Z-score (if available, otherwise using a "missing" group as above), country). The regression will also include prior CRP and temperature values to adjust for post-baseline differences. For week 1 these will be the day 2 and day 5 values, if rescue treatment had not started before these dates. If rescue treatment had started before these

dates, values will be carried forward from the last value before rescue treatment (including on the day rescue treatment started). For later weeks CRP and temperature from the two most recent scheduled visits will be used. If there are missing values at these time points a missing indicator will be included, and the value imputed as the median. If this model does not converge, then we will use last observation carried forward to enable probability weights to be calculated for all children.

Results will also be presented from the GEE with weights based on baseline data only, and the unweighted model.

It is expected that participants who died from KD would receive rescue treatment prior to death. In the circumstance of a death occurring without use of rescue treatment, a sensitivity analysis will censor this death and incorporate this censoring into the inverse probability weights (ie the weights will reflect rescue treatment or death). This would then estimate the efficacy of the intervention assuming that children stayed on it without switching to rescue medication or dying without switching.

Secondary analysis

A secondary analysis will use ordinal logistic GEE, adjusted for rescue treatment using weights as above, to analyse z-scores in the categories <2, ≥2 to <2.5, ≥2.5 to <5, ≥5 to <10, ≥10. The difference between arms will be presented as an odds ratio with 95% confidence interval.

7.7.2 Secondary outcome measures

- **At each of weeks 1, 2, 6 and 12 individually, the maximum of the Z-score of the internal diameters of the proximal right coronary artery or left anterior descending coronary artery.**

Average maximum Z-score, and difference between arms with 95% confidence intervals will be calculated at each timepoint using normal linear regression adjusting for baseline Z-scores in three strata: missing, below median and above median. Normality of each measure will be assessed using the Shapiro-Wilk test, and in the case gross departure from normality ($p<0.0001$), the regression will be performed on a transformed variable (using Stata lnskew as negative values for Z-scores means boxcox cannot be used). These analyses will adjust for use of rescue treatment using the same inverse probability of treatment weights as above, censoring any child who has switched to rescue treatment before each timepoint.

- **Any CAA defined using a stricter definition of a luminal internal diameter Z-score of ≥2.5 alone documented within the 12 weeks of trial follow-up**

- **Receipt of rescue treatment**

- **Receipt of second dose of IVIG**

For these three outcomes, the difference in proportions of children developing each outcome will be estimated by calculating marginal effects after a logistic regression to obtain risk difference and 95% confidence intervals. If there are fewer than 5 events in either treatment arm, or less than 5% of participants experience this event, an exact test will be used to test for differences between arms.

- **Duration of fever after enrolment (time to temperature <38°C).**

Time from randomisation to a recorded temperature below 38°C will be estimated using competing risks analysis, with death before achieving temperature of <38°C treated as a competing risk. Cumulative incidence curves (non-parametric, analogous to Kaplan-Meier curves but incorporating the fact that death means that fever resolution can never be observed) will be plotted by arm. Semi-parametric sub-hazard regression models analogous to Cox proportional hazards regression will be used to estimate a sub-hazard ratio and 95% confidence interval for the difference between arms.

- **Daily serum concentrations of CRP from days 1-5, and at 1 and 2 weeks after enrolment and time to normalisation of CRP (≤10mg/L)**

Normal linear regression adjusting for baseline CRP will be used to calculate mean difference and 95% confidence intervals between the treatment arms at each timepoint and generalised estimating equations with independent correlation structure used to provide an overall test of difference between randomised arms across all timepoints. Time from randomisation to CRP ≤10mg/L will be analysed competing risks analysis, with death before normalisation of CRP treated as a competing risk, and cumulative incidence curves will be plotted and difference between arms estimated as above.

- **Duration of hospitalisation**

Time from randomisation to discharge from hospital will be analysed using competing risks analysis with any deaths before discharge treated as a competing risk. Cumulative incidence curves will be plotted by arm and difference between arms estimated as above.

7.7.3 Other outcome measures

- **Changes in other laboratory parameters of inflammation (haemoglobin, white cell count, platelet count, ESR, albumin)**

Mean changes from baseline, and difference between arms, in laboratory parameters will be estimated at day 2, day 5, week 2 and week 6 using linear regression adjusting for baseline values. Generalised estimating equations with independent correlation structure will be used to provide an overall test of difference between randomised arms across all timepoints.

- **Duration of corticosteroid therapy**

Duration of corticosteroid therapy in the experimental group, and in those receiving corticosteroids in the control group, will be summarised by median (IQR).

- **Cumulative weight adjusted dose of corticosteroids received**

Cumulative weight adjusted dose of corticosteroids received in the experimental group, and in those receiving corticosteroids in the control group, will be summarised by median (IQR).

- **Proportion of patients who continue prednisolone at 2 mg/kg/day beyond day 5 (experimental group)**

The number and percentage of those who started prednisolone who continued with prednisolone at 2 mg/kg/day after day 5 will be presented.

7.8 Safety analyses

7.8.1 Secondary outcome measures

- **Serious adverse events including deaths**
- **Grade 3 or 4 adverse events**
- **Clinical adverse events of any grade judged related to IVIG, aspirin or corticosteroids**

The number (%) of children ever having an SAE, grade 3 or 4 AE, or a clinical AE related to IVIG, aspirin or corticosteroids, will be tabulated and compared across randomised groups with a chi-squared test. Given the number of children in the trial it is inevitable that power will be low to detect differences between arms, and therefore these p-values are indicative only, as a flagging device, and are not intended to be definitive. If any p-value is under 0.1, then a risk difference and 95% CI will be calculated for the difference between arms, using unadjusted logistic regression (given likely low numbers) and marginalised across covariates to give a mean.

Relationship of the above events to IVIG, aspirin, and corticosteroids will be tabulated across randomised groups. The total number of events will also be tabulated by SAE criteria (fatal, life threatening, cause or prolonged hospitalisation, persistent or significant disability, other) and randomisation group.

All SAEs will also be listed by treatment arm and trial number.

7.9 Other analyses

- Paediatric appropriate quality of life scores

Normal linear regression adjusting for baseline score will be used to compare between arms the change in the paediatric quality of life (PedsQL) total score at week 12. Mean changes and 95% confidence intervals by arm will be presented.

- Paediatric corticosteroid toxicity index (pGTI) to assess glucocorticoid related morbidity

The pGTI score for changes between week 0 and week 12 will be compared between arms using normal linear regression.

- Incremental costs and cost-effectiveness (incorporating HRQL); budget impact

Health economic analyses are not included in this Statistical Analysis Plan.

7.10 Subgroup analyses

Given the size of the trial, subgroup analyses are planned only by minimisation factors (excluding country), namely age (<1 vs ≥1 year) and gender. It is not known what percentages will fall into these different groups; depending on numbers (eg, under 20% in one subgroup), these may not be possible. Subgroup analyses will be performed for the primary outcomes only and will be based on tests of interaction, although the range of the 95% CI will be used to identify the potential for greater harm or greater benefit in any subgroup.

Exploratory subgroup analyses will also be conducted for baseline z-score and day of illness. For baseline z-score two categorisations will be considered: above median, below median, or missing; and <2.5, ≥2.5 or missing.

Subgroup analyses will be performed at the final analyses only.

8. DISSEMINATION OF RESULTS

Details of the publication policy are described in the trial protocol.

9. REFERENCES

1. de Graeff N, Groot N, Ozen S, Eleftheriou D, Avcin T, Bader-Meunier B, Dolezalova P, Feldman BM, Kone-Paut I, Lahdenne P *et al*: **European consensus-based recommendations for the diagnosis and treatment of Kawasaki disease - the SHARE initiative**. *Rheumatology (Oxford)* 2019, **58**(4):672-682.

Certificate Of Completion

Envelope Id: D60D4999-E9B7-42C5-B5B0-35B5F15967A7		Status: Completed
Subject: Complete with Docusign: KD CAAP Statistical Analysis Plan v5.0.docx		
Source Envelope:		
Document Pages: 24	Signatures: 4	Envelope Originator:
Certificate Pages: 5	Initials: 0	Roisin Connon
AutoNav: Enabled		90 High Holborn 2nd Floor London
EnvelopeId Stamping: Enabled		London, London WC1V 6LJ
Time Zone: (UTC) Dublin, Edinburgh, Lisbon, London		r.connon@ucl.ac.uk
		IP Address: 128.40.216.23

Record Tracking

Status: Original<br>22 November 2024 | 11:14	Holder: Roisin Connon<br>r.connon@ucl.ac.uk	Location: DocuSign

Signer Events	Signature	Timestamp
Ann Sarah Walker<br>rmjlasw@ucl.ac.uk<br>Security Level: Email, Account Authentication (Optional)	Signed by:<br>Ann Sarah Walker<br>A21A8226533E49A...<br><br>Signature Adoption: Pre-selected Style<br>Using IP Address: 86.129.162.100	Sent: 22 November 2024 | 11:15<br>Viewed: 22 November 2024 | 11:57<br>Signed: 22 November 2024 | 11:57
**Electronic Record and Signature Disclosure:**<br>Accepted: 18 May 2023 | 14:17<br>ID: 9ad929cc-a8ee-47b3-9d62-0b65b30a1dc0		
Despina Eleftheriou<br>d.eleftheriou@ucl.ac.uk<br>Professor of paediatric rheumatology<br>Security Level: Email, Account Authentication (Optional)	Signed by:<br>Despina Eleftheriou<br>BD2BDAFC2A364D6...<br><br>Signature Adoption: Pre-selected Style<br>Using IP Address: 144.82.8.238	Sent: 22 November 2024 | 11:15<br>Viewed: 22 November 2024 | 11:17<br>Signed: 22 November 2024 | 11:17
**Electronic Record and Signature Disclosure:**<br>Accepted: 22 November 2024 | 11:17<br>ID: 079e894c-cf46-4b38-a5b4-002c460e8049		
Paul Brogan<br>p.brogan@ucl.ac.uk<br>Security Level: Email, Account Authentication (Optional)	DocuSigned by:<br>Paul Brogan<br>9B9612DDBB3049F...<br><br>Signature Adoption: Pre-selected Style<br>Using IP Address: 82.36.101.42<br>Signed using mobile	Sent: 22 November 2024 | 11:15<br>Resent: 25 November 2024 | 16:52<br>Viewed: 25 November 2024 | 17:28<br>Signed: 25 November 2024 | 17:28
**Electronic Record and Signature Disclosure:**<br>Accepted: 07 September 2023 | 16:20<br>ID: 2f989e8d-98de-407c-80a1-068c120b3989		
Roisin Connon<br>r.connon@ucl.ac.uk<br>Statistician<br>University College London<br>Security Level: Email, Account Authentication (Optional)	DocuSigned by:<br>Roisin Connon<br>1671D28A21C9466...<br><br>Signature Adoption: Pre-selected Style<br>Using IP Address: 128.40.216.23	Sent: 22 November 2024 | 11:15<br>Viewed: 22 November 2024 | 11:16<br>Signed: 22 November 2024 | 11:16
**Electronic Record and Signature Disclosure:**<br>Not Offered via DocuSign		

In Person Signer Events	Signature	Timestamp

Editor Delivery Events	Status	Timestamp

Agent Delivery Events	Status	Timestamp

Intermediary Delivery Events	Status	Timestamp

Certified Delivery Events	Status	Timestamp

Carbon Copy Events	Status	Timestamp

Witness Events	Signature	Timestamp

Notary Events	Signature	Timestamp

Envelope Summary Events	Status	Timestamps
Envelope Sent	Hashed/Encrypted	22 November 2024 | 11:15
Certified Delivered	Security Checked	22 November 2024 | 11:16
Signing Complete	Security Checked	22 November 2024 | 11:16
Completed	Security Checked	25 November 2024 | 17:28

Payment Events	Status	Timestamps

Electronic Record and Signature Disclosure

ELECTRONIC RECORD AND SIGNATURE DISCLOSURE

From time to time, MRC Clinical Trials Unit at UCL (we, us or Company) may be required by law to provide to you certain written notices or disclosures. Described below are the terms and conditions for providing to you such notices and disclosures electronically through the DocuSign system. Please read the information below carefully and thoroughly, and if you can access this information electronically to your satisfaction and agree to this Electronic Record and Signature Disclosure (ERSD), please confirm your agreement by selecting the check-box next to 'I agree to use electronic records and signatures' before clicking 'CONTINUE' within the DocuSign system.

Getting paper copies

At any time, you may request from us a paper copy of any record provided or made available electronically to you by us. You will have the ability to download and print documents we send to you through the DocuSign system during and immediately after the signing session and, if you elect to create a DocuSign account, you may access the documents for a limited period of time (usually 30 days) after such documents are first sent to you. After such time, if you wish for us to send you paper copies of any such documents from our office to you, you will be charged a $0.00 per-page fee. You may request delivery of such paper copies from us by following the procedure described below.

Withdrawing your consent

If you decide to receive notices and disclosures from us electronically, you may at any time change your mind and tell us that thereafter you want to receive required notices and disclosures only in paper format. How you must inform us of your decision to receive future notices and disclosure in paper format and withdraw your consent to receive notices and disclosures electronically is described below.

Consequences of changing your mind

If you elect to receive required notices and disclosures only in paper format, it will slow the speed at which we can complete certain steps in transactions with you and delivering services to you because we will need first to send the required notices or disclosures to you in paper format, and then wait until we receive back from you your acknowledgment of your receipt of such paper notices or disclosures. Further, you will no longer be able to use the DocuSign system to receive required notices and consents electronically from us or to sign electronically documents from us.

All notices and disclosures will be sent to you electronically

Unless you tell us otherwise in accordance with the procedures described herein, we will provide electronically to you through the DocuSign system all required notices, disclosures, authorizations, acknowledgements, and other documents that are required to be provided or made available to you during the course of our relationship with you. To reduce the chance of you inadvertently not receiving any notice or disclosure, we prefer to provide all of the required notices and disclosures to you by the same method and to the same address that you have given us. Thus, you can receive all the disclosures and notices electronically or in paper format through the paper mail delivery system. If you do not agree with this process, please let us know as described below. Please also see the paragraph immediately above that describes the consequences of your electing not to receive delivery of the notices and disclosures electronically from us.

How to contact MRC Clinical Trials Unit at UCL:

You may contact us to let us know of your changes as to how we may contact you electronically, to request paper copies of certain information from us, and to withdraw your prior consent to receive notices and disclosures electronically as follows:
To contact us by email send messages to: s.assam@ucl.ac.uk

To advise MRC Clinical Trials Unit at UCL of your new email address

To let us know of a change in your email address where we should send notices and disclosures electronically to you, you must send an email message to us at s.assam@ucl.ac.uk and in the body of such request you must state: your previous email address, your new email address. We do not require any other information from you to change your email address.

If you created a DocuSign account, you may update it with your new email address through your account preferences.

To request paper copies from MRC Clinical Trials Unit at UCL

To request delivery from us of paper copies of the notices and disclosures previously provided by us to you electronically, you must send us an email to s.assam@ucl.ac.uk and in the body of such request you must state your email address, full name, mailing address, and telephone number. We will bill you for any fees at that time, if any.

To withdraw your consent with MRC Clinical Trials Unit at UCL

To inform us that you no longer wish to receive future notices and disclosures in electronic format you may:

i. decline to sign a document from within your signing session, and on the subsequent page, select the check-box indicating you wish to withdraw your consent, or you may;

ii. send us an email to s.assam@ucl.ac.uk and in the body of such request you must state your email, full name, mailing address, and telephone number. We do not need any other information from you to withdraw consent.. The consequences of your withdrawing consent for online documents will be that transactions may take a longer time to process..

Required hardware and software

The minimum system requirements for using the DocuSign system may change over time. The current system requirements are found here: https://support.docusign.com/guides/signer-guide-signing-system-requirements.

Acknowledging your access and consent to receive and sign documents electronically

To confirm to us that you can access this information electronically, which will be similar to other electronic notices and disclosures that we will provide to you, please confirm that you have read this ERSD, and (i) that you are able to print on paper or electronically save this ERSD for your future reference and access; or (ii) that you are able to email this ERSD to an email address where you will be able to print on paper or save it for your future reference and access. Further, if you consent to receiving notices and disclosures exclusively in electronic format as described herein, then select the check-box next to ‘I agree to use electronic records and signatures’ before clicking ‘CONTINUE’ within the DocuSign system.

By selecting the check-box next to ‘I agree to use electronic records and signatures’, you confirm that:

- You can access and read this Electronic Record and Signature Disclosure; and
- You can print on paper this Electronic Record and Signature Disclosure, or save or send this Electronic Record and Disclosure to a location where you can print it, for future reference and access; and
- Until or unless you notify MRC Clinical Trials Unit at UCL as described above, you consent to receive exclusively through electronic means all notices, disclosures, authorizations, acknowledgements, and other documents that are required to be provided or made available to you by MRC Clinical Trials Unit at UCL during the course of your relationship with MRC Clinical Trials Unit at UCL.
