# Supplementary material for: Multi-centre, randomised, open-label, blinded endpoint assessed, trial of corticosteroids plus intravenous immunoglobulin (IVIG) and aspirin, versus IVIG and aspirin for prevention of coronary artery aneurysms (CAA) in Kawasaki disease (KD): the KD-CAA prevention (KD-CAAP) trial
Source: eClinicalMedicine. 2026 Jul 13;97:104044. doi: 10.1016/j.eclinm.2026.104044 (PMC13382441; doi:10.1016/j.eclinm.2026.104044)
Supplement: Supplementary material [file mmc6.docx]

**Supplementary material Page**

**Table of contents**

**Other members of the trial team and committees 2**

**Supplementary methods 3**

**Supplementary tables 7**

**Supplementary figures 21**

**Other members of the trial team and committees**

Bjorn Cools^1^, Thomas Salaets^1^, Luc Bruyndonckx^2^,Mahmoud Zaqout^2,^ Daphné Christiaens^3^ ,Ilse Coomans^3^ , Karen Logghe^3^ , Kristof Vandekerckhove^3^ , Anneli Kõiv^4^, Galina Redkina^4^ Irja Lutsar^4^, Jana Lass^4^, Jelena Peuša^4^, Kristel Köbas^4^, Marika Metsoja^4^, Olga Šveikina^4^, Pille Kaires^4^, Saale Koonik^4^, Silje Kilk^4,^ Eveliina Riuttanen^5^, Hanna Väärälä^5^, Heidi Alanen^5^, Henna Jylhä^5^, Kaisa Ylänen^5^, Maria Pohjanpää^5^, Minna Mecklin^5^, Ninni Keränen^5^, Salla Kuusela^5^, Satu Vuolle^5^, Tuija Poutanen^5^, Camille Bertho^6^, Bellabes Ghezzoul^6^, Marion Audie^6^, Pierre-Marie Duboue^6^, Clément Brunet^6^, Jérôme Granel^6^, Marion Favier^6^, Olivier Richer^6^, Pascal Pillet^6 ,^ Jerome Harambat^6^, Maud Sordet^6^, Camille Berth^6^, Michael Fayon^6,^ Edoardo Marrani^7^, Ilaria Maccora^7^, Ilaria Pagnini^7^, Maria Vincenza Mastrolia^7^, Christian Schroer^8^, Jörg Heuser^8^, Marieke Vervoorn^8^, Paul De Klaver^8^,Annet Van Royen^9^, Erika Van Nieuwenhove^9^, Joris Van Montfrans^9^, Almudena Ortiz-Garrido^10^, Laura Martín-Pedraz^10^, Judith Sanchez-Manubens^11^, Sonia Carriqui^11^, Juan Manuel Mosquera^11^, Annika Jarting^12^, Felicia Nordenstam^12^, Kerstin Magnusson^12^ , Lilly-Ann Mohlkert^13^, Maria Berner^13 ,^ Deepthi Jyothish^14^, Faheem Patel^14^, Sakeena Samar^14^,Aubrey Cunnington^15^, Joseph Wacher^15^, Liz Whittaker^15^, Silvia Caroli^15^, Sobia Mustafa^15^, Leanne Taylor^16^, Lily Kirkpatrick^16^, Alex Jones^17,^ Jennie Moreton^17,^ Nicola Howell^17,^ Rebecca Beckley^17^, Sarah Hopton^17^,Claire McGinn^18^, Frank Casey^18^, Nicola McCay^18^, Samantha LaRoche^18,^ Jocelyn Nocerino^19^, Susanne Scott^19^,Charlotte Reigan^20,^ Alison Kent^21^, Zoe Whitehouse^21,^ Aimee-Louise Hamlin^22^, Anna Riddell^22^, Archana Bansal^22^, Luisa Teixeira^22^, Nicolene Plaatjies^22^, Nosheen Khalid^22^, Theresa Simangan^22,^ Donna McNaughton^23^, Jaclyn Keightley^23^ ,Leslie Armour^23^, Susanne Cathcart^23^, Serena Cruickshank-Hull^24,^ Lucinda Dawson^24^ ,Justine Cape^24^, Ellie Couch^24^, Rumena Begum^24^, Rebekka Choudhury^24^.

Affiliations:

1. Division of Pediatric Rheumatology, Department of Pediatrics, University Hospitals Leuven, Leuven, Belgium.

2. Universitair Ziekenhuis Antwerpen, Belgium

3. Universitair Ziekenhuis Gent, Belgium

4. Children´s Clinic of Tartu University Hospital, Department of Acute Infections, Estonia

5. Tampere Center for Child, Adolescent and Maternal Health Research, Faculty of Medicine and Health Technology, Tampere University and University Hospital, Tampere, Finland.

6. University Hospital of Bordeaux, Pole de Pediatrie, France

7. Rheumatology Unit, Meyer Childrens Hospital IRCCS, Firenze

8. MMC Veldhoven, Pediatrics, Netherlands

9. University Medical Center Utrecht, Pediatric Immunology and Rheumatology, Netherlands

10. Hospital Regional Universitario de Málaga, Spain

11. Hospital Sant Joan de Déu / Pediatric Rheumatology Department, Spain

12. Department of Women's and Children's Health, Karolinska Institutet, Sweden

13. Karolinska Institutet, Dept of Clinical Science and Education, Södersjukhuset, Sweden

14. Birmingham Women's and Children's NHS Foundation Trust, University of Birmingham, Birmingham, UK

15. Department of Infectious Disease - Faculty of Medicine, St Mary’s Hospital, UK

16. Department of Paediatrics, The Whittington Hospital, London, UK

17. Department of Paediatrics, University of Oxford, UK

18. Paediatric Cardiology, Royal Belfast Hospital for Sick Children, Belfast, Ireland

19. Paediatric Emergency Dept, Epsom & St Helier University Hospitals NHS Trust, U

20. Paediatric Infectious Disease and Immunology, Great North Children’s Hospital, Newcastle-upon-Tyne, UK

21. Paediatric cardiology, Evelina Children's Hospital, London, UK

22. Paediatric infectious diseases and immunology, Royal London Hospital, Barts Health NHS Trust, London, UK

23. Royal Hospital for sick children, Glasgow, UK

24. Great Ormond Street Hospital, London, UK

**Trial Steering Committee** (independent members): Bas Vastert (Chair), Mark Little, Laura Cove, Kate Armon

**Data Monitoring Committee**: Brian Feldman (Chair), Jane Burns, Toby Prevost.

**Supplementary Methods**

**Additional details on echocardiography**

There was a detailed manual of operations for echocardiography, included as part of investigator training before sites were opened. The maximal internal diameter between the endocardial borders was measured at 2-5 mm from the origin of the coronary artery, avoiding sites of branching. Coronary artery measurements were made using low-scale colour mapping with dual display to avoid erroneous measurement of cardiac veins or artefacts that resemble the coronary arteries. The left main, proximal and distal left anterior descending, circumflex, and proximal right coronary artery diameters were measured in parasternal short-axis views; the distal right coronary artery diameter was measured in apical 4-chamber view with posterior angulation; and the posterior descending coronary artery diameter was measured in parasternal long-axis views with rightward posterior angulation.

**Justification for criteria for non-response and additional IVIG doses**

Non-response is not just dependent on fever, but also systemic markers of inflammation. When the drive to systemic inflammation is truly switched off (and hepatic production of CRP is switched off), the half-life of CRP is roughly 18 hours. Thus, since fever is overall a poor biomarker for systemic inflammation, we also used CRP to monitor “response or non-response”. For some years European guidelines have indicated that a more appropriate therapeutic target is to aim for “zero fever and zero CRP” as highlighted in the SHARE guidelines.^1^ Patients with other autoinflammatory diseases can have severe ongoing systemic inflammation but may not have fever. So, we adopted an approach that acknowledged this. This in some ways also reflected the Japanese RAISE study protocol which also focused on CRP to guide dose reductions in prednisolone. In that study, when concentration of C-reactive protein normalised (≤5 mg/L), they tapered the prednisolone dose over 15 days in 5-day steps, from 2 mg/kg per day to 1 mg/kg per day to 0·5 mg/kg per day. They also did laboratory testing two to three times per week until concentration of C-reactive protein had decreased to 5 mg/L or less. So, there is an evolving understanding that just relying on fever is too crude to confidently gauge systemic inflammation.

**Pediatric Glucocorticoid Toxicity Index (pGTI)**

This tool examines 10 weighted toxicity domains (plus subdomains) that capture common, important, and dynamic glucocorticoid (GC) adverse effects in children.^2^ These domains measure change over time and contribute to the overall toxicity score.

Below is a brief summary of the domains included in the full tool:

1. Change in Body Weight (BMI)

Assesses increases or decreases in BMI units. Large increases (≥5 BMI units) carry high weight due to their clinical impact.

2. Growth Velocity

Evaluates changes in height Z-score (unique to paediatrics). A decrease >0.5 in Z-score reflects GC-related growth suppression.

3. Glucose Metabolism

Assesses:

- HbA1c changes
- Changes in diabetes medication

Captures steroid-induced hyperglycemia or diabetes.

4. Blood Pressure

Evaluates:

- Changes in blood pressure levels
- Need for antihypertensive medication

Accounts for paediatric age- and sex-adjusted norms.

5. Hyperlipidemia

Measures changes in LDL cholesterol and lipid-lowering therapy.

6. Bone Mineral Density (BMD)

Assesses changes in bone density Z-scores, reflecting GC-induced bone loss.

7. Steroid Myopathy

Measures muscle weakness severity (minor to moderate), including functional impact.

8. Skin Toxicity (Subdomains)

Includes:

- Acne
- Hirsutism
- Striae/atrophy
- Easy bruising

These are graded by severity and change over time.

9. Neuropsychiatric Toxicity (Subdomains)

Includes:

- Sleep disturbance
- Mood disturbance (major mood disturbance carries the highest weight overall)
- Cognitive impairment

This domain captures both behavioral and neurodevelopmental effects.

10. Infections

Assesses:

- Frequency and severity
- Particularly Grade 3–4 infections (highly weighted)

**Additional Component: Damage Checklist (Unweighted)**

The tool also includes a Damage Checklist (separate from scoring), capturing serious but typically irreversible GC effects:

- Osteonecrosis
- Cataracts
- Tendon rupture
- Severe psychiatric complications
- Hypertensive emergencies
- Diabetic complications

These do not contribute to the quantitative score because they are less likely to change with GC dose adjustments

In summary the pGTI evaluates multi-system glucocorticoid toxicity across metabolic, cardiovascular, musculoskeletal, dermatologic, neuropsychiatric, infectious, and growth domains, using weighted scoring to measure improvement or worsening over time in paediatric patients.

In KDCAAP the following domains were not assessed: glucose metabolism, hyperlipidaemia and bone mineral density, due to excessive blood volume sampling in young children; and need for DEXA scanning to assess bone density (expensive, not practical in KD-CAAP, and limited normative data in young children) respectively.

**Additional details of sample size**

The trial was powered to detect a reduction in CAA from 20% to 8%, requiring 262 participants (80% power, α=0.05). There were no data to inform what effect estimate could be anticipated on the continuous Z-score co-primary endpoint, nor its standard deviation, and therefore the effect size for this comparison was pragmatically determined, based on the sample size for the binary CAA endpoint. Specifically, 262 children/adolescents provided >80% power to detect changes in the maximum coronary artery Z-score of 0.4 times the standard deviation (two-sided alpha=0.05), assuming 13% children/adolescents had missing values. The protocol specified that the two co-primary endpoints were to be considered separately, each with a nominal 0.05 level of significance, reflecting the fact that KD is a relatively rare disease in which it is important to generate randomised unbiased evidence and consider its totality.^3^ The two co-primary endpoints were designed to address different aspects (efficacy vs effectiveness) on different outcomes, comprising a highly clinically relevant effectiveness (intention-to-treat) binary endpoint with lower intrinsic power (due to its binary nature) vs a potentially less relevant continuous efficacy endpoint (adjusting for rescue treatment using inverse probability of (rescue) treatment weights (IPTW)) with higher power (due to its continuous nature). Therefore, it was considered entirely possible to reach significance on one and not the other.

During the trial, given the challenges of COVID-19 and the fixed funding end date, it became apparent that the trial would not be able to recruit to its originally targeted sample size. Again, given the relative rareness of KD, and the importance of conducting randomised trials in small populations, after independent statistical review by the funder and well before the end of the trial, it was agreed that the primary frequentist analysis of the co-primary outcome CAA rate should be supplemented by a Bayesian analysis which provides much richer information for clinicians interpreting the results of the randomised comparisons, using uninformative (primary; analogous to the frequentist analysis), enthusiastic and sceptical priors (supplementary figure 2).^4^ The Statistical Analysis Plan (SAP) was updated to reflect this agreement with the funder. The revised SAP stated that if the final sample size was <80% target, these Bayesian analyses were considered the primary analysis. The Bayesian primary analysis were planned to be adjusted for age, sex and country as specified in the revised SAP, as for the frequentist analyses. The final analyses were not adjusted for country due to large numbers of countries and countries with no events in one or both randomised groups.

The prior for the rate of CAAs in the control group was N (-1.39, 0.66) on the logit scale. The prior for the primary treatment comparison was the uninformative prior N (0, 10000) on the logit scale. Sensitivity analyses used an enthusiastic prior N (-1.0560527, 0.29030801) (equivalent to the hypothesised -12% risk difference (12% benefit to the experimental group), variance determined such that the 97.5th percentile = no difference), and sceptical prior N (0, 0.29030801) (no benefit, same variance as the enthusiastic prior).

**Additional details of statistical analysis**

In analyses of efficacy, children who received rescue treatment were censored at the time of starting rescue treatment, and inverse probability of (change from) treatment weights (IPTW) were used to adjust for use of rescue treatment: that is, comparable children who did not start rescue treatment (as estimated by a logistic model for starting rescue treatment) were upweighted to effectively replace those censored. This is a more principled version of a standard per-protocol analysis (which introduces bias as it relies on post-randomisation exclusion of trial participants).^5^ If the date of starting rescue treatment was the same as the date of an echocardiogram, data from that scan was not censored, assuming that rescue treatment was most likely started as a result of the scan. This analysis also adjusted for baseline Z-score using three categories: missing (as not all patients had an echocardiogram at baseline), below median and above median. Weights were calculated using logistic regression to determine the probability of receiving rescue treatment by Weeks 1, 2, 6 based on baseline characteristics (age, CRP and temperature at screening, baseline Z-score (categorised as above), country). The regression also included prior CRP and temperature values to adjust for post-baseline differences. For Week-1 these were the Day-2 and Day-5 values, if rescue treatment had not started before these dates. If rescue treatment had started before these dates, values were carried forward from the last value before rescue treatment (including on the day rescue treatment started). For later weeks CRP and temperature from the two most recent scheduled visits were used, with last observation carried forward for missing data. Results are also presented from the GEE with weights based on baseline data only, and an unweighted model (data not shown; results very similar).

**Additional details of health economics analysis**

The main QALY analysis used a random forest approach with the fit transform method MissForest (v4.2.3) to impute missing CHU-9D scores (supplementary table 17a). Less than 20% of CHU-9D were missing at each timepoint other than at the week 2 follow-up where 36% of children had missing CHU-9D. Sensitivity analyses estimated QALY differences between randomized groups using mixed effects models on all observed data, and in complete cases with CHU-9D scores at every visit (supplementary table 17b).

**References:**

1. de Graeff N, Groot N, Brogan P et al. European consensus-based recommendations for the diagnosis and treatment of rare paediatric vasculitides - the SHARE initiative. *Rheumatology (Oxford).* 2019;**58**, 656-671.
2. Brogan P, Naden R, Ardoin SP et al.. The pediatric glucocorticoid toxicity index. *Semin Arthritis Rheum*. 2022;**56**, 152068.
3. Parmar, M.K., Sydes, M.R., Morris, T.P. How do you design randomised trials for smaller populations? A framework. *BMC Med* 2016; **14**, 183.
4. Clements MN, White IR, Copas AJ, Cornelius V, Cro S, Dunn DT, Quartagno M, Turner RM, Tweed CD, Walker AS. Improving clinical trial interpretation with ACCEPT analyses. *NEJM Evid.* 2022;**1**:evidctw2200018.
5. Hernán MA, Robins JM. Per-Protocol Analyses of Pragmatic Trials. *N Engl J Med.* 2017; 377, 1391-1398.

**Supplementary tables**

| **Day** | **Temperature** | **CRP** | **Treatment Plan** |
| --- | --- | --- | --- |
| Day 2 | <38°C | ≤10 mg/L | No further treatment required; reassess on Day 5. Reduce aspirin to 3–5 mg/kg/day when afebrile ≥48h and continue for at least 21 days after fever^a^ |
| Day 2 | <38°C | >10 mg/L but ≤50% of baseline | No further treatment required; reassess on Day 5. Reduce aspirin as above^a^ |
| Day 2 | <38°C | >10 mg/L and >50% of baseline | Administer second IVIG dose. Reduce aspirin as above^a^ |
| Day 2 | ≥38°C | ≤10 mg/L | Administer second IVIG dose. Continue aspirin at 40 mg/kg/day until afebrile. |
| Day 2 | ≥38°C | >10 mg/L but ≤50% of baseline | Administer second IVIG dose. Continue aspirin at 40 mg/kg/day until afebrile. |
| Day 2 | ≥38°C | >10 mg/L and >50% of baseline | Administer second IVIG dose. Continue aspirin at 40 mg/kg/day until afebrile. |
| Day 5 | <38°C | ≤10 mg/L | Continue aspirin at 3–5 mg/kg/day for ≥21 days after fever resolution.* No further treatment required. |
| Day 5 | <38°C | >10 mg/L | Consider rescue treatment at investigator’s discretion. Continue aspirin at 3–5 mg/kg/day for ≥21 days after fever resolution* |
| Day 5 | ≥38°C | ≤10 mg/L | Consider rescue treatment at investigator’s discretion. Continue aspirin at 40 mg/kg/day until afebrile. |
| Day 5 | ≥38°C | >10 mg/L | Consider rescue treatment at investigator’s discretion. Continue aspirin at 40 mg/kg/day until afebrile. |

* Treatments administered according to local standard of care.

**Supplementary table 1. Management plan for participants in the control group based on temperature and C-reactive protein (CRP) levels at Day 2 and Day 5 post-randomisation.** Management included decisions regarding second intravenous immunoglobulin (IVIG) dosing, potential rescue treatment, and adjustments to aspirin therapy. CRP values were interpreted in relation to baseline measurements. Rescue treatments were chosen by the local investigator in line with their centres’ standard of care.

| **Day** | **Temperature** | **CRP** | **Treatment Plan** |
| --- | --- | --- | --- |
| Day 2 | <38°C | ≤10 mg/L | Start corticosteroid taper: prednisolone 1 mg/kg/day for 5 days, then 0.5 mg/kg/day for 5 days, then stop. Continue aspirin at 3–5 mg/kg/day for ≥21 days after fever resolution.^a^ |
| Day 2 | <38°C | >10 mg/L | Continue prednisolone 2 mg/kg/day until afebrile and CRP ≤10 mg/L, then taper. Consider rescue treatment at investigator’s discretion. Continue aspirin at 3–5 mg/kg/day for ≥21 days after fever resolution^a^ |
| Day 2 | ≥38°C | ≤10 mg/L | Continue prednisolone 2 mg/kg/day until afebrile and CRP ≤10 mg/L, then taper. Consider rescue treatment at investigator’s discretion. Continue aspirin at 40 mg/kg/day until afebrile. |
| Day 2 | ≥38°C | >10 mg/L | Continue prednisolone 2 mg/kg/day until afebrile and CRP ≤10 mg/L, then taper. Consider rescue treatment at investigator’s discretion. Continue aspirin at 40 mg/kg/day until afebrile. |
| Day 5 | <38°C | ≤10 mg/L | Continue taper as above if already initiated. Maintain aspirin at 3–5 mg/kg/day for ≥21 days after fever resolution*. No further treatment required. |
| Day 5 | <38°C | >10 mg/L | Continue prednisolone 2 mg/kg/day if taper not yet initiated. Consider rescue treatment at investigator’s discretion. Maintain aspirin at 3–5 mg/kg/day for ≥21 days after fever resolution*. |
| Day 5 | ≥38°C | ≤10 mg/L | Continue prednisolone 2 mg/kg/day if taper not yet initiated. Consider rescue treatment at investigator’s discretion. Continue aspirin at 40 mg/kg/day until afebrile. |
| Day 5 | ≥38°C | >10 mg/L | Continue prednisolone 2 mg/kg/day if taper not yet initiated. Consider rescue treatment at investigator’s discretion. Continue aspirin at 40 mg/kg/day until afebrile. |

* Treatments administered according to local standard of care.

**Supplementary table 2.** **Management plan for participants in the** e**xperimental group, based on temperature and C-reactive protein (CRP) levels at Day 2 and Day 5 post-randomisation.** Prednisolone was tapered only after the participant was both afebrile and had a CRP ≤10 mg/L. The taper regimen was 1 mg/kg/day for 5 days followed by 0.5 mg/kg/day for 5 days, then stopped. Rescue treatments were chosen by the local investigator in line with their centres’ standard of care.

| **Treatment** | **Total (n=103)** | **Experimental group (n=50)** | **Control group (n=53)** | **p-value*** |
| --- | --- | --- | --- | --- |
| **IVIG** |  |  |  |  |
| Received IVIG - n (%) | 103 (100%) | 50 (100%) | 53 (100%) | 1.00 |
| Started IVIG before randomisation - n (%) | 84 (82%) | 42 (84%) | 42 (79%) | 0.53 |
| Hours from starting IVIG to randomisation (if started before) - median (IQR) | 17 (13, 20) | 17 (13, 20) | 17 (14, 21) | 0.69 |
| IVIG dose prescribed at randomisation - median (IQR), g/kg | 2.0 (2.0, 2.0) | 2.0 (2.0, 2.0) | 2.0 (2.0, 2.0) | 0.33 |
| Received second dose of IVIG - n (%) | 29 (28%) | 9 (18%) | 20 (38%) | 0.021** |
| Hours from randomisation to second dose of IVIG (among those receiving it) - median (IQR) | 47 (44, 61) | 47 (38, 105) | 48 (44, 55) | 0.56 |
| Hours between first and second dose of IVIG if received - median (IQR) | 57 (48, 72) | 55 (48, 112) | 57 (47, 68) | 0.56 |
| Second IVIG dose - median (IQR), g/kg | 2.0 (1.9, 2.0) | 2.0 (1.8, 2.0) | 2.0 (1.9, 2.0) | 0.16 |
| **Aspirin** |  |  |  |  |
| Received aspirin - n (%) | 103 (100%) | 50 (100%) | 53 (100%) | 1.00 |
| Aspirin dose at randomisation - median (IQR), mg/kg/day | 39.4  (37.0, 40.0) | 39.7  (37.2, 40.0) | 39.4  (37.0, 40.0) | 0.77 |
| Days to aspirin dose reduction - median (IQR) | 3 (2, 4) | 3 (2, 3) | 3 (3, 4) | 0.080 |

*P-values from chi-squared tests (binary) or Wilcoxon rank-sum tests (continuous) unless otherwise specified.
** Secondary efficacy outcome: P-value from logistic regression adjusted for age and sex, see Supplementary Table 4.

**Supplementary table 3. Use of intravenous immunoglobulin (IVIG) and aspirin.**

| **Outcome** | **Experimental group (n=50)** | **Control group (n=53)** | **Adjusted difference between groups on modelled scale* (95% CI)** | **p-value (adjusted for age and sex)** |
| --- | --- | --- | --- | --- |
| Received rescue treatment - n (%) | 8 (16%) | 17 (32%) | -16% (-32, -0) | 0.044 |
| Received second dose of IVIG - n (%) | 9 (18%) | 20 (38%) | -20% (-37, -3) | 0.021 |
| Mean maximum z-score (95% CI)*  Week 1  Week 2  Week 6  Week 12 | 0.9 (0.5, 1.2)  0.8 (0.4, 1.3)  0.2 (-0.1, 0.5)  0.1 (-0.2, 0.4) | 1.0 (0.6, 1.3)  0.6 (0.2, 1.1)  0.4 (0.1, 0.8)  0.7 (0.4, 1.1) | -0.0 (-0.3, 0.2)  0.1 (-0.2, 0.4)  -0.1 (-0.5, 0.2)  -0.4 (-0.7, -0.1) | 0.69  0.61  0.37  0.011 |
| Days of fever after enrolment - median (IQR) | 1 (1, 1) | 1 (1, 2) | 1.4 (1.1, 1.7) | 0.00062 |
| Mean CRP (mg/L) (95% CI)*  Day 1  Day 2  Day 3  Day 4  Day 5  Week 1  Week 2  Mean CRP across day 1 - week 2 | 80 (71, 90)  42 (36, 50)  25 (20, 30)  18 (14, 23)  10 (8, 12)  3 (3, 5)  2 (1, 3)  13 (12, 14) | 73 (64, 83)  47 (40, 56)  31 (26, 37)  20 (16, 26)  11 (9, 15)  4 (3, 6)  2 (1, 3)  15 (14, 16) | 0.1 (-0.1, 0.4)  -0.2 (-0.5, 0.2)  -0.3 (-0.7, 0.1)  -0.2 (-0.7, 0.3)  -0.2 (-0.8, 0.3)  -0.4 (-1.0, 0.2)  -0.0 (-0.8, 0.7)  -0.2 (-0.4, 0.0) | 0.33  0.37  0.10  0.43  0.34  0.20  0.94  0.059 |
| Days to CRP normalisation (≤10 mg/L) - median (IQR) | 6 (4, 8) | 6 (4, 9) | 1.2 (0.8, 1.7) | 0.39 |
| Days of hospitalisation - median (IQR) | 4 (3, 6) | 5 (4, 6) | 1.1 (0.8, 1.6) | 0.46 |

***** Z-scores transformed using ln (x + 1.40), CRP transformed using log2. Modelled difference presented on the transformed scale: mean values are back-transformed for interpretability. Adjusted differences for duration of fever and hospitalisation, and time to CRP normalisation, are hazard ratios from Cox regression (>1 means faster time in experimental group).

**Supplementary table 4. Secondary efficacy outcomes.** Details of IVIG in Supplementary table 3 and of rescue treatment in Supplementary table 5.

| **(a) Rescue treatment received** | **Total (n=103)** | **Experimental group (n=50)** | **Control group (n=53)** | **p-value^**^** |
| --- | --- | --- | --- | --- |
| Received rescue treatment - n (%) | 25 (24%) | 8 (16%) | 17 (32%) | 0.044^†^ |
| Days from randomisation to starting rescue treatment (among those starting rescue treatment) - median (IQR) | 3 (2, 5) | 4 (2, 12) | 2 (1, 4) | 0.13^‡^ |
| **Type of rescue treatment*** |  |  |  |  |
| Retreatment with IVIG | 7 (7%) | 4 (8%) | 3 (6%) | 0.71 |
| IV methylprednisolone | 15 (15%) | 6 (12%) | 9 (17%) | 0.47 |
| Starting/continuing oral prednisolone | 16 (16%) | 3 (6%) | 13 (25%) | 0.013 |
| Infliximab | 6 (6%) | 4 (8%) | 2 (4%) | 0.43 |
| Ciclosporin | 1 (1%) | 1 (2%) | 0 (0%) | 0.49 |
| IL-1 blockade therapy | 3 (3%) | 1 (2%) | 2 (4%) | 1.00 |
| **Reasons for rescue treatment** |  |  |  |  |
| Planned as per protocol on day 5 | 3 (3%) | 1 (2%) | 2 (4%) | 0.10 |
| Clinical decision before day 5 | 18 (17%) | 4 (8%) | 14 (26%) |  |
| Clinical decision after day 5 | 4 (4%) | 3 (6%) | 1 (2%) |  |

* Some children received multiple types of rescue treatment: percentage is of those randomised. ^**^ P-values from chi-squared test, or Fisher’s exact test where <5 in a group, unless otherwise specified. ^†^ Secondary efficacy outcome: P-value from logistic regression, adjusted for age and sex (supplementary table 4). ^‡^ P-value from Wilcoxon rank-sum test

| **(b) Rescue treatment doses** | **Experimental group** | | | **Control group** | | |
| --- | --- | --- | --- | --- | --- | --- |
|  | N | Dose (mg/kg/day)§ | Days of treatment given § | N | Dose (mg/kg/day) § | Days of treatment given § |
| Prednisolone | 3 | 1.2 (0.6, 2.0) | 26 (1, 39) | 13 | 1.1 (0.9, 1.3) | 14 (13, 16) |
| Methylprednisolone | 6 | 11.0 (10.0, 28.6) | 3 (3, 3) | 9 | 2.2 (1.6, 9.9) | 3 (3, 4) |
| IVIG | 4 | 1.8 (1.7, 2.1) | 1 (1, 1) | 3 | 1.9 (1.9, 2.1) | 1 (1, 1) |
| Infliximab | 4 | 5.8 (5.4, 5.9) | 2 (1, 3) | 2 | 4.8, 5.4 | 1, 4 |
| Ciclosporin | 1 | 9.3 | 61 |  |  |  |
| IL-1 blockade therapy | 1 | 7.1 | 63 | 2 | 5.2, 5.9 | 10, 14 |

§ Median (IQR).

**Supplementary table 5. Rescue treatments (a) received (b) doses.**

|  | **Total (n=103)** | **Experimental group (n=50)** | **Control group (n=53)** |
| --- | --- | --- | --- |
| Received corticosteroids - n (%) | 66 (64%) | 50 (100%) | 16 (30%) |
| Days to first dose of corticosteroids* - median (IQR) | 0 (0, 1) | 0 (0, 0) | 2.5 (1.5, 4.5) |
| First dose of corticosteroids* - median (IQR), mg/kg/day | 2.0 (1.6, 2.0) | 2.0 (1.6, 2.0) | 2.0 (1.8, 2.2) |
| Days from corticosteroid start to first dose reduction* - median (IQR) | 6 (5, 7) | 6 (5, 7) | 6 (4, 10) |
| Days from first to second dose reduction* - median (IQR) | 5 (5, 5) | 5 (5, 5) | 5 (5, 8) |
| Total days of corticosteroid therapy* - median (IQR) | 16.5 (15, 20) | 17 (16, 20) | 15 (11.5, 20) |
| Cumulative weight adjusted dose of corticosteroids received* - median (IQR, mg/kg) | 19.6 (17.2, 24.8) | 19.9 (18.3, 24.1) | 18.0 (15.9, 27.3) |
| Patients on prednisolone ≥2mg/kg/day beyond day 5* - n (%)** | 31 (69%) | 31 (69%) | N/A |

***** of those who received corticosteroid
** excluding 5 patients who received rescue treatment prior to or on day 5. For reporting, the dose of methylprednisolone was converted to the equivalent dose of prednisolone by multiplying by 1.25 (expressed in mg/kg).

**Supplementary table 6. Corticosteroid use.**

|  | Experimental (N=50) | Control (N=53) |
| --- | --- | --- |
| Week 1 |  |  |
| Echocardiogram result available | 50 (100%) | 50 (94%) |
| In follow-up but no echocardiogram result available | 0 (0%) | 3 (6%) |
| Lost to follow-up or withdrawn | 0 (0%) | 0 (0%) |
|  |  |  |
| Week 2 |  |  |
| Echocardiogram result available | 49 (98%) | 48 (91%) |
| In follow-up but no echocardiogram result available | 1 (2%) | 5 (9%) |
| Lost to follow-up or withdrawn | 0 (0%) | 0 (0%) |
|  |  |  |
| Week 6 |  |  |
| Echocardiogram result available | 50 (100%) | 51 (96%) |
| In follow-up but no echocardiogram result available | 0 (0%) | 1 (2%) |
| Lost to follow-up or withdrawn | 0 (0%) | 1 (2%) |
|  |  |  |
| Week 12 |  |  |
| Echocardiogram result available | 47 (94%) | 49 (92%) |
| In follow-up but no echocardiogram result available | 2 (4%) | 1 (2%) |
| Lost to follow-up or withdrawn | 1 (2%) | 3 (6%) |

**Supplementary table 7.** Completeness of post-baseline echocardiogram data. Data are presented as N=number and (%) of participants.

|  | Experimental (N=12) | Control (N=12) |
| --- | --- | --- |
| Number of CAA criteria met: |  |  |
| 1 | 6 (50%) | 5 (42%) |
| 2 | 0 (0%) | 4 (33%) |
| 3 | 2 (17%) | 1 (8%) |
| 4 | 4 (33%) | 2 (17%) |
|  |  |  |
| Among those who had irregular contour, number of other criteria met: | Experimental (N=7) | Control (N=5) |
| 0 | 1 (14%) | 1 (20%) |
| 1 | 0 (0%) | 2 (40%) |
| 2 | 2 (29%) | 0 (0%) |
| 3 | 4 (57%) | 2 (40%) |
| Other criteria in combination with irregular contour: |  |  |
| Luminal internal diameter z-score of ≥2.5 (secondary outcome) | 6 (86%) | 3 (60%) |
| CAA defined by absolute luminal diameter (> 3 mm if < 5 years; >4 mm if ≥ 5 years) | 5 (71%) | 3 (60%) |
| Internal diameter of a segment at least 1.5 times that of an adjacent segment | 5 (71%) | 2 (40%) |

**Supplementary table 8.** Summary of CAA criteria met in experimental and control groups. Data are presented as N=number and (%) of participants meeting CAA criteria.

| **Maximum coronary z score^*^** | **Experimental group (n=50)** | **Control group (n=53)** |
| --- | --- | --- |
| <2.5 | 42 (84%) | 43 (81%) |
| ≥ 2.5-<5^a^ | 4 (8%) | 5 (9%) |
| ≥ 5-<10^b^ | 0 (0%) | 4 (8%) |
| ≥ 10^c^ | 4 (8%) | 1 (2%) |

* Maximum z score of Right coronary artery (RCA) or left anterior descending (LAD). The protocol did not define aneurysm severity based on Z score, but these Z scores equate to a. Small; b. Medium; and c. Large/giant CAA.

**Supplementary table 9. Exploratory (post hoc) analysis of CAA severity based on z-scores.** Odds ratio (experimental vs control) = 0.83 (95% CI 0.29-2.43) p=0.74 from ordinal logistic regression adjusted for age and sex.

| **Subgroup** | | **Experimental group (n=50)** | **Control group (n=53)** | **Adjusted difference between groups (95% CI)** | **Inter-action p-value** |
| --- | --- | --- | --- | --- | --- |
| **CAA (binary co-primary outcome: n/N (%) for categorical variables, estimated percentage with 95% CI for continuous variables)** | | | | | |
| Age | <1 | 4/6 (67%) | 2/6 (33%) | 31.9% (-19.0, 82.7) | 0.23 |
|  | ≥1 | 8/44 (18%) | 10/47 (21%) | -3.0% (-19.4, 13.3) |  |
| Sex | Male | 9/29 (31%) | 10/31 (32%) | -1.5% (-24.2, 21.2) | 0.60 |
|  | Female | 3/21 (14%) | 2/22 (9%) | 5.5% (-14.9, 25.9) |  |
| Days of fever at randomisation (continuous) | Day 5 | 28.1% (8.5, 47.6) | 29.7% (9.7, 49.8) | -1.7% (-29.8, 26.4) | 0.78 |
|  | Day 10 | 19.3% (0.3, 38.2) | 15.1% (-1.5, 31.7) | 4.1% (-21.1, 29.4) |  |
| Baseline z-score | Missing | 4/20 (20%) | 9/25 (36%) | N/A non-convergence due to perfect prediction | |
|  | Below median | 5/19 (26%) | 0/10 (0%) |  |  |
|  | Above median | 3/11 (27%) | 3/18 (17%) |  |  |
| Baseline z-score (alternate categorisation) | Missing | 4/20 (20%) | 9/25 (36%) | N/A non-convergence due to perfect prediction | |
|  | <2.5 | 7/29 (24%) | 2/27 (7%) |  |  |
|  | ≥2.5 | 1/1 (100%) | 1/1 (100%) |  |  |
| **Maximum z-score over weeks 1, 2 and 6 (continuous co-primary endpoint: mean with 95% CI)** | | | | | |
| Age | <1 | 0.4 (-0.6, 2.3) | 1.3 (0.8, 2.0) | -0.4 (-1.2, 0.4) | 0.29 |
|  | ≥1 | 0.6 (0.4, 0.8) | 0.6 (0.4, 0.9) | -0.0 (-0.2, 0.2) |  |
| Sex | Male | 0.6 (0.4, 0.9) | 0.7 (0.4, 1.0) | -0.0 (-0.2, 0.2) | 0.99 |
|  | Female | 0.5 (0.1, 1.1) | 0.6 (0.2, 1.1) | -0.0 (-0.4, 0.3) |  |
| Days of fever at randomisation (continuous) | Day 5 | 0.8 (0.4, 1.2) | 0.8 (0.4, 1.3) | -0.0 (-0.3, 0.3) | 0.75 |
|  | Day 10 | 0.4 (0.2, 0.8) | 0.6 (0.3, 1.0) | -0.1 (-0.3, 0.2) |  |
| Baseline z-score | Missing | 0.5 (0.2, 1.0) | 0.7 (0.4, 1.1) | -0.1 (-0.4, 0.2) | 0.78 |
|  | Below median | 0.4 (0.1, 0.6) | 0.5 (-0.0, 1.3) | -0.1 (-0.5, 0.3) |  |
|  | Above median | 1.1 (0.3, 2.2) | 0.9 (0.6, 1.3) | 0.1 (-0.3, 0.5) |  |
| Baseline z-score (alternate categorisation) | Missing | 0.6 (0.2, 1.0) | 0.7 (0.4, 1.1) | -0.1 (-0.3, 0.2) | 0.98 |
|  | <2.5 | 0.5 (0.3, 0.9) | 0.7 (0.4, 1.2) | -0.1 (-0.4, 0.1) |  |
|  | ≥2.5 | 0.7 (0.5, 1.1) | 0.9 (0.6, 1.3) | -0.1 (-0.1, -0.1) |  |

**Supplementary table 10. Subgroup analyses of co-primary outcomes.** Differences between groups adjusted for age, sex, days of fever at randomization and baseline z-scores. Continuous Z-scores also adjusted for receipt of rescue treatment using IPTW. The interaction p-value tests for differential treatment effects across subgroups.

| **Outcome** | **Experimental group (n=50): Mean change from baseline (95% CI)** | **Control group (n=53): Mean change from baseline (95% CI)** | **Adjusted difference between groups (95%CI)** | **p-value** |
| --- | --- | --- | --- | --- |
| Haemoglobin (g/L)  Day 2  Day 5  Week 2  Week 6  Mean across day 2 - week 6 | -2 (-5, 1)  4 (0, 8)  8 (5, 10)  14 (12, 17)  6 (4, 7) | -3 (-6, -0)  -2 (-5, 2)  5 (2, 7)  13 (10, 15)  3 (2, 4) | 1 (-3, 5)  6 (0, 11)  3 (-1, 7)  2 (-2, 5)  3 (1, 5) | 0.69  0.038  0.12  0.39  0.0089 |
| White cell count (x10^9/L)*  Day 2  Day 5  Week 2  Week 6  Mean across day 2 - week 6 | 0.9 (0.8, 1.0)  1.4 (1.2, 1.6)  0.9 (0.8, 1.0)  0.7 (0.7, 0.8)  1.0 (0.9, 1.0) | 0.8 (0.7, 0.9)  0.9 (0.8, 1.0)  0.7 (0.6, 0.8)  0.7 (0.6, 0.8)  0.8 (0.7, 0.8) | 0.2 (-0.0, 0.5)  0.7 (0.4, 0.9)  0.4 (0.2, 0.6)  0.0 (-0.2, 0.2)  0.3 (0.2, 0.5) | 0.081  <0.0001  0.00064  0.73  <0.0001 |
| Platelet count (x10^9/L)  Day 2  Day 5  Week 2  Week 6  Mean across day 2 - week 6^a^ | 110 (71, 150)  314 (248, 381)  149 (88, 209)  84 (49, 120)  163 (137, 189) | 81 (44, 118)  310 (252, 369)  130 (74, 187)  29 (-8, 65)  135 (110, 159) | 29 (-25, 84)  4 (-88, 95)  18 (-65, 102)  56 (4, 108)  28 (-9, 64) | 0.29  0.94  0.66  0.036  0.13 |
| ESR (mm/hr)  Day 2  Day 5  Week 2  Week 6  Mean across day 2 - week 6^a^ | 24 (5, 42)  -3 (-21, 15)  -46 (-66, -25)  -62 (-71, -54)  -23 (-31, -15) | 17 (3, 30)  16 (3, 29)  -31 (-48, -13)  -66 (-73, -59)  -16 (-23, -10) | 7 (-17, 31)  -19 (-42, 5)  -15 (-44, 14)  4 (-8, 16)  -6 (-17, 4) | 0.55  0.11  0.29  0.49  0.23 |
| Albumin (g/L)  Day 2  Day 5  Week 2  Week 6  Mean across day 2 - week 6^a^ | -1.4 (-2.6, -0.3)  2.9 (1.3, 4.4)  8.7 (7.3, 10.1)  11.8 (10.5, 13.2)  5.4 (4.8, 6.0) | -2.0 (-3.1, -1.0)  1.9 (0.6, 3.3)  9.2 (8.0, 10.4)  12.8 (11.6, 14.0)  5.3 (4.7, 5.9) | 0.6 (-1.0, 2.2)  0.9 (-1.2, 3.1)  -0.5 (-2.5, 1.4)  -1.0 (-2.9, 0.9)  0.1 (-0.8, 0.9) | 0.46  0.39  0.59  0.28  0.89 |

***** WCL log2 transformed for analysis, all other variables analysed on the original scale. Modelled difference between groups presented on the transformed scale: mean change from baseline in experimental and control groups are back-transformed for interpretability. Differences adjusted for baseline values and stratification factors. Mean difference across Day 2- Week 6 estimated using generalised estimation equations (GEE) with independent correlation structure.

**Supplementary table 11. Changes in laboratory parameters**.

| **Patient** | **Randomisation** | **SAE criteria** | **Relatedness to corticosteroids** | **Relatedness to IVIG** | **Relatedness**  **to aspirin** | **Event** | **Age at randomisation** | **Days from randomisation** |
| --- | --- | --- | --- | --- | --- | --- | --- | --- |
| 1 | Control | Life threatening | Unrelated | Possibly | Unrelated | Myocardial depression | 5 years | 1 |
| 2 | Control | Life threatening | Unrelated | Probably | Unrelated | Haemolytic Anaemia^1^ | 3 years | 2 |
| 2 | Control | Hospitalisation | Unrelated | Unrelated | Unrelated | Fever | 3 years | 10 |
| 1 | Control | Hospitalisation | Unrelated | Unrelated | Unrelated | Aneurysm of Coronary Vessels | 5 years | 17 |
| 3 | Control | Hospitalisation (prolongation) | Unrelated | Unrelated | Unrelated | Kawasaki's disease^2^ | 1 years | 4 |
| 7 | Experimental | Hospitalisation | Unrelated | Unrelated | Unrelated | Disease progression (Kawasaki disease)^3^ | 3 months | 23 |
| 7 | Experimental | Hospitalisation | Unrelated | Unrelated | Unrelated | Disease progression (Kawasaki disease)^3^ | 3 months | 59 |
| 9 | Experimental | Hospitalisation | Unlikely | Unrelated | Unrelated | Disease progression (Kawasaki disease)^4^ | 2 months | 15 |
| 4 | Experimental | Hospitalisation | Unrelated | Unrelated | Unrelated | Coronary Artery Aneurysm | 5 years | 14 |
| 5 | Experimental | Hospitalisation | Unrelated | Unrelated | Unrelated | Parvovirus B19 infection | 4 years | 22 |
| 6 | Experimental | Hospitalisation | Unrelated | Unrelated | Unlikely | Vomiting | 4 years | 27 |
| 7 | Experimental | Hospitalisation | Unrelated | Unlikely | Unlikely | Upper respiratory tract infection | 3 months | 13 |
| 8 | Experimental | Hospitalisation | Unlikely | Unlikely | Unlikely | Tachypnoea | 2 months | 18 |
| 8 | Experimental | Hospitalisation | Possibly | Unlikely | Unlikely | Infection susceptibility increased^5^ | 2 months | 24 |
| 9 | Experimental | Hospitalisation | Probably | Unrelated | Unrelated | Fever | 2 months | 75 |
| 10 | Experimental | Other important medical condition | Unrelated | Unrelated | Unrelated | Staphylococcus aureus test positive (carriage) | 2 years | 0 |

**Supplementary table 12. Serious Adverse Events (SAEs) by participant.** Each row represents a unique SAE, identified by participant number, as reported by the local investigators. Relatedness to study drugs (prednisolone, IVIG, and aspirin) based on investigator assessment and categorised as definitely, probably, possibly, unlikely, or unrelated. ^1^Following second dose of IVIG; ^2^ Patient had ongoing hyperinflammation as indicated by persistently raised CRP; ^3^Patient had both high CRP and fever and during a second admission continued to have high CRP and irritability; ^4^ Patient already had a CAA previously but readmitted with rising CRP and worsening z-score; ^5^The main diagnosis given by the site was increased work of breathing, changed at clinical review to “infection susceptibility increased” as the cause of increased work of breathing was an upper respiratory tract infection possibly related to prednisolone/methylprednisolone.

| **Patient** | **Randomisation** | **Event description** | **Grade** | **Relatedness to corticosteroids** | **Relatedness to IVIG** | **Relatedness to aspirin** | **Age at randomisation** | **Days from randomisation** |
| --- | --- | --- | --- | --- | --- | --- | --- | --- |
| 1 | Control | Low mood | 1 | Not taken | Possibly | Unlikely | 7 years | 10 |
| 1 | Control | Nausea | 1 | Not taken | Possibly | Unlikely | 7 years | 10 |
| 1 | Control | Leg pain | 1 | Not taken | Possibly | Unlikely | 7 years | 5 |
| 1 | Control | Nosebleed | 1 | Not taken | Not related | Possibly | 7 years | 25 |
| 1 | Control | Nosebleed | 1 | Not taken | Not related | Possibly | 7 years | 20 |
| 2 | Experimental | Aggressive behaviour | 3 | Definitely | Not related | Not related | 3 years | 5 |
| 3 | Control | Low haemoglobin | 2 | Not taken | Possibly | Unlikely | 1 years | 0* |
| 4 | Control | Infusion-related reaction | 3 | Not related | Probably | Possibly | 1 years | 0* |
| 5 | Control | Low haemoglobin | 3 | Not taken | Possibly | Not related | 1 years | 1 |
| 6 | Experimental | Low haemoglobin | 3 | Not related | Possibly | Not related | 3 months | 0* |
| 6 | Experimental | Hypertension | 3 | Possibly | Unlikely | Unlikely | 3 months | 82 |
| 7 | Control | Elevated AST | 4 | Not taken | Possibly | Unlikely | 4 years | 5 |
| 7 | Control | Elevated ALT | 3 | Not taken | Possibly | Unlikely | 4 years | 3 |
| 7 | Control | Elevated AST | 3 | Not taken | Possibly | Unlikely | 4 years | 0* |
| 8 | Experimental | High creatinine | 3 | Not related | Possibly | Possibly | 2 years | 7 |
| 8 | Experimental | Low haemoglobin | 3 | Not related | Possibly | Not related | 2 years | 1 |
| 9 | Control | High creatinine | 3 | Not related | Possibly | Not related | 7 months | 84 |
| 9 | Control | High creatinine | 3 | Not related | Possibly | Not related | 7 months | 49 |
| 9 | Control | High creatinine | 3 | Not related | Possibly | Not related | 7 months | 5 |
| 9 | Control | Low haemoglobin | 3 | Not taken | Possibly | Unlikely | 7 months | 0* |
| 10 | Control | Low haemoglobin | 3 | Not taken | Possibly | Unlikely | 1 years | 0* |
| 10 | Control | High creatinine | 3 | Not related | Possibly | Not related | 1 years | 5 |
| 11 | Experimental | Nosebleed | 1 | Unlikely | Not related | Possibly | 1 years | 10 |
| 12 | Experimental | Behavioural disturbance | 2 | Possibly | Possibly | Unlikely | 5 years | 4 |
| 13 | Control | Low haemoglobin | 3 | Not related | Probably | Not related | 3 years | 2 |
| 14 | Experimental | Elevated CRP | 2 | Probably | Not related | Not related | 2 months | 75 |
| 14 | Experimental | Low haemoglobin | 3 | Not related | Possibly | Not related | 2 months | 2 |
| 14 | Experimental | Fever | 2 | Probably | Not related | Not related | 2 months | 75 |
| 15 | Experimental | Oral thrush | 1 | Possibly | Unlikely | Unlikely | 2 months | 7 |
| 16 | Control | Elevated ALT | 2 | Not taken | Not related | Probably | 6 years | 6 |
| 16 | Control | Elevated AST | 1 | Not taken | Not related | Probably | 6 years | 6 |
| 17 | Control | Blood in urine | 3 | Not taken | Unlikely | Possibly | 4 years | 1 |

* Occurred after randomisation

**Supplementary table 13. Other adverse events definitely, probably or possibly related to corticosteroids, IVIG or aspirin.** Each row represents a unique AE, labelled by patient number (different numbers to supplementary table 10), as reported by the local investigators. CRP=C-reactive protein

|  | **Experimental**  **(N=48)** | **Control**  **(N=47)** | **Adjusted difference between arms on modelled scale** | **p-value** |
| --- | --- | --- | --- | --- |
| **Aggregate improvement score at week 12** | 0.7 (-30.6, 32.1) | -7.4 (-39.1, 24.3) | 8.1 (-37.3, 53.5) | 0.72 |
| **Cumulative worsening score at week 12** | 11.4 (6.0, 21.8) | 8.9 (4.6, 17.2) | 0.2 (-0.7, 1.2) | 0.61 |

**Supplementary table 14. pGTI.** Cumulative worsening score log-transformed prior to analysis due to non-normality. Modelled difference between groups presented on the transformed scale: mean absolute values are back-transformed for interpretability.

| **Variable** | **Experimental** | **Control** | **Difference (95% confidence interval) *** |
| --- | --- | --- | --- |
| Total N of patients | 50 | 53 |  |
| Aspirin mean cost (SD) | £14 (9) | £17 (21) | -3 (-9 to 4) |
| Prednisolone mean cost (SD) | £9 (6) | £2 (8) | 6 (4 to 9) |
| Methylprednisolone mean cost (SD) | £15 (28) | £6 (19) | 9 (-1 to 18) |
| IVIG mean cost (SD) | £360 (937) | £798 (1221) | -438 (-865 to -11) |
| Infliximab mean cost (SD) | £121 (491) | £128 (834) | -7 (-277 to 262) |
| IL-1 blockade therapy mean cost (SD) | £2 (15) | £1 (7) | 1 (-4 to 5) |
| Ciclosporin mean cost (SD) | £1 (6) | £0 (0) | 1 (-1 to 3) |
| **Total trial drug mean cost (SD)** | **£522 (1050)** | **£953 (1430)** | **-431 (-924 to 61)** |

**Supplementary table 15. Mean cost per patient for trial medication (excluding first dose of IVIG)**. N=number; SD=standard deviation; IVIG=intravenous immunoglobulin; IL-1=interelukin-1

Medication was costed based on total dose reported and based on costs obtained from the British National Formulary for Children (Joint Formulary Committee 2025 BNF for Children. London: BMJ Group and Pharmaceutical Press. Available at: https://bnfc.nice.org.uk/)

| **Variable** | **Experimental** | **Control** | **Difference (95% confidence interval) *** |
| --- | --- | --- | --- |
| Total N of patients | 50 | 53 |  |
| ***Cost of index admission*** |  |  |  |
| Inpatient mean cost (SD) | £782 (405) | £834 (367) | -52 (-203 to 98) |
| ICU mean cost (SD) | £139 (775) | £99 (718) | 41 (-251 to 333) |
| HDU mean cost (SD) | £0 (0) | £99 (531) | -99 (-248 to 50) |
| Total healthcare cost mean cost (SD) | £1182 (1070) | £1302 (1337) | -121 (-596 to 354) |
| Lost earnings mean cost (SD) | £261 (349) | £271 (344) | -10 (-146 to 125) |
| ***Costs related to KD*** |  |  |  |
| GP mean cost (SD) | £0 (0) | £0 (0) | 0 (0 to 0) |
| A&E mean cost (SD) | £10 (48) | £9 (47) | 1 (-18 to 19) |
| Outpatient mean cost (SD) | £83 (244) | £32 (142) | 51 (-27 to 128) |
| Daycase mean cost (SD) | £0 (0) | £162 (1177) | -162 (-492 to 169) |
| Inpatient mean cost (SD) | £104 (737) | £83 (606) | 21 (-242 to 284) |
| ICU mean cost (SD) | £105 (739) | £0 (0) | 105 (-97 to 306) |
| Total healthcare cost mean (SD) | £301 (1711) | £286 (1803) | 15 (-673 to 703) |
| Lost earnings mean cost (SD) | £211 (349) | £160 (420) | 51 (-100 to 203) |
| ***Non-KD costs*** |  |  |  |
| GP mean cost (SD) | £13 (36) | £19 (88) | -6 (-32 to 21) |
| A&E mean cost (SD) | £29 (93) | £23 (71) | 6 (-26 to 39) |
| Outpatient mean cost (SD) | £26 (128) | £11 (47) | 15 (-22 to 52) |
| Daycase mean cost (SD) | £24 (173) | £23 (168) | 1 (-65 to 68) |
| Inpatient mean cost (SD) | £120 (639) | £30 (154) | 90 (-89 to 269) |
| Total healthcare cost mean (SD) | £215 (844) | £108 (270) | 107 (-135 to 349) |
| All total healthcare costs mean (SD) | £1698 (2322) | £1697 (2752) | 1 (-997 to 1000) |
| All healthcare and medication costs mean (SD) | £2220 (2871) | £2650 (3714) | -430 (-1733 to 873) |

**Supplementary Table 16. Mean healthcare costs and lost earnings per participant** – imputed. N=number; SD=standard deviation; IVIG=intravenous immunoglobulin; KD=Kawasaki disease; ICU=intensive care unit; HDU=high dependency unit; GP=General practitioner; A&E=accident and emergency.

(a) Imputation using random forest

| **Variable** | **Experimental** | **Control** | **Difference (95% CI)** | **P-value** |
| --- | --- | --- | --- | --- |
| ***CHU-9D Day-0 N*** | 50 | 53 |  |  |
| CHU-9D Day-0 mean (SD) | 0.629(0.125) | 0.599(0.125) |  |  |
| ***CHU-9D week-1 N*** | 50 | 53 |  |  |
| CHU-9D week-1 mean (SD) | 0.876(0.083) | 0.885(0.087) |  |  |
| ***CHU-9D week-2 N*** | 50 | 53 |  |  |
| CHU-9D week-2 mean (SD) | 0.901(0.072) | 0.913(0.062) |  |  |
| ***CHU-9D week-6 N*** | 50 | 53 |  |  |
| CHU-9D week-6 mean (SD) | 0.928(0.07) | 0.952(0.07) |  |  |
| ***CHU-9D week-12 N*** | 50 | 53 |  |  |
| CHU-9D week-12 mean (SD) | 0.962(0.06) | 0.969(0.06) |  |  |
| ***CHU-9D total QALYs N*** | 50 | 53 |  |  |
| CHU-9D total QALYs mean (SD) | 0.219(0.011) | 0.223(0.012) | -0.004 (-0.008 to 0.000) | 0.072 |

(b) Observed data

| **Variable** | **Experimental** | **Control** | **Difference (95% CI)** | **P-value** |
| --- | --- | --- | --- | --- |
| ***CHU-9D Day-0 N*** | 38 | 44 |  |  |
| CHU-9D Day-0 mean (SD) | 0.641(0.141) | 0.597(0.137) |  |  |
| ***CHU-9D week-1 N*** | 39 | 46 |  |  |
| CHU-9D week-1 mean (SD) | 0.865(0.1) | 0.883(0.094) |  |  |
| ***CHU-9D week-2 N*** | 33 | 33 |  |  |
| CHU-9D week-2 mean (SD) | 0.9(0.088) | 0.911(0.08) |  |  |
| ***CHU-9D week-6 N*** | 42 | 47 |  |  |
| CHU-9D week-6 mean (SD) | 0.927(0.075) | 0.95(0.074) |  |  |
| ***CHU-9D week-12 N*** | 41 | 45 |  |  |
| CHU-9D week-12 mean (SD) | 0.957(0.062) | 0.962(0.064) |  |  |
| ***CHU-9D total QALYs N*** | 24 | 26 |  |  |
| CHU-9D total QALYs mean (SD) | 0.218(0.014) | 0.224(0.011) | -0.007 (-0.014 to -0.001) | 0.030 |

**Supplementary Table 17. CHU-9D (a) imputed using random forests (main analysis) (b) complete cases**. CHU-9D= Child Health Utility Index 9 dimension; N=number; SD=standard deviation; QALYs=quality adjusted life years. QALY difference using mixed effects models rather than multiple imputation -0.003 (95% CI -0.009 to 0.002) p=0.286.

**Supplementary figures.**

**
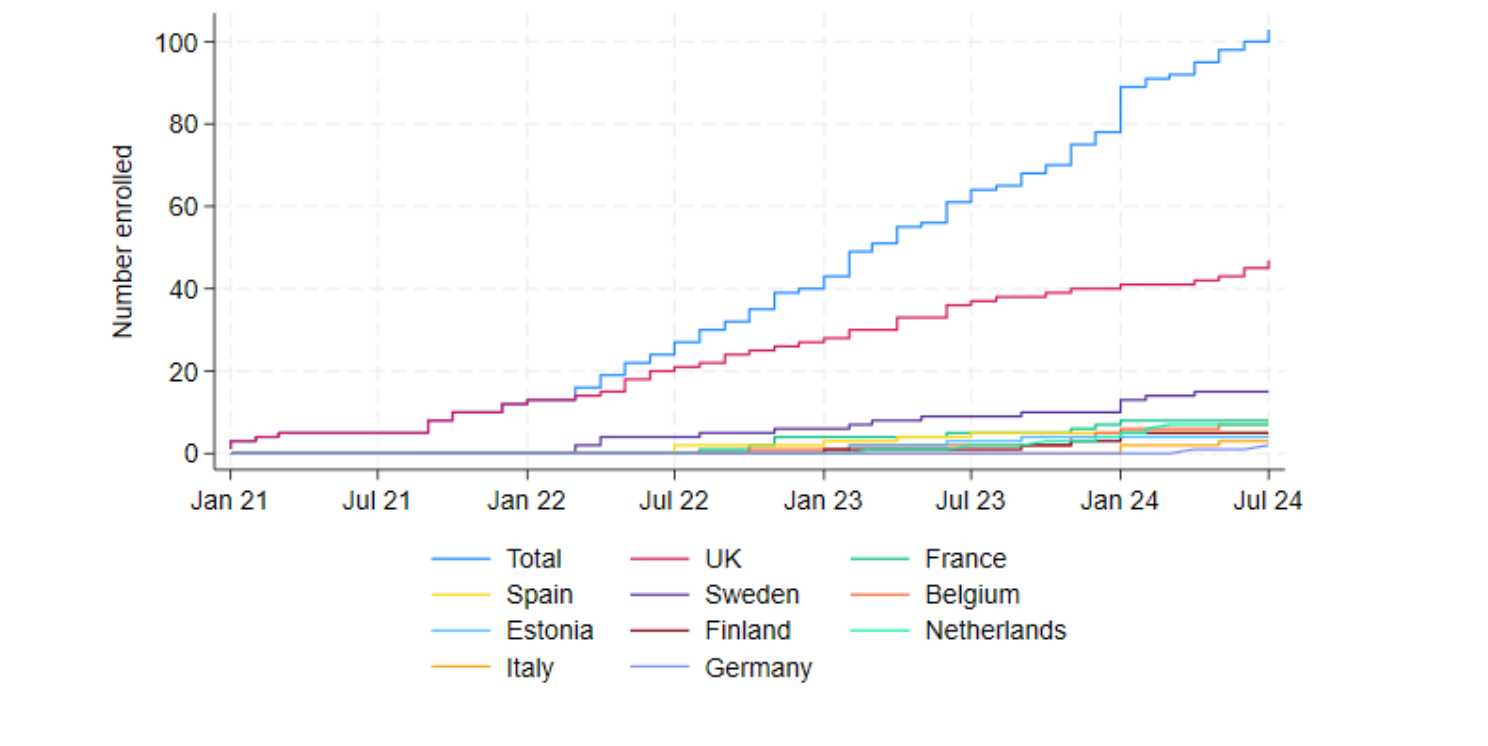
**

**Supplementary figure 1. Cumulative number of participants enrolled over time to the KD-CAAP trial, by country.**


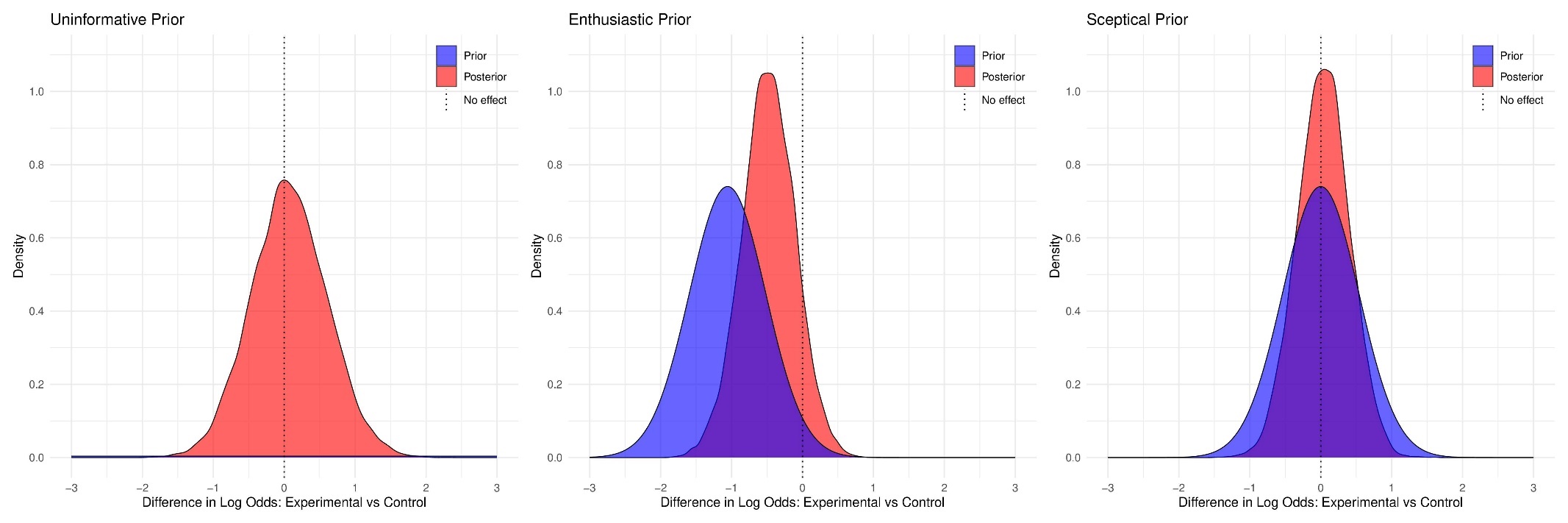


**Supplementary figure 2. Posterior distributions (red) for the difference in log odds of having a coronary artery aneurysm (CAA) between experimental and control groups under three Bayesian prior assumptions (enthusiastic, sceptical, and uninformative).** The figure displays prior (blue) and posterior (red) distributions for the difference in log odds of having a CAA in the experimental group compared to control, under three Bayesian prior assumptions. A Bayesian analysis combines the prior information (blue) with the data from the trial to produce a posterior distribution of the likely difference between the randomised arms caused by the randomised intervention. The vertical dashed line at 0 indicates no treatment effect. In each panel, negative log odds mean lower CAA rates in the experimental group, and positive log odds mean lower CAA rates in the control group. The three prior assumptions are as follows (A) An uninformative prior (very flat blue curve with very little information) yielded a posterior risk difference of 1.1% (95% CrI –13.8 to 16.1) and a 45% probability of benefit from steroids (red curve has mean 1.1% on the absolute probability scale). (B) An enthusiastic prior, assuming benefit from steroids (blue curve centred around substantial benefit from steroids with only 5% of the probability distribution to the right of 0) resulted in a posterior risk difference of –6.8% (95% CrI –17.4 to 3.8) and a 90% probability of benefit (red curve predominantly to the left of zero). (C) A sceptical prior, assuming no benefit from steroids but with much more confidence than the uninformative prior shown in (A), produced a posterior risk difference of 0.6% (95% CrI –10.1 to 11.3) with a 46% probability of benefit.

**
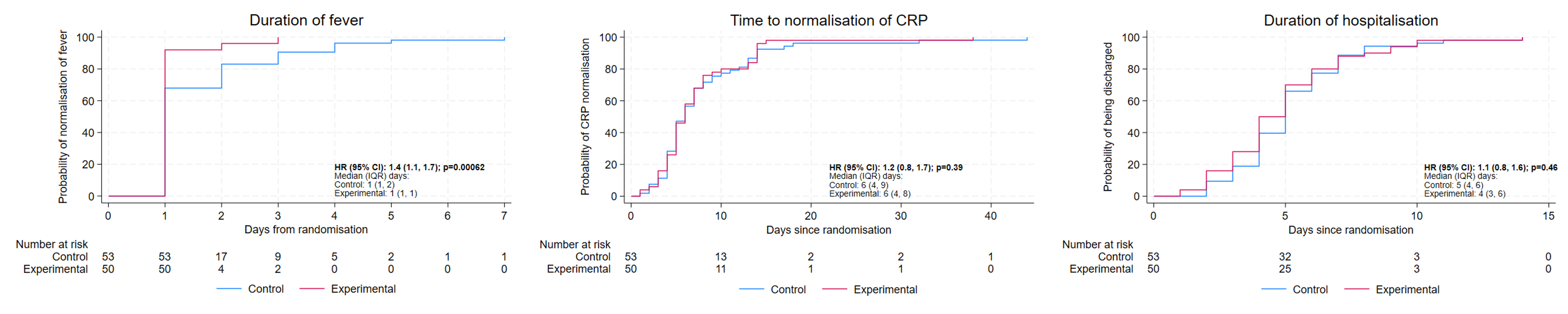
**

**Supplementary figure 3. Secondary efficacy outcomes** (A) Fever resolution (temperature <38°C). (B) CRP normalisation. (C) Duration of hospitalisation. HR=hazard ratios

**
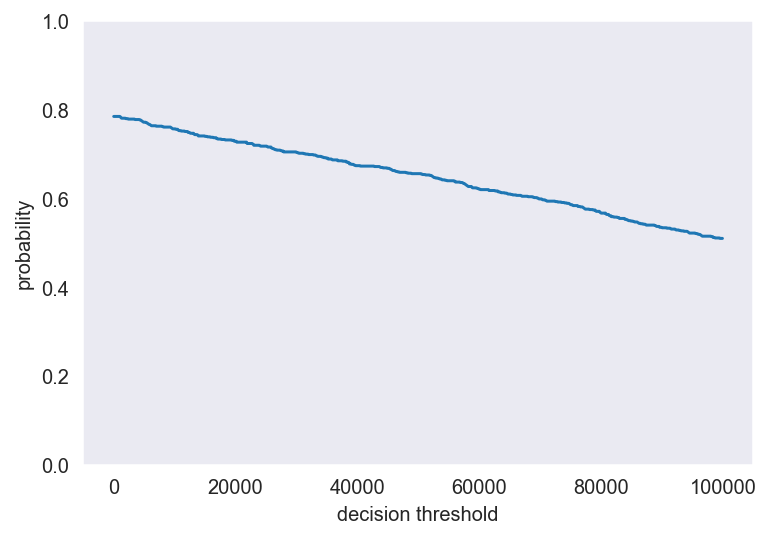
**
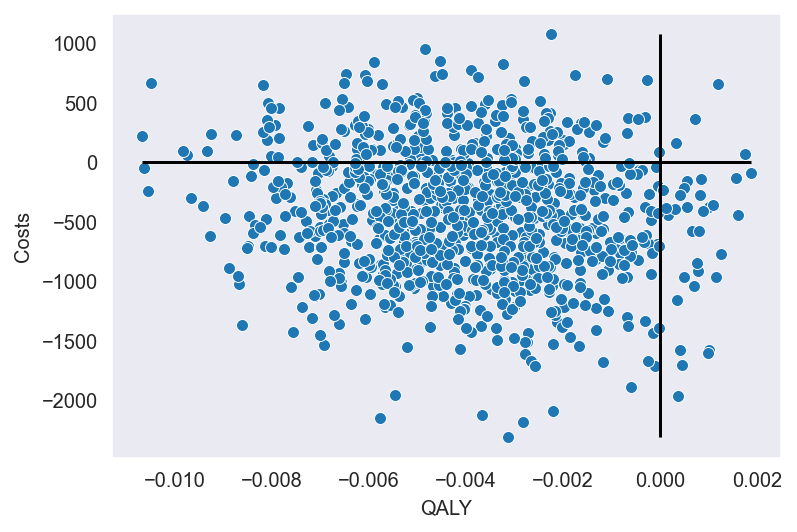


**B**

**A**

**Supplementary figure 4. Cost-effectiveness of adjunctive corticosteroids compared with standard care over 12 weeks.** (A) Cost-effectiveness plane showing incremental costs versus incremental quality-adjusted life years (QALYs) for the corticosteroid group compared with current practice. Each point represents one simulation from the probabilistic sensitivity analysis. The majority of points lie below the horizontal axis, indicating reduced costs, with little change in QALYs. (B) Cost-effectiveness acceptability curve showing the probability that adjunctive corticosteroids are cost-effective at different willingness-to-pay thresholds per QALY gained. At a threshold of £20,000 per QALY, there was approximately a 70% probability that corticosteroids were cost-effective, largely due to reduced intravenous immunoglobulin (IVIG) costs (mean difference –£438, 95% CI –£865 to –£11).
